# Supplementary figures and images for: The long noncoding RNA lnc-FANCI-2 intrinsically restricts RAS signaling in human papillomavirus type 16-infected cervical cancer cells (part 1 of 2)
Source: eLife. 2025 Aug 29;13:RP102681. doi: 10.7554/eLife.102681 (PMC12396819; doi:10.7554/eLife.102681)

Figure 2

B

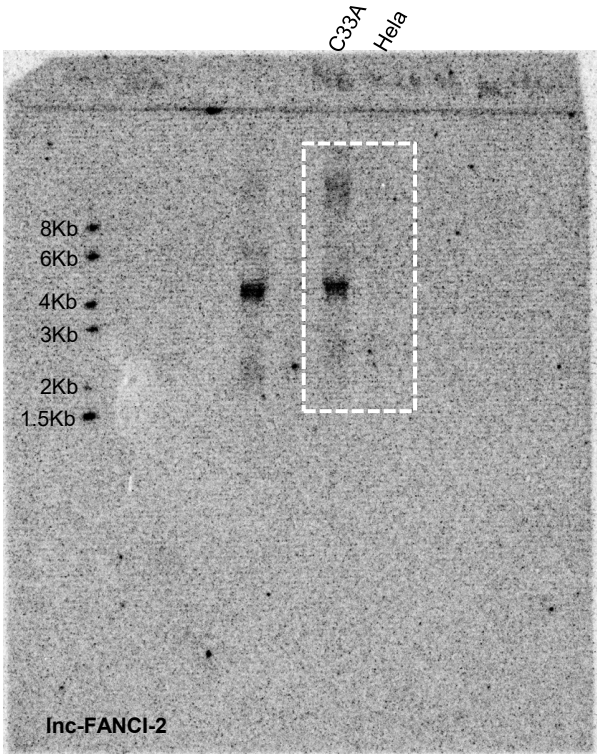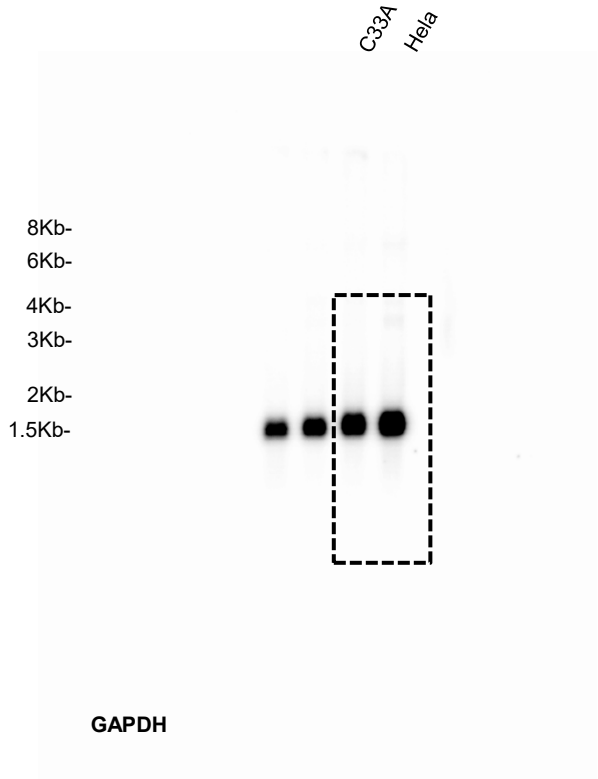

Figure 2

C

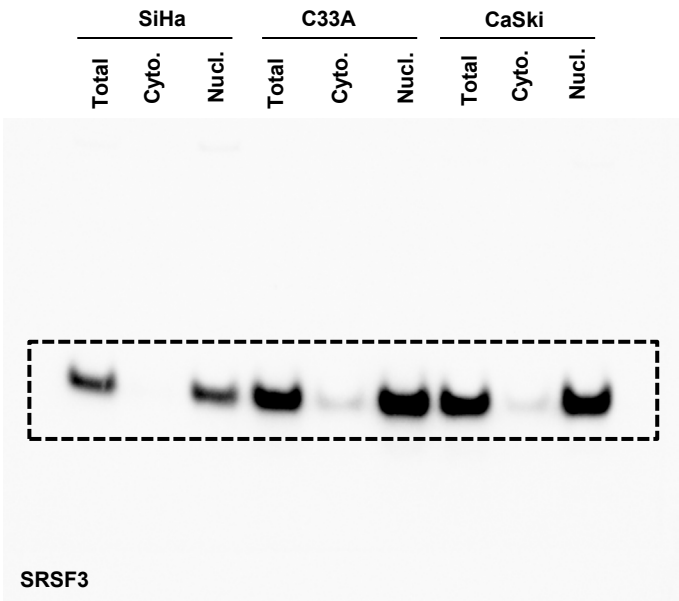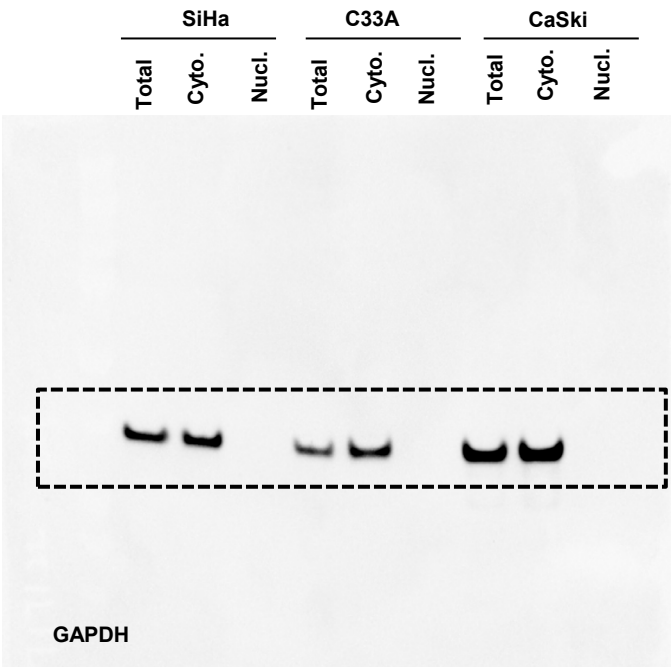

Supplement: Figure 2—source data 1. [file elife-102681-fig2-data1.pdf]

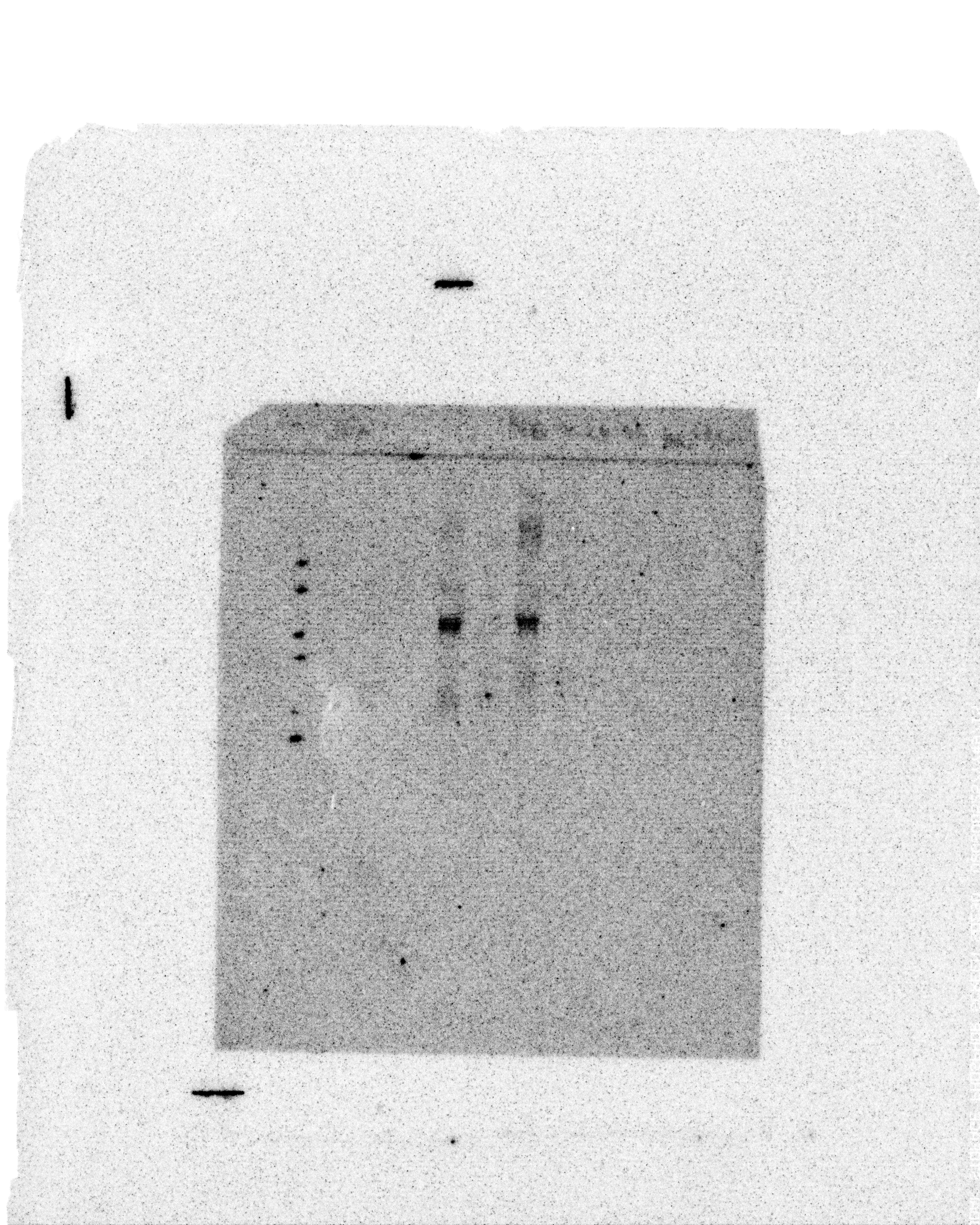

Supplement: Figure 2—source data 2. [file elife-102681-fig2-data2.zip › Figure 2-source data_2/20181004.tif]

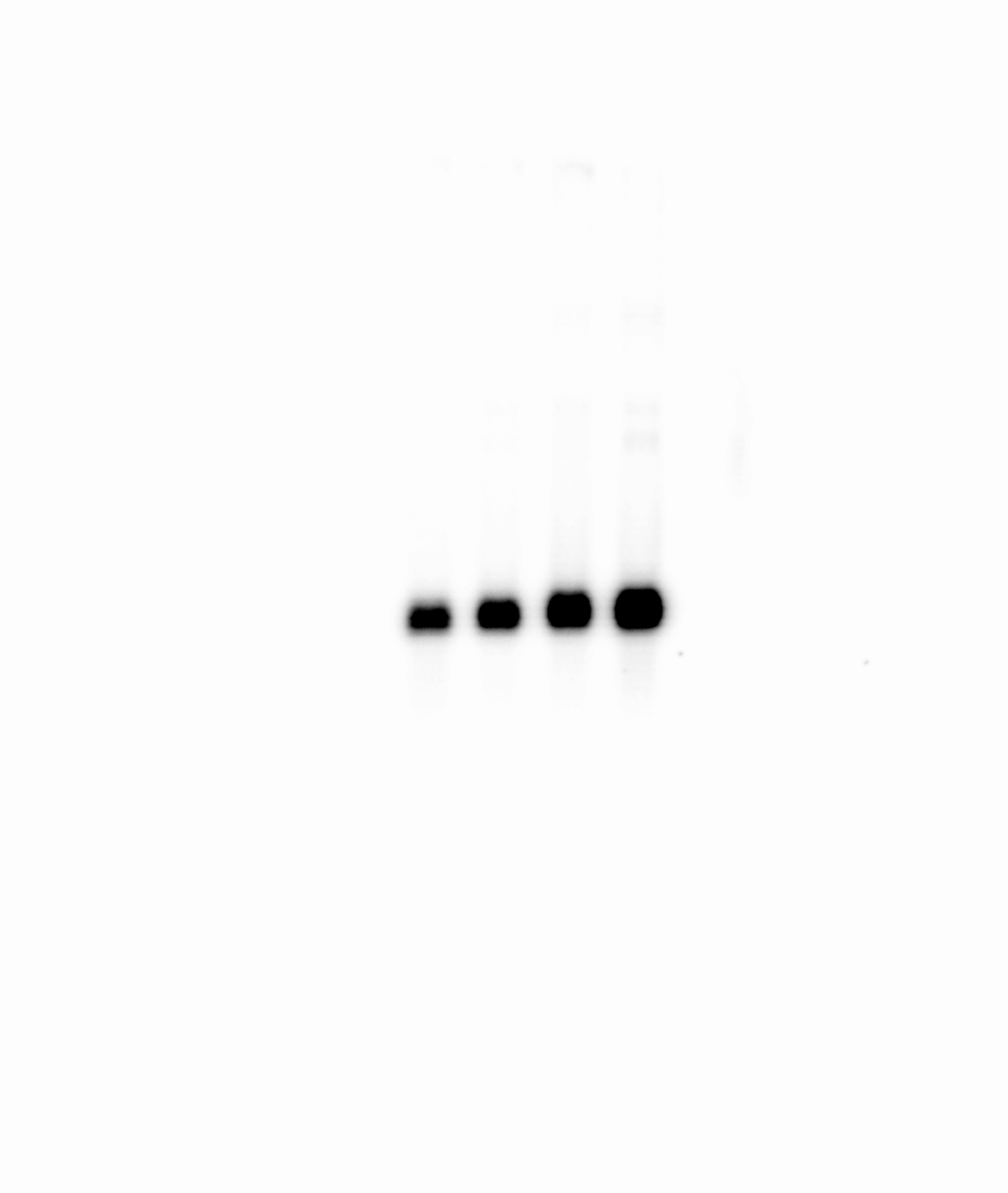

Supplement: Figure 2—source data 2. [file elife-102681-fig2-data2.zip › Figure 2-source data_2/20181009.tif]

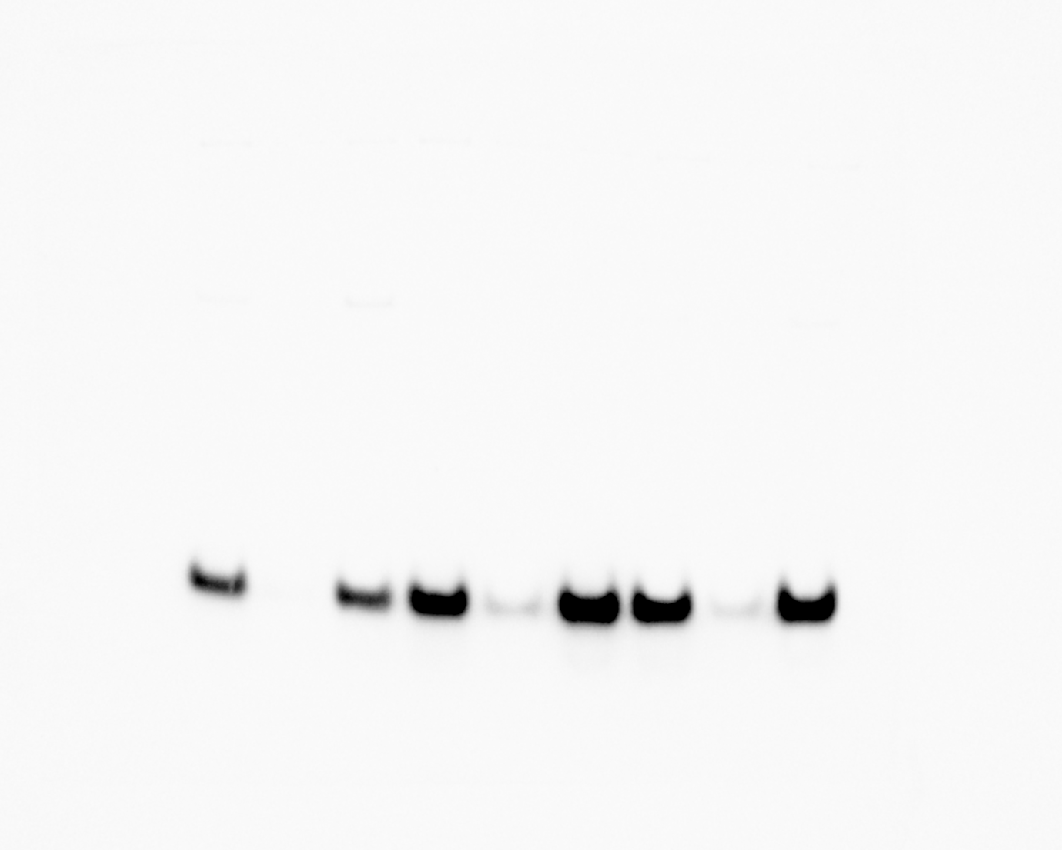

Supplement: Figure 2—source data 2. [file elife-102681-fig2-data2.zip › Figure 2-source data_2/zheng lab 2022-07-08 10h16m47s.tif]

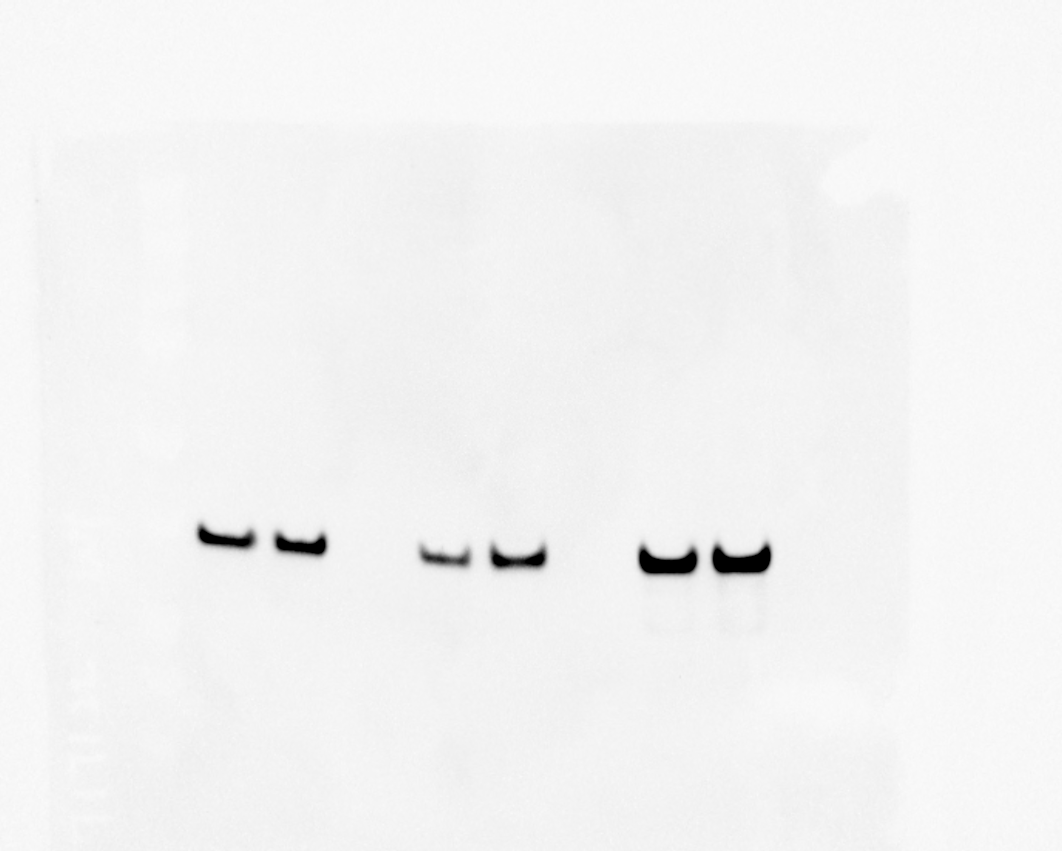

Supplement: Figure 2—source data 2. [file elife-102681-fig2-data2.zip › Figure 2-source data_2/zheng lab 2022-07-08 12h31m36s.tif]

Figure 3

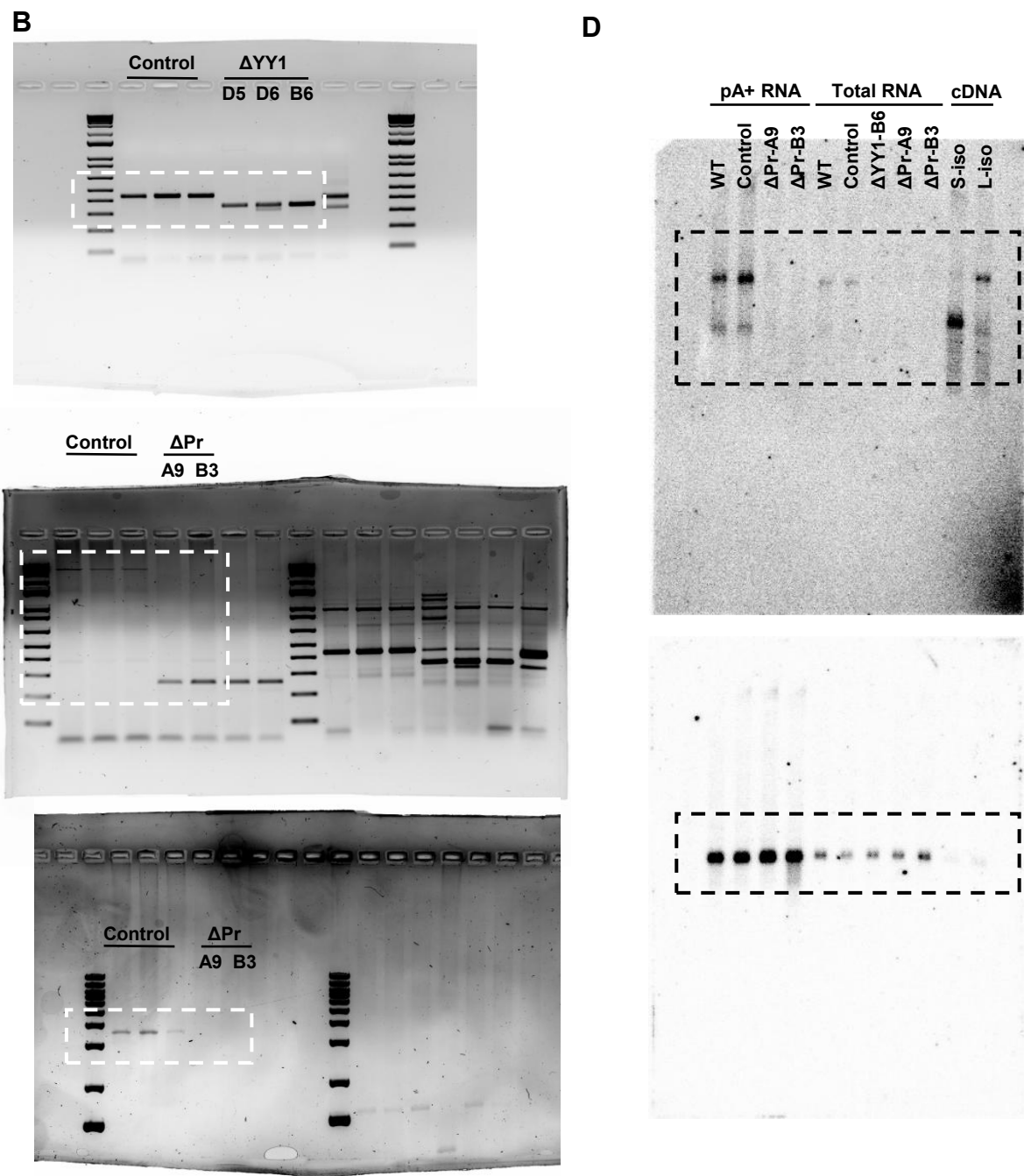

Supplement: Figure 3—source data 1. [file elife-102681-fig3-data1.pdf]

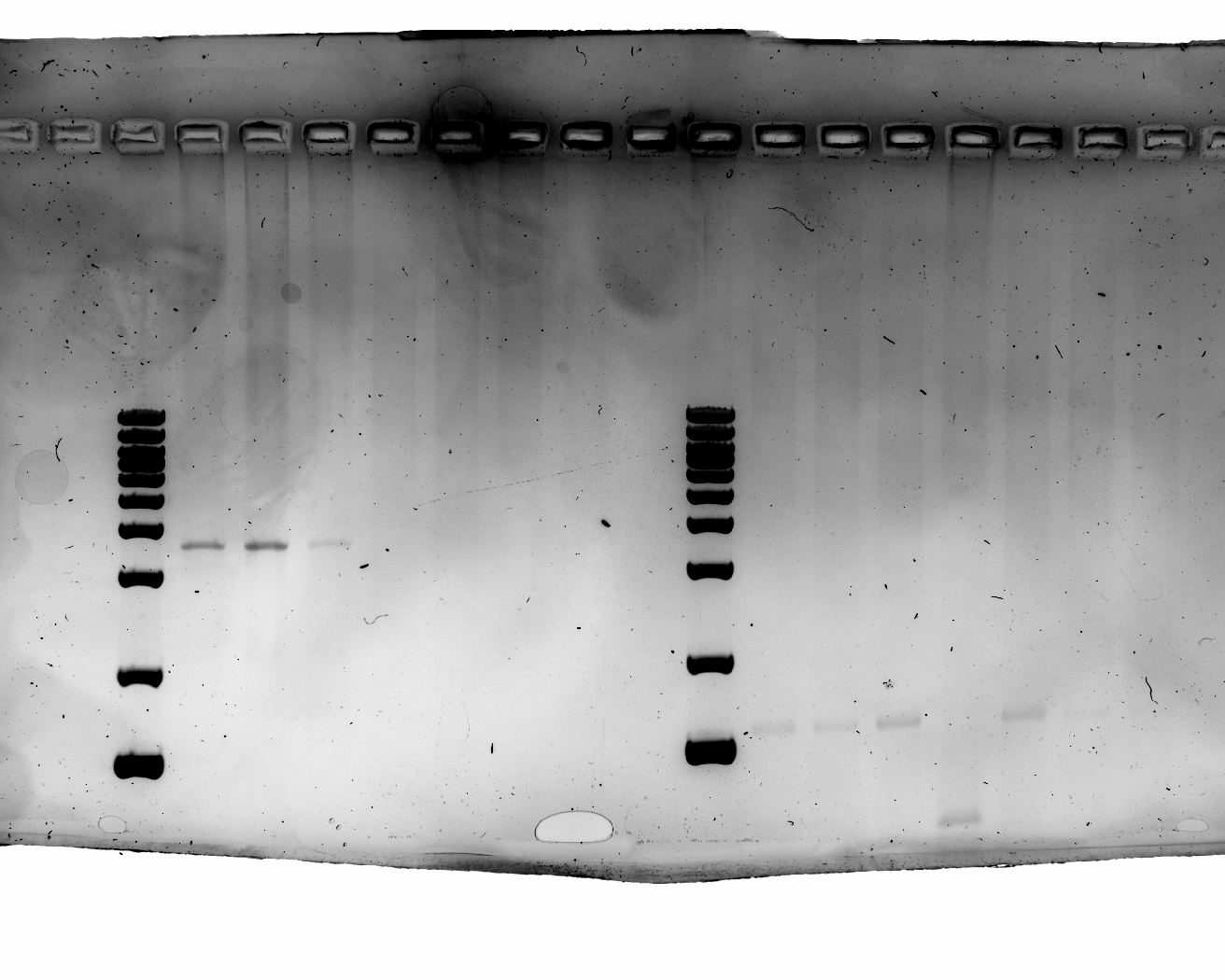

Supplement: Figure 3—source data 2. [file elife-102681-fig3-data2.zip › Figure 3-source data 2/2019-01-22 17hr 17min 51sec.tif]

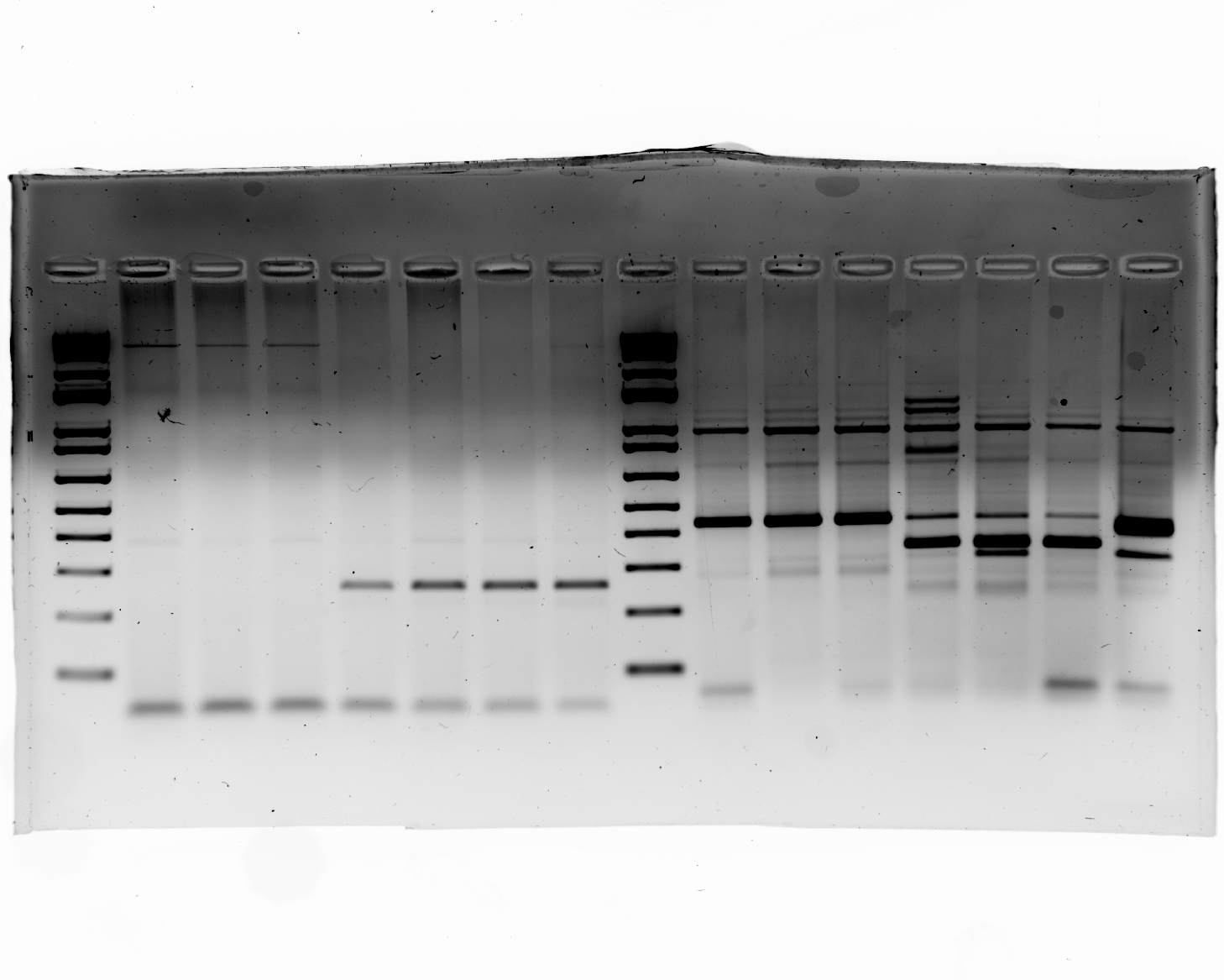

Supplement: Figure 3—source data 2. [file elife-102681-fig3-data2.zip › Figure 3-source data 2/2019-01-23 12hr 31min 38sec.tif]

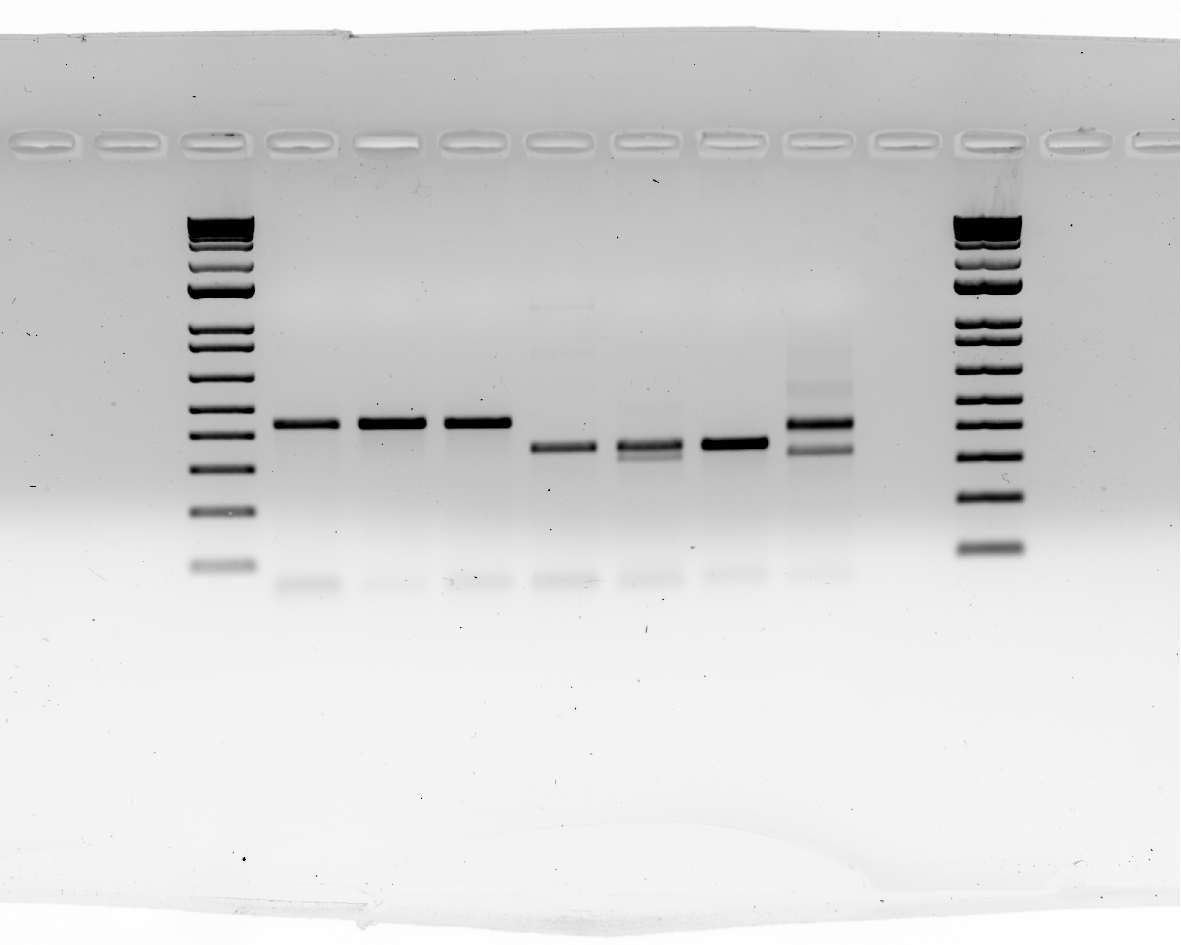

Supplement: Figure 3—source data 2. [file elife-102681-fig3-data2.zip › Figure 3-source data 2/2019-01-24 16hr 56min 56sec.tif]

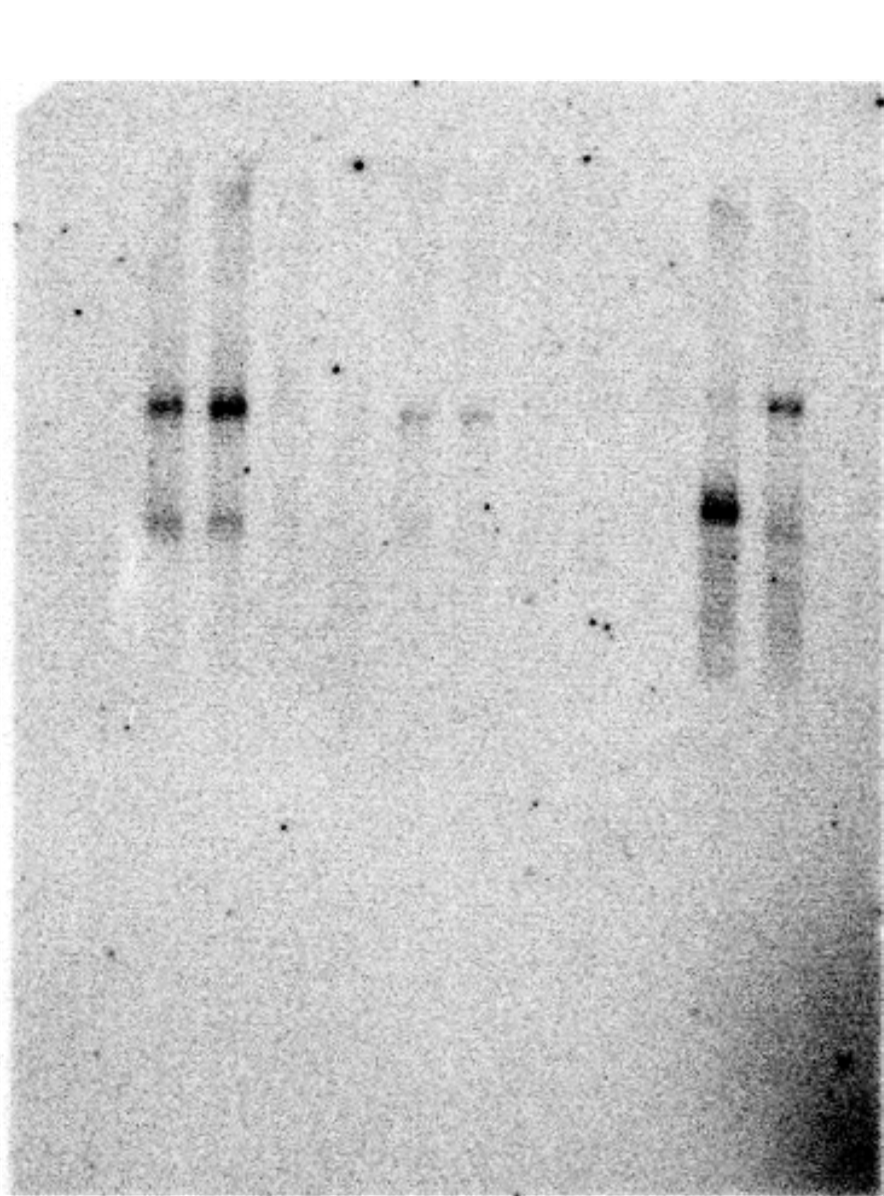

Supplement: Figure 3—source data 2. [file elife-102681-fig3-data2.zip › Figure 3-source data 2/Northern-1.tif]

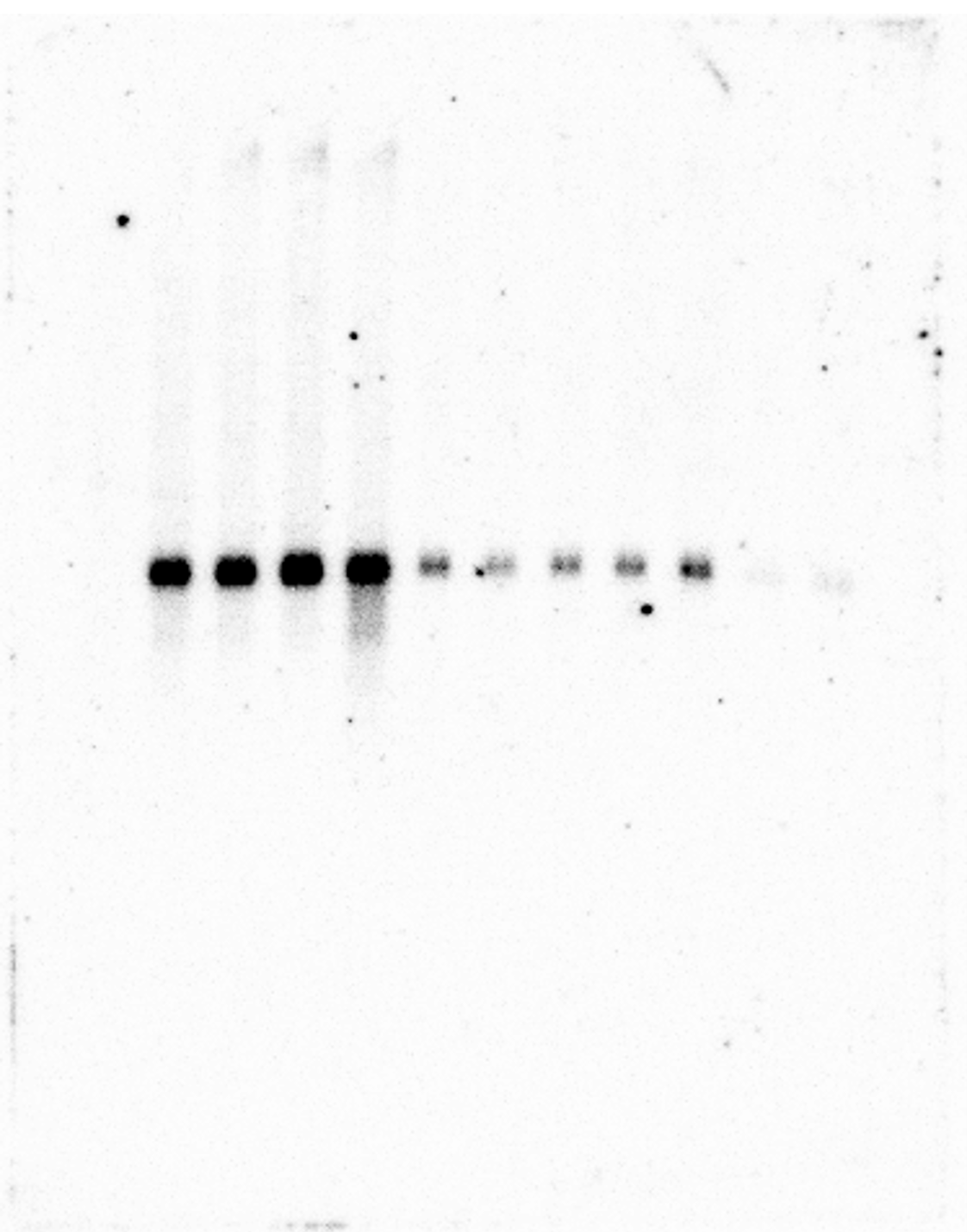

Supplement: Figure 3—source data 2. [file elife-102681-fig3-data2.zip › Figure 3-source data 2/Northern-2.tif]

Figure 3-figure supplement 2

A

Expt I

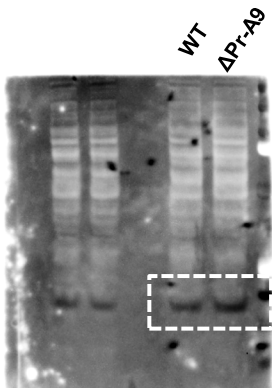

E6

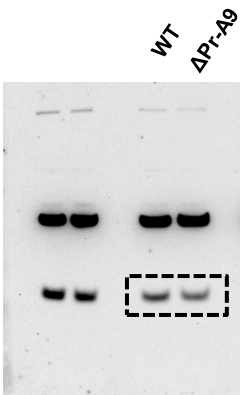

E7

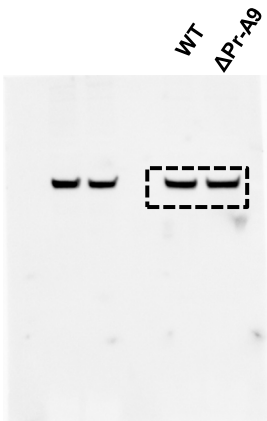

GAPDH

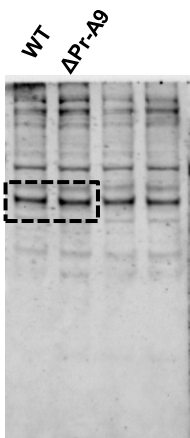

E2F1

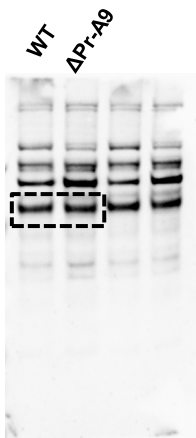

P53

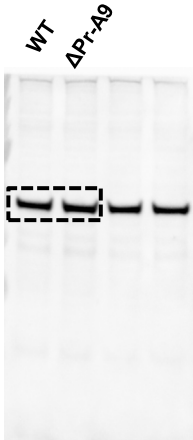

Tubulin

Figure 3-figure supplement 2

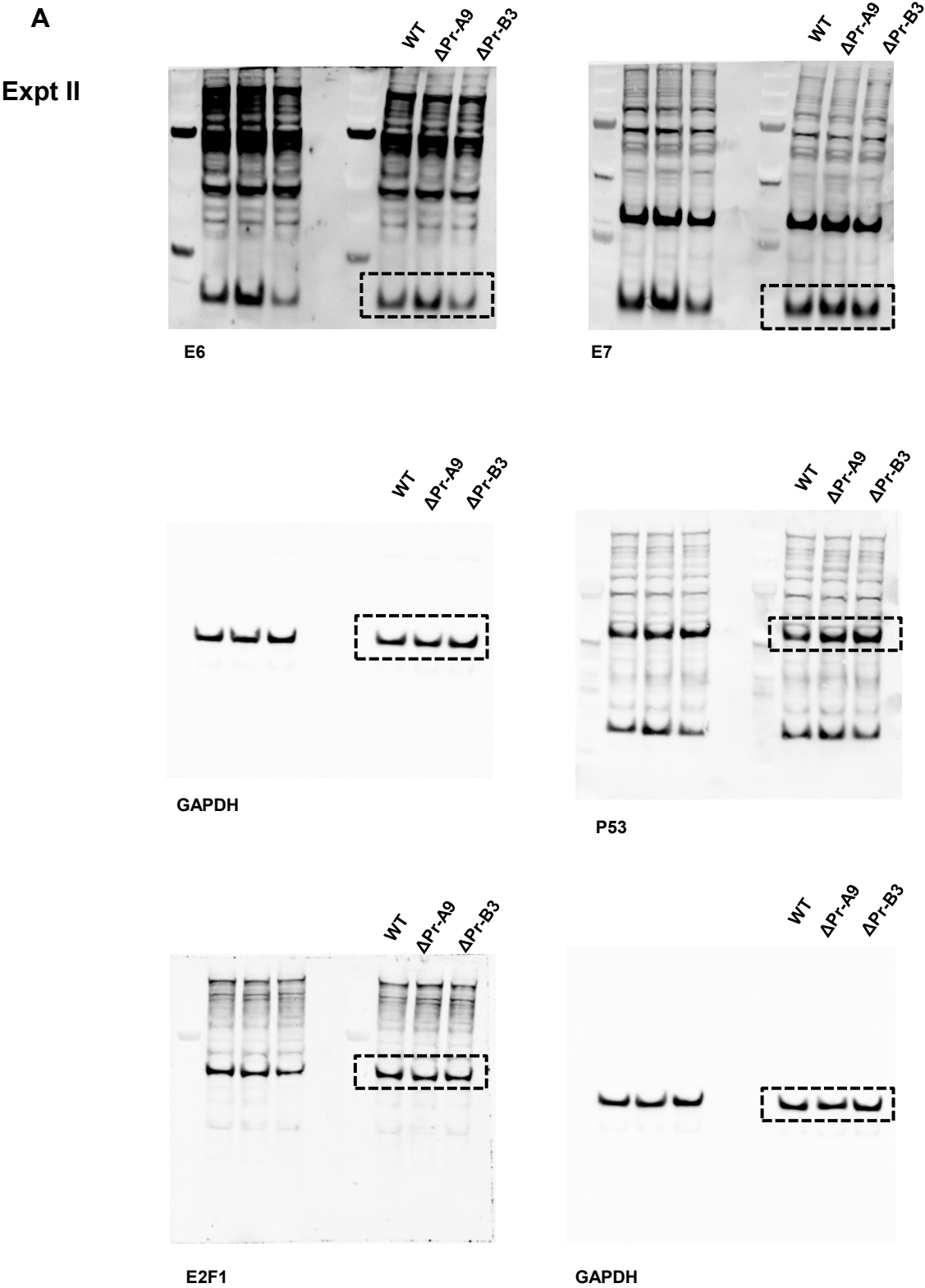

Supplement: Figure 3—figure supplement 2—source data 1. [file elife-102681-fig3-figsupp2-data1.pdf]

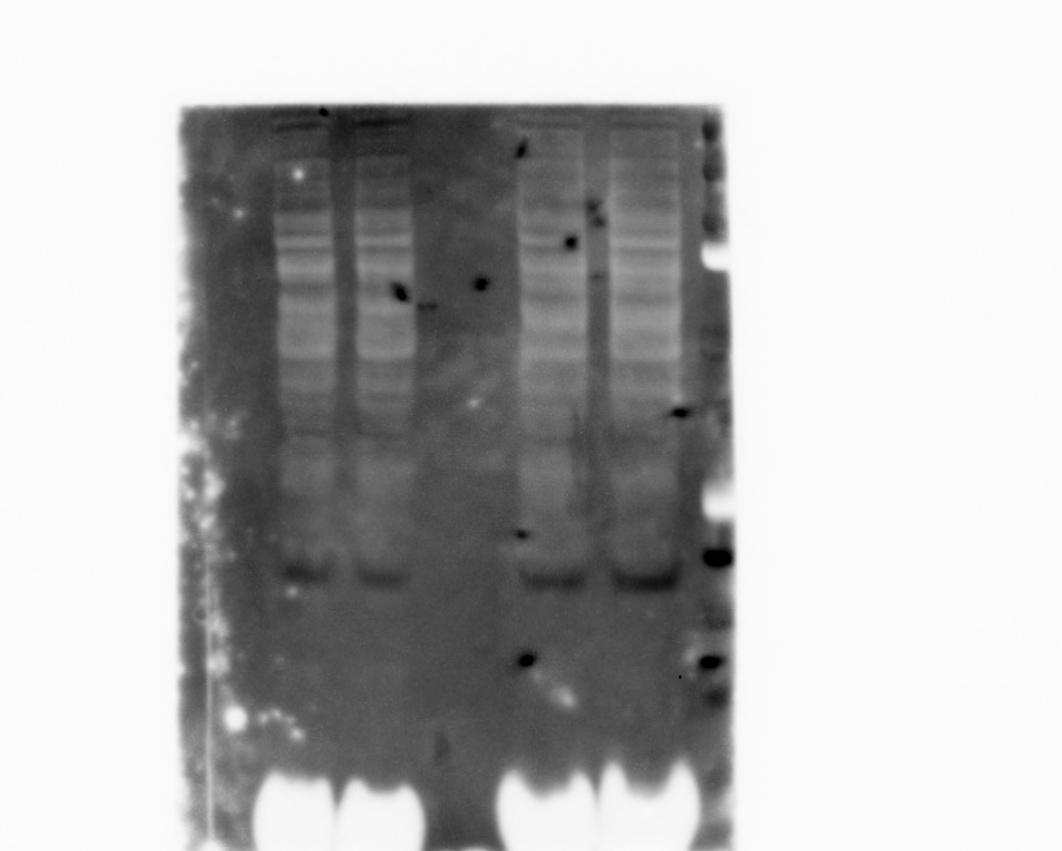

Supplement: Figure 3—figure supplement 2—source data 2. [file elife-102681-fig3-figsupp2-data2.zip › Figure 3-figure supplement 2-source_data_2/zheng lab 2021-09-02 13h07m13s.tif]

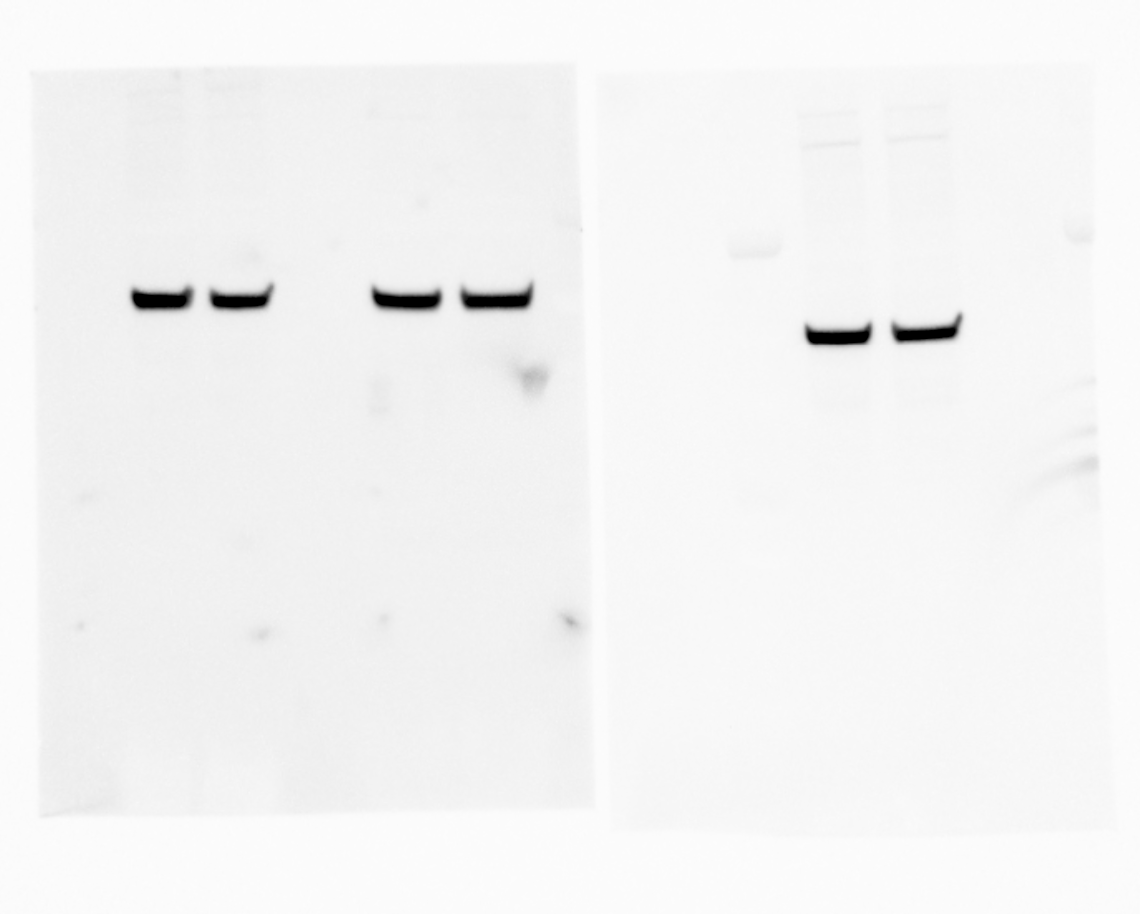

Supplement: Figure 3—figure supplement 2—source data 2. [file elife-102681-fig3-figsupp2-data2.zip › Figure 3-figure supplement 2-source_data_2/zheng lab 2021-09-20 12h22m30s.tif]

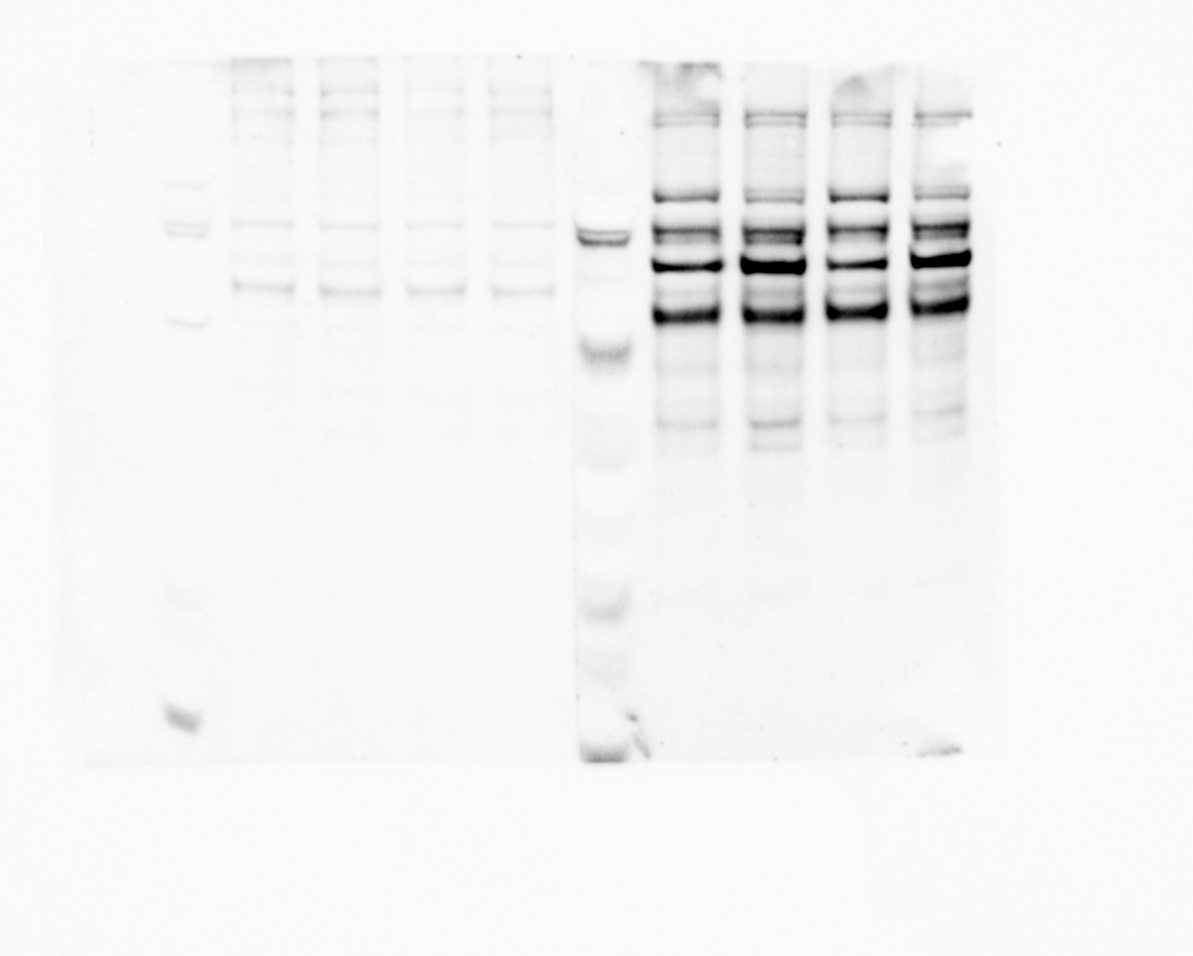

Supplement: Figure 3—figure supplement 2—source data 2. [file elife-102681-fig3-figsupp2-data2.zip › Figure 3-figure supplement 2-source_data_2/zheng lab 2022-03-15 14h12m13s.tif]

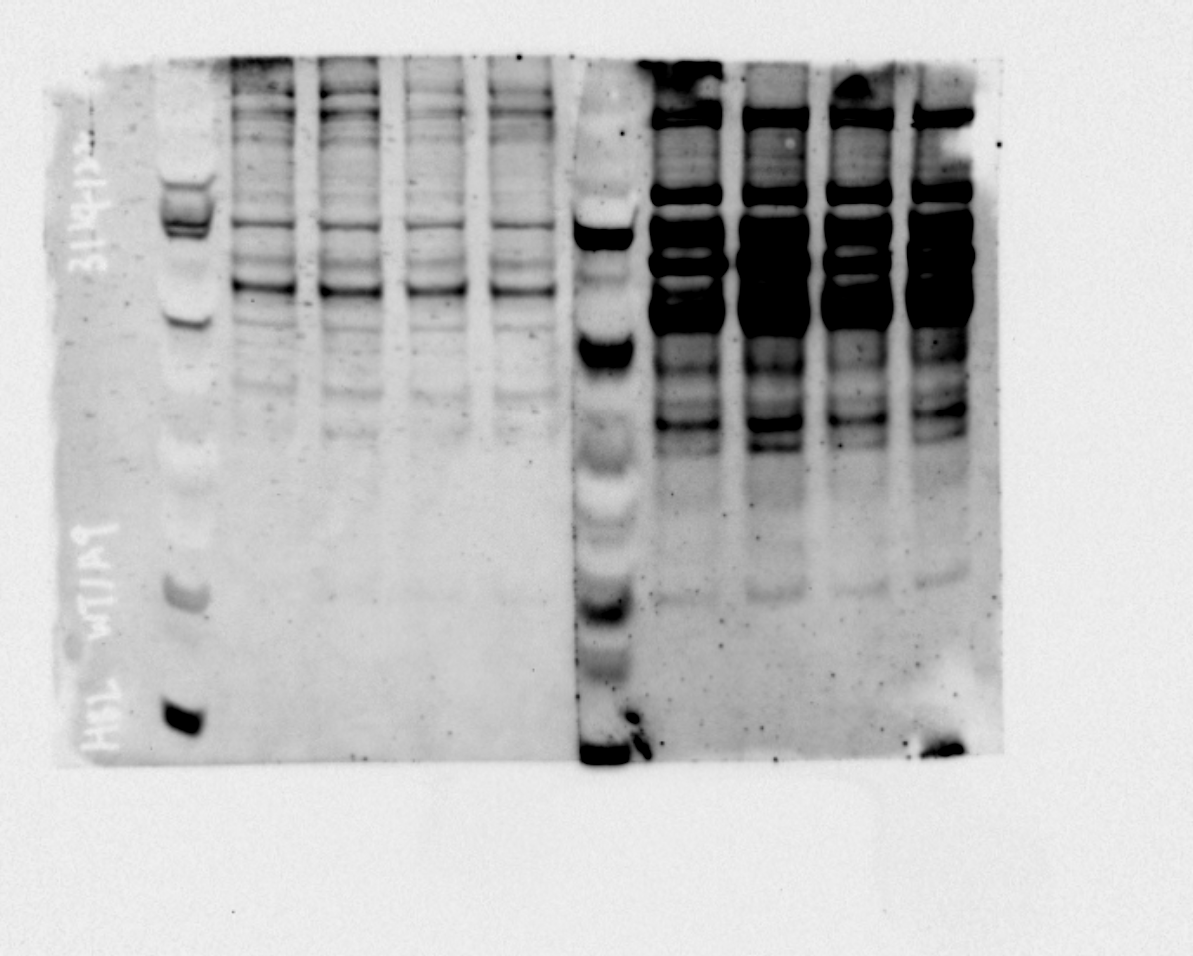

Supplement: Figure 3—figure supplement 2—source data 2. [file elife-102681-fig3-figsupp2-data2.zip › Figure 3-figure supplement 2-source_data_2/zheng lab 2022-03-16 14h13m27s.tif]

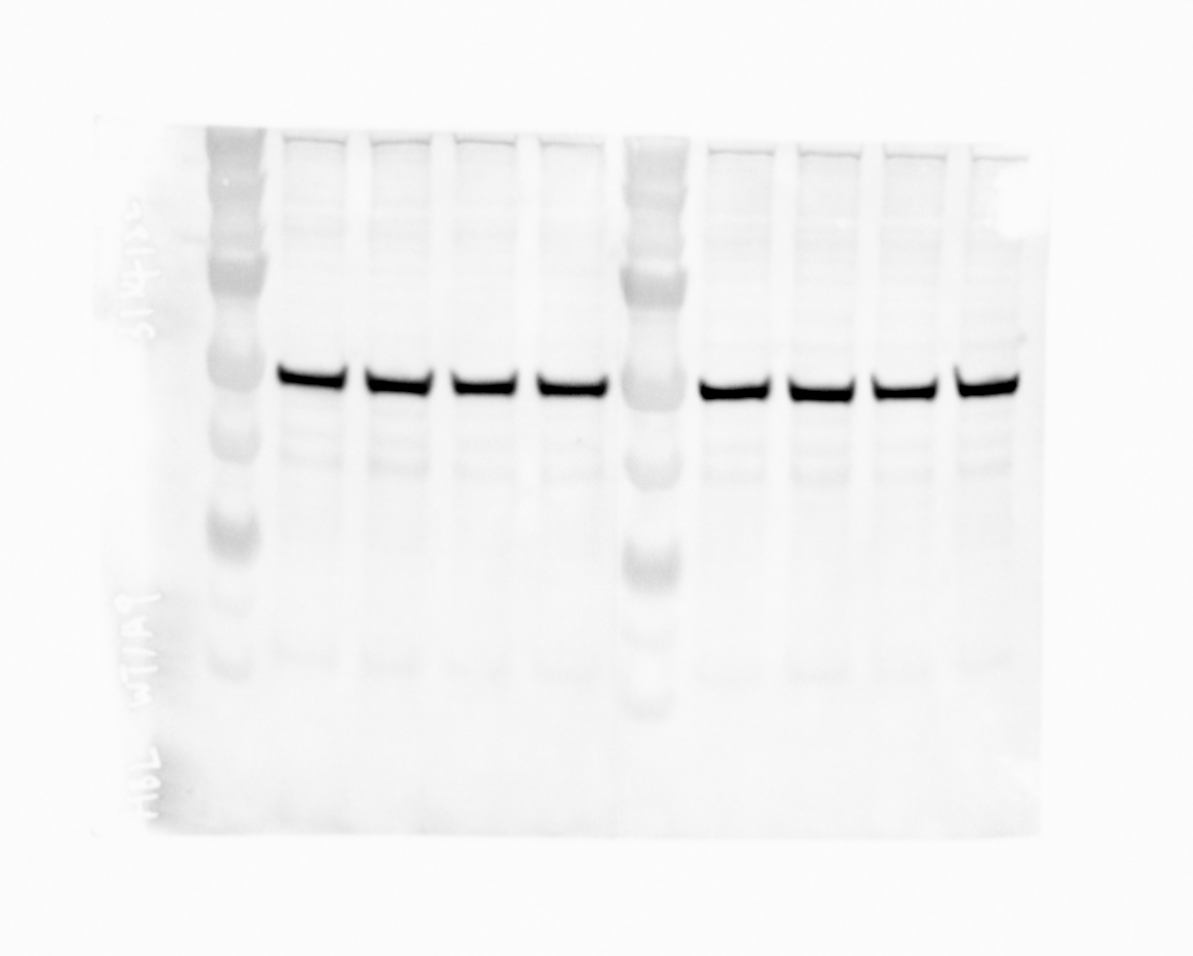

Supplement: Figure 3—figure supplement 2—source data 2. [file elife-102681-fig3-figsupp2-data2.zip › Figure 3-figure supplement 2-source_data_2/zheng lab 2022-03-17 10h52m38s.tif]

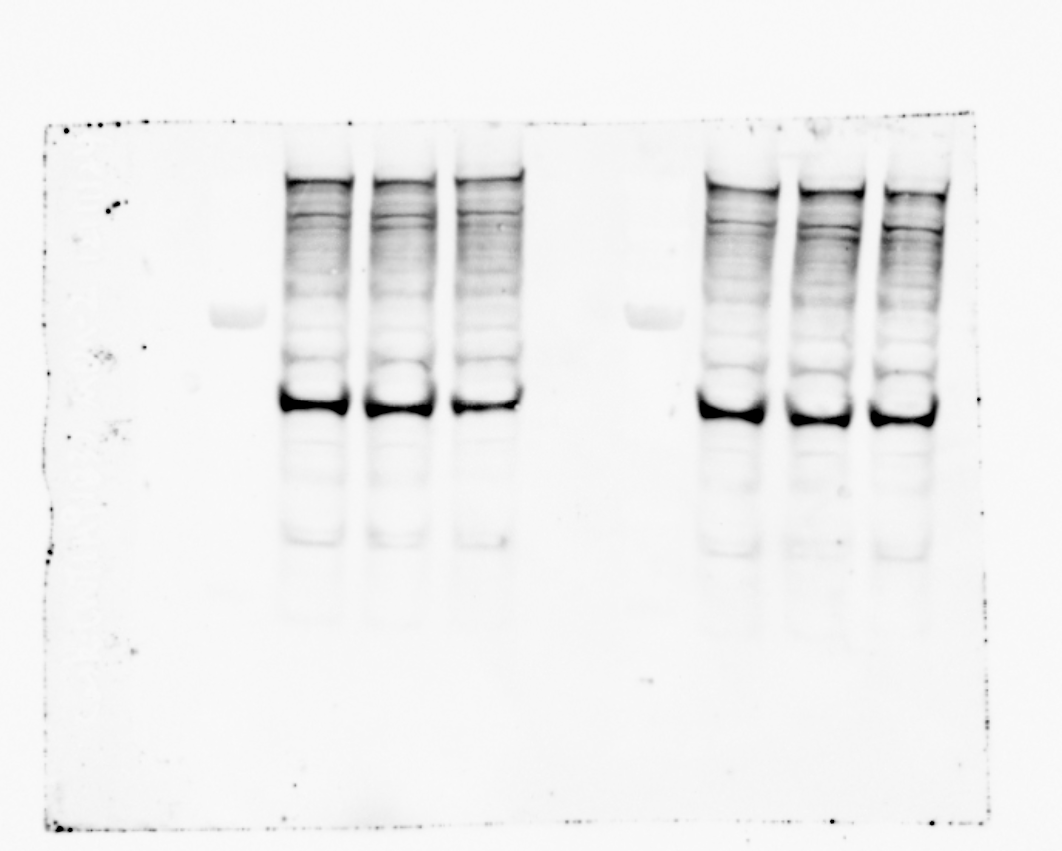

Supplement: Figure 3—figure supplement 2—source data 2. [file elife-102681-fig3-figsupp2-data2.zip › Figure 3-figure supplement 2-source_data_2/zheng lab 2024-12-13 10h10m09s.tif]

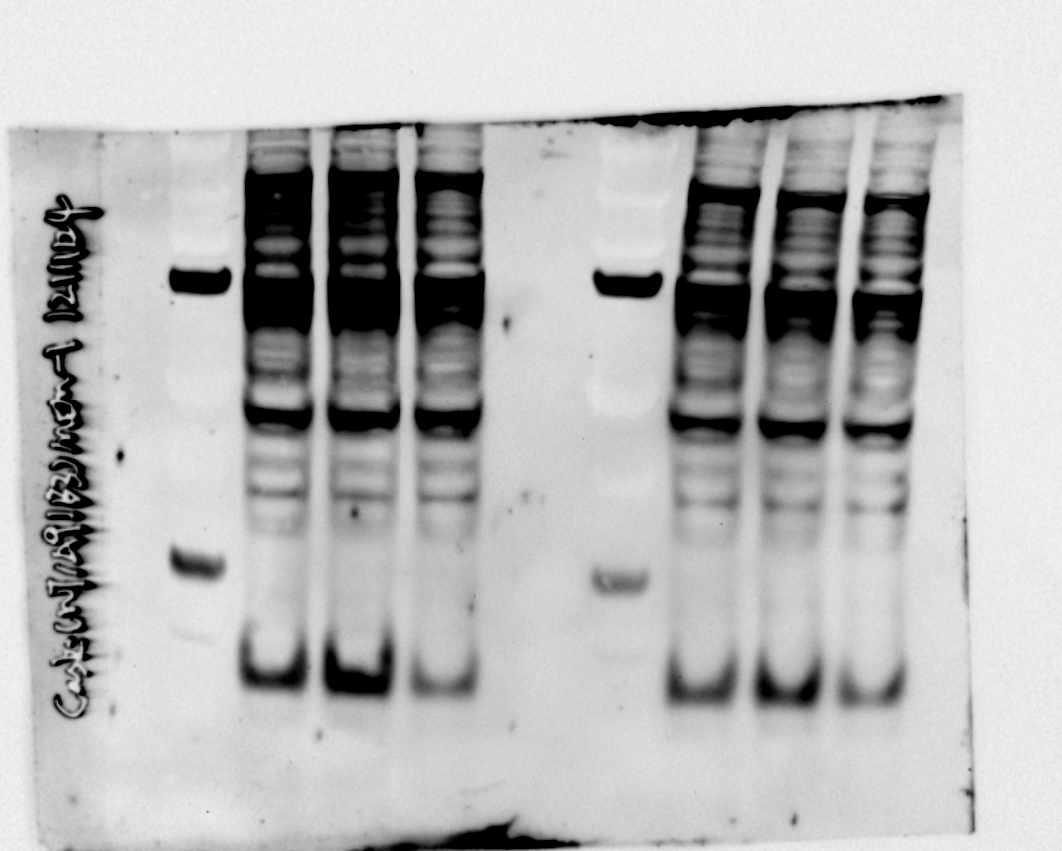

Supplement: Figure 3—figure supplement 2—source data 2. [file elife-102681-fig3-figsupp2-data2.zip › Figure 3-figure supplement 2-source_data_2/zheng lab 2024-12-13 10h13m51s.tif]

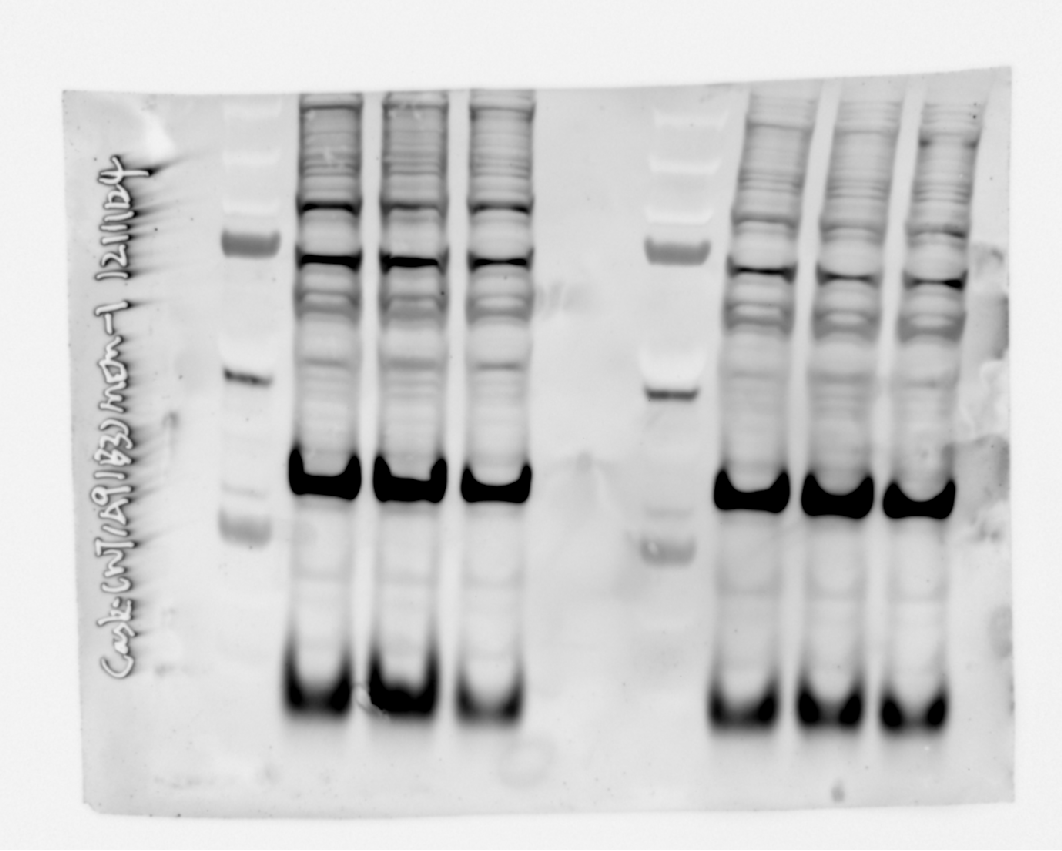

Supplement: Figure 3—figure supplement 2—source data 2. [file elife-102681-fig3-figsupp2-data2.zip › Figure 3-figure supplement 2-source_data_2/zheng lab 2024-12-16 09h07m21s.tif]

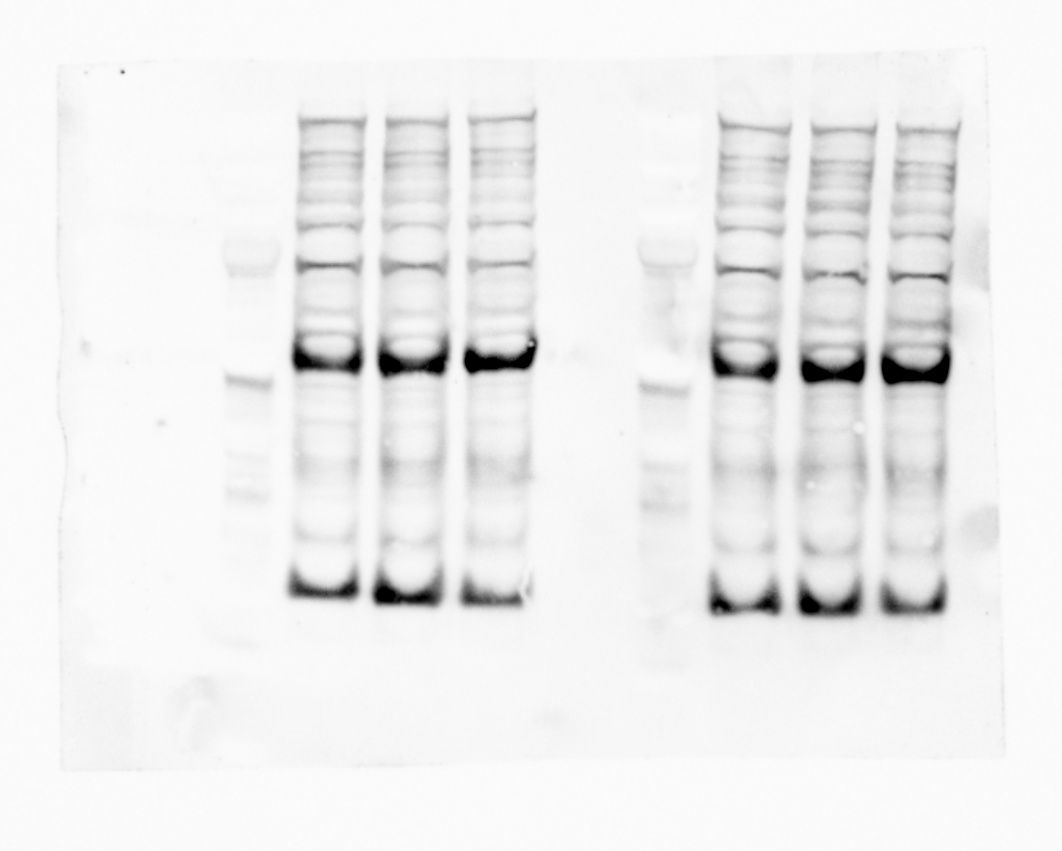

Supplement: Figure 3—figure supplement 2—source data 2. [file elife-102681-fig3-figsupp2-data2.zip › Figure 3-figure supplement 2-source_data_2/zheng lab 2024-12-16 09h14m07s.tif]

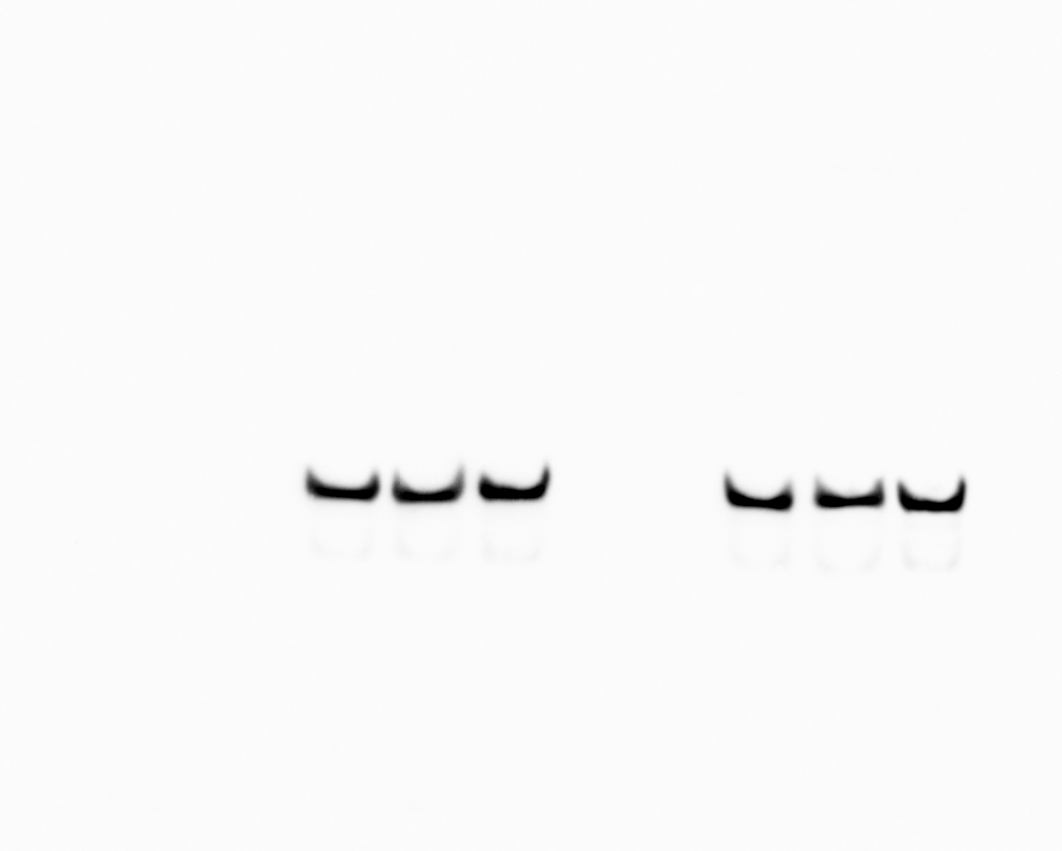

Supplement: Figure 3—figure supplement 2—source data 2. [file elife-102681-fig3-figsupp2-data2.zip › Figure 3-figure supplement 2-source_data_2/zheng lab 2024-12-16 14h05m53s.tif]

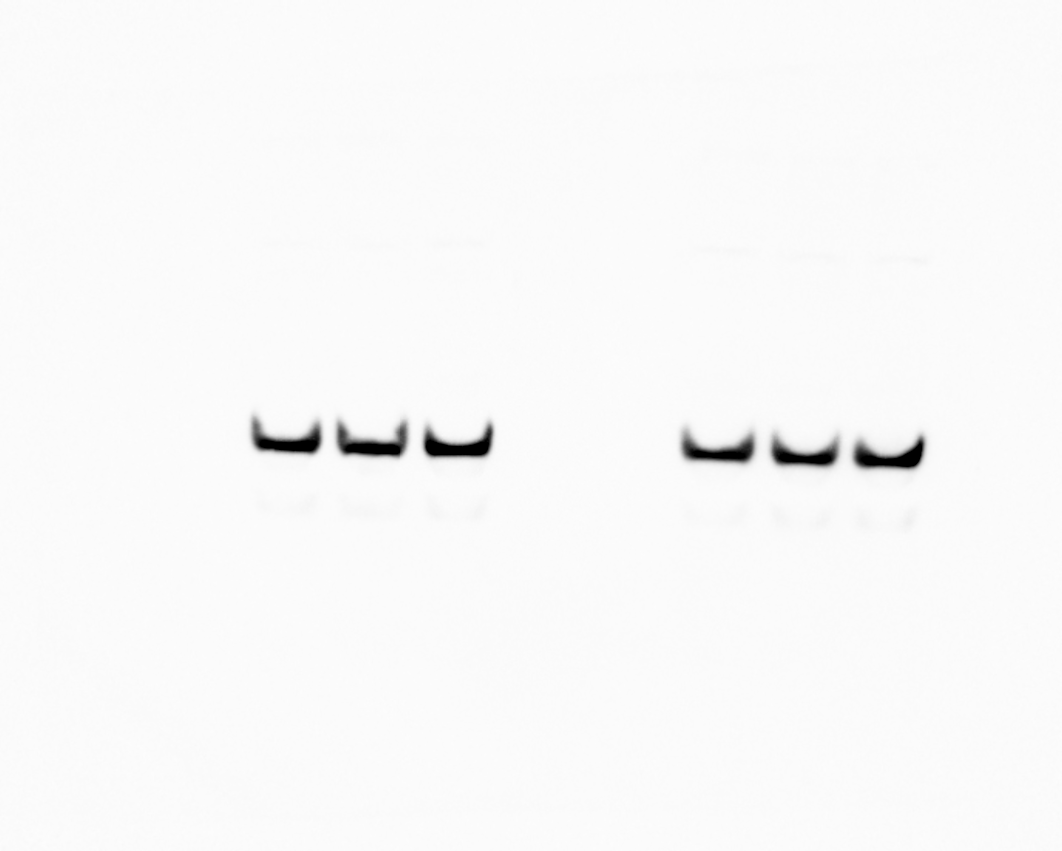

Supplement: Figure 3—figure supplement 2—source data 2. [file elife-102681-fig3-figsupp2-data2.zip › Figure 3-figure supplement 2-source_data_2/zheng lab 2024-12-16 14h10m04s.tif]

**A**

## Cell lysis

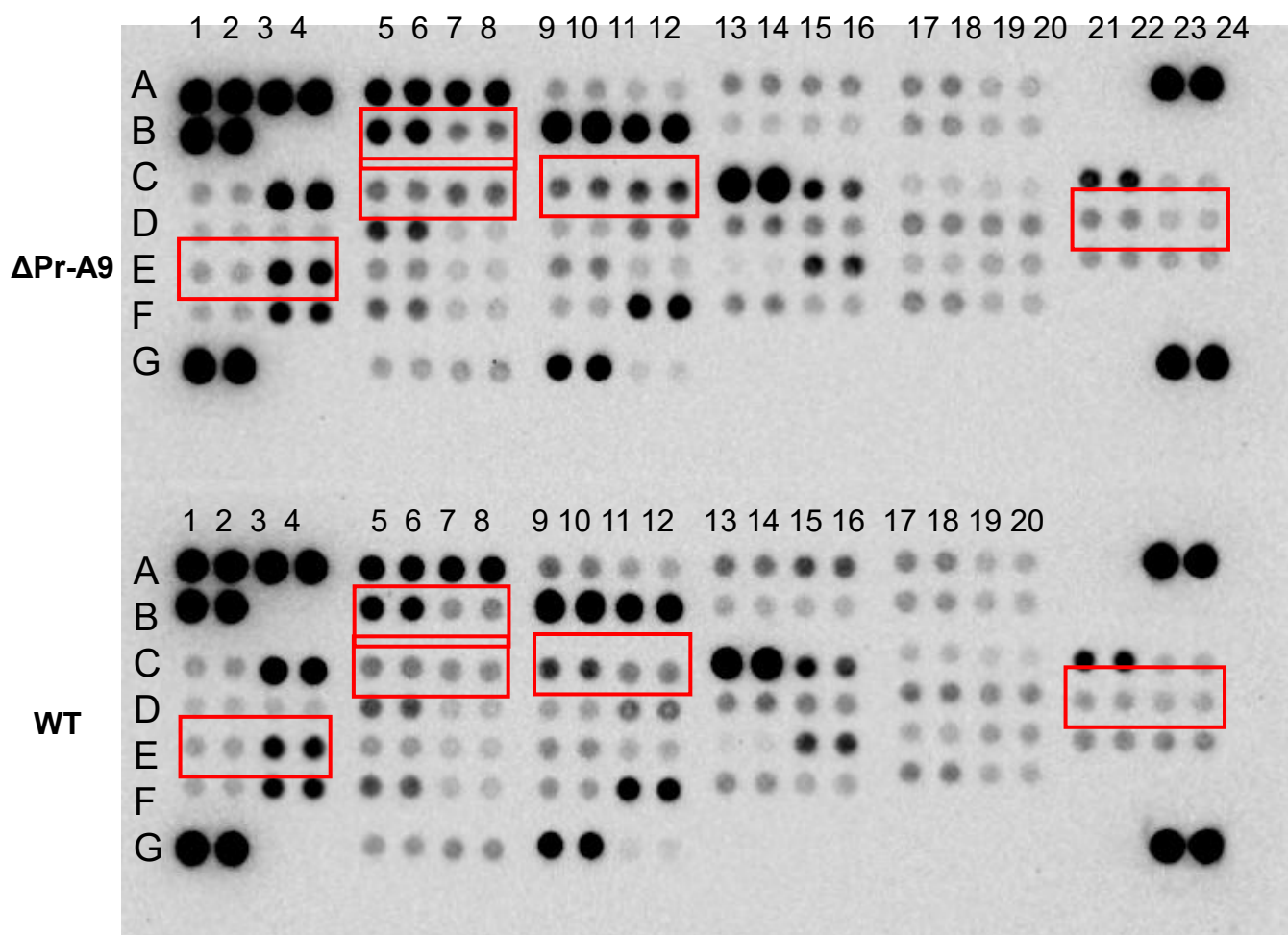

**A**

## Cell culture supernates

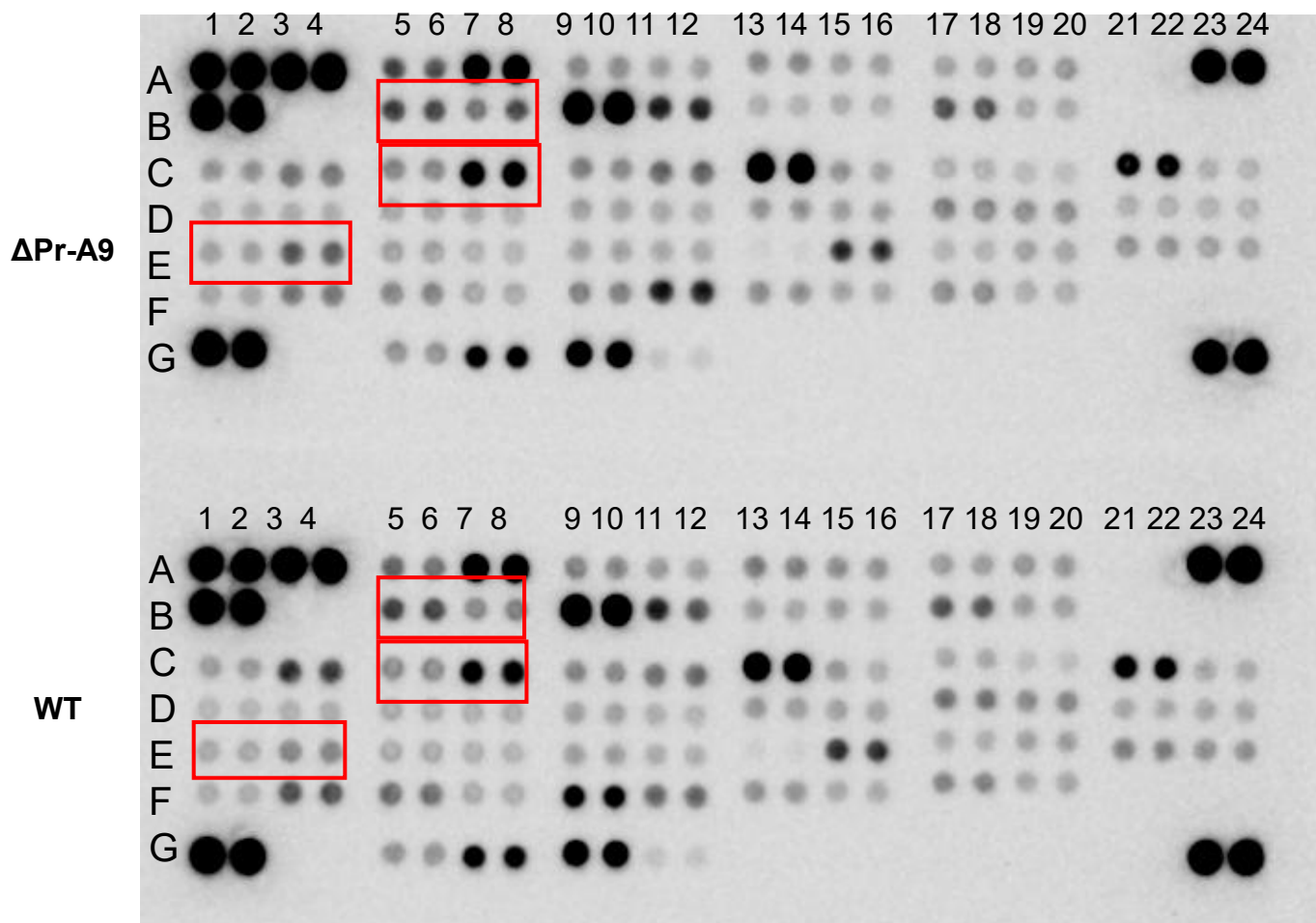

**A**

## Cell lysis

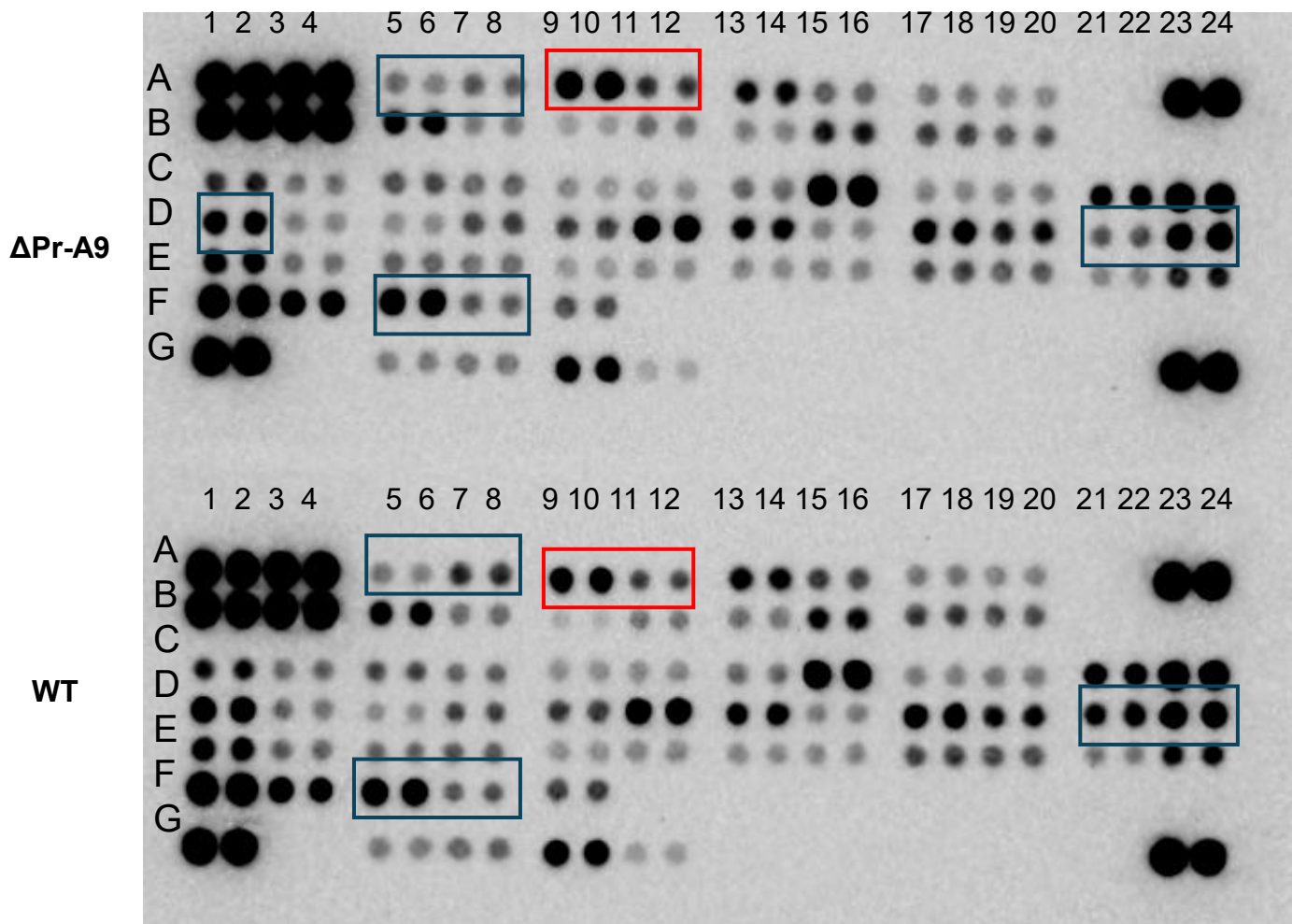

Figure 4

A

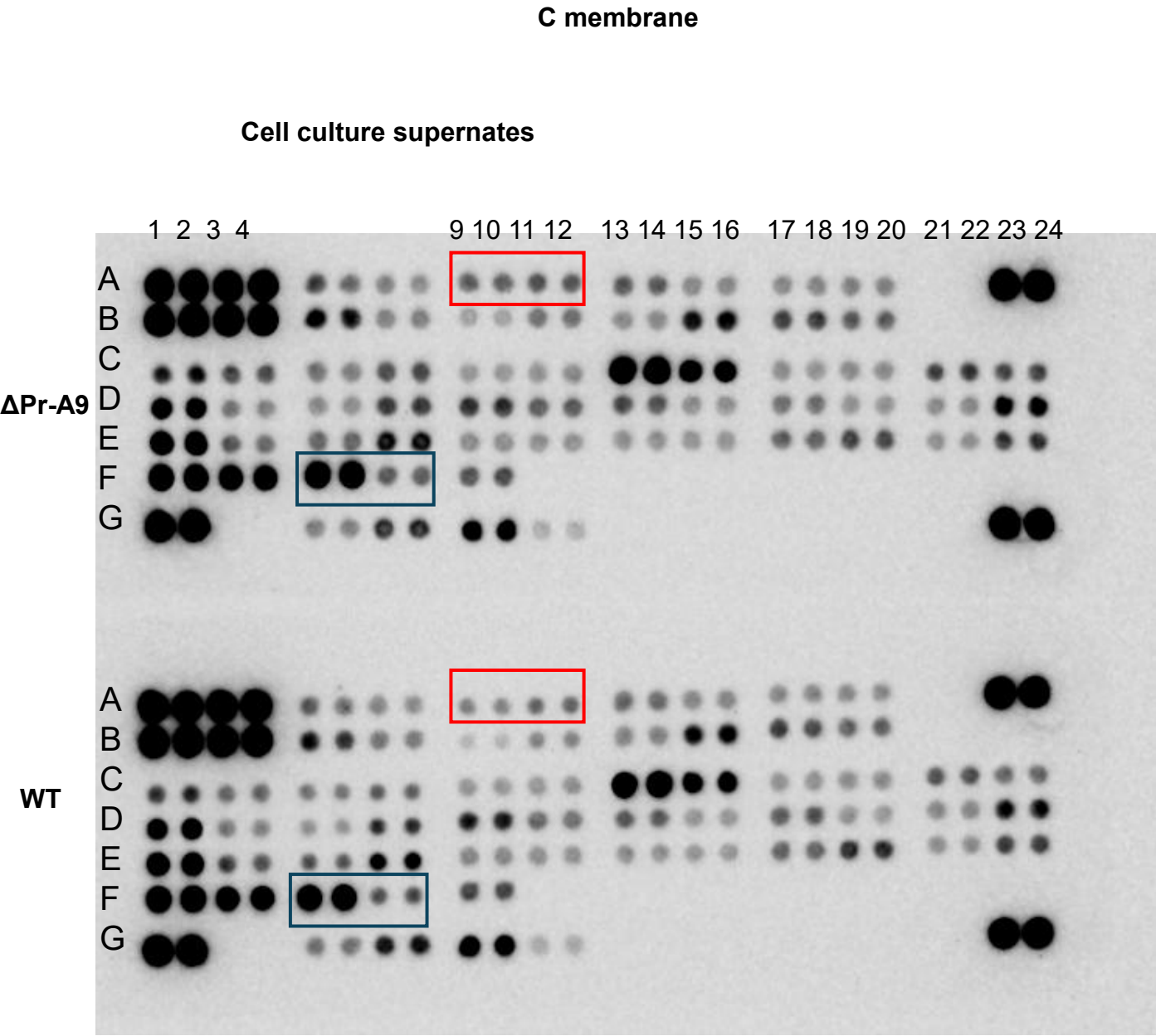

Figure 4

C

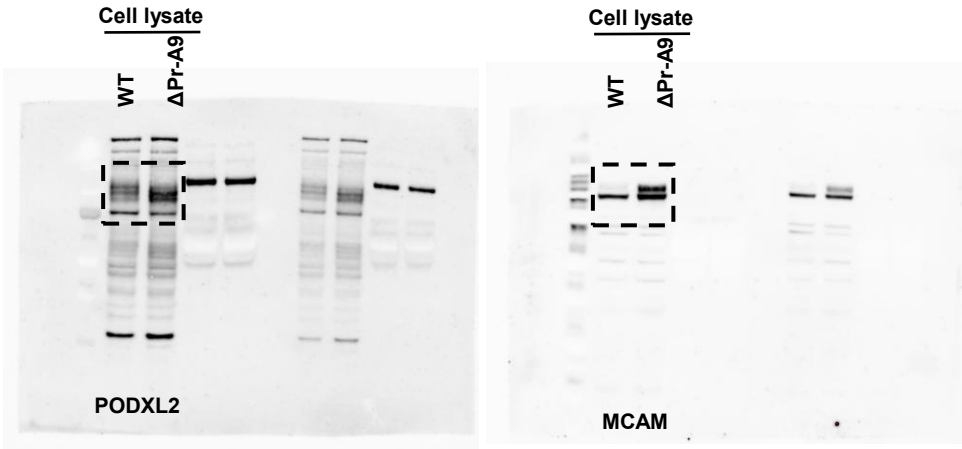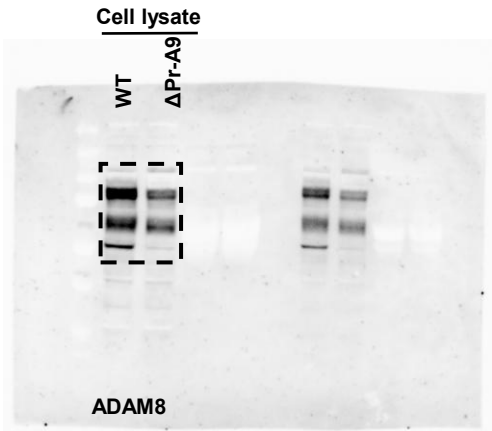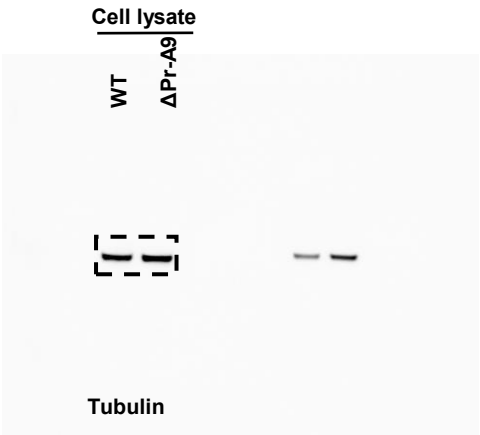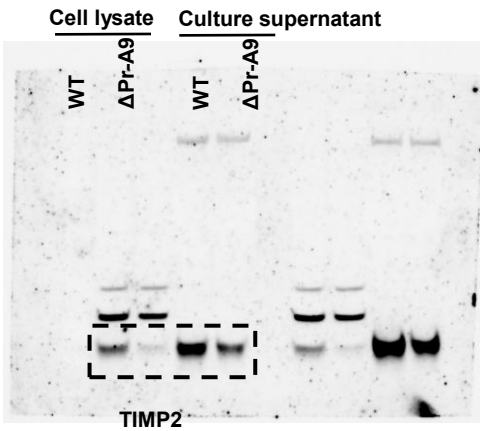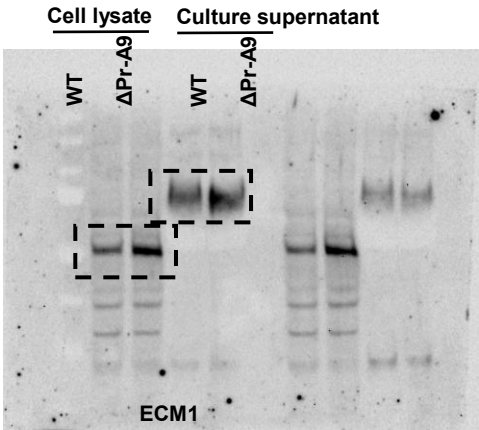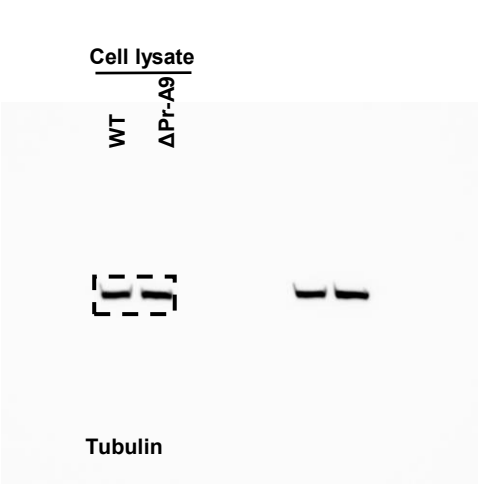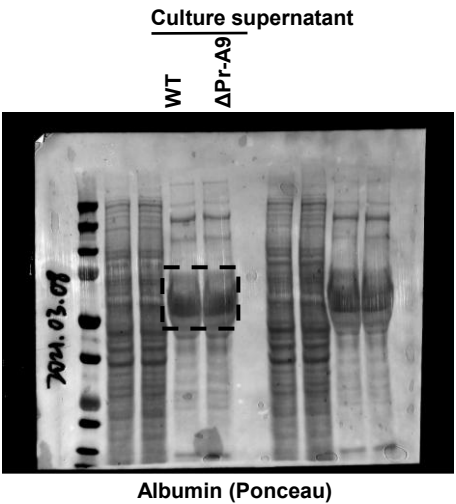

Supplement: Figure 4—source data 1. [file elife-102681-fig4-data1.pdf]

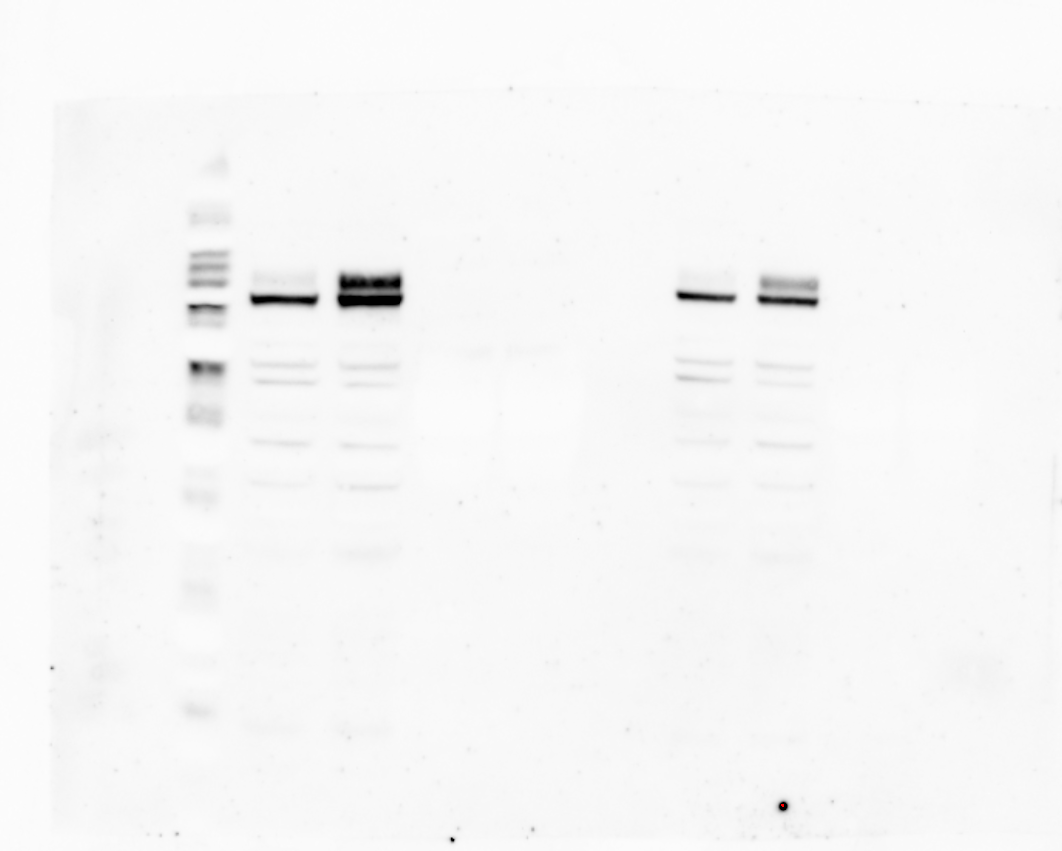

Supplement: Figure 4—source data 2. [file elife-102681-fig4-data2.zip › Figure 4-source_data_2/zheng lab 2021-01-12 15h20m11s.tif]

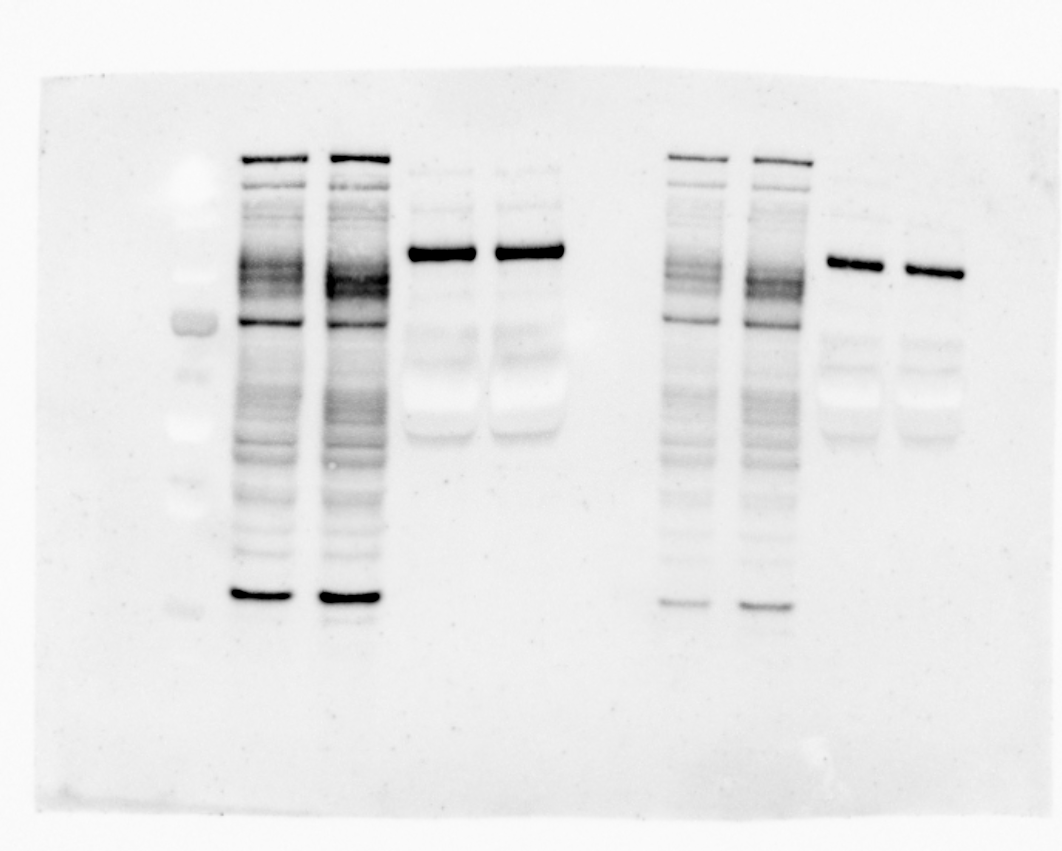

Supplement: Figure 4—source data 2. [file elife-102681-fig4-data2.zip › Figure 4-source_data_2/zheng lab 2021-01-25 12h44m02s.tif]

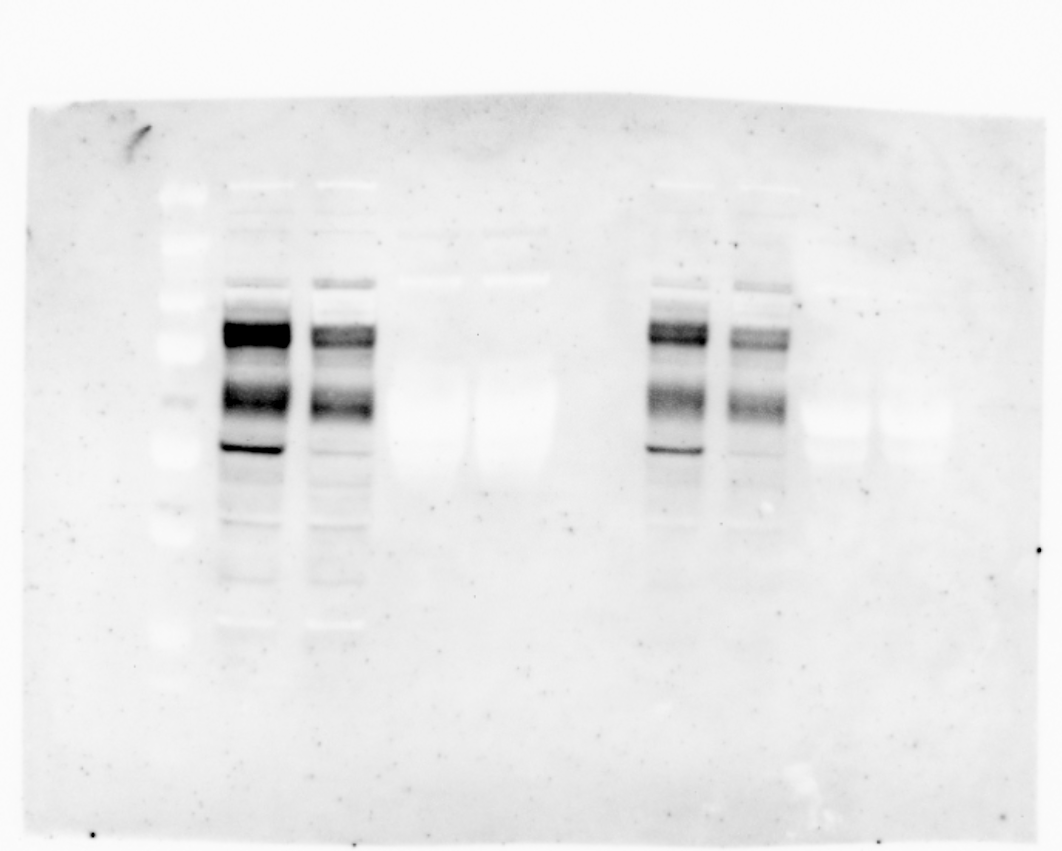

Supplement: Figure 4—source data 2. [file elife-102681-fig4-data2.zip › Figure 4-source_data_2/zheng lab 2021-01-26 14h01m43s.tif]

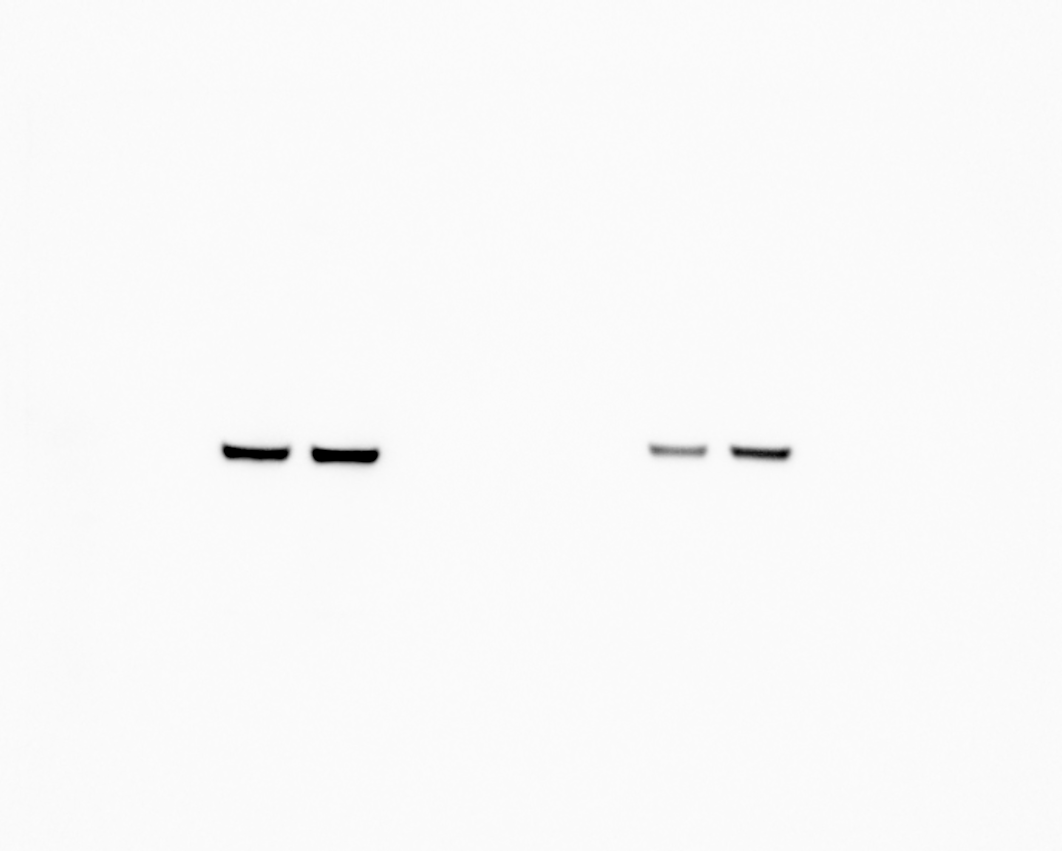

Supplement: Figure 4—source data 2. [file elife-102681-fig4-data2.zip › Figure 4-source_data_2/zheng lab 2021-01-28 12h32m23s.tif]

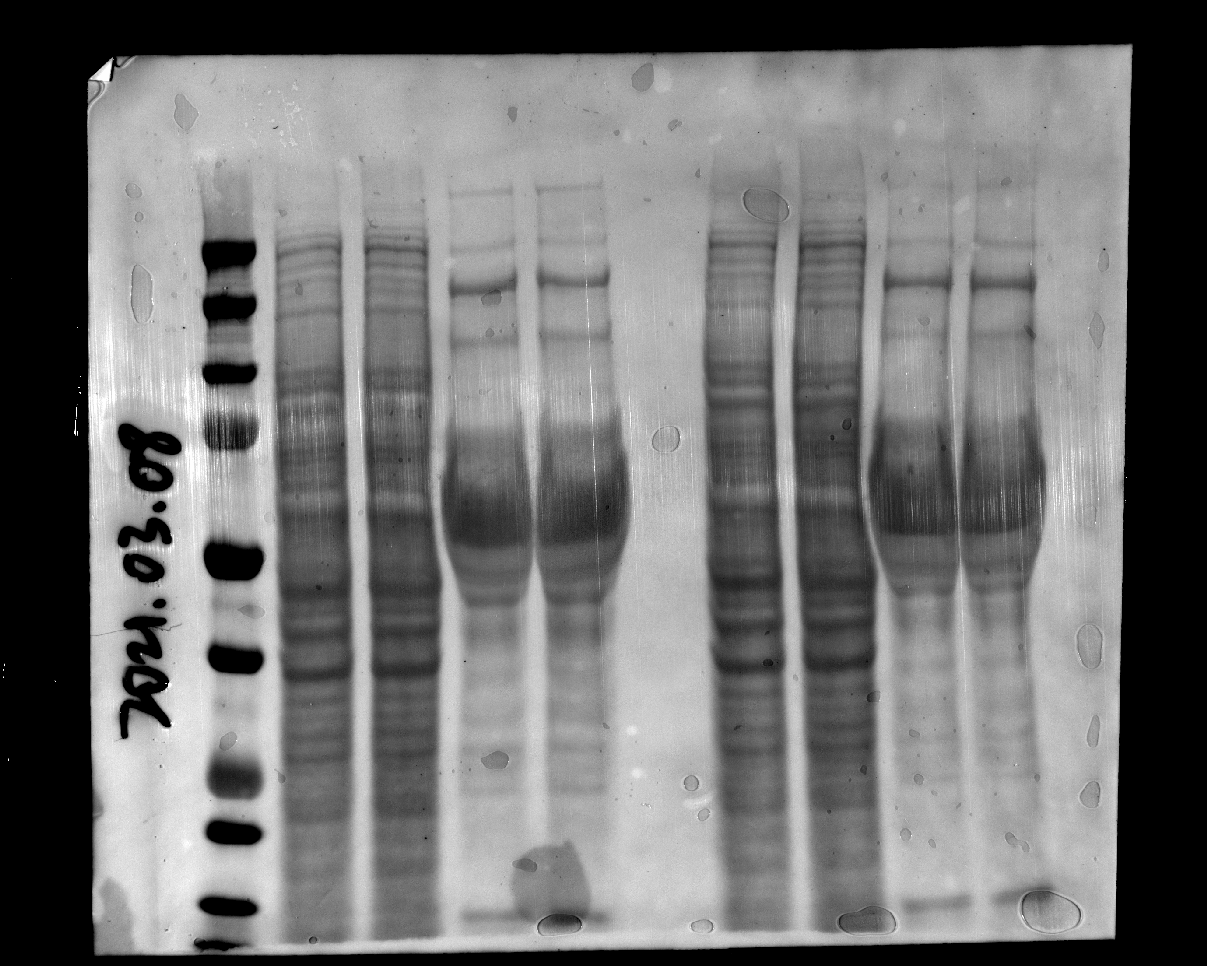

Supplement: Figure 4—source data 2. [file elife-102681-fig4-data2.zip › Figure 4-source_data_2/zheng lab 2021-03-08 15h27m30s.tif]

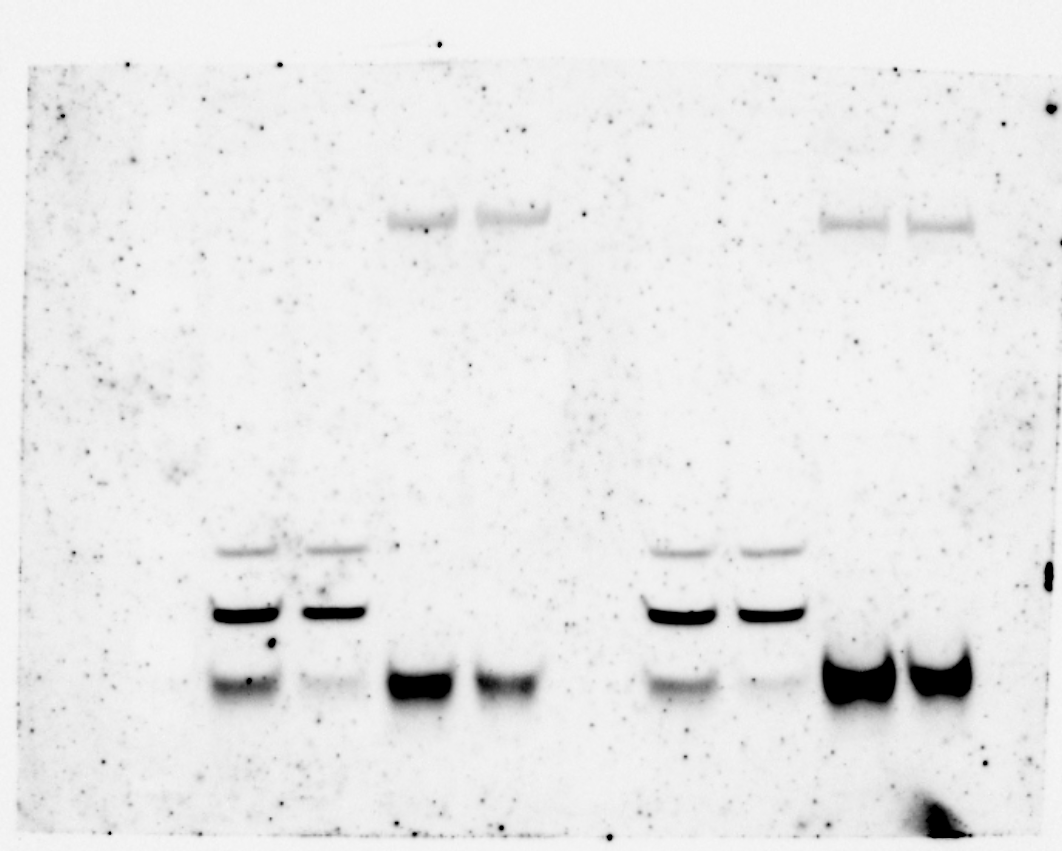

Supplement: Figure 4—source data 2. [file elife-102681-fig4-data2.zip › Figure 4-source_data_2/zheng lab 2021-03-09 16h22m58s.tif]

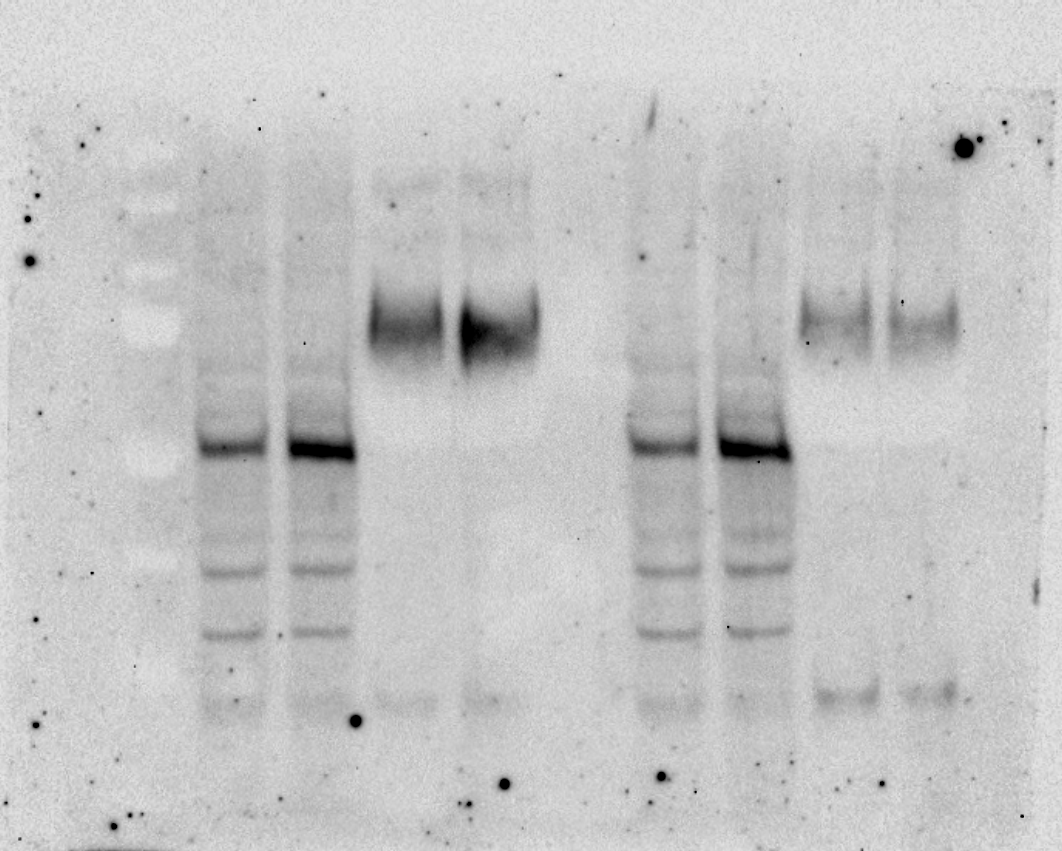

Supplement: Figure 4—source data 2. [file elife-102681-fig4-data2.zip › Figure 4-source_data_2/zheng lab 2021-03-11 12h34m01s.tif]

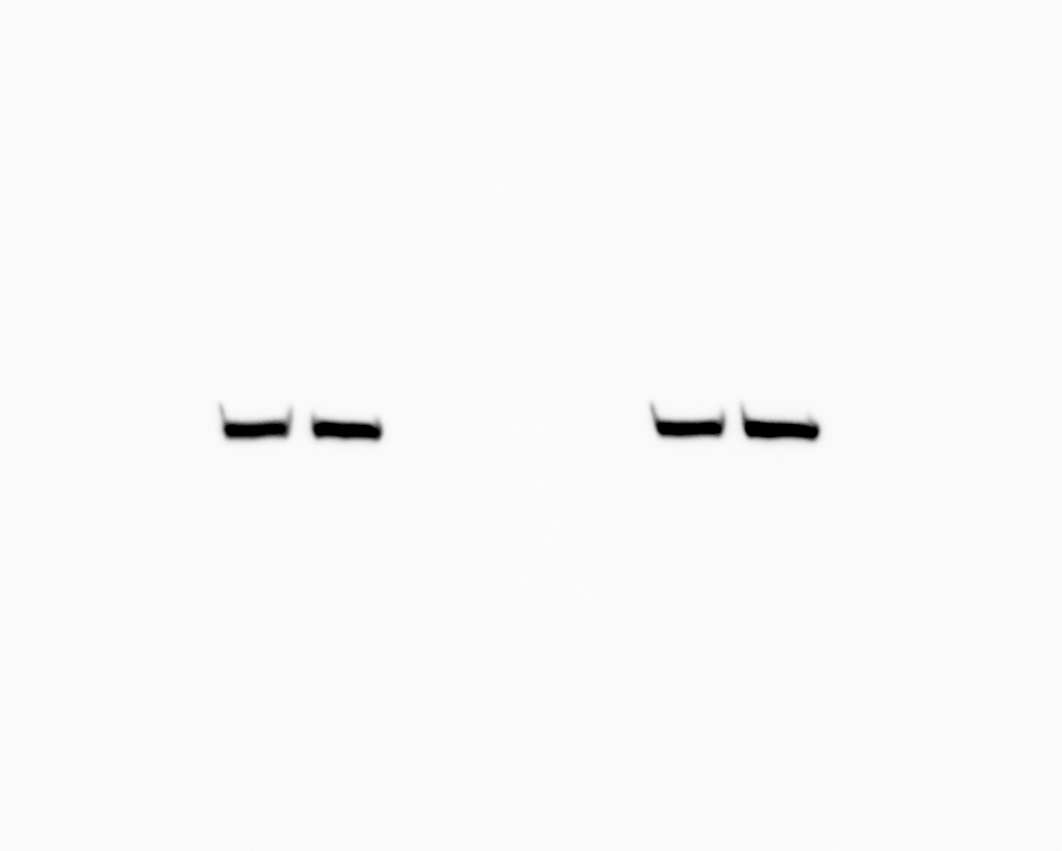

Supplement: Figure 4—source data 2. [file elife-102681-fig4-data2.zip › Figure 4-source_data_2/zheng lab 2021-03-12 12h50m53s.tif]

Figure 5

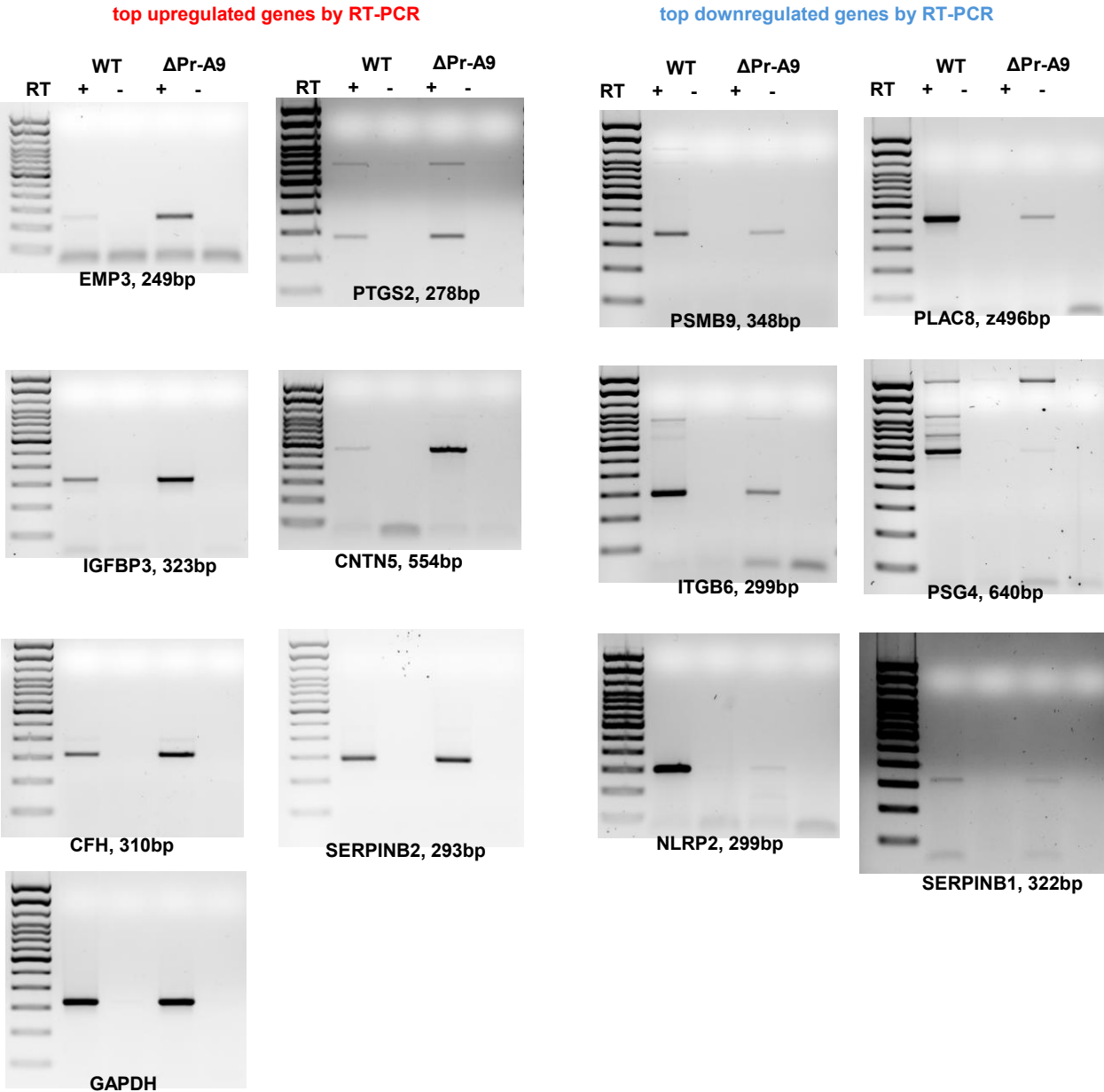

Supplement: Figure 5—source data 1. [file elife-102681-fig5-data1.pdf]

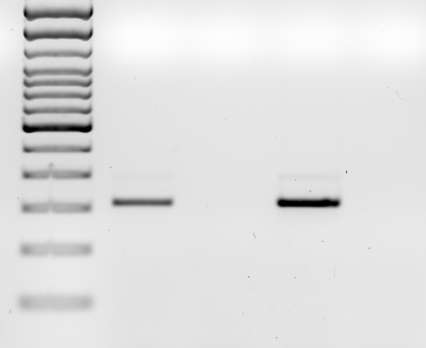

Supplement: Figure 5—source data 2. [file elife-102681-fig5-data2.zip › Figure 5-source_data_2/CFH.tif]

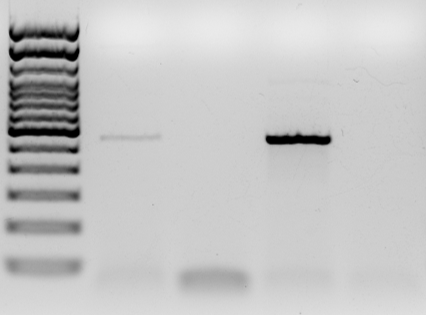

Supplement: Figure 5—source data 2. [file elife-102681-fig5-data2.zip › Figure 5-source_data_2/CNTN5.tif]

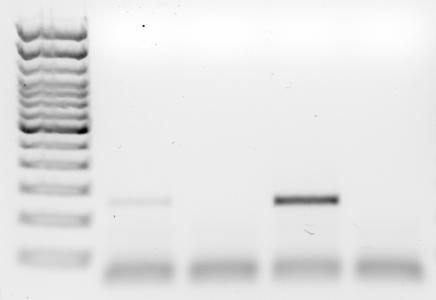

Supplement: Figure 5—source data 2. [file elife-102681-fig5-data2.zip › Figure 5-source_data_2/EMP3.tif]

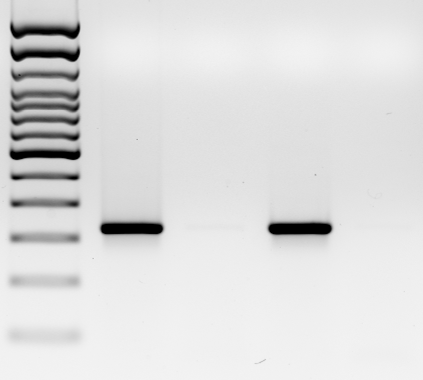

Supplement: Figure 5—source data 2. [file elife-102681-fig5-data2.zip › Figure 5-source_data_2/GAPDH.tif]

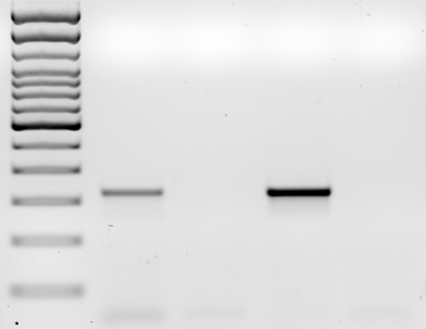

Supplement: Figure 5—source data 2. [file elife-102681-fig5-data2.zip › Figure 5-source_data_2/IGFBP3.tif]

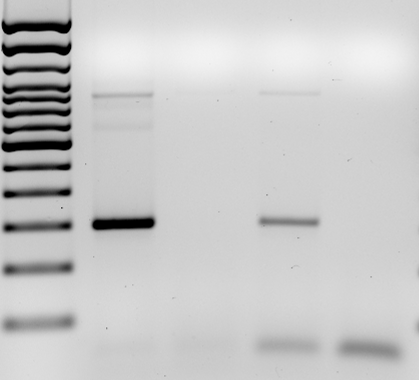

Supplement: Figure 5—source data 2. [file elife-102681-fig5-data2.zip › Figure 5-source_data_2/ITGB6.tif]

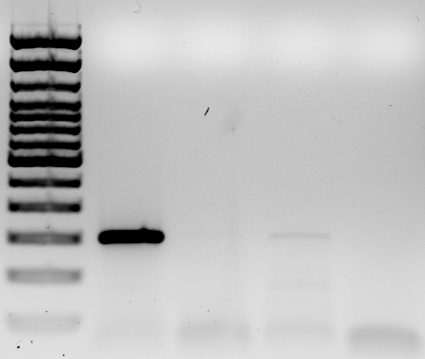

Supplement: Figure 5—source data 2. [file elife-102681-fig5-data2.zip › Figure 5-source_data_2/NLRP2.tif]

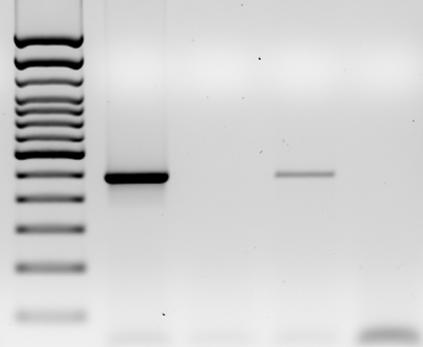

Supplement: Figure 5—source data 2. [file elife-102681-fig5-data2.zip › Figure 5-source_data_2/PLAC8.tif]

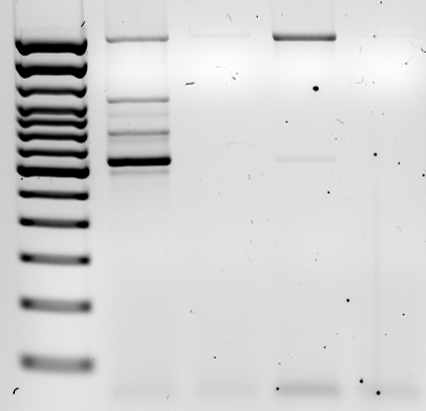

Supplement: Figure 5—source data 2. [file elife-102681-fig5-data2.zip › Figure 5-source_data_2/PSG4.tif]

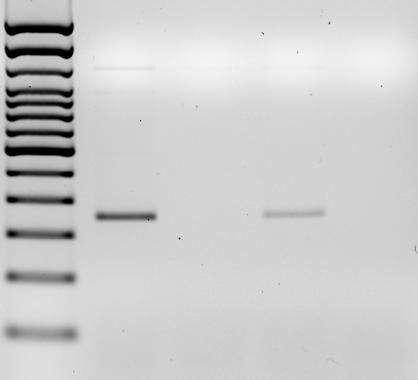

Supplement: Figure 5—source data 2. [file elife-102681-fig5-data2.zip › Figure 5-source_data_2/PSMB9.tif]

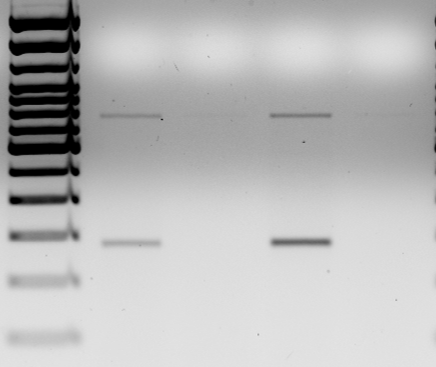

Supplement: Figure 5—source data 2. [file elife-102681-fig5-data2.zip › Figure 5-source_data_2/PTGS2.tif]

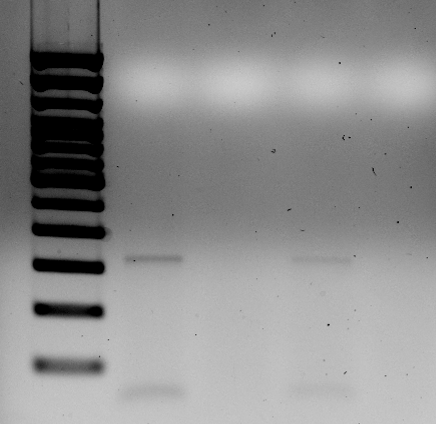

Supplement: Figure 5—source data 2. [file elife-102681-fig5-data2.zip › Figure 5-source_data_2/SERPINB1.tif]

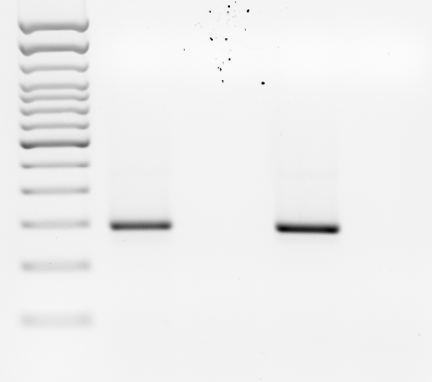

Supplement: Figure 5—source data 2. [file elife-102681-fig5-data2.zip › Figure 5-source_data_2/SERPINB2.tif]

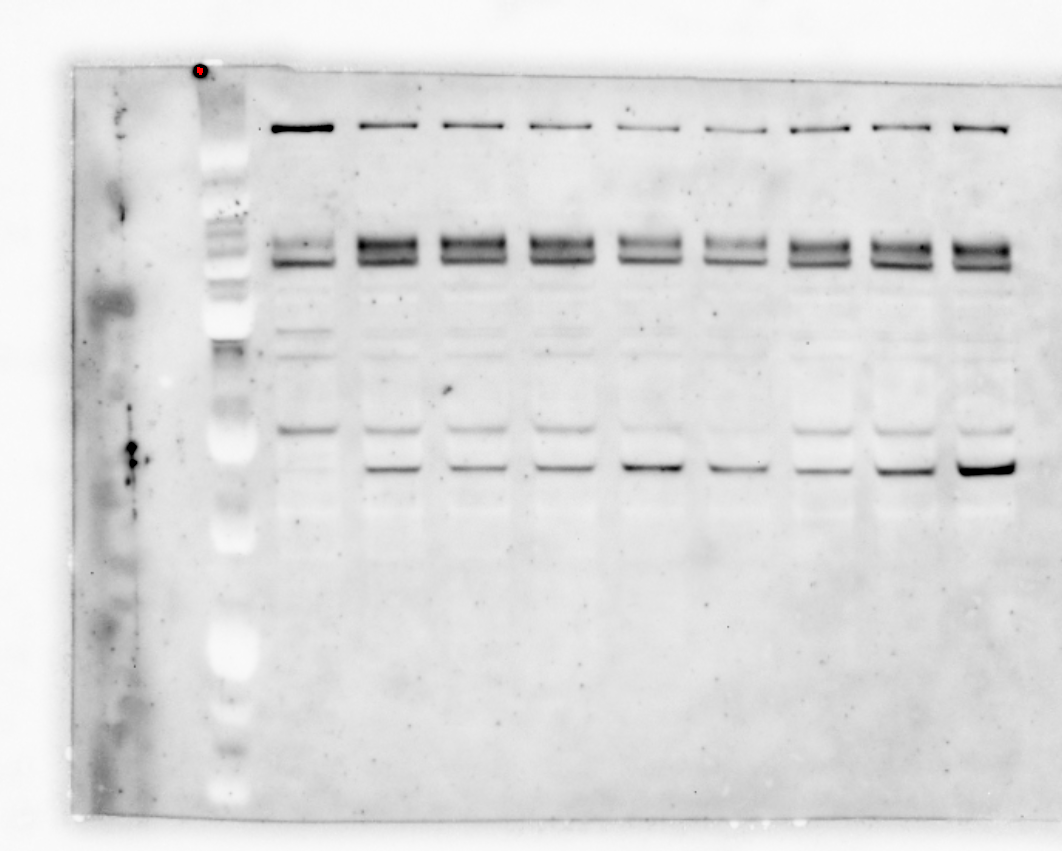

Supplement: Figure 7—source data 2. [file elife-102681-fig7-data2.zip › Figure 7-source_data_2/zheng lab 2020-12-04 12h07m51s.tif]

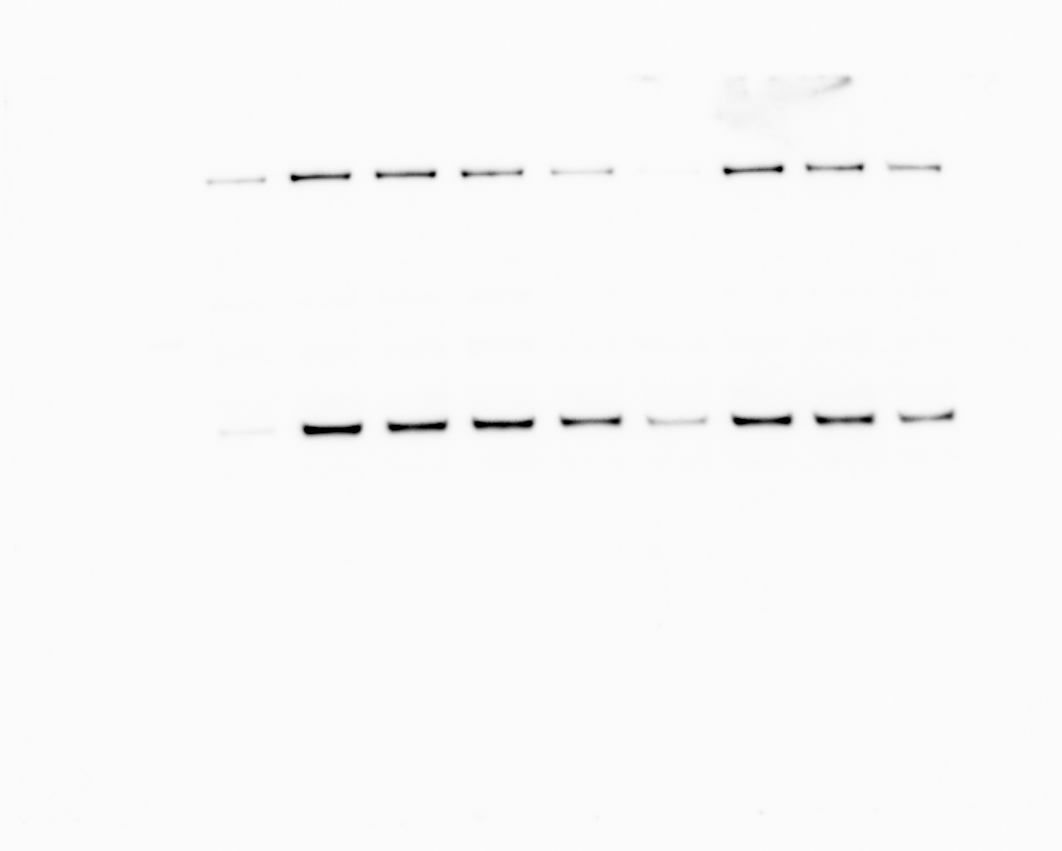

Supplement: Figure 7—source data 2. [file elife-102681-fig7-data2.zip › Figure 7-source_data_2/zheng lab 2020-12-07 11h44m33s.tif]

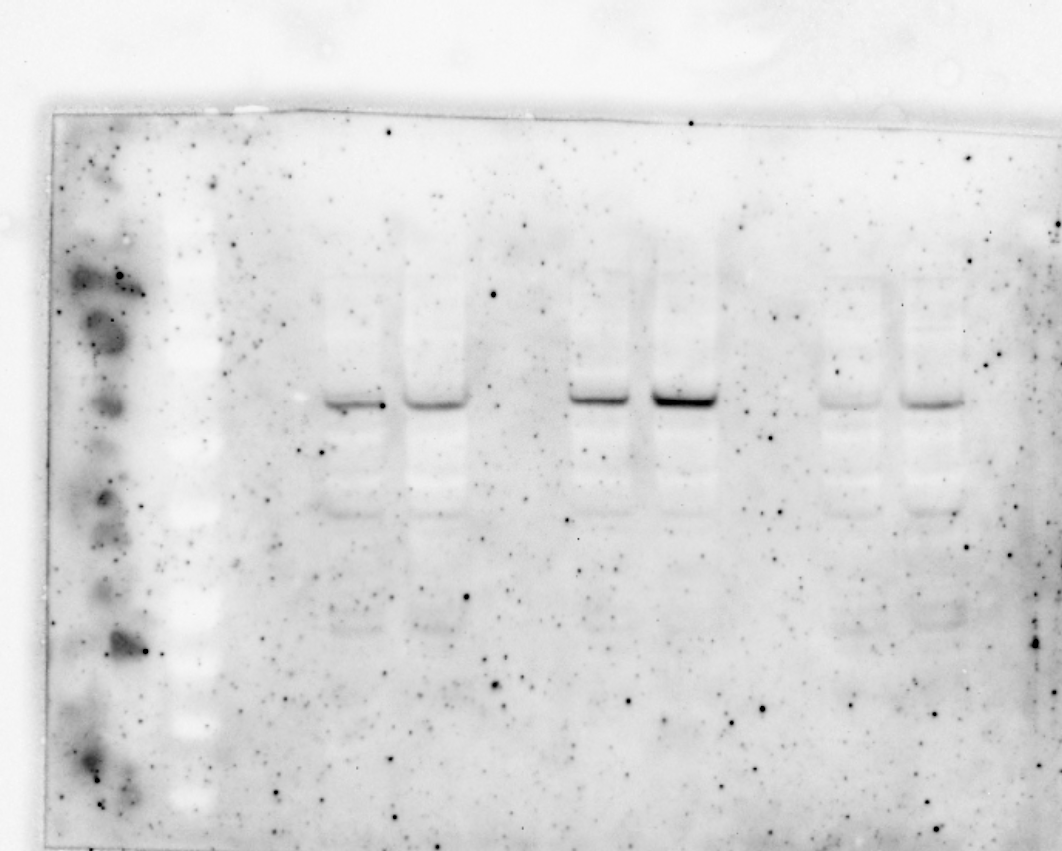

Supplement: Figure 7—source data 2. [file elife-102681-fig7-data2.zip › Figure 7-source_data_2/zheng lab 2020-12-08 12h56m20s.tif]

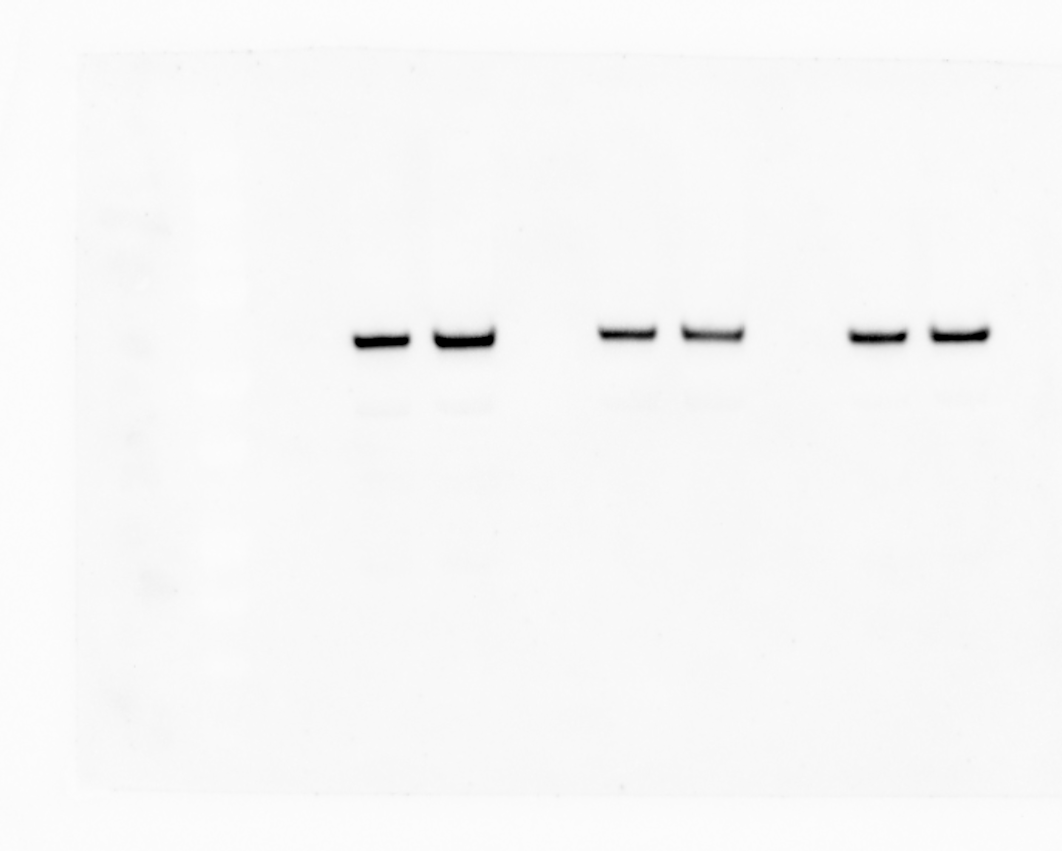

Supplement: Figure 7—source data 2. [file elife-102681-fig7-data2.zip › Figure 7-source_data_2/zheng lab 2020-12-09 13h05m25s.tif]

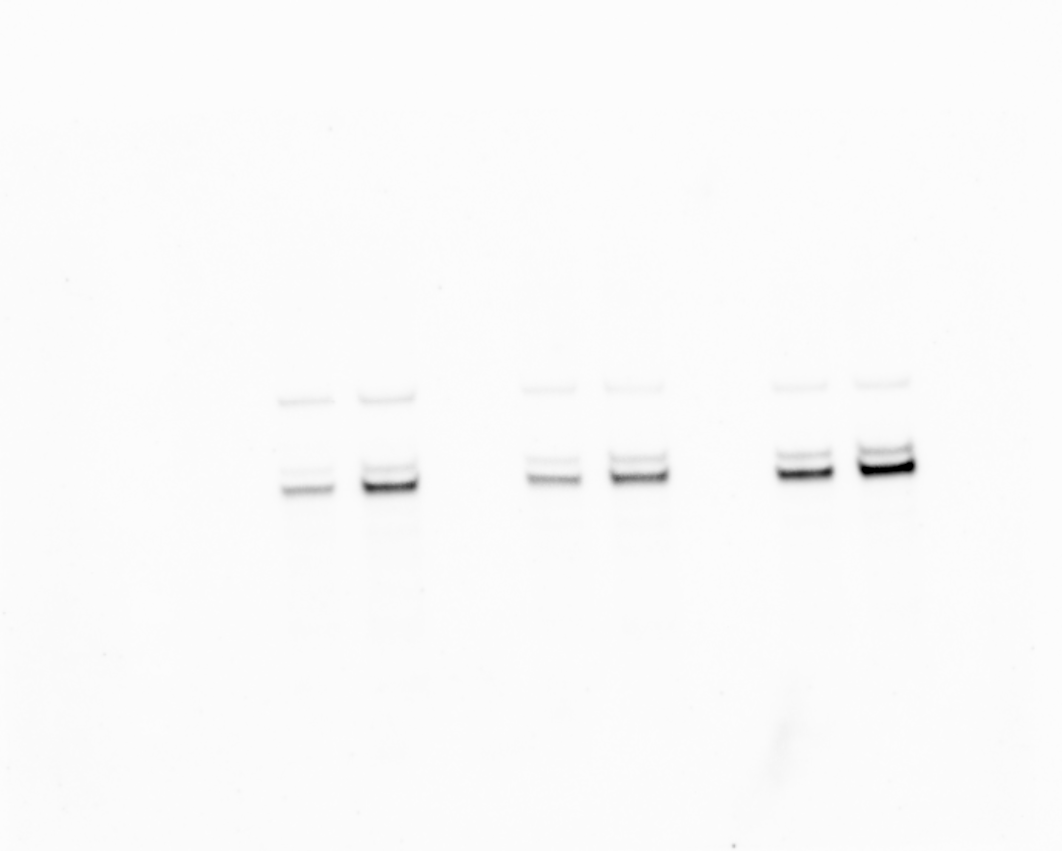

Supplement: Figure 7—source data 2. [file elife-102681-fig7-data2.zip › Figure 7-source_data_2/zheng lab 2020-12-10 14h15m15s.tif]

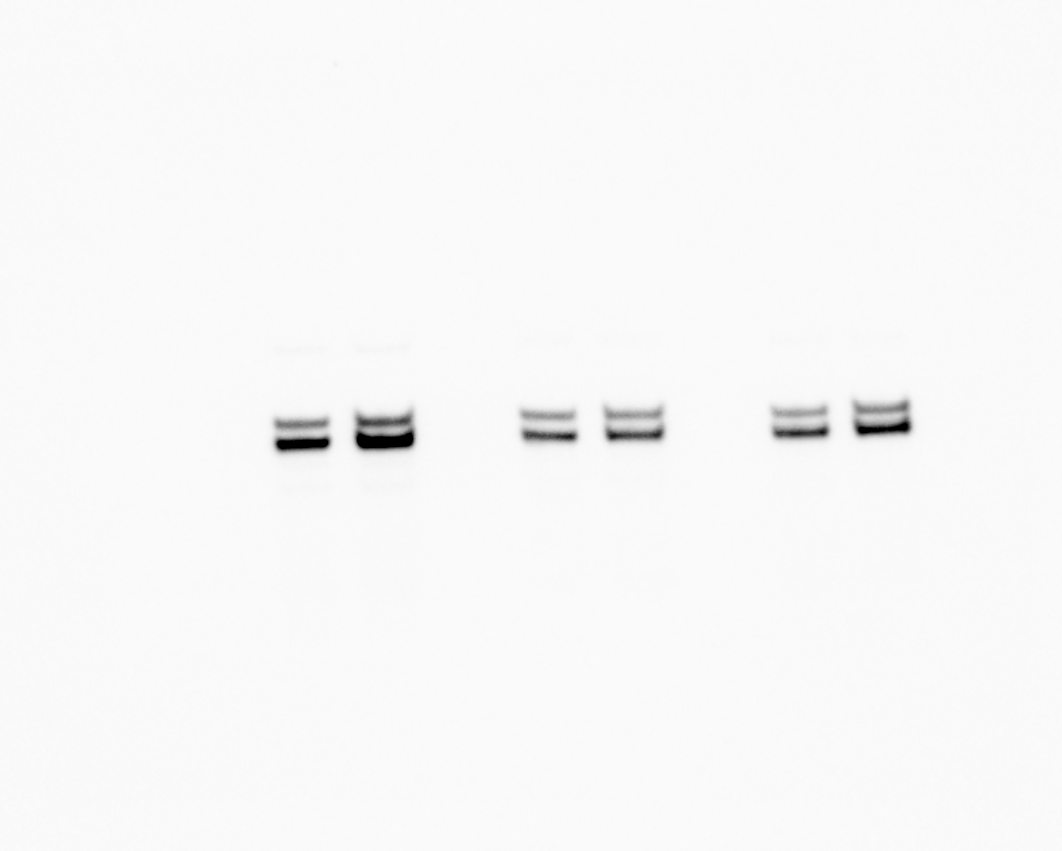

Supplement: Figure 7—source data 2. [file elife-102681-fig7-data2.zip › Figure 7-source_data_2/zheng lab 2020-12-11 12h31m24s.tif]

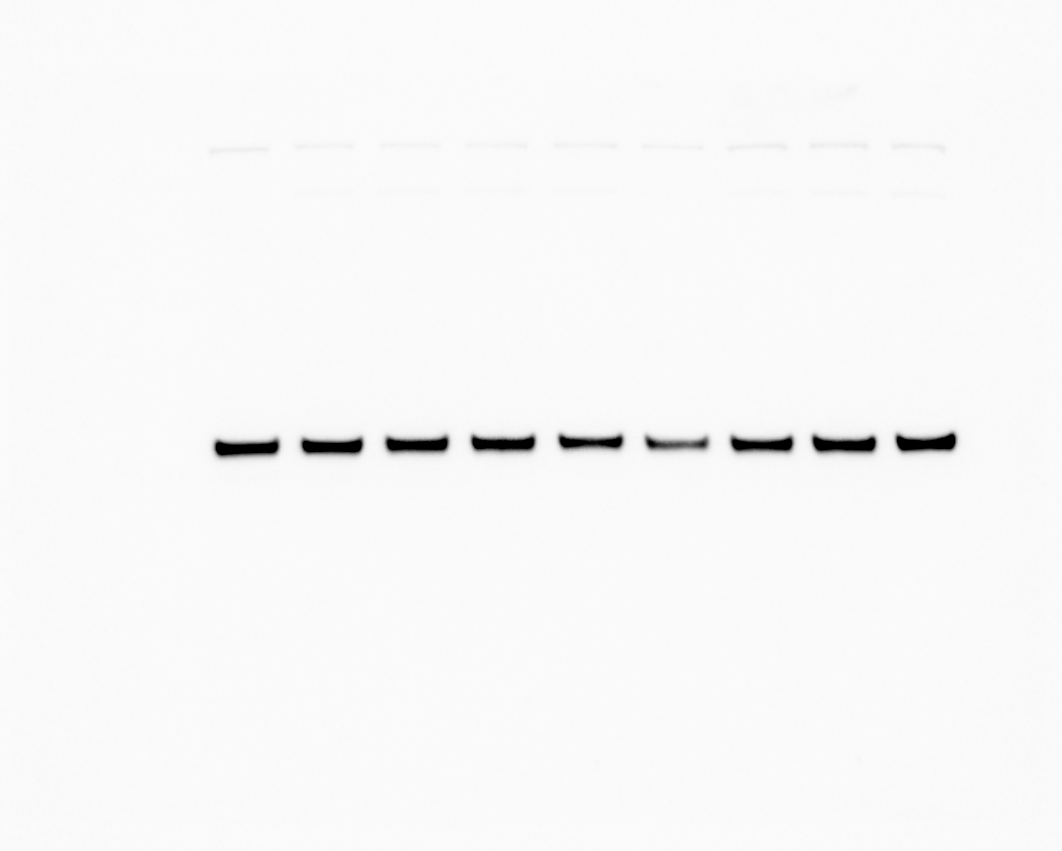

Supplement: Figure 7—source data 2. [file elife-102681-fig7-data2.zip › Figure 7-source_data_2/zheng lab 2020-12-14 13h23m21s.tif]

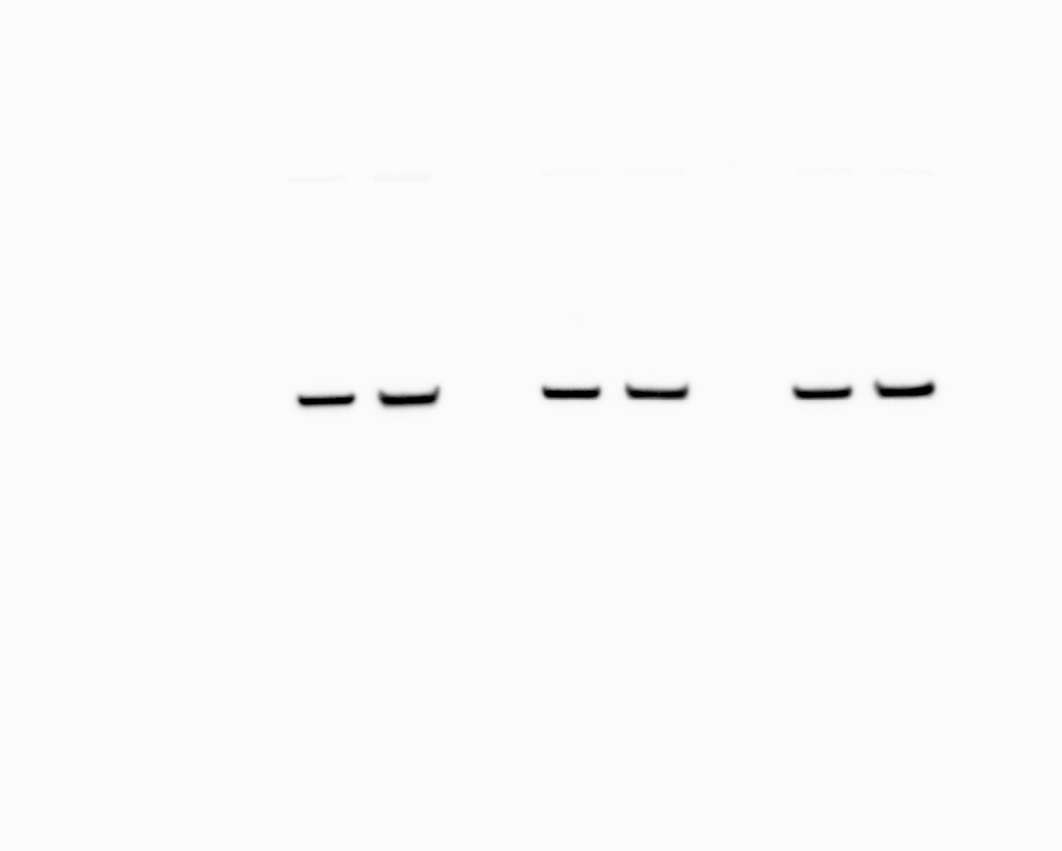

Supplement: Figure 7—source data 2. [file elife-102681-fig7-data2.zip › Figure 7-source_data_2/zheng lab 2020-12-14 13h29m01s.tif]

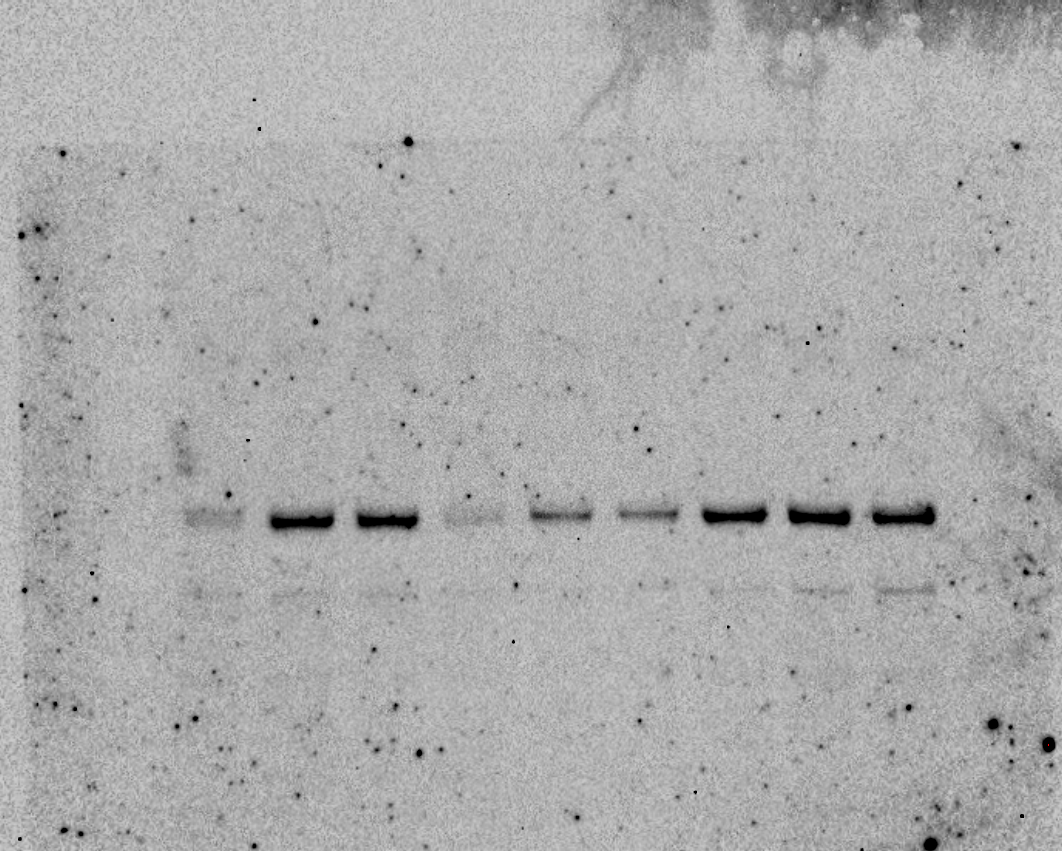

Supplement: Figure 7—source data 2. [file elife-102681-fig7-data2.zip › Figure 7-source_data_2/zheng lab 2020-12-17 13h31m30s.tif]

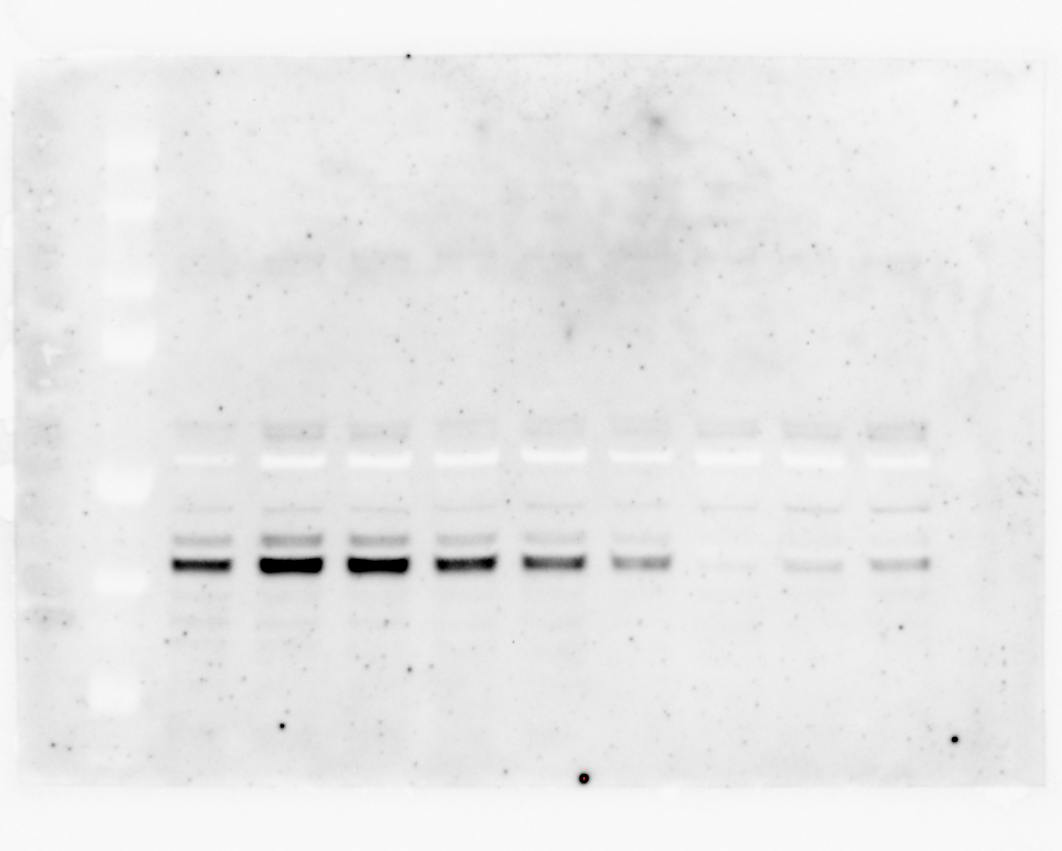

Supplement: Figure 7—source data 2. [file elife-102681-fig7-data2.zip › Figure 7-source_data_2/zheng lab 2020-12-18 13h07m46s.tif]

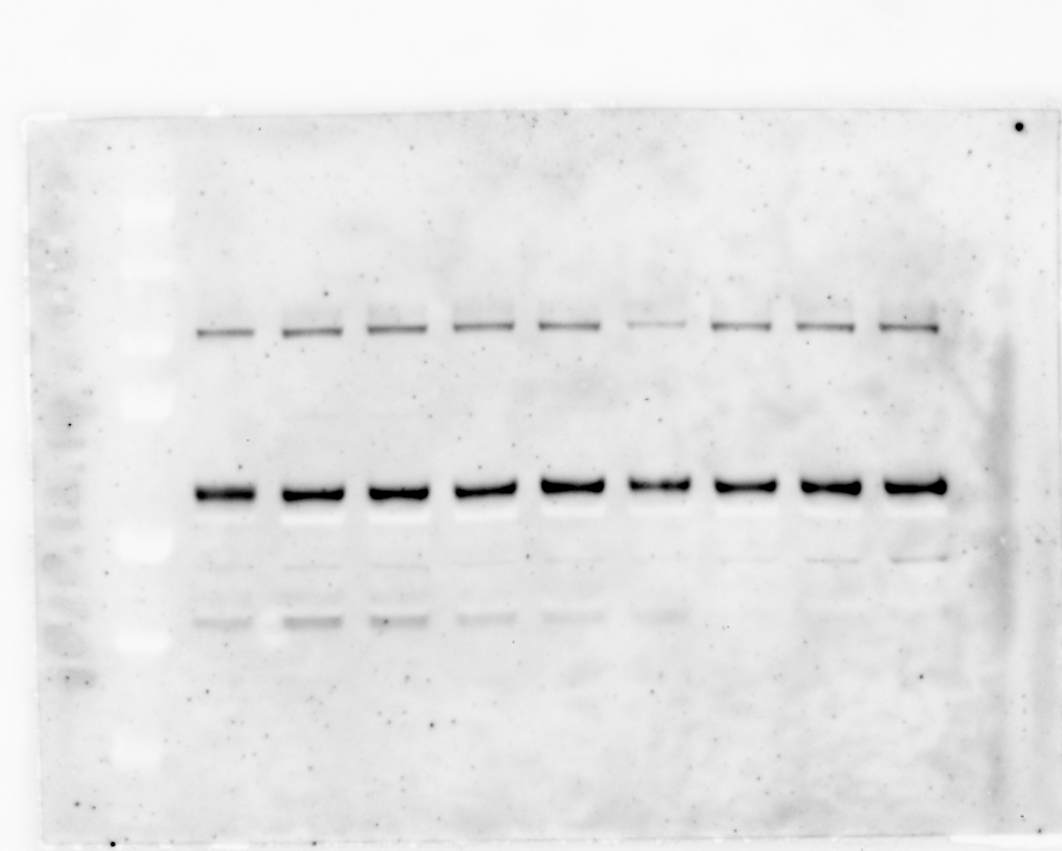

Supplement: Figure 7—source data 2. [file elife-102681-fig7-data2.zip › Figure 7-source_data_2/zheng lab 2020-12-22 13h19m11s.tif]

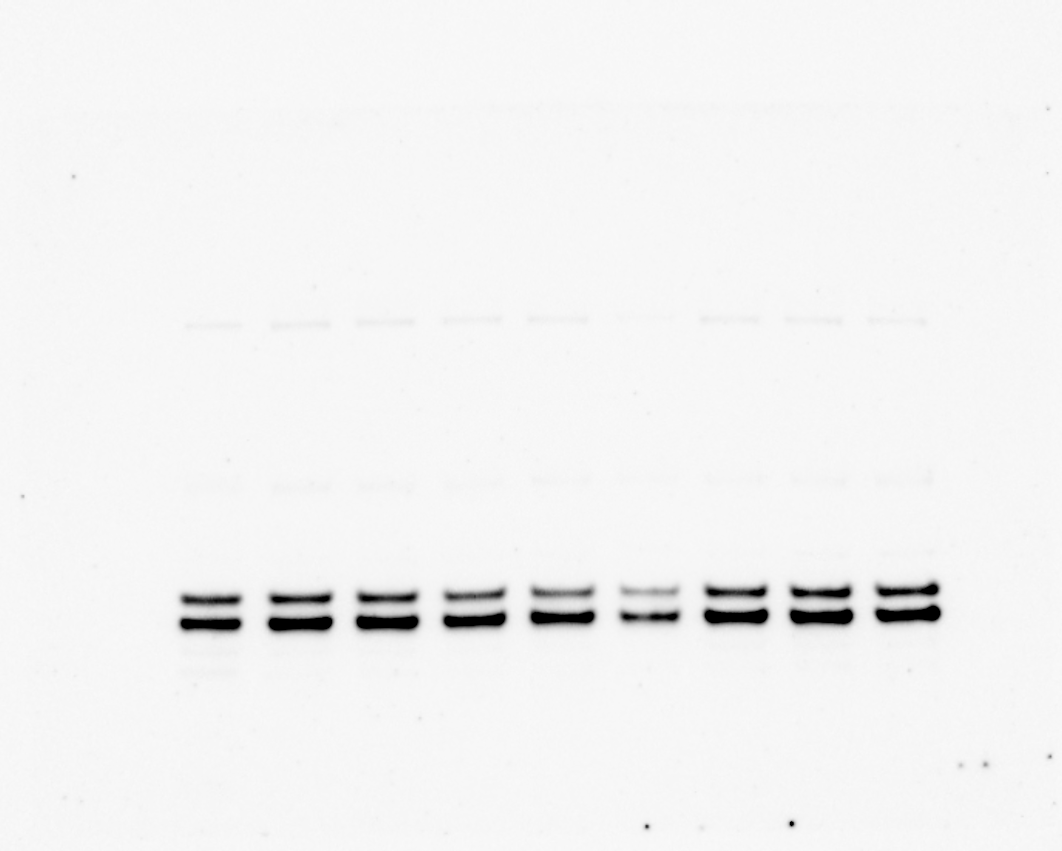

Supplement: Figure 7—source data 2. [file elife-102681-fig7-data2.zip › Figure 7-source_data_2/zheng lab 2020-12-23 11h56m05s.tif]

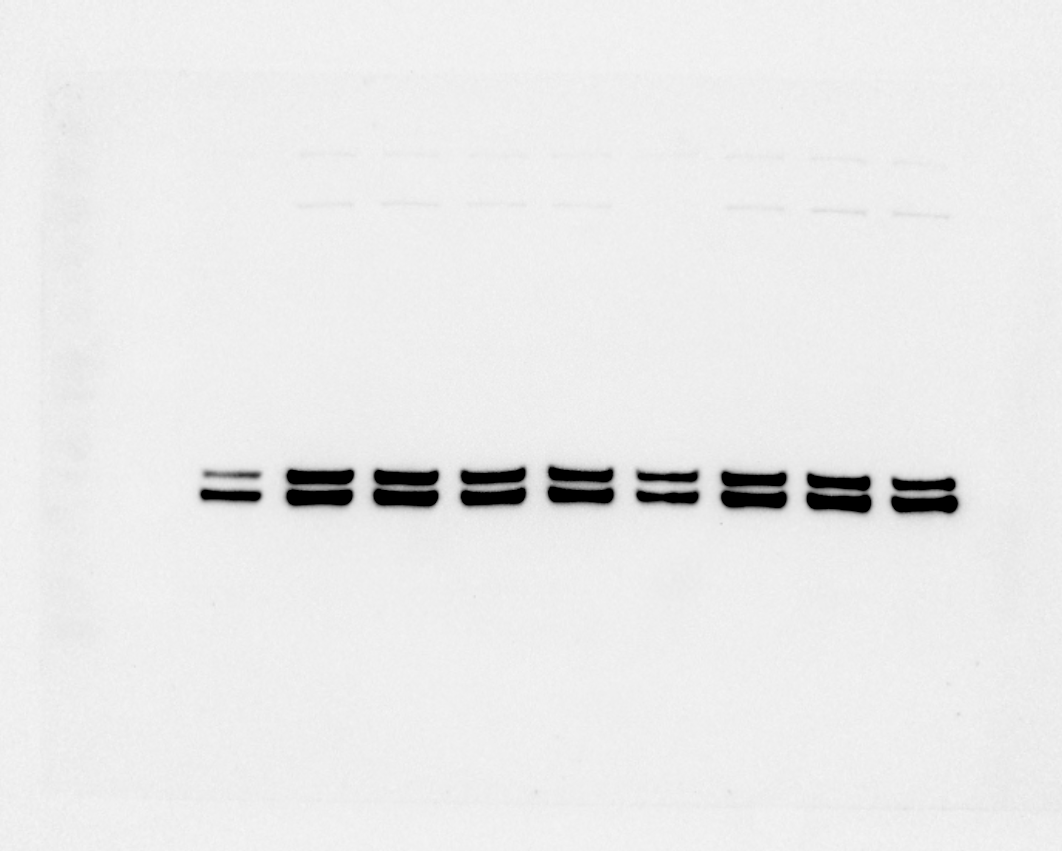

Supplement: Figure 7—source data 2. [file elife-102681-fig7-data2.zip › Figure 7-source_data_2/zheng lab 2020-12-25 13h07m57s.tif]

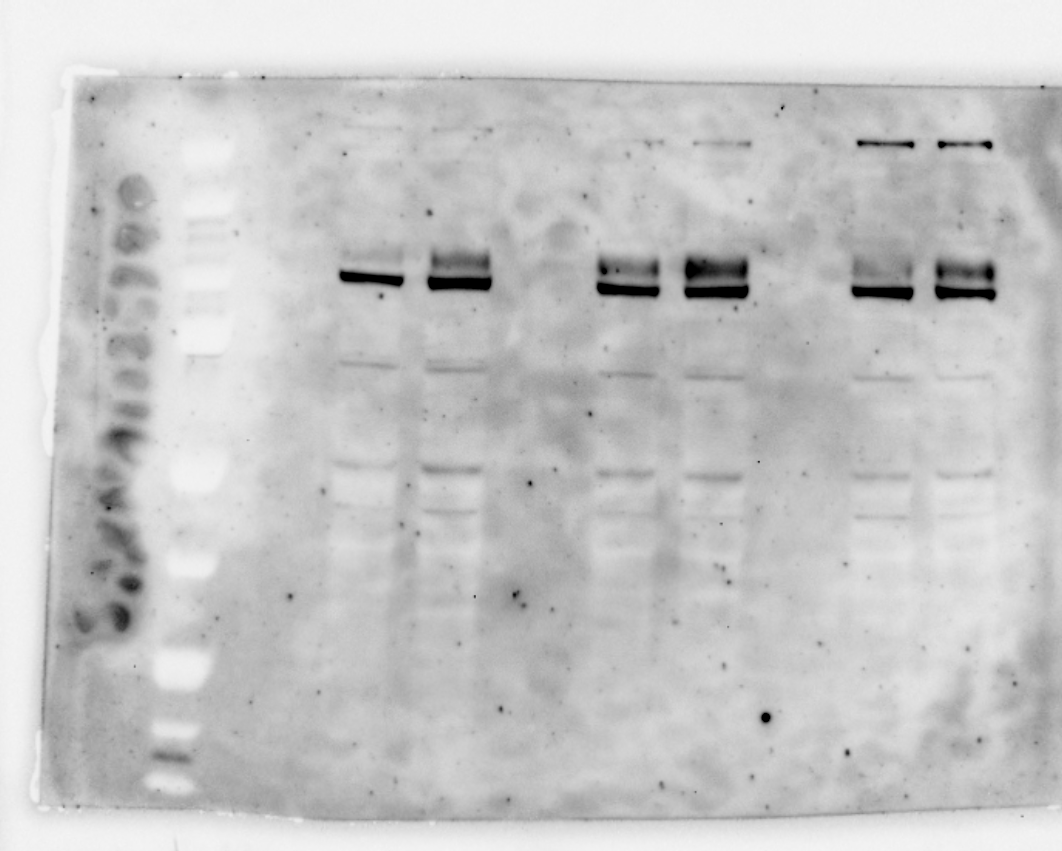

Supplement: Figure 7—source data 2. [file elife-102681-fig7-data2.zip › Figure 7-source_data_2/zheng lab 2020-12-28 14h06m02s.tif]

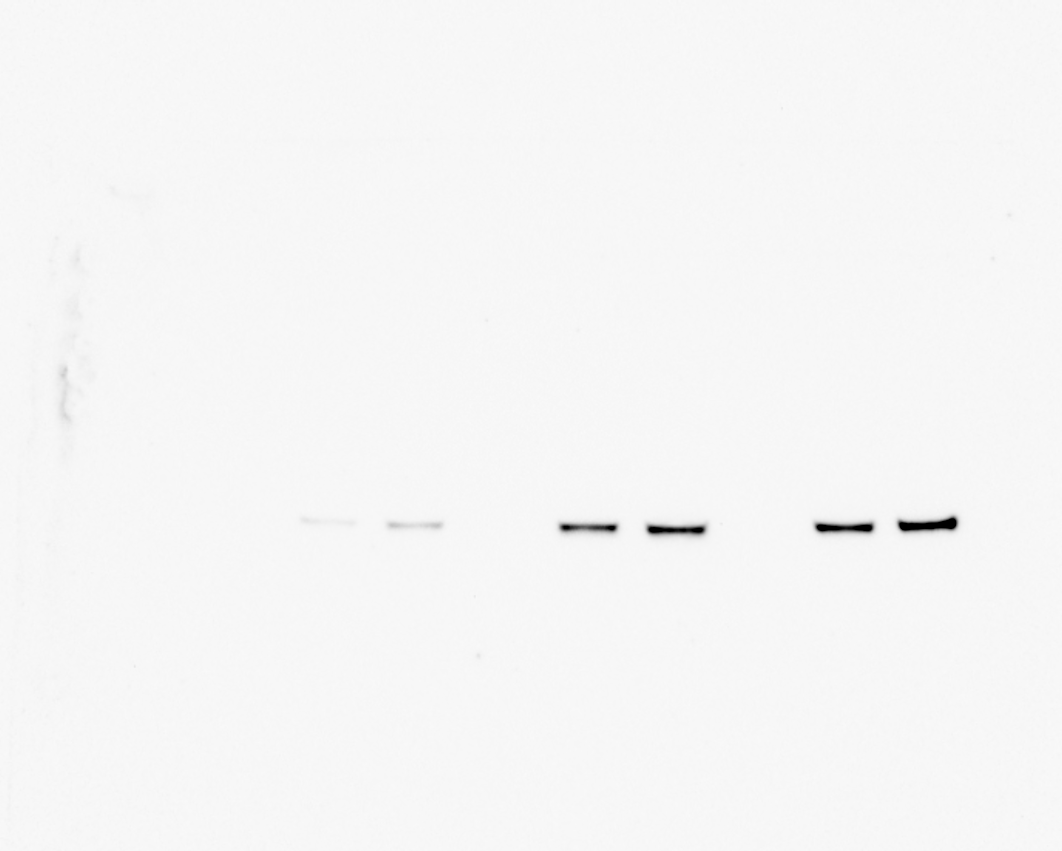

Supplement: Figure 7—source data 2. [file elife-102681-fig7-data2.zip › Figure 7-source_data_2/zheng lab 2020-12-29 12h28m47s.tif]

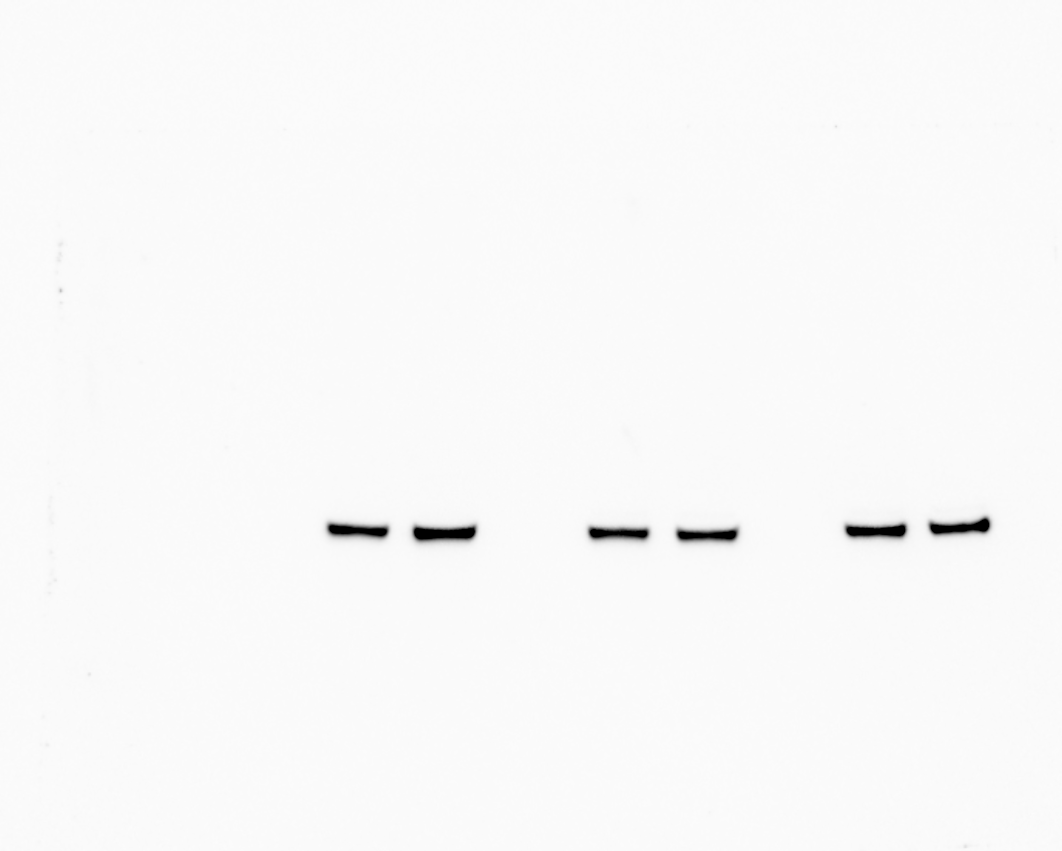

Supplement: Figure 7—source data 2. [file elife-102681-fig7-data2.zip › Figure 7-source_data_2/zheng lab 2020-12-31 13h07m30s.tif]

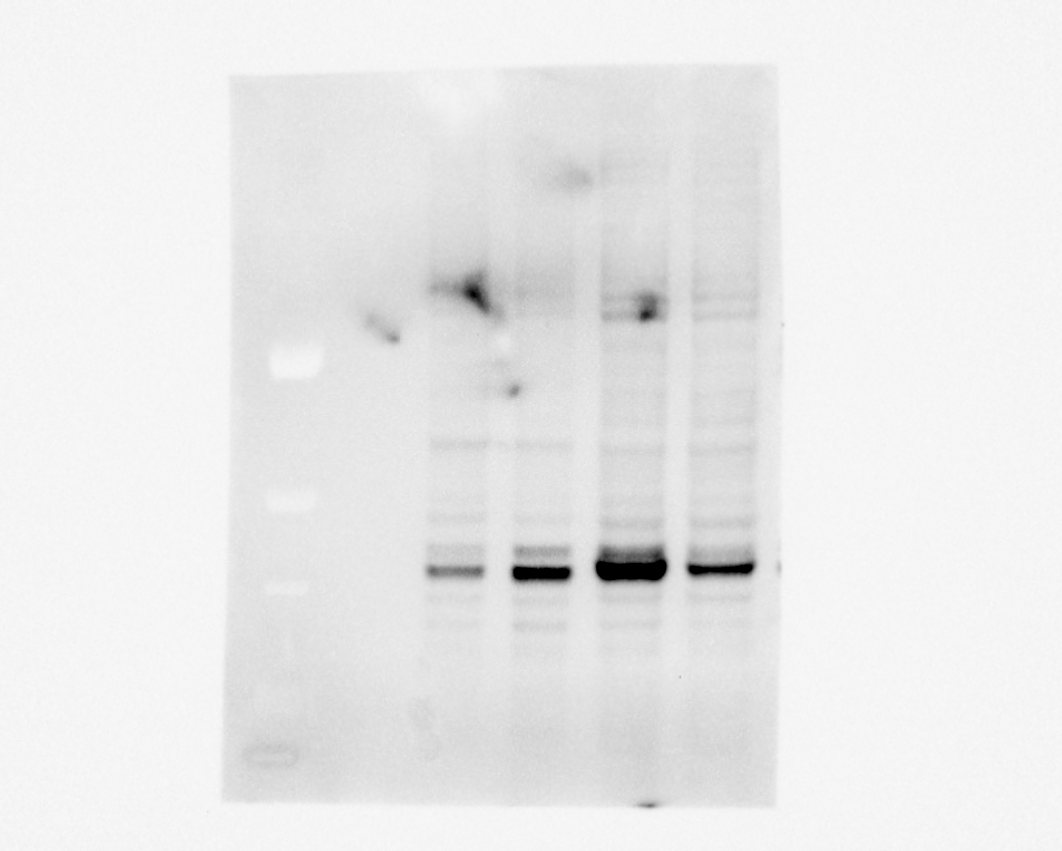

Supplement: Figure 7—source data 2. [file elife-102681-fig7-data2.zip › Figure 7-source_data_2/zheng lab 2021-03-01 13h19m31s.tif]

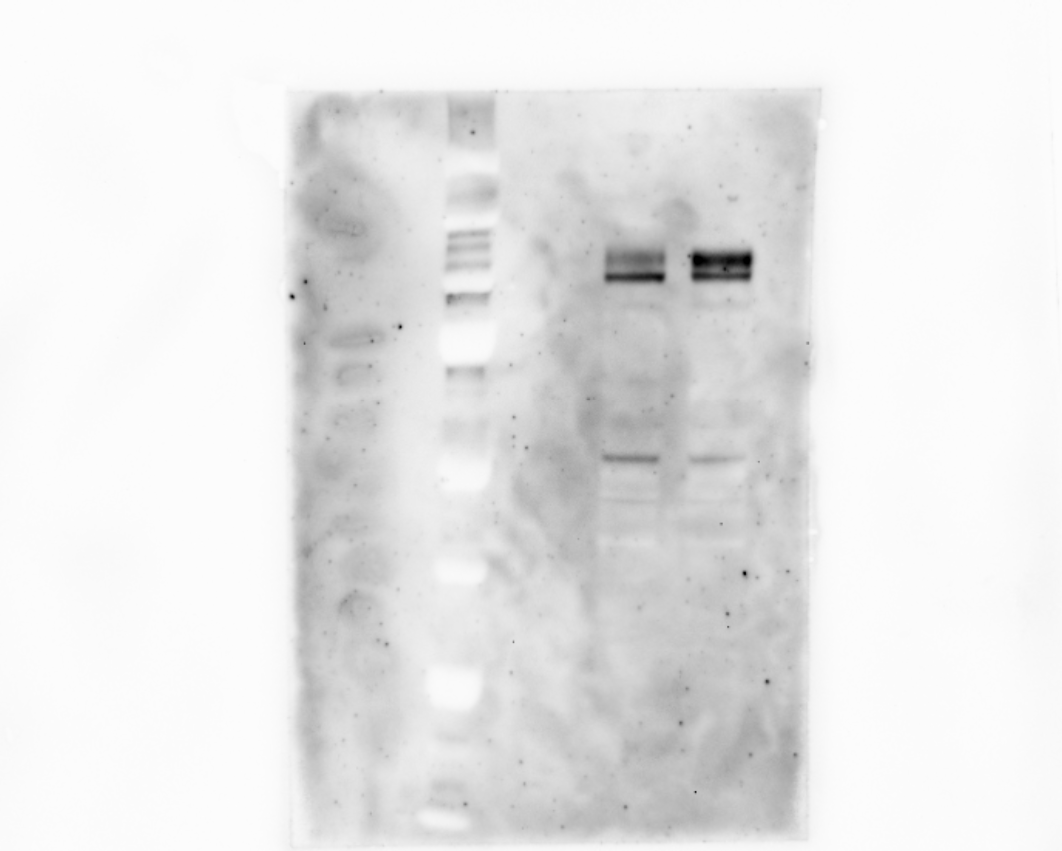

Supplement: Figure 7—source data 2. [file elife-102681-fig7-data2.zip › Figure 7-source_data_2/zheng lab 2021-03-02 15h35m45s.tif]

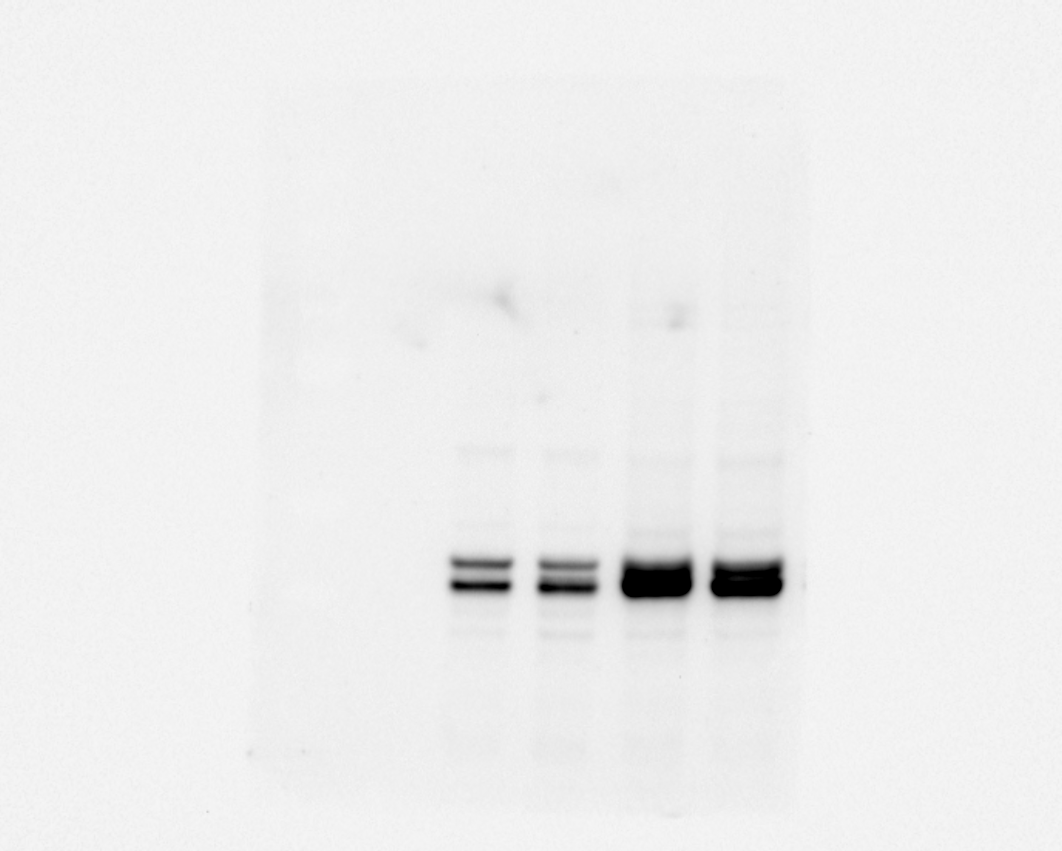

Supplement: Figure 7—source data 2. [file elife-102681-fig7-data2.zip › Figure 7-source_data_2/zheng lab 2021-03-03 12h42m34s.tif]

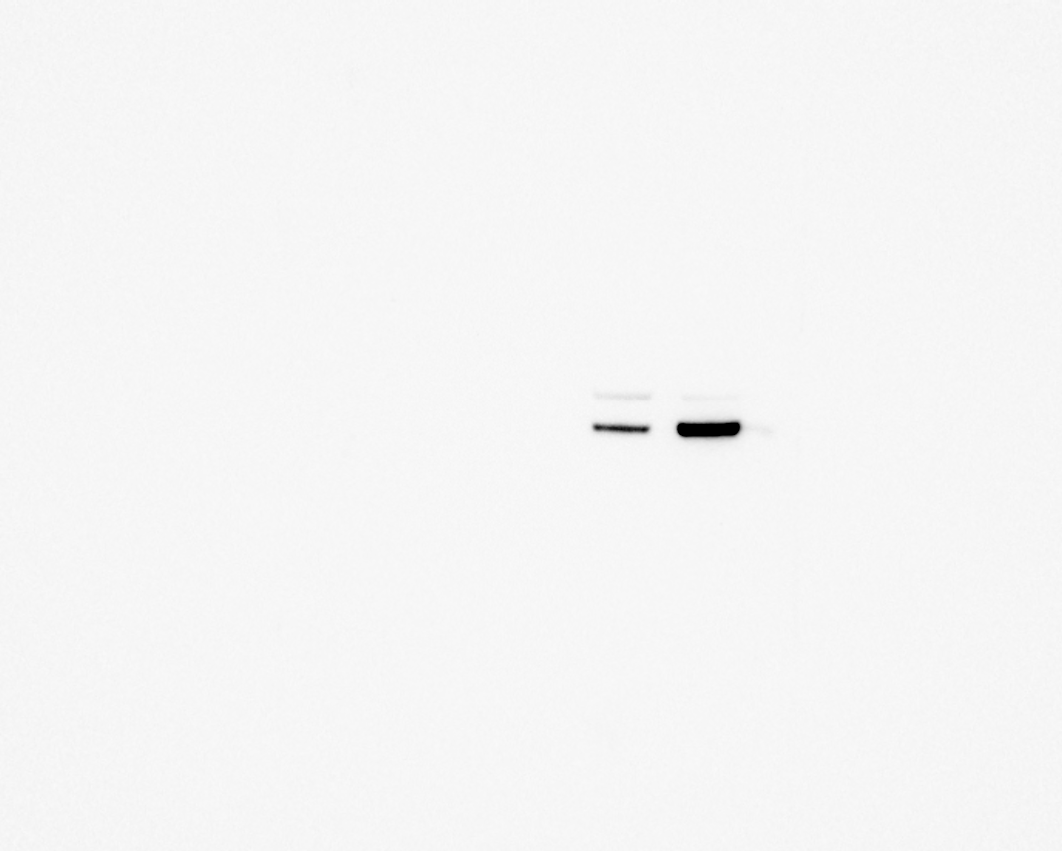

Supplement: Figure 7—source data 2. [file elife-102681-fig7-data2.zip › Figure 7-source_data_2/zheng lab 2021-03-04 12h32m54s.tif]

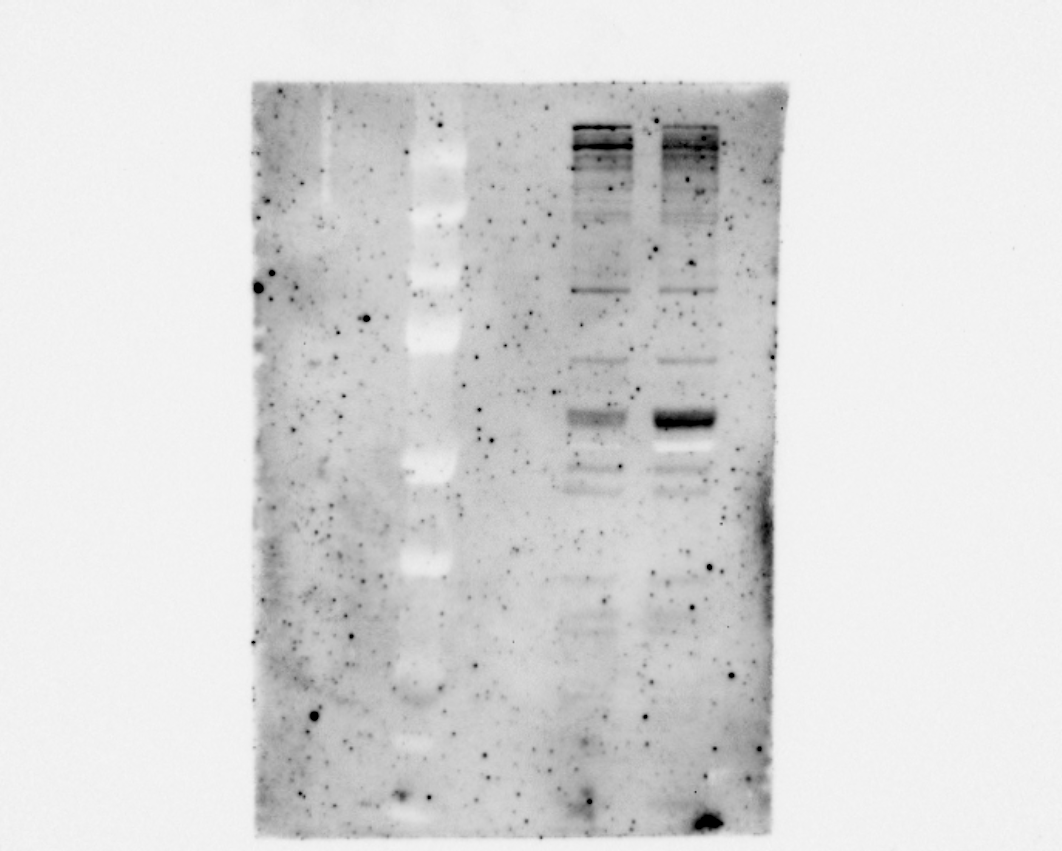

Supplement: Figure 7—source data 2. [file elife-102681-fig7-data2.zip › Figure 7-source_data_2/zheng lab 2021-03-05 13h07m23s.tif]

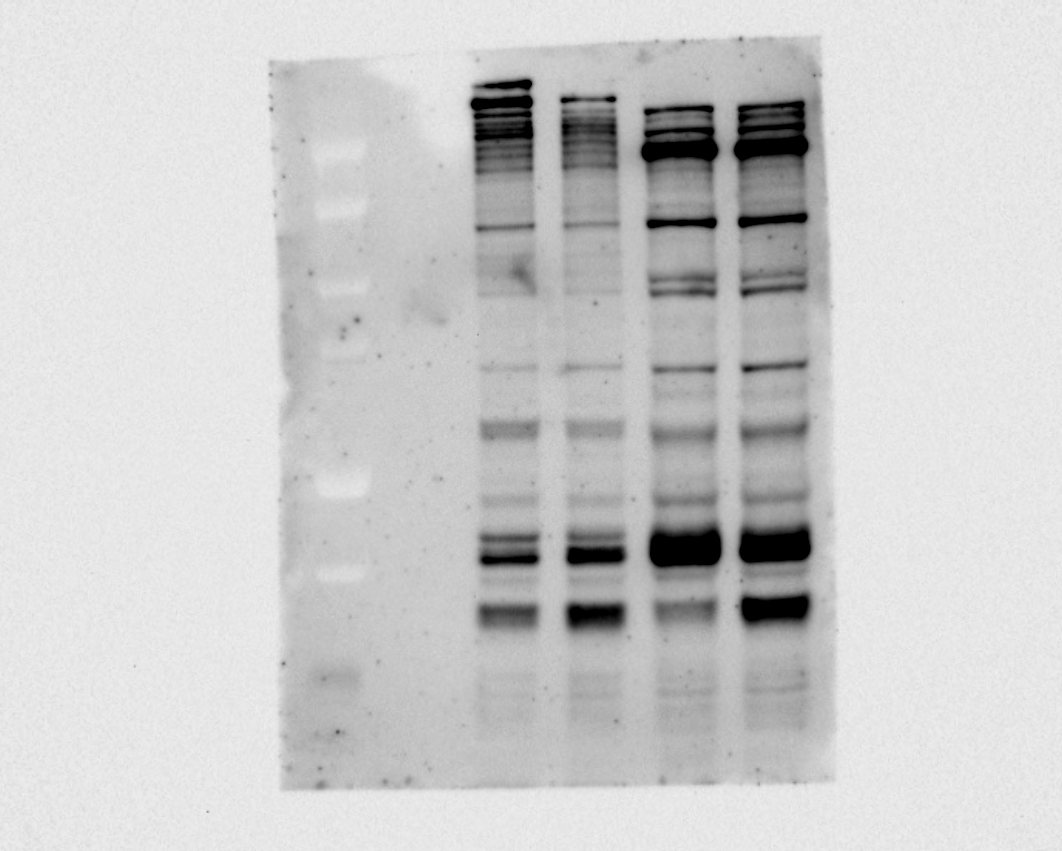

Supplement: Figure 7—source data 2. [file elife-102681-fig7-data2.zip › Figure 7-source_data_2/zheng lab 2021-03-08 13h32m09s.tif]

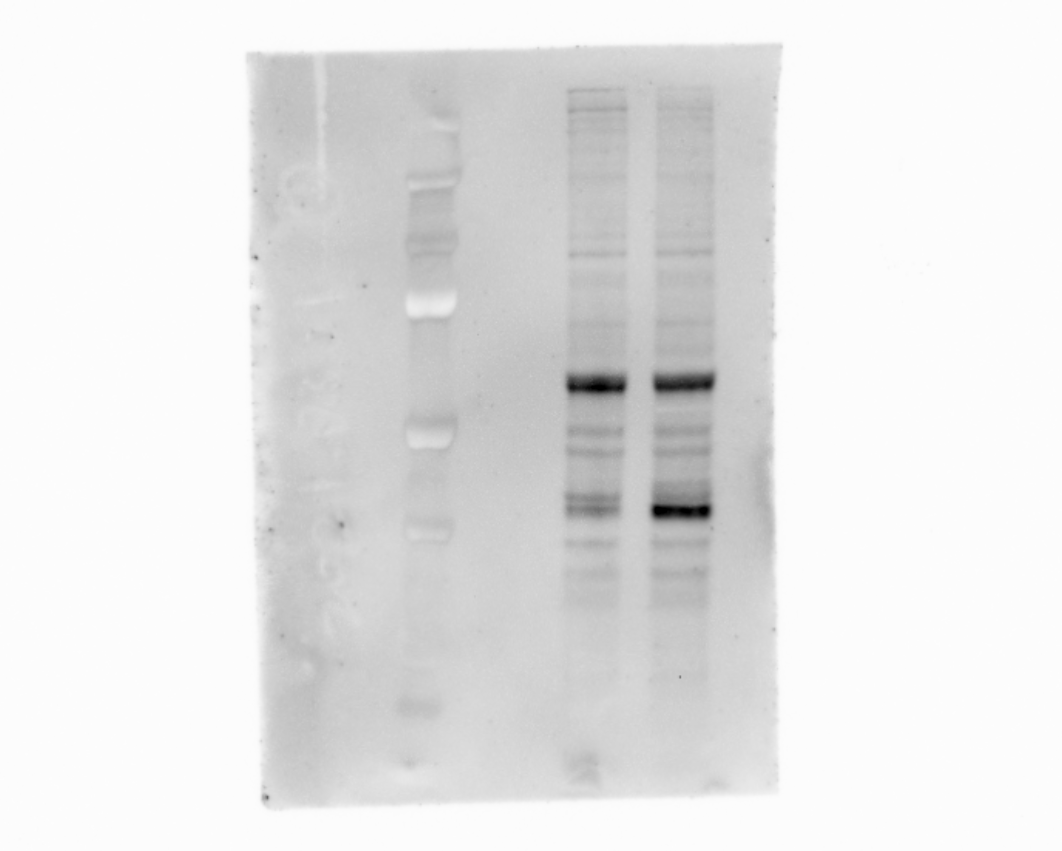

Supplement: Figure 7—source data 2. [file elife-102681-fig7-data2.zip › Figure 7-source_data_2/zheng lab 2021-03-09 16h09m39s.tif]

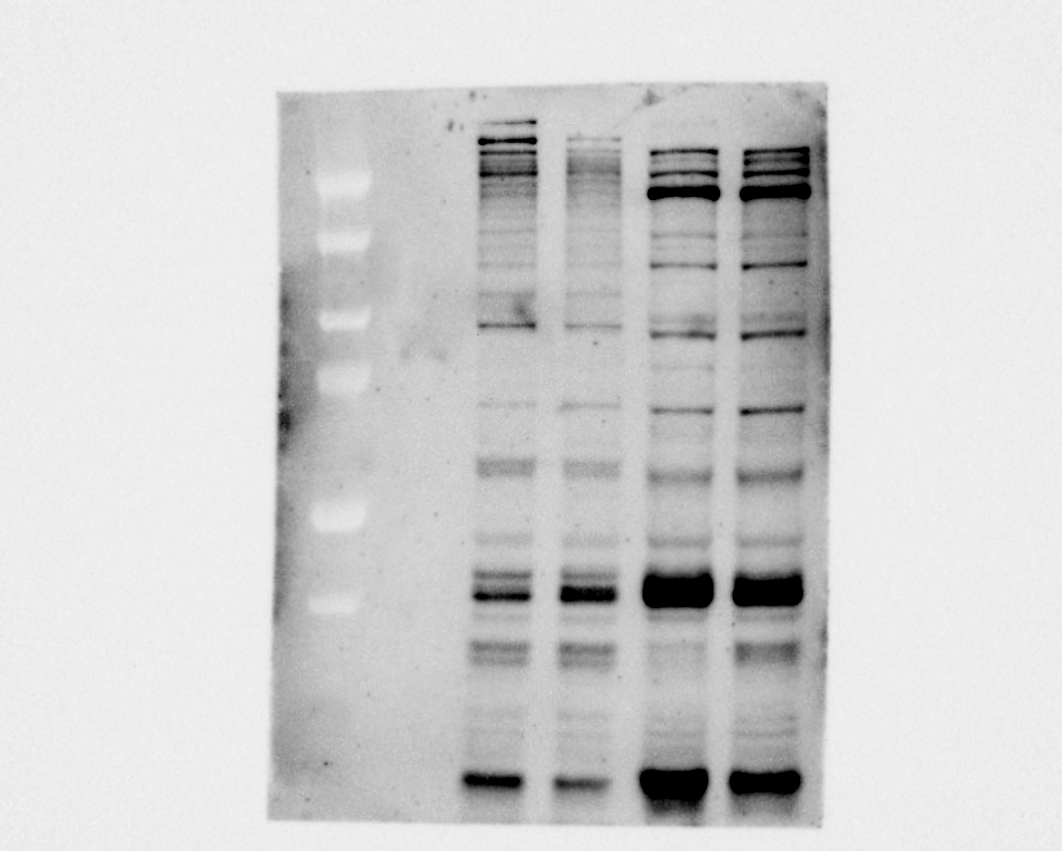

Supplement: Figure 7—source data 2. [file elife-102681-fig7-data2.zip › Figure 7-source_data_2/zheng lab 2021-03-09 16h15m46s.tif]

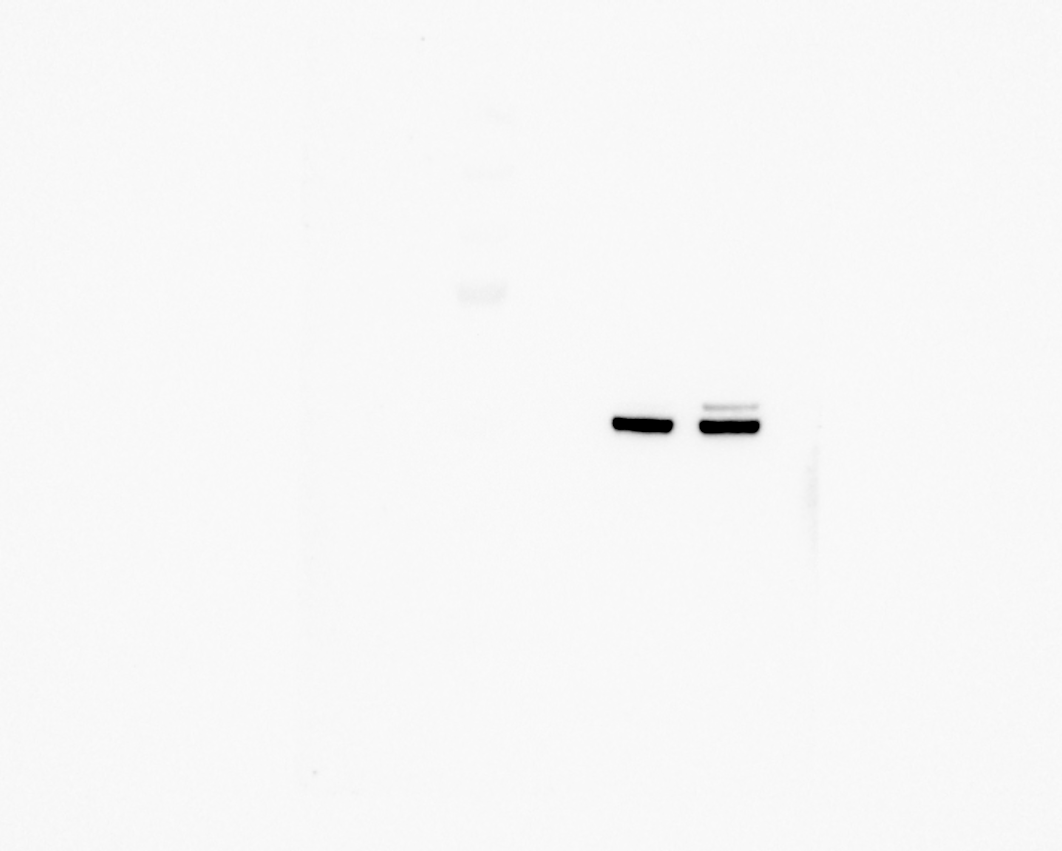

Supplement: Figure 7—source data 2. [file elife-102681-fig7-data2.zip › Figure 7-source_data_2/zheng lab 2021-03-10 12h39m51s.tif]

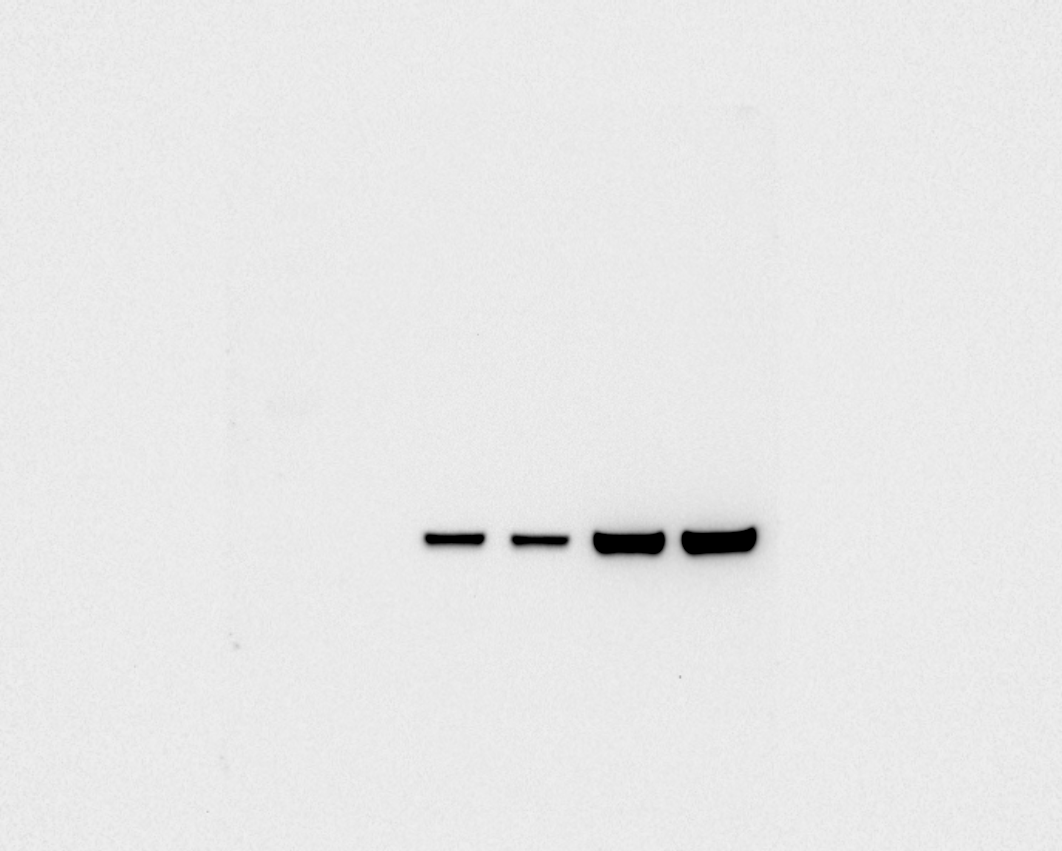

Supplement: Figure 7—source data 2. [file elife-102681-fig7-data2.zip › Figure 7-source_data_2/zheng lab 2021-03-10 12h44m35s.tif]

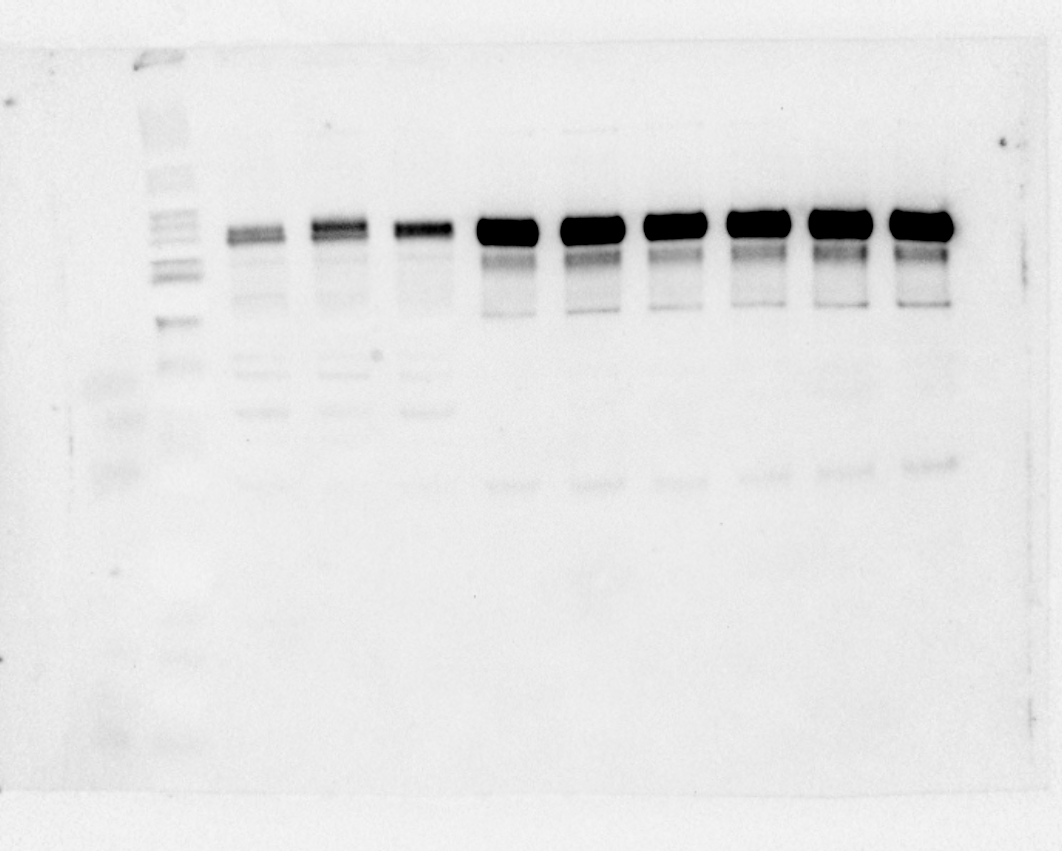

Supplement: Figure 7—figure supplement 2—source data 2. [file elife-102681-fig7-figsupp2-data2.zip › Figure 7-figure supplement 2-source_data_2/zheng lab 2020-07-20 15h27m02s.tif]

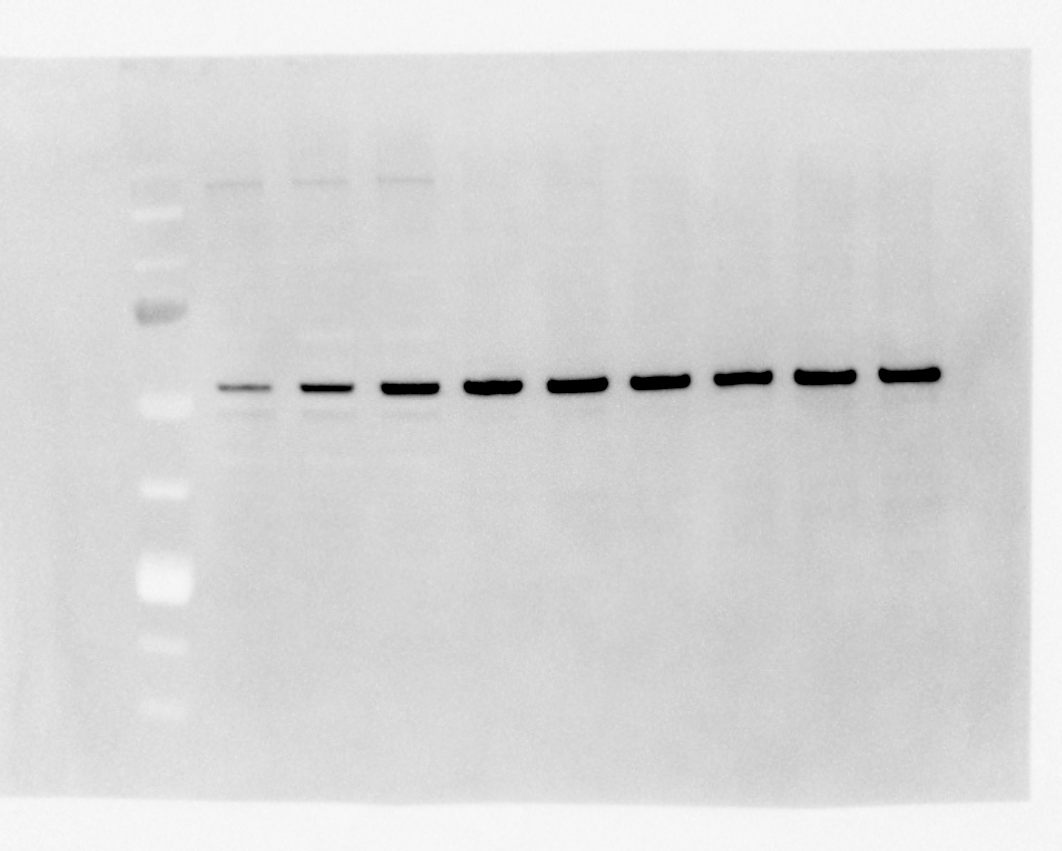

Supplement: Figure 7—figure supplement 2—source data 2. [file elife-102681-fig7-figsupp2-data2.zip › Figure 7-figure supplement 2-source_data_2/zheng lab 2020-07-21 09h29m17s.tif]

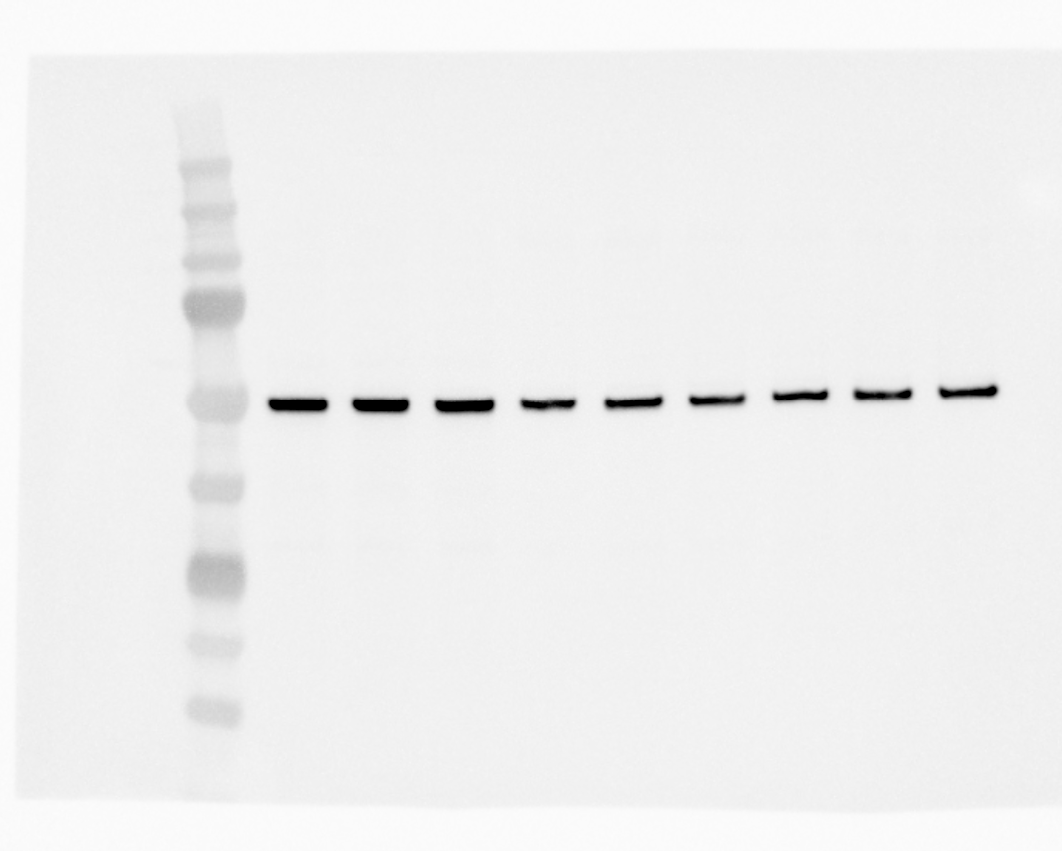

Supplement: Figure 7—figure supplement 2—source data 2. [file elife-102681-fig7-figsupp2-data2.zip › Figure 7-figure supplement 2-source_data_2/zheng lab 2020-07-22 09h28m10s.tif]

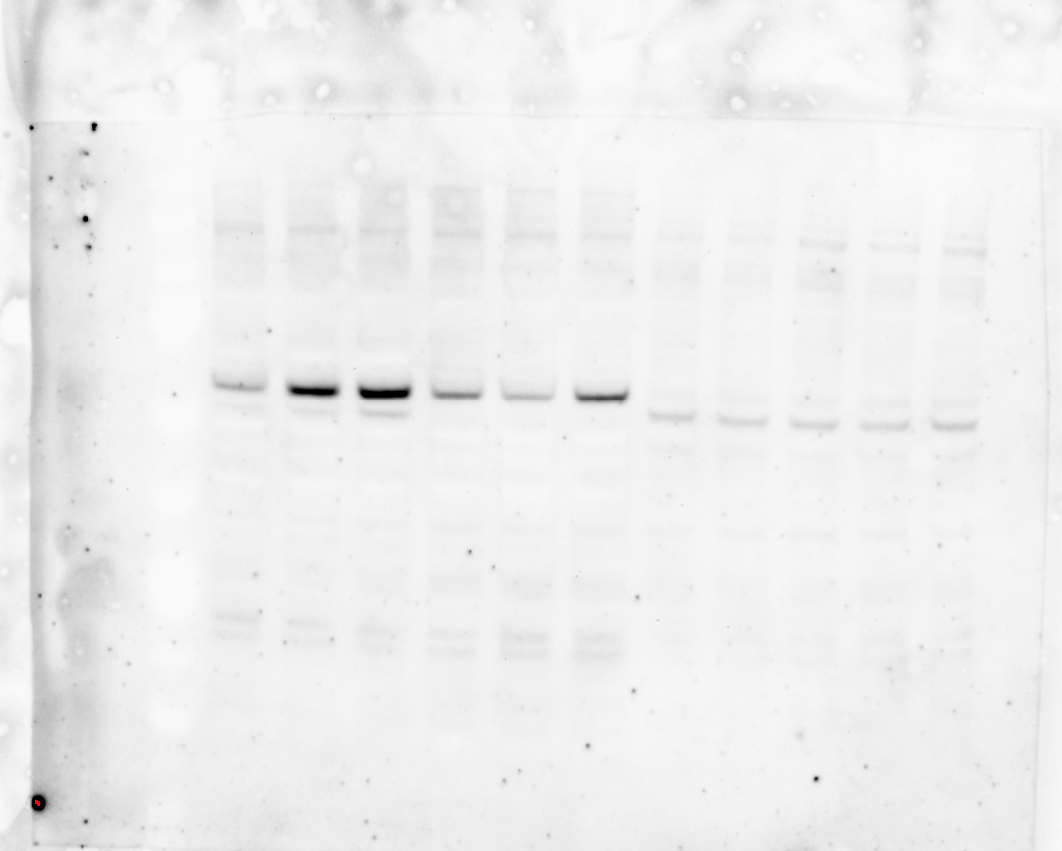

Supplement: Figure 7—figure supplement 2—source data 2. [file elife-102681-fig7-figsupp2-data2.zip › Figure 7-figure supplement 2-source_data_2/zheng lab 2020-07-28 15h16m17s.tif]

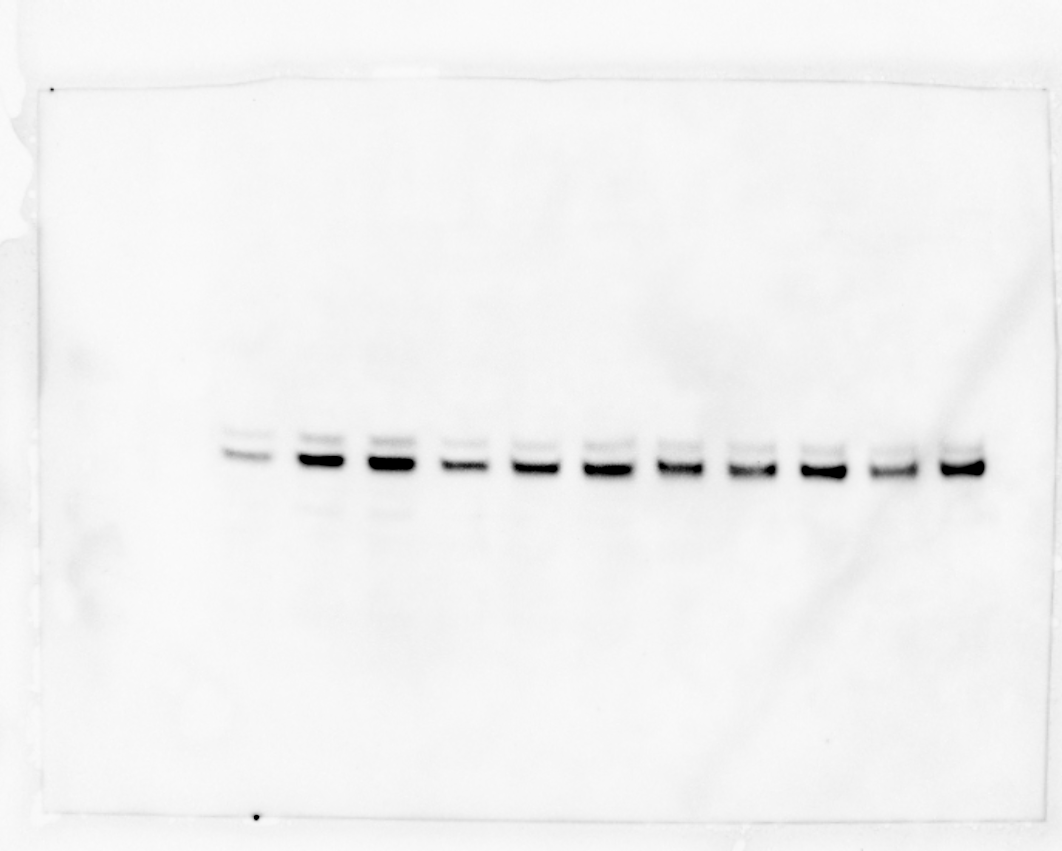

Supplement: Figure 7—figure supplement 2—source data 2. [file elife-102681-fig7-figsupp2-data2.zip › Figure 7-figure supplement 2-source_data_2/zheng lab 2020-07-29 09h17m15s.tif]

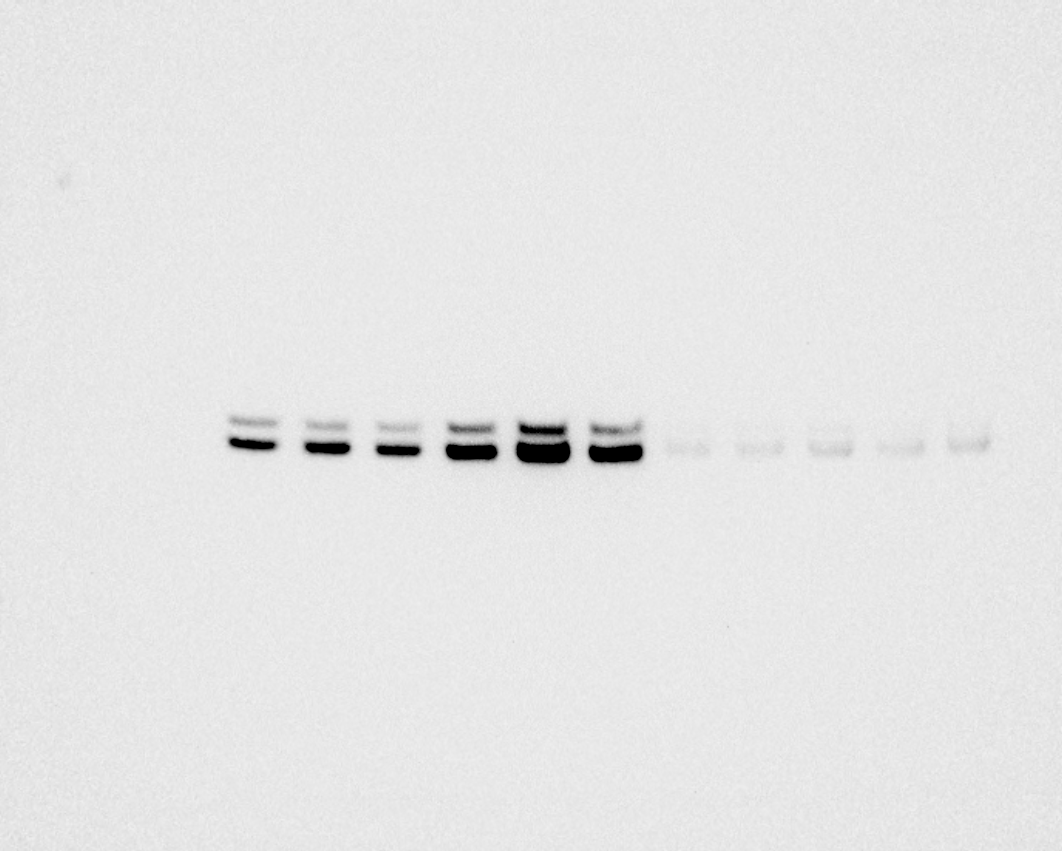

Supplement: Figure 7—figure supplement 2—source data 2. [file elife-102681-fig7-figsupp2-data2.zip › Figure 7-figure supplement 2-source_data_2/zheng lab 2020-07-30 15h00m51s.tif]

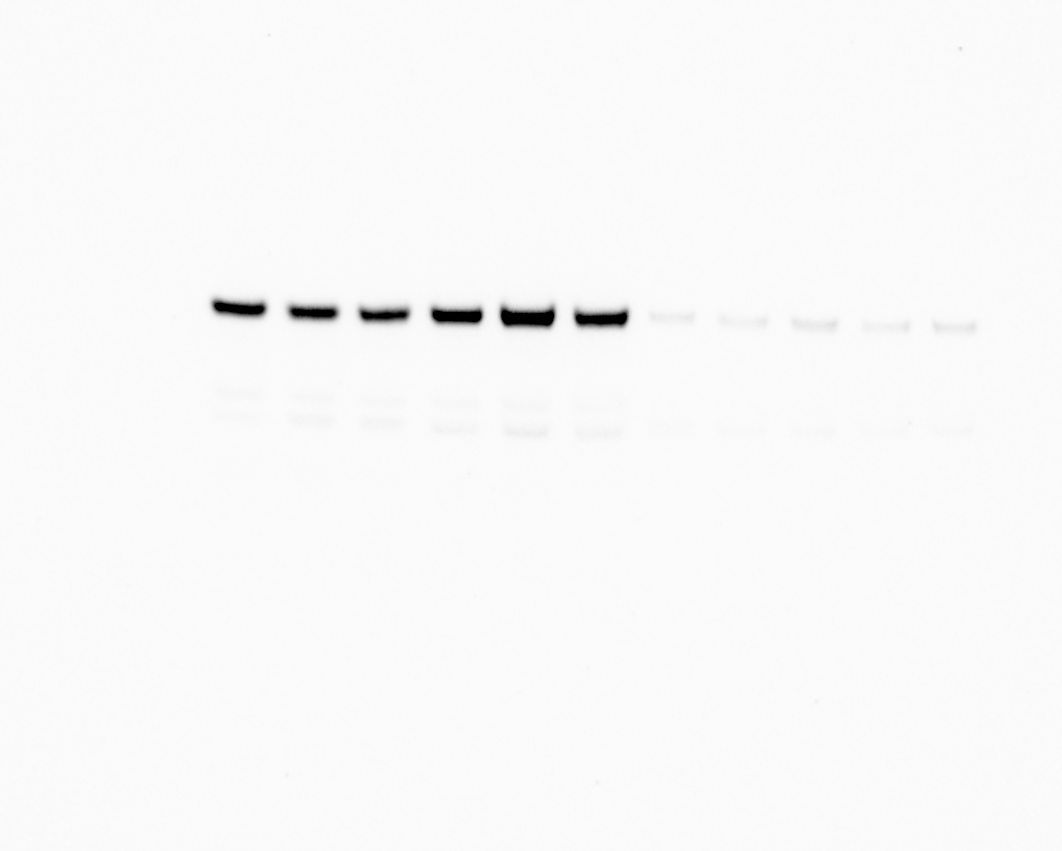

Supplement: Figure 7—figure supplement 2—source data 2. [file elife-102681-fig7-figsupp2-data2.zip › Figure 7-figure supplement 2-source_data_2/zheng lab 2020-07-31 14h36m14s.tif]

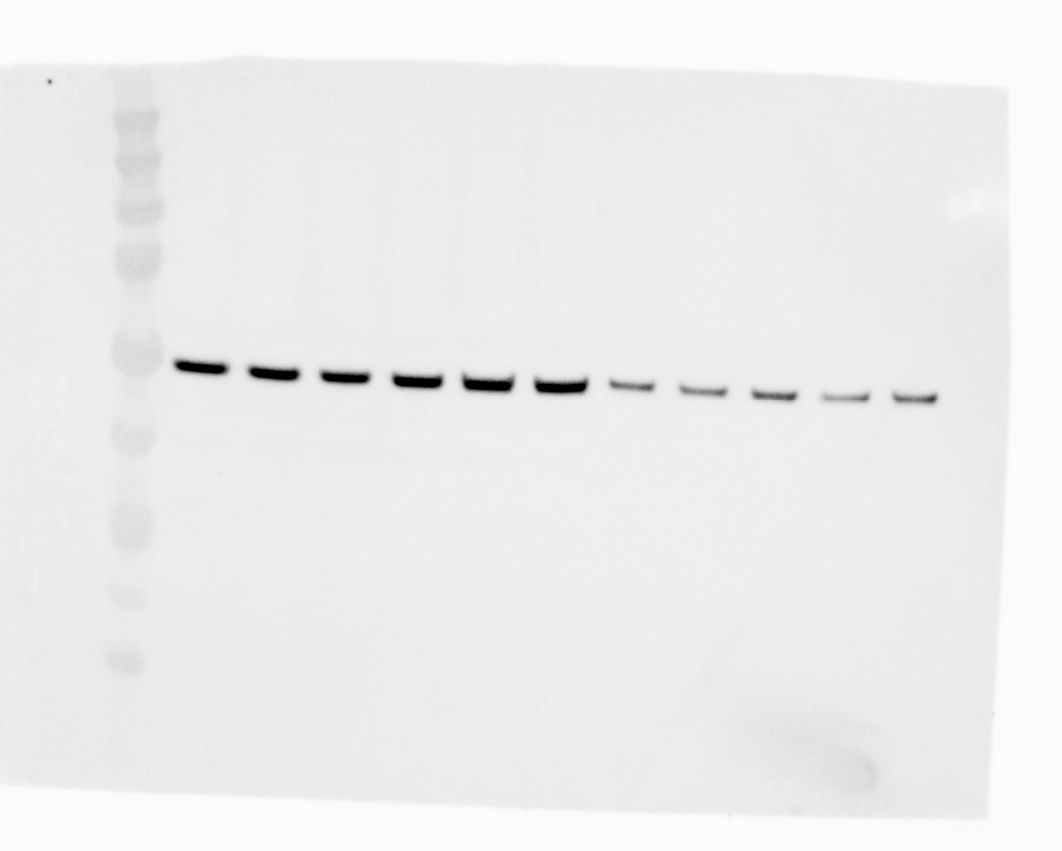

Supplement: Figure 7—figure supplement 2—source data 2. [file elife-102681-fig7-figsupp2-data2.zip › Figure 7-figure supplement 2-source_data_2/zheng lab 2020-08-02 16h05m47s.tif]

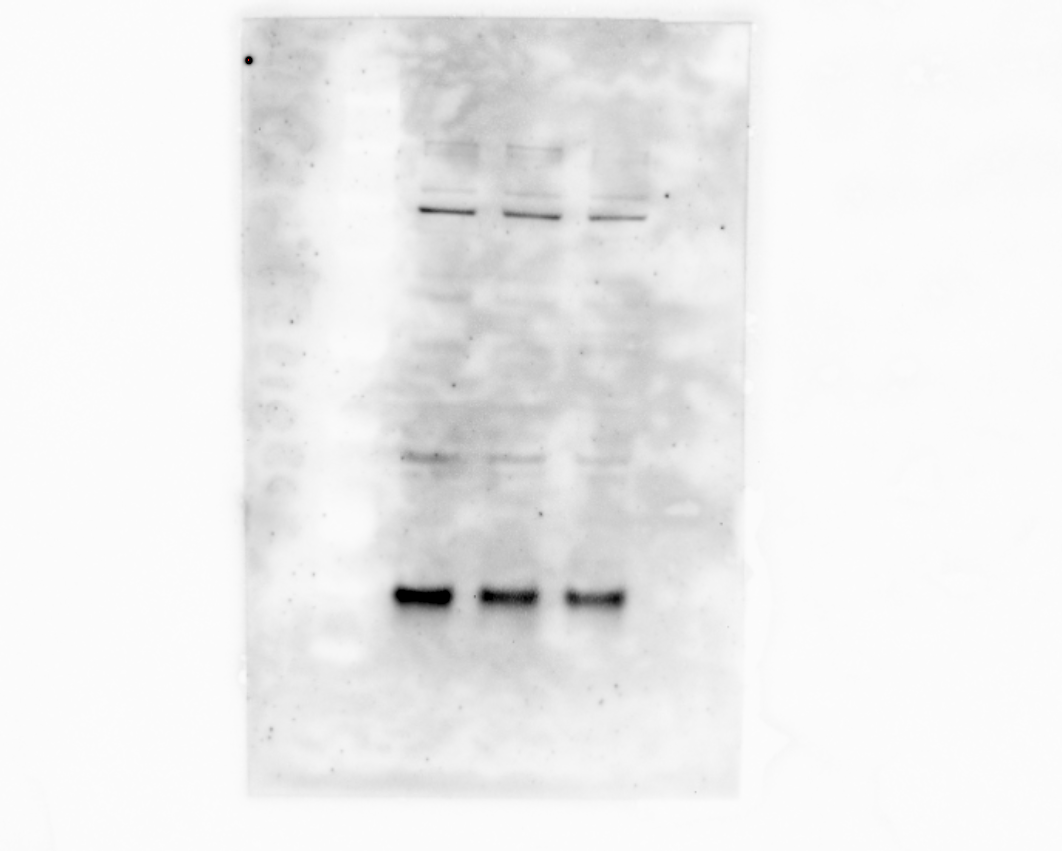

Supplement: Figure 7—figure supplement 2—source data 2. [file elife-102681-fig7-figsupp2-data2.zip › Figure 7-figure supplement 2-source_data_2/zheng lab 2020-10-23 13h49m41s.tif]

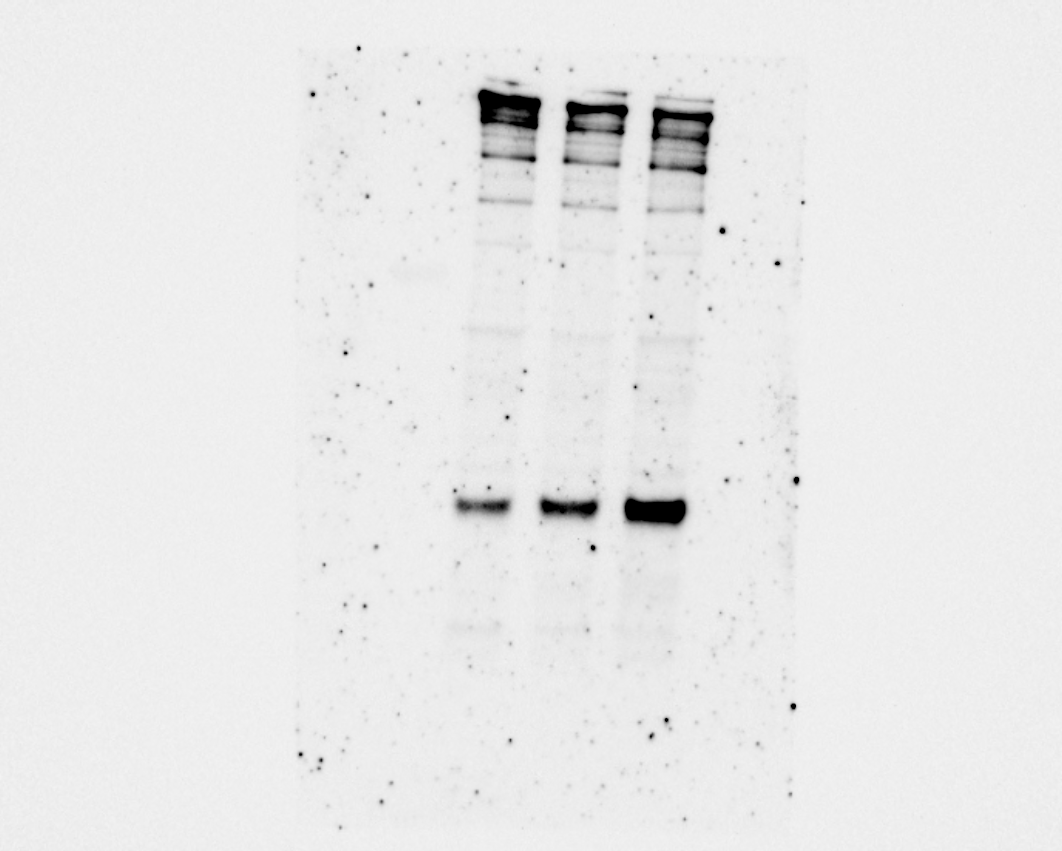

Supplement: Figure 7—figure supplement 2—source data 2. [file elife-102681-fig7-figsupp2-data2.zip › Figure 7-figure supplement 2-source_data_2/zheng lab 2020-10-27 16h02m58s.tif]

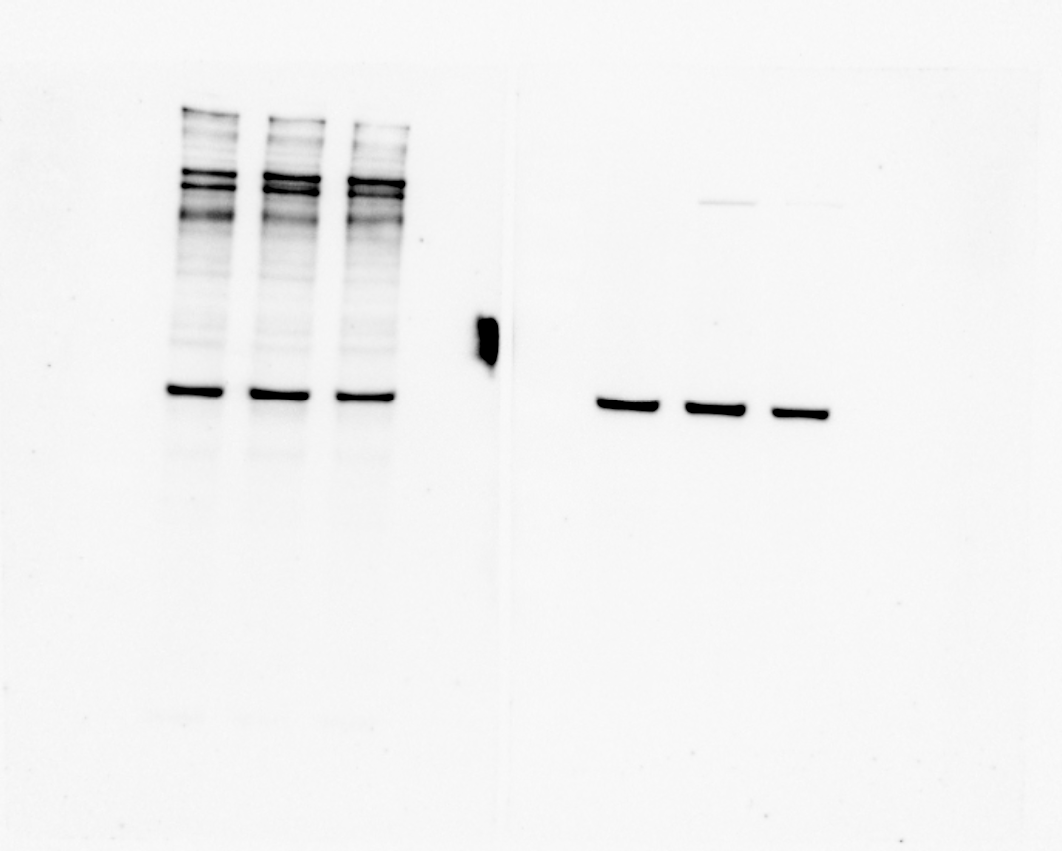

Supplement: Figure 7—figure supplement 2—source data 2. [file elife-102681-fig7-figsupp2-data2.zip › Figure 7-figure supplement 2-source_data_2/zheng lab 2020-10-28 13h11m21s.tif]

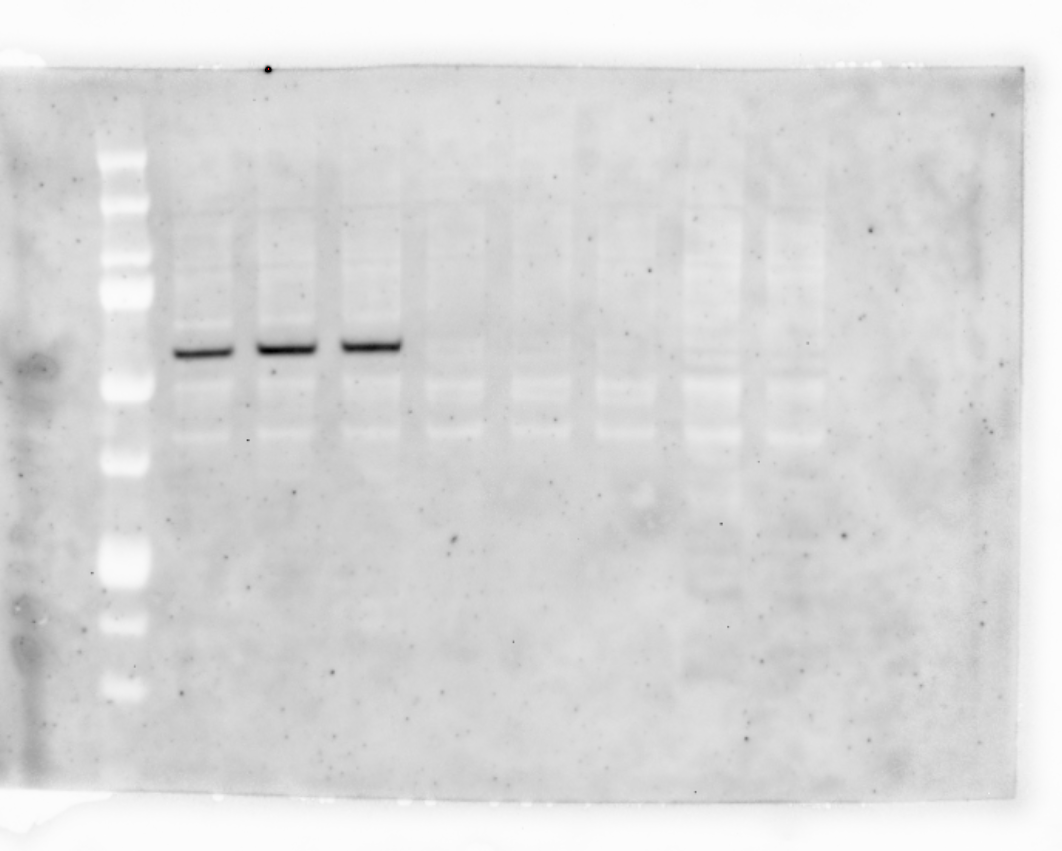

Supplement: Figure 7—figure supplement 2—source data 2. [file elife-102681-fig7-figsupp2-data2.zip › Figure 7-figure supplement 2-source_data_2/zheng lab 2020-11-18 12h48m08s.tif]

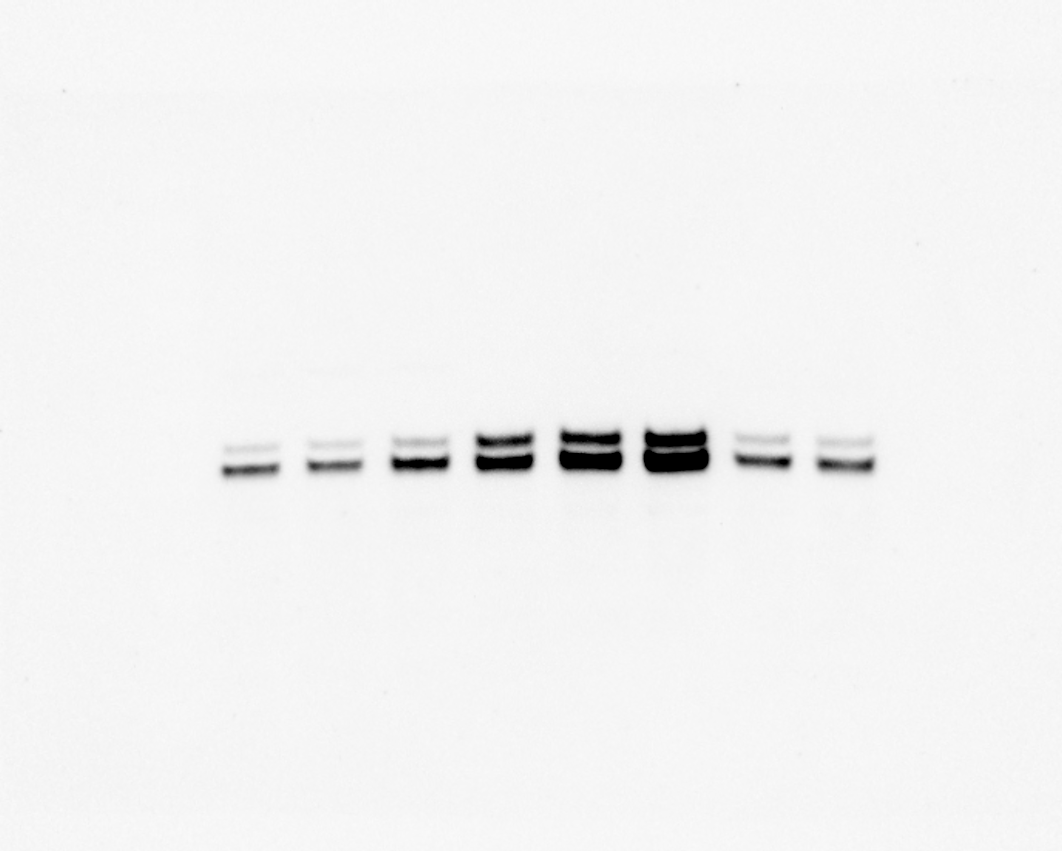

Supplement: Figure 7—figure supplement 2—source data 2. [file elife-102681-fig7-figsupp2-data2.zip › Figure 7-figure supplement 2-source_data_2/zheng lab 2020-11-19 13h30m47s.tif]

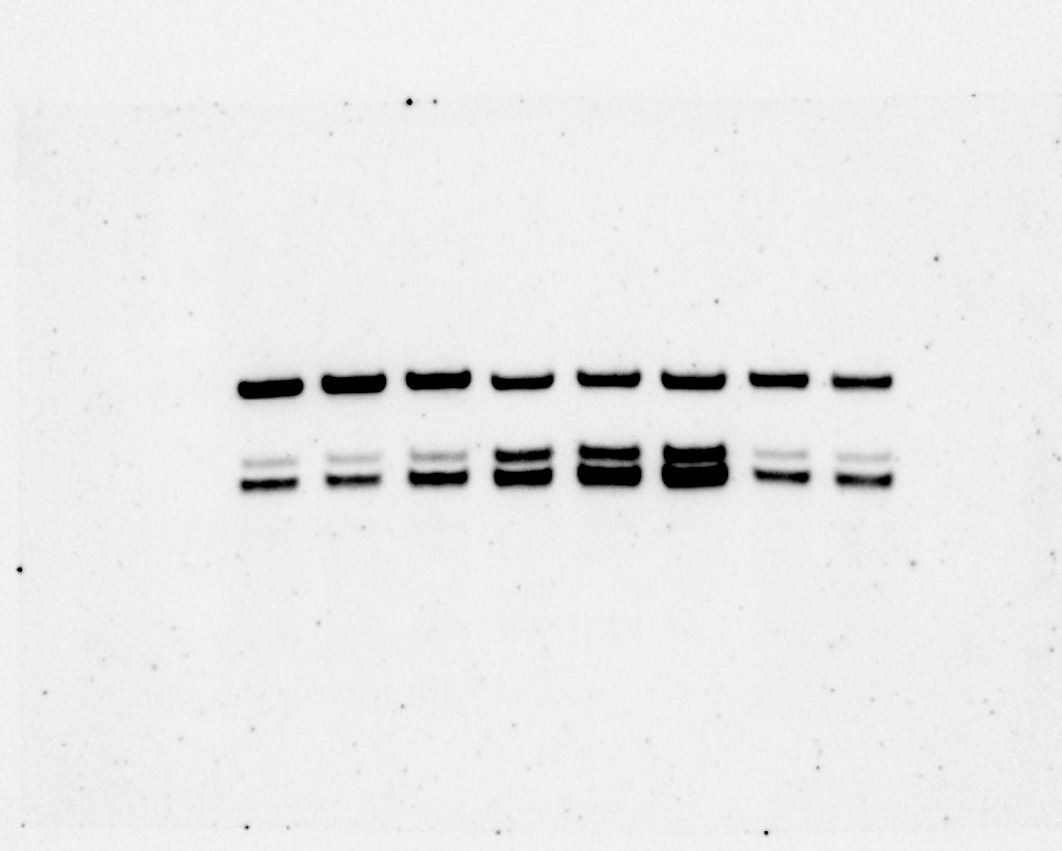

Supplement: Figure 7—figure supplement 2—source data 2. [file elife-102681-fig7-figsupp2-data2.zip › Figure 7-figure supplement 2-source_data_2/zheng lab 2020-11-20 12h34m47s.tif]

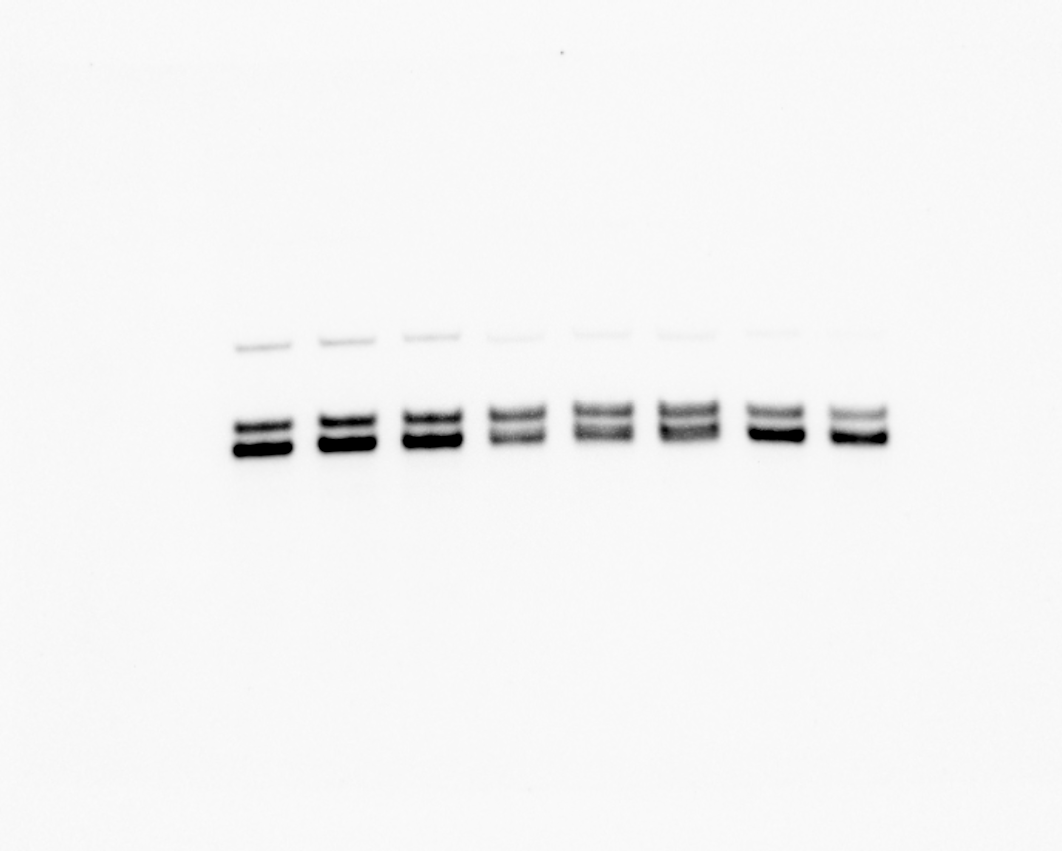

Supplement: Figure 7—figure supplement 2—source data 2. [file elife-102681-fig7-figsupp2-data2.zip › Figure 7-figure supplement 2-source_data_2/zheng lab 2020-11-24 14h47m12s.tif]

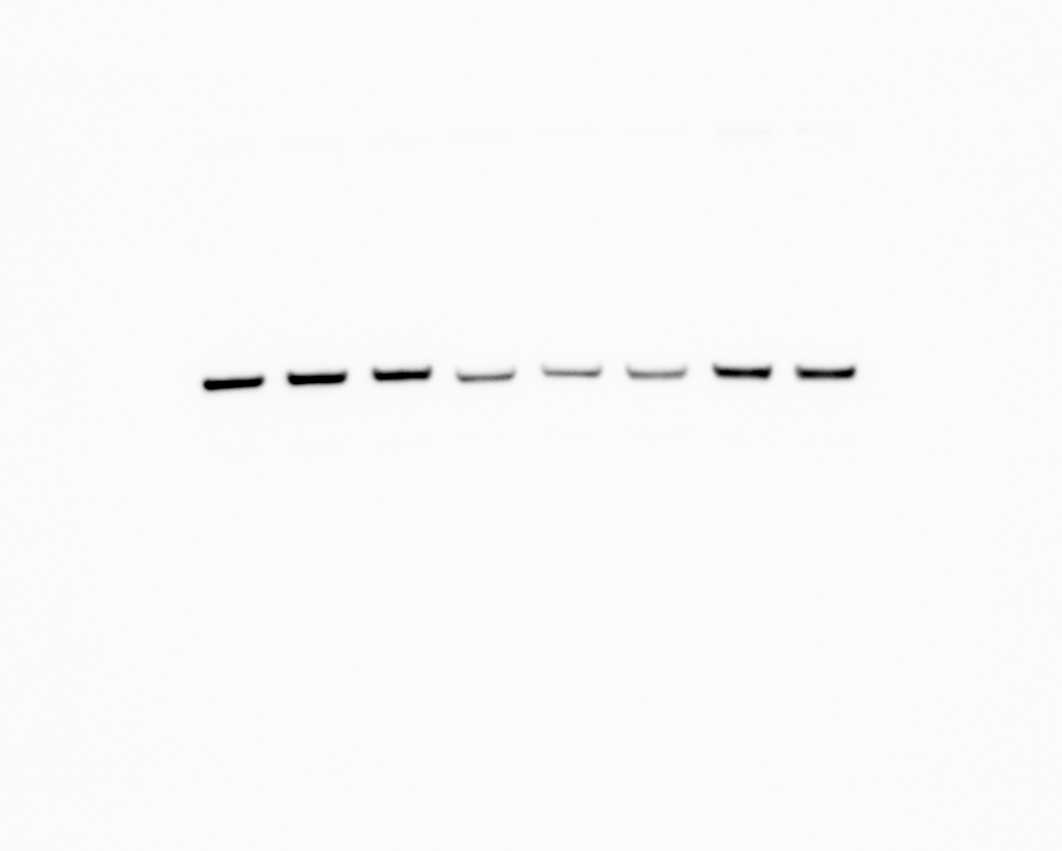

Supplement: Figure 7—figure supplement 2—source data 2. [file elife-102681-fig7-figsupp2-data2.zip › Figure 7-figure supplement 2-source_data_2/zheng lab 2020-11-25 13h21m09s.tif]

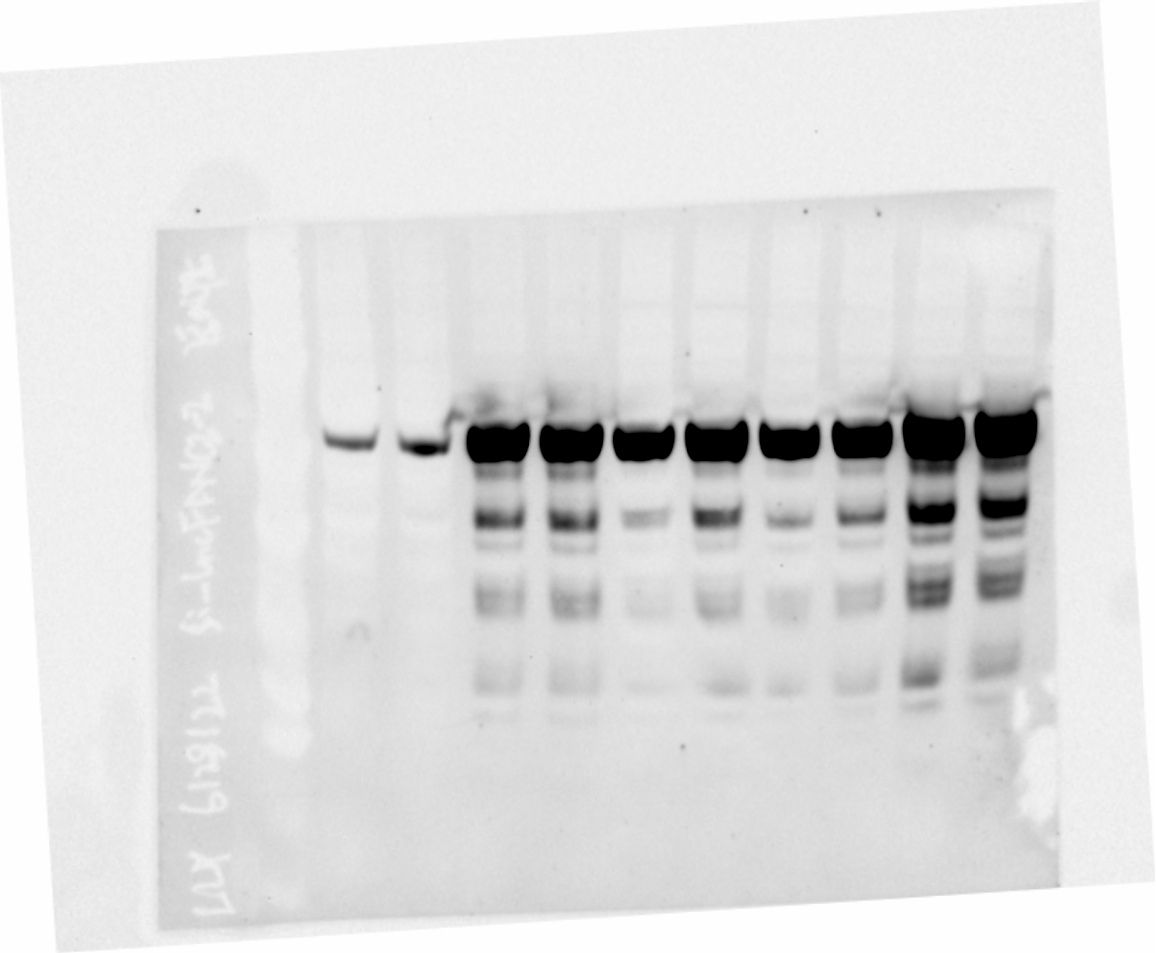

Supplement: Figure 7—figure supplement 2—source data 2. [file elife-102681-fig7-figsupp2-data2.zip › Figure 7-figure supplement 2-source_data_2/zheng lab 2022-06-28 13h36m12s.tif]

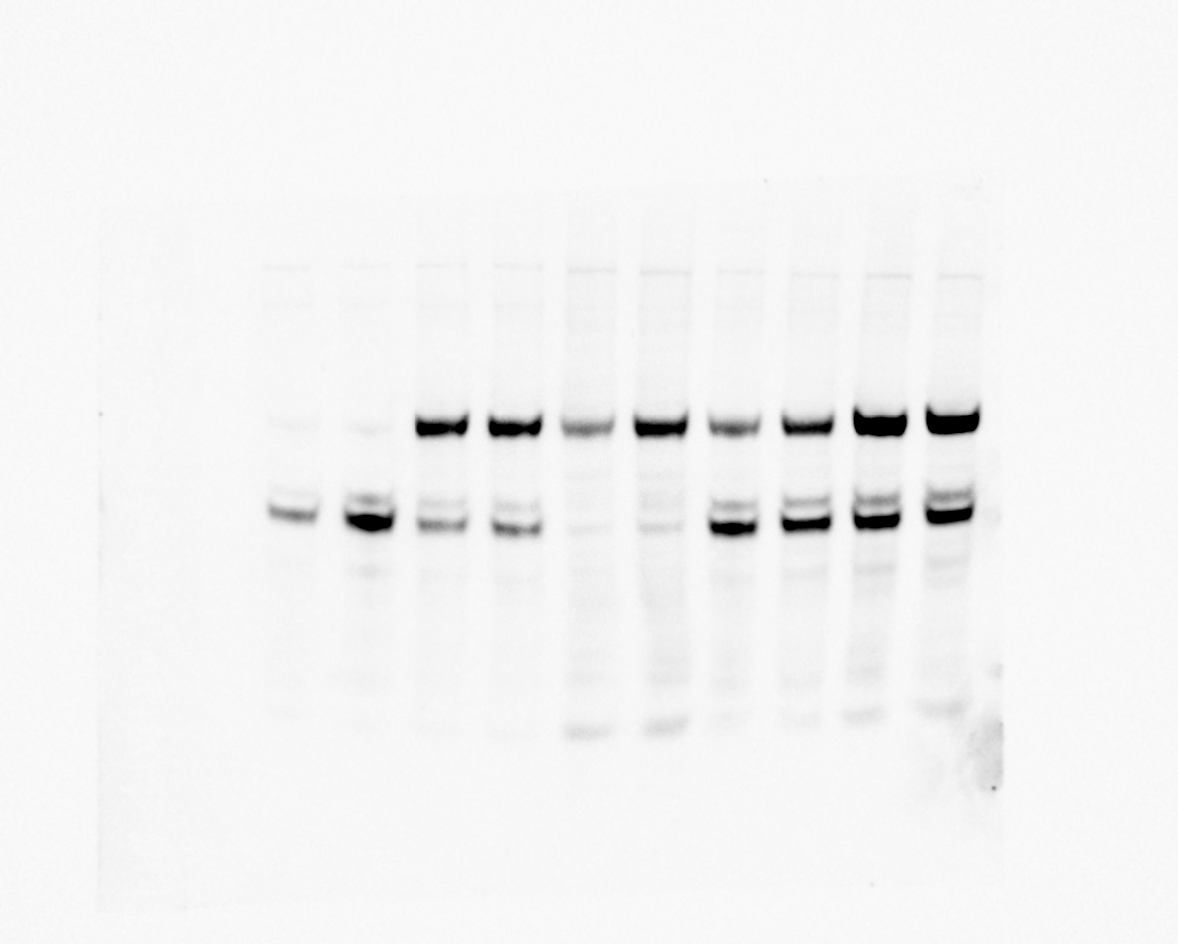

Supplement: Figure 7—figure supplement 2—source data 2. [file elife-102681-fig7-figsupp2-data2.zip › Figure 7-figure supplement 2-source_data_2/zheng lab 2022-06-29 15h11m33s.tif]

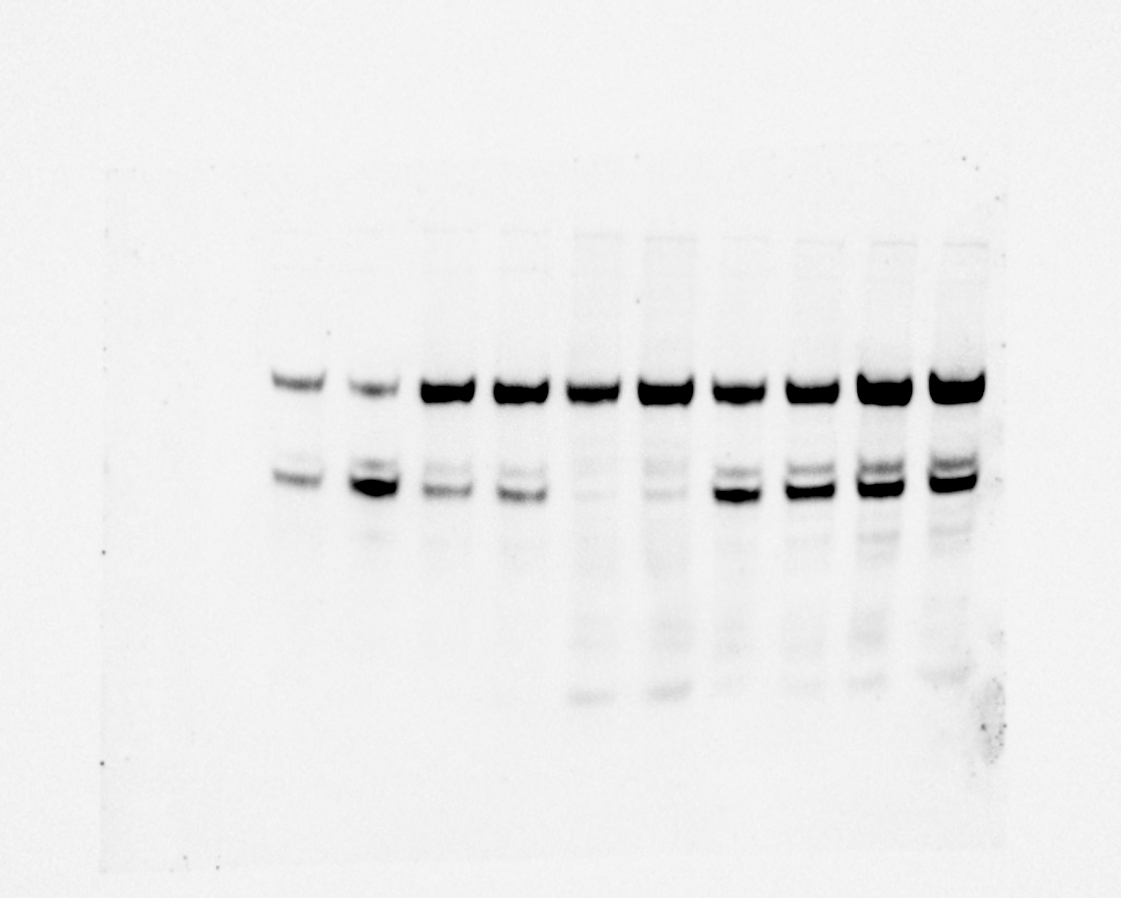

Supplement: Figure 7—figure supplement 2—source data 2. [file elife-102681-fig7-figsupp2-data2.zip › Figure 7-figure supplement 2-source_data_2/zheng lab 2022-06-30 14h13m29s.tif]

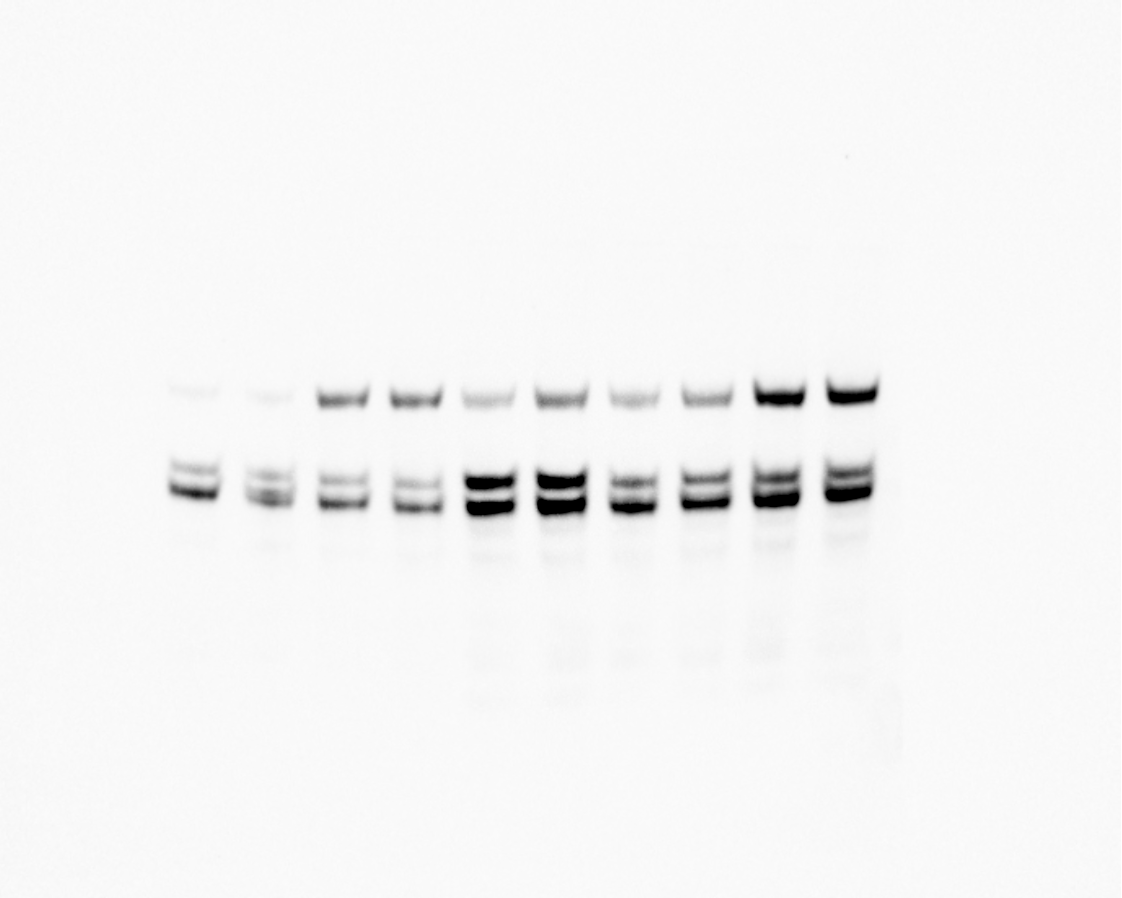

Supplement: Figure 7—figure supplement 2—source data 2. [file elife-102681-fig7-figsupp2-data2.zip › Figure 7-figure supplement 2-source_data_2/zheng lab 2022-07-01 10h20m38s.tif]

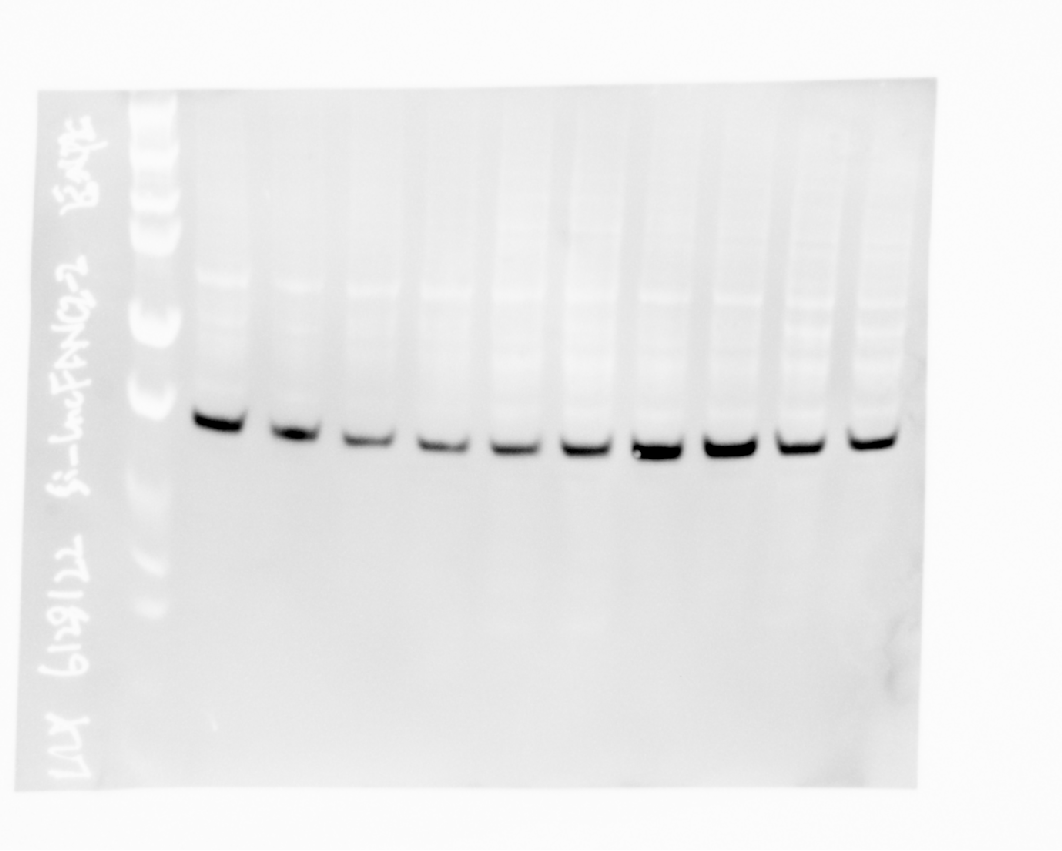

Supplement: Figure 7—figure supplement 2—source data 2. [file elife-102681-fig7-figsupp2-data2.zip › Figure 7-figure supplement 2-source_data_2/zheng lab 2022-07-05 10h44m56s.tif]

Figure 8

G

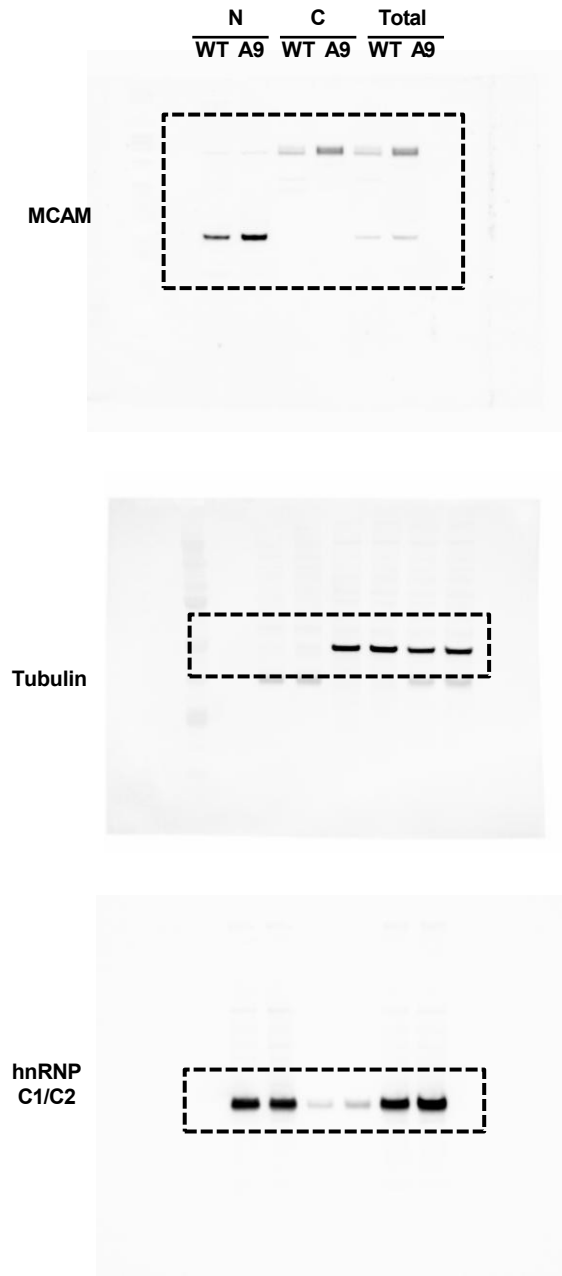

Figure 8

J

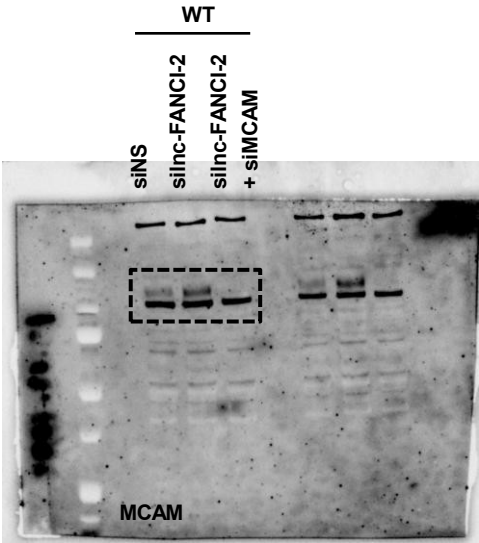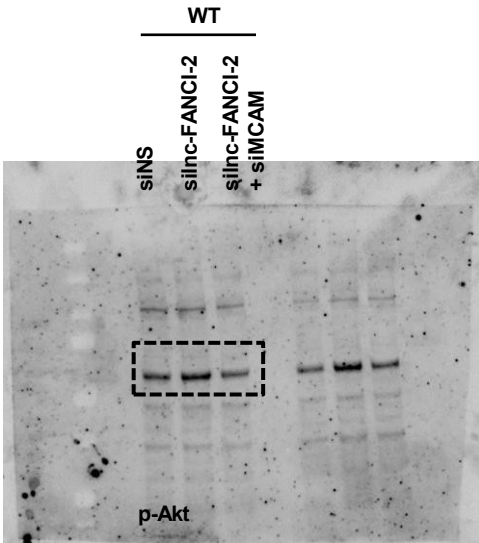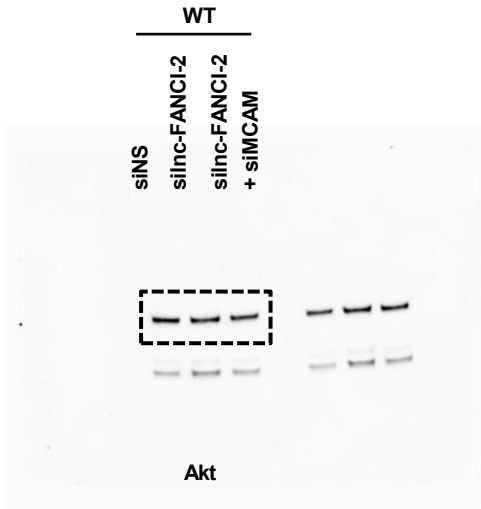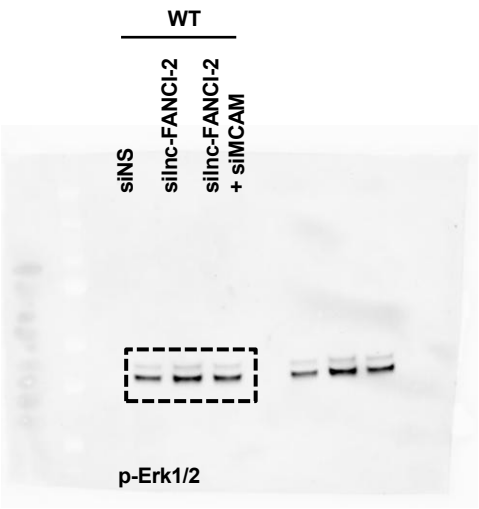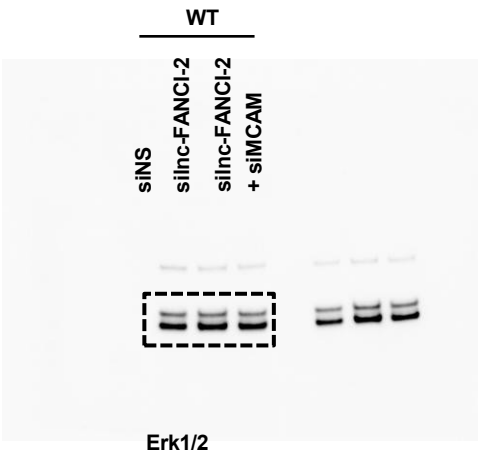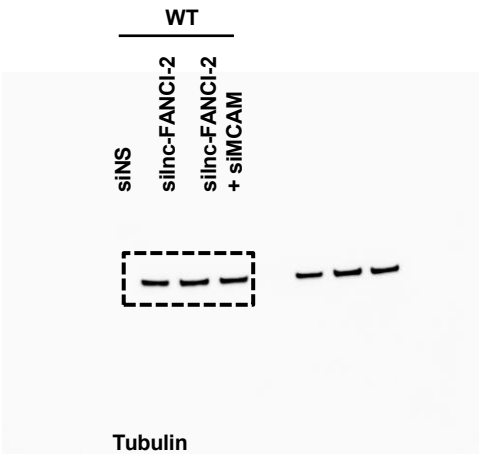

Figure 8

J

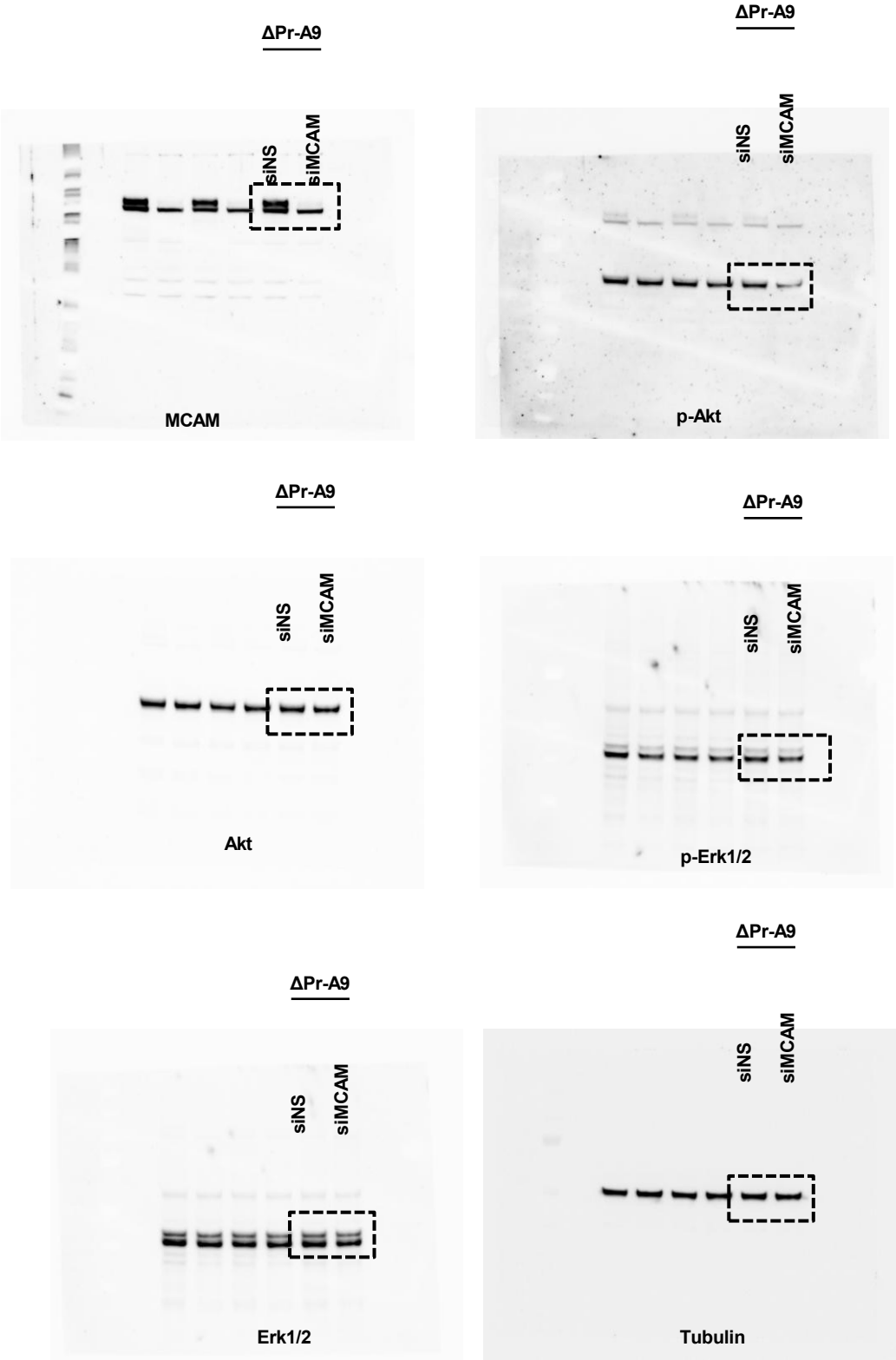

Figure 8

K

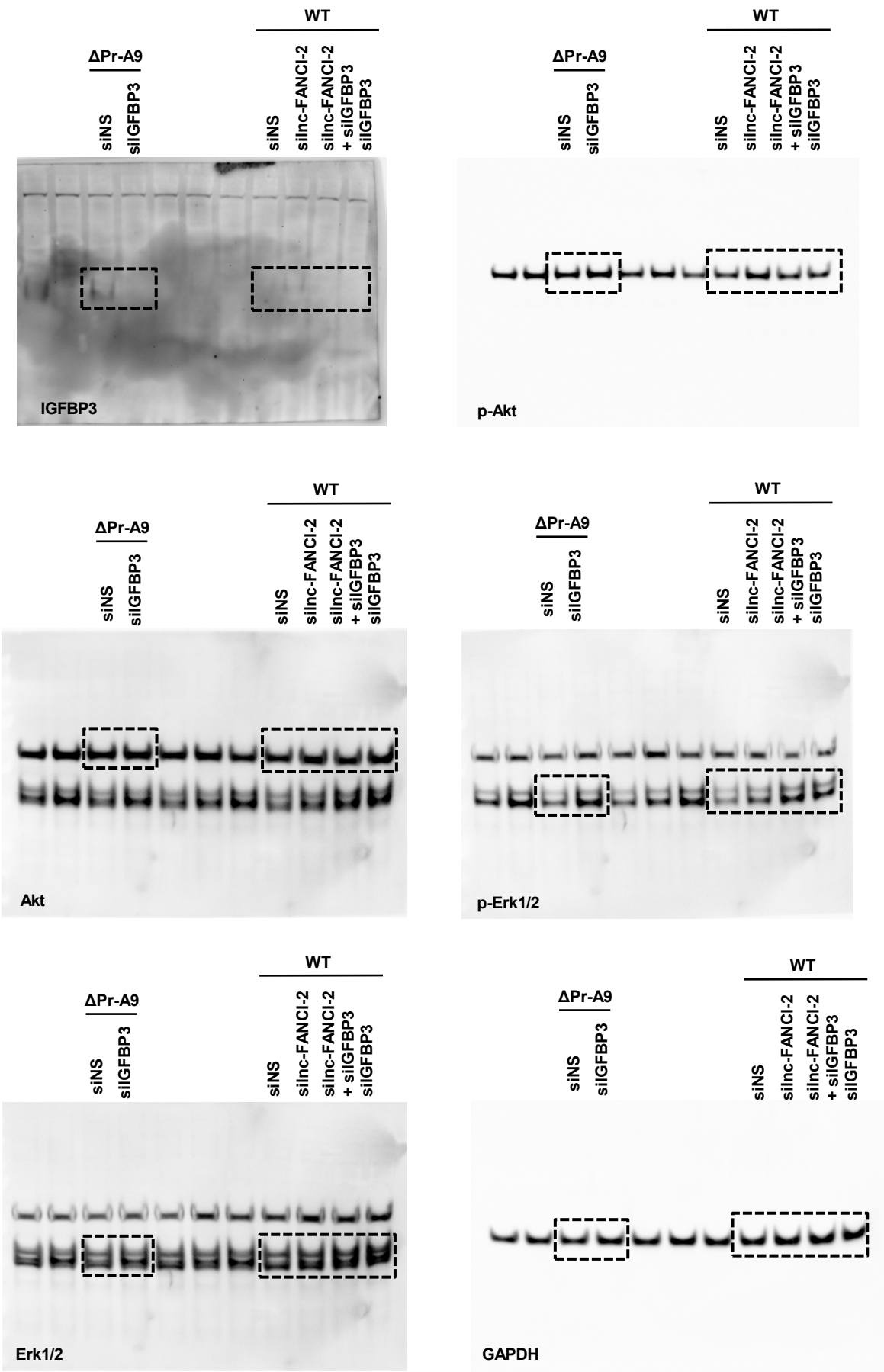

Supplement: Figure 8—source data 1. [file elife-102681-fig8-data1.pdf]

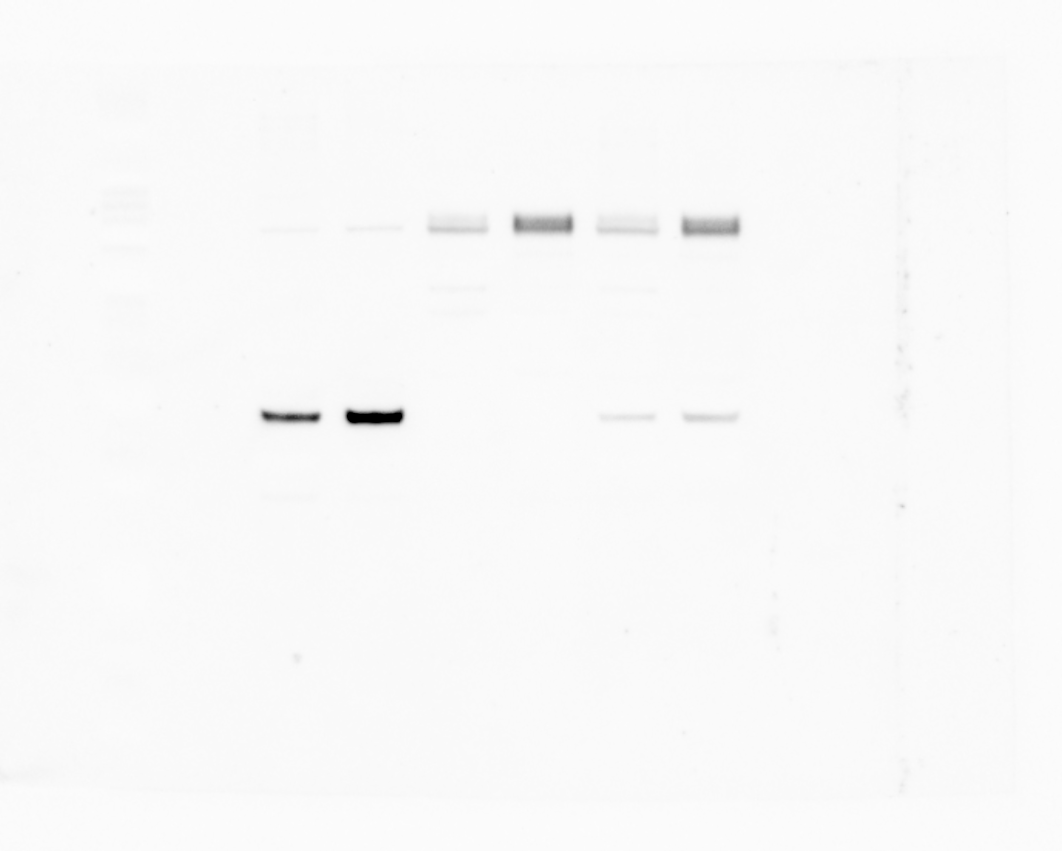

Supplement: Figure 8—source data 2. [file elife-102681-fig8-data2.zip › Figure 8-source data_2/zheng lab 2020-03-04 11h59m44s.tif]

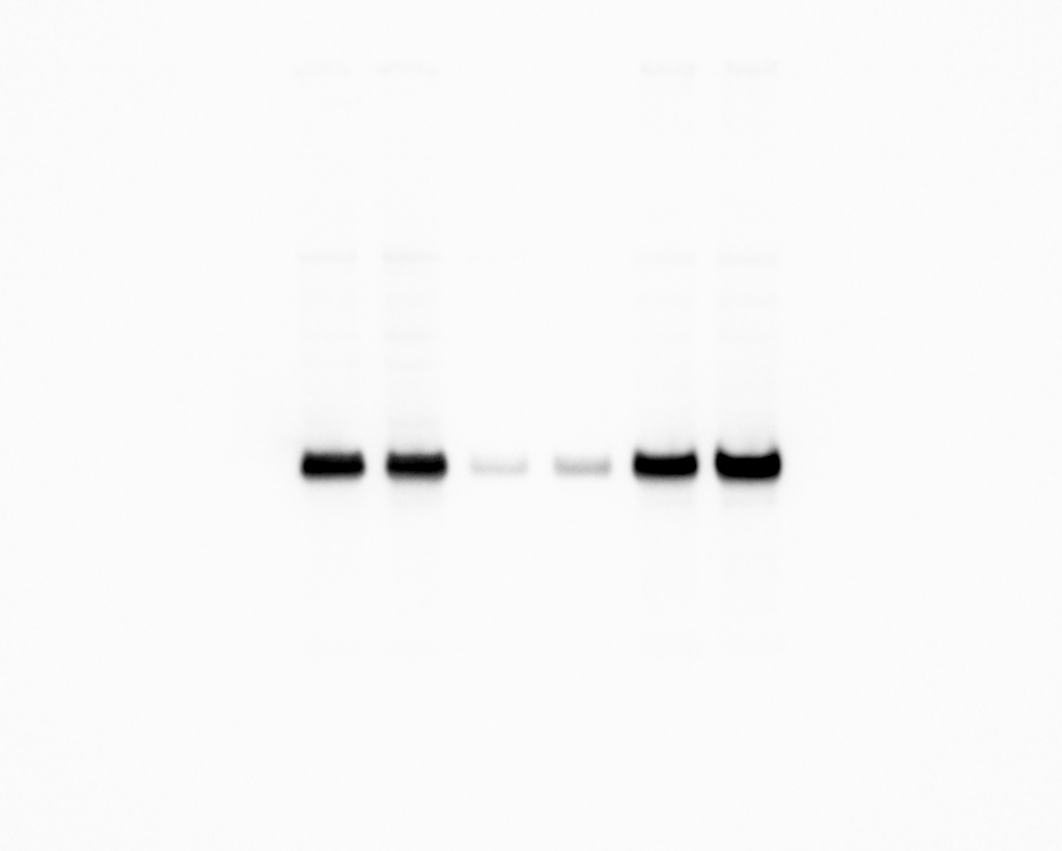

Supplement: Figure 8—source data 2. [file elife-102681-fig8-data2.zip › Figure 8-source data_2/zheng lab 2020-03-10 17h43m27s.tif]

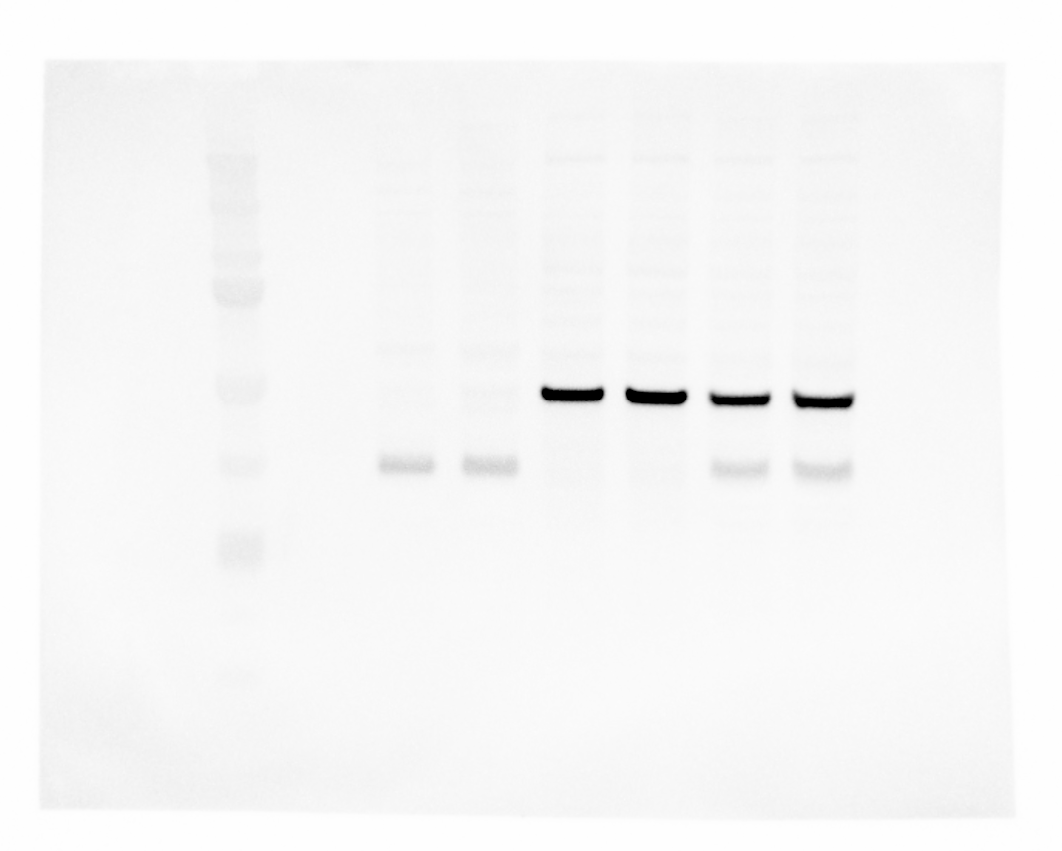

Supplement: Figure 8—source data 2. [file elife-102681-fig8-data2.zip › Figure 8-source data_2/zheng lab 2020-03-12 11h07m32s.tif]

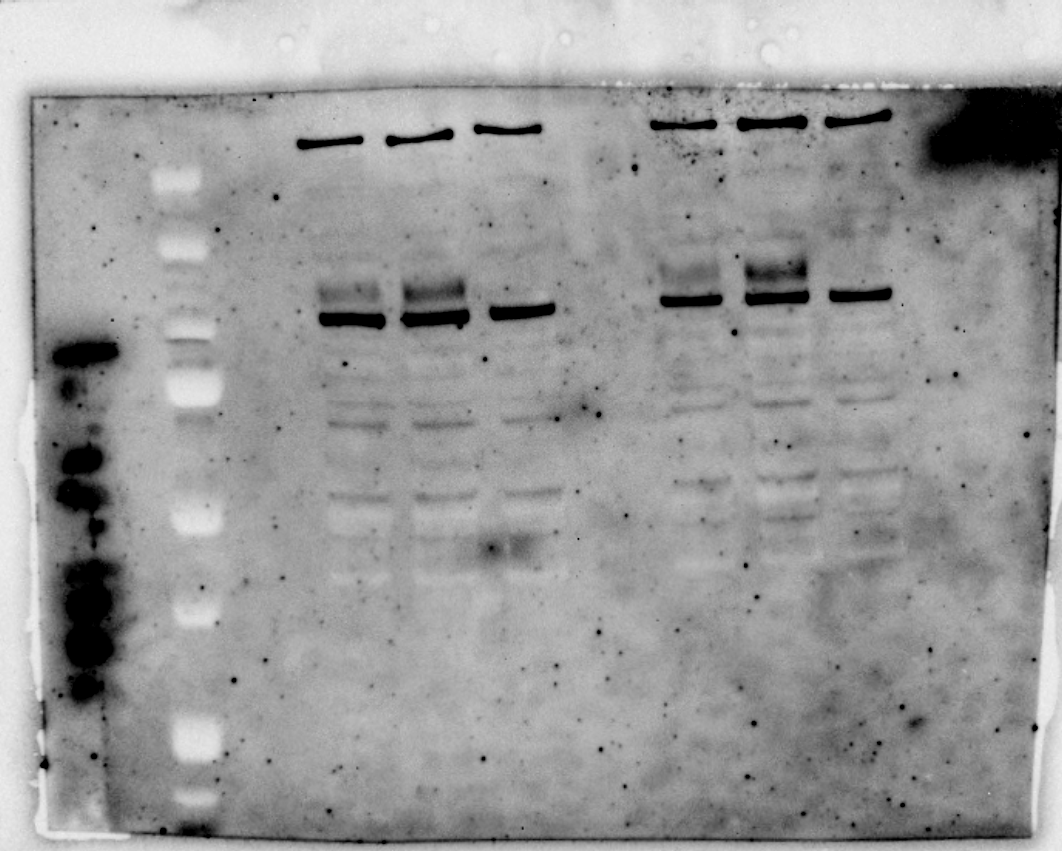

Supplement: Figure 8—source data 2. [file elife-102681-fig8-data2.zip › Figure 8-source data_2/zheng lab 2021-02-02 15h33m58s.tif]

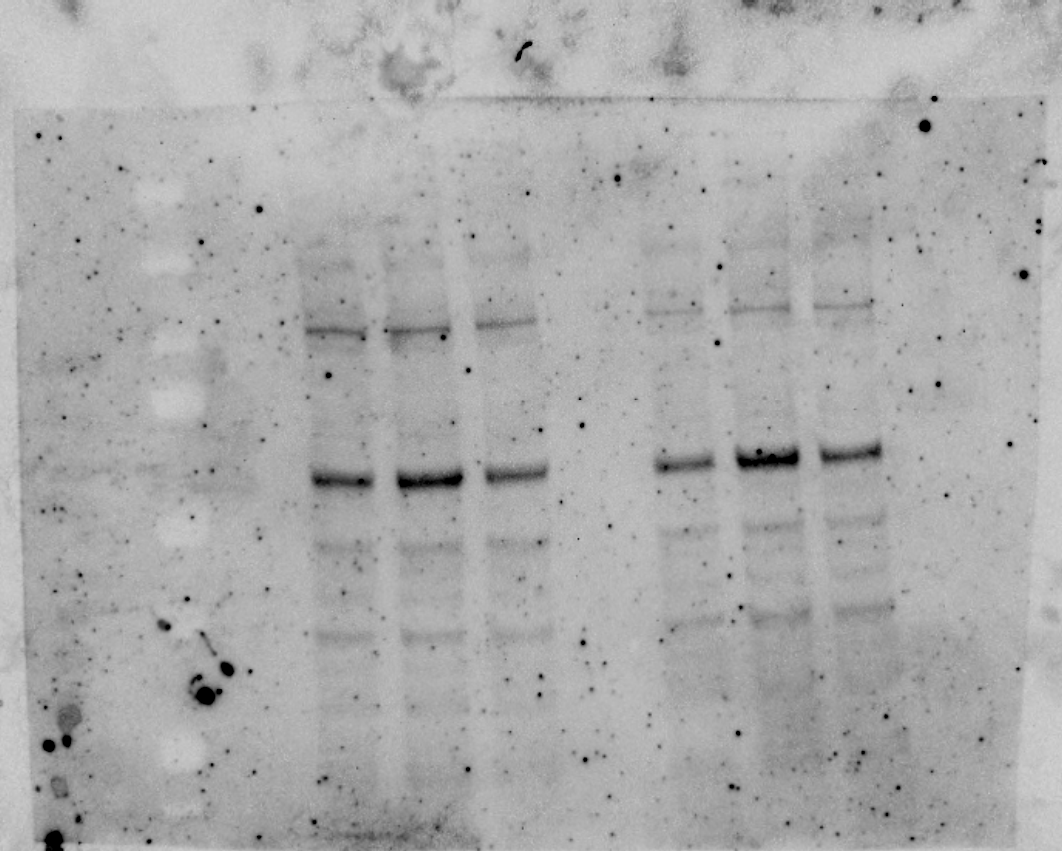

Supplement: Figure 8—source data 2. [file elife-102681-fig8-data2.zip › Figure 8-source data_2/zheng lab 2021-02-03 13h20m58s.tif]

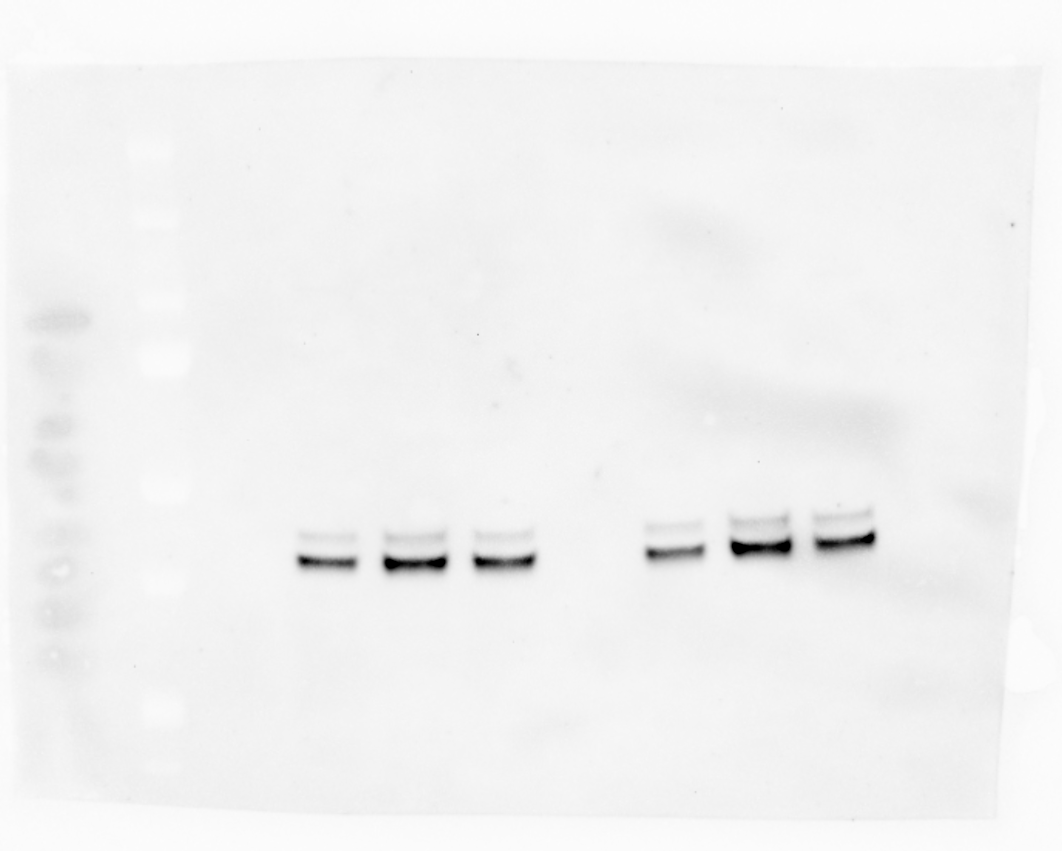

Supplement: Figure 8—source data 2. [file elife-102681-fig8-data2.zip › Figure 8-source data_2/zheng lab 2021-02-04 11h48m32s.tif]
