# Supplementary material for: The long noncoding RNA lnc-FANCI-2 intrinsically restricts RAS signaling in human papillomavirus type 16-infected cervical cancer cells
Source: eLife. 2025 Aug 29;13:RP102681. doi: 10.7554/eLife.102681 (PMC12396819; doi:10.7554/eLife.102681)
Supplement: Figure 7—source data 1. [file elife-102681-fig7-data1.pdf]

Figure 7

B

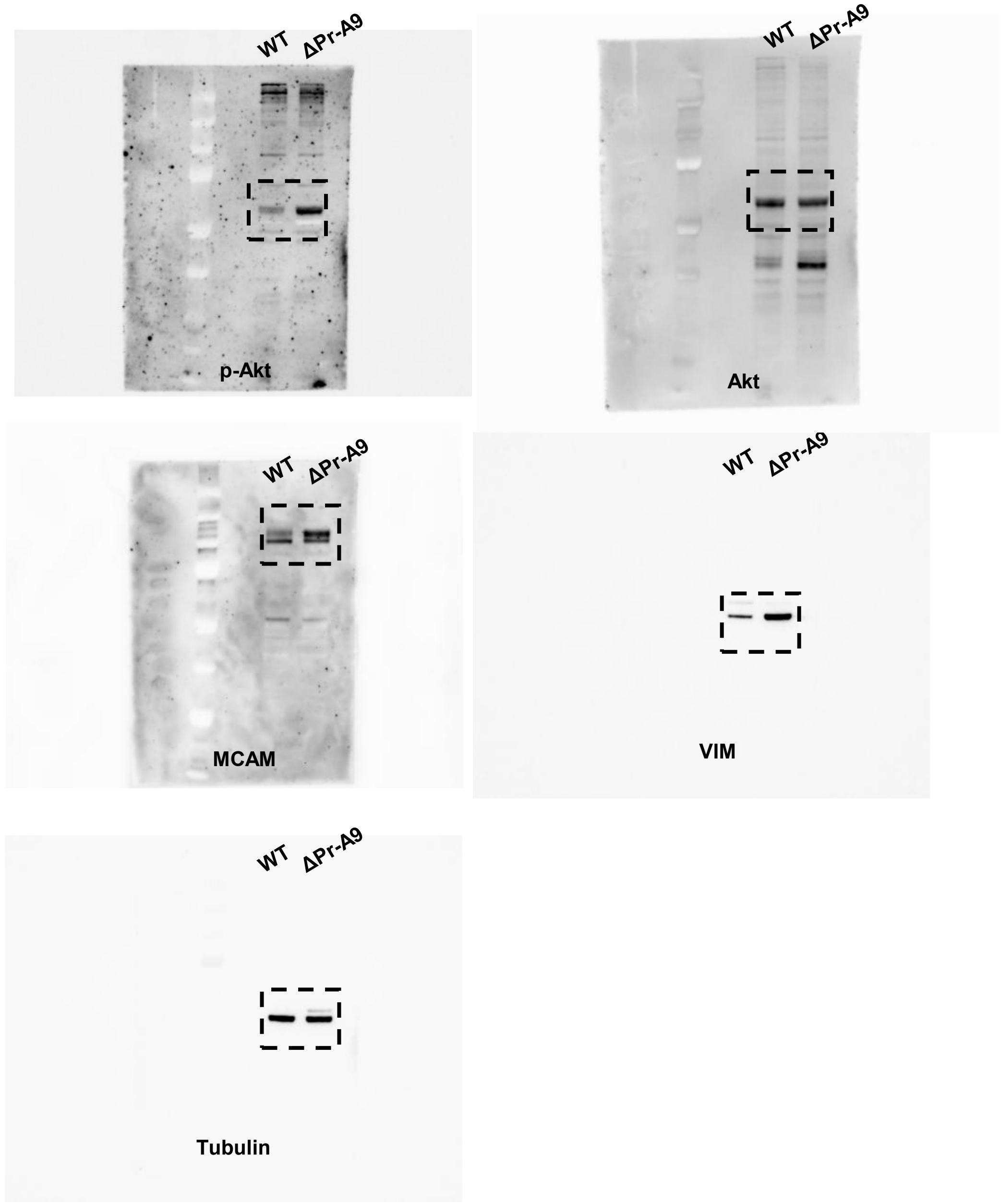
WT
ΔPr-A9
p-Akt
WT
ΔPr-A9
Akt
WT
ΔPr-A9
MCAM
WT
ΔPr-A9
VIM
WT
ΔPr-A9
Tubulin

Figure 7

B

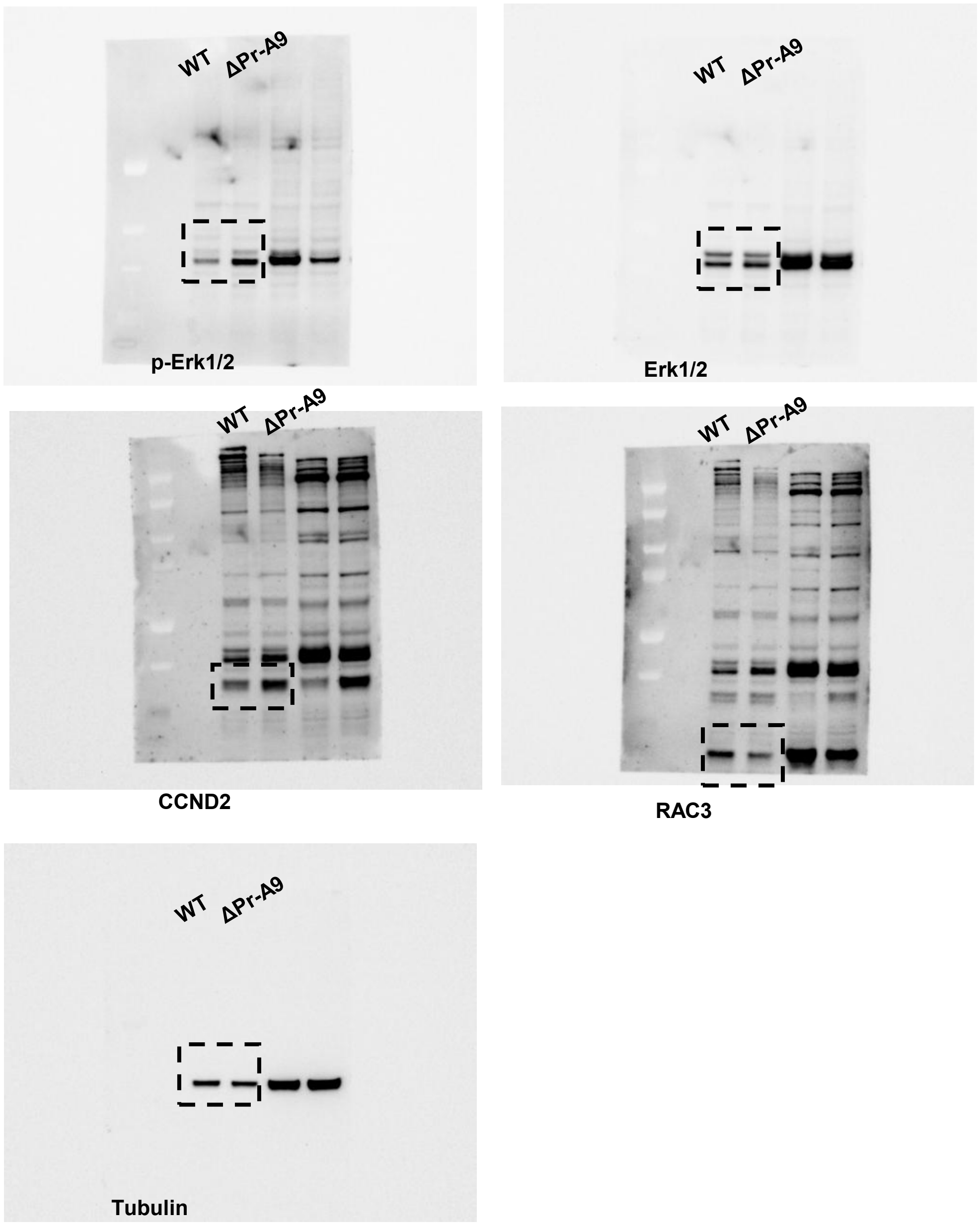

Figure 7

C

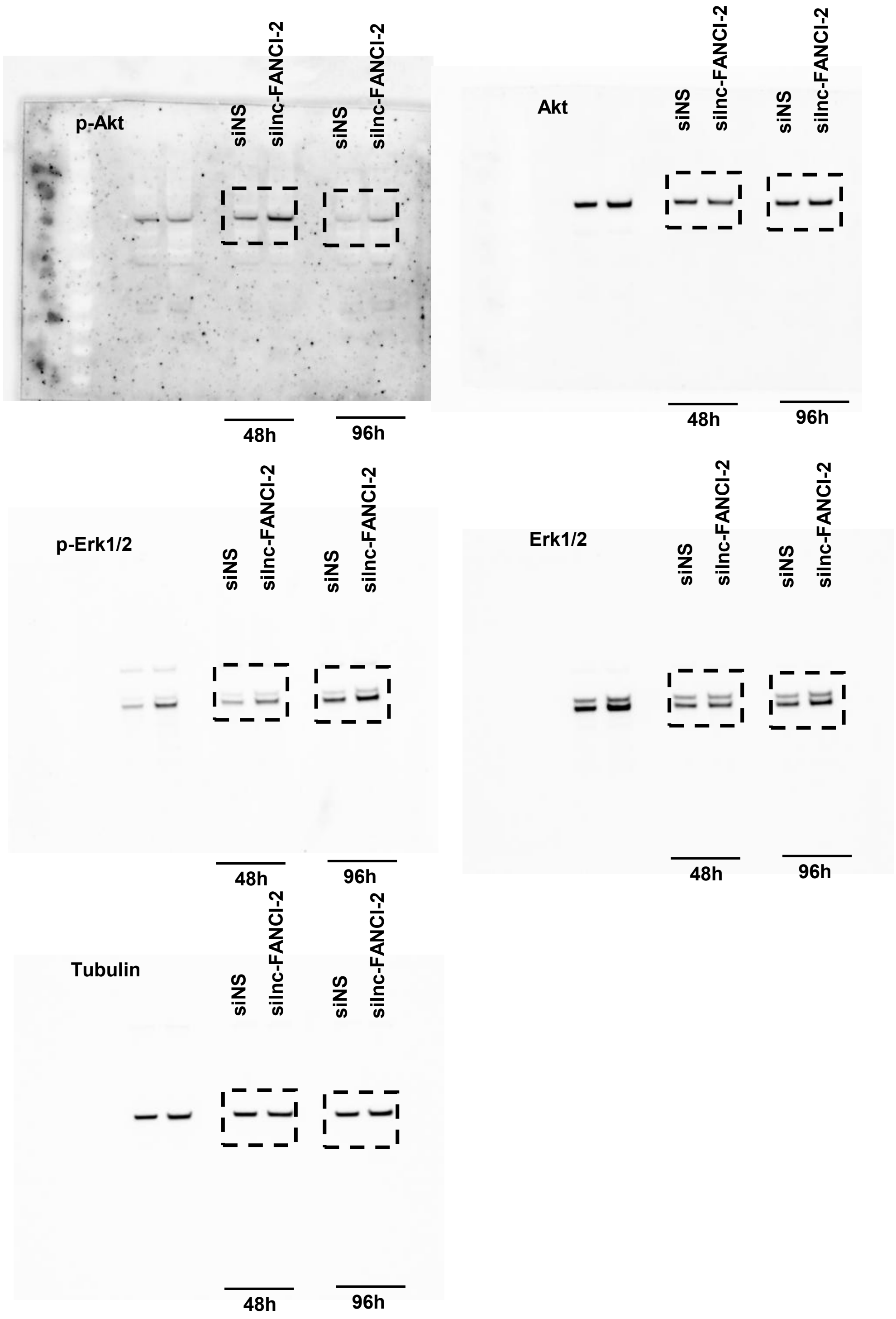
p-Akt
siNS
silnc-FANCI-2
siNS
silnc-FANCI-2
48h
96h
Akt
siNS
silnc-FANCI-2
siNS
silnc-FANCI-2
48h
96h
p-Erk1/2
siNS
silnc-FANCI-2
siNS
silnc-FANCI-2
48h
96h
Erk1/2
siNS
silnc-FANCI-2
siNS
silnc-FANCI-2
48h
96h
Tubulin
siNS
silnc-FANCI-2
siNS
silnc-FANCI-2
48h
96h

Figure 7

C

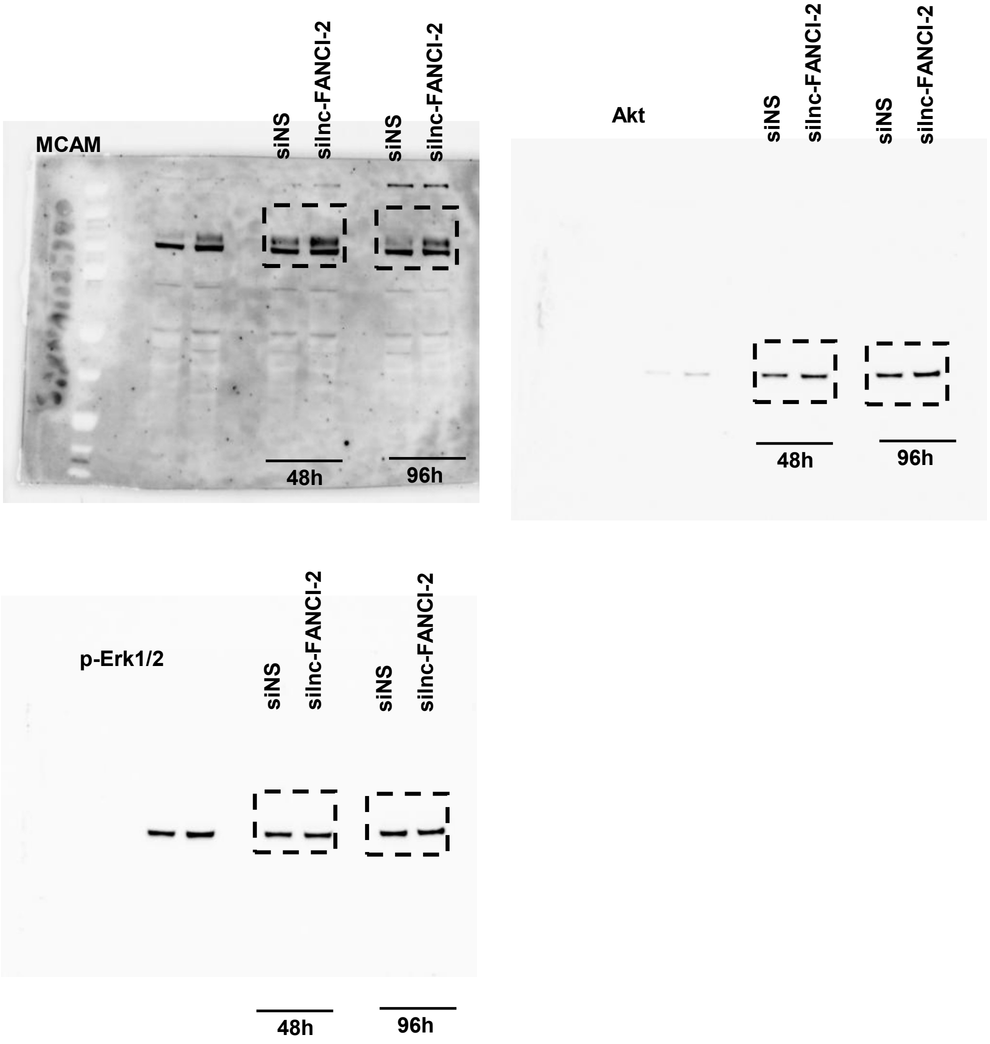
MCAM
siNS
silnc-FANCI-2
siNS
silnc-FANCI-2
48h
96h
Akt
siNS
silnc-FANCI-2
siNS
silnc-FANCI-2
48h
96h
p-Erk1/2
siNS
silnc-FANCI-2
siNS
silnc-FANCI-2
48h
96h

Figure 7

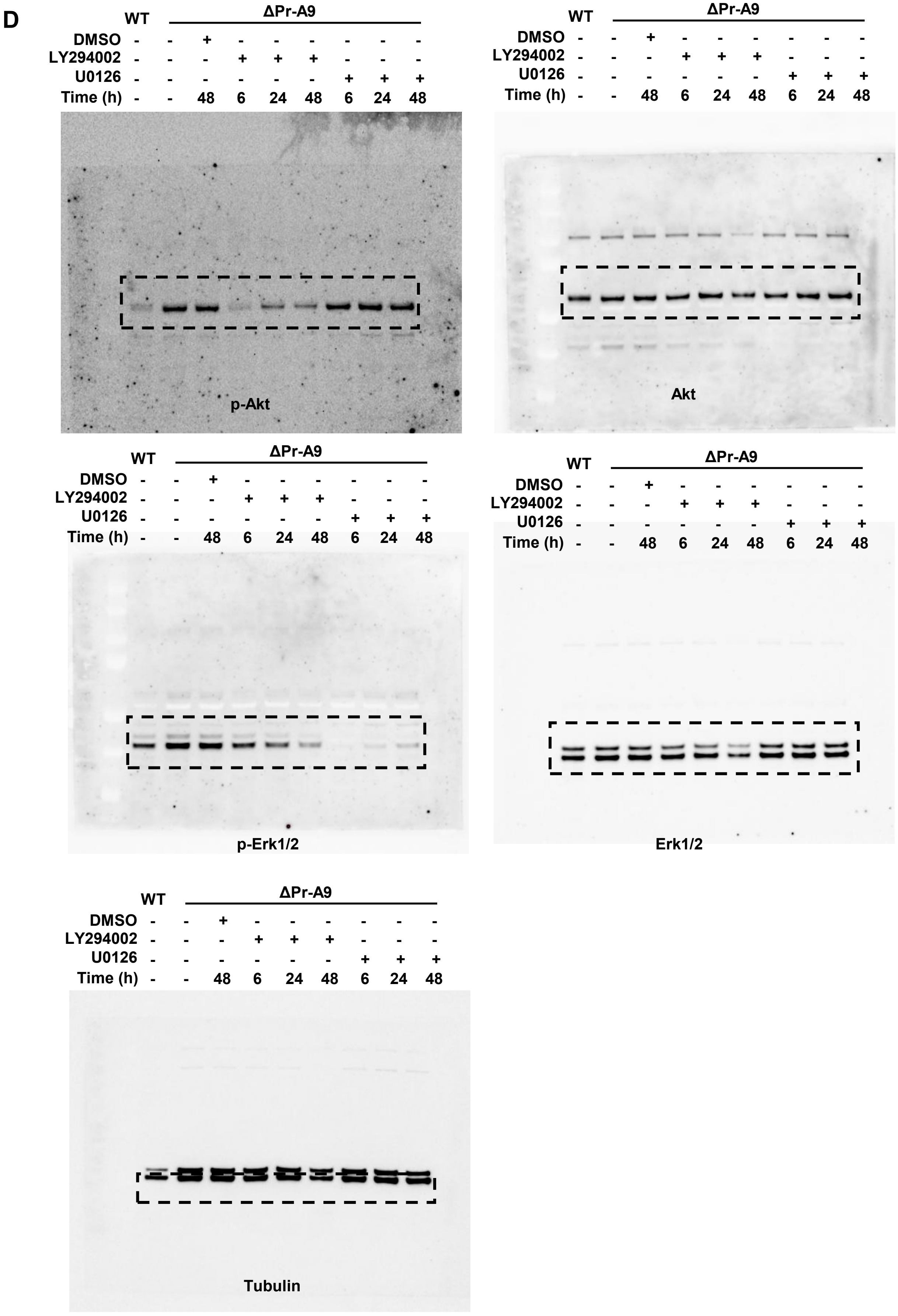
D
WT
ΔPr-A9
DMSO - - + - - - - - -
LY294002 - - - + + + - - -
U0126 - - - - - - + + +
Time (h) - - 48 6 24 48 6 24 48
p-Akt
Akt
p-Erk1/2
Erk1/2
Tubulin

Figure 7

D

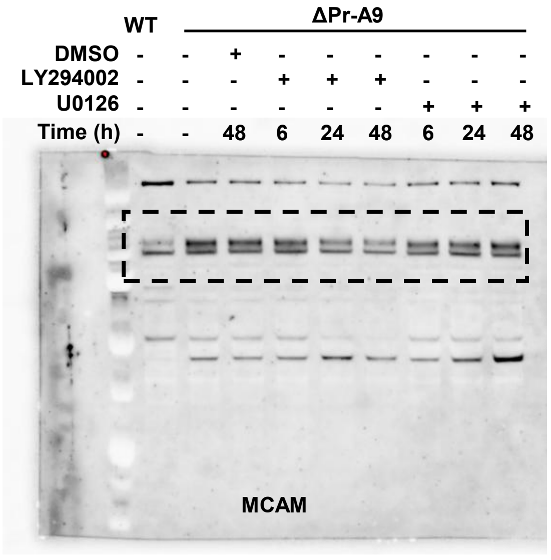
WT
ΔPr-A9
DMSO - - + - - - - - -
LY294002 - - - + + + - - -
U0126 - - - - - - + + +
Time (h) - - 48 6 24 48 6 24 48
MCAM

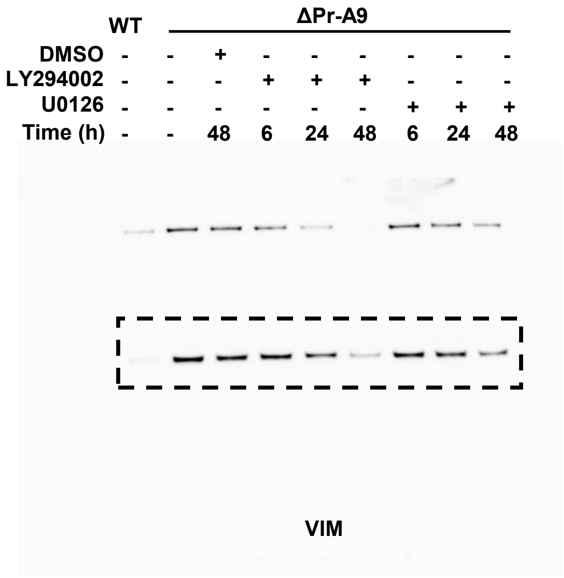
WT
ΔPr-A9
DMSO - - + - - - - - -
LY294002 - - - + + + - - -
U0126 - - - - - - + + +
Time (h) - - 48 6 24 48 6 24 48
VIM

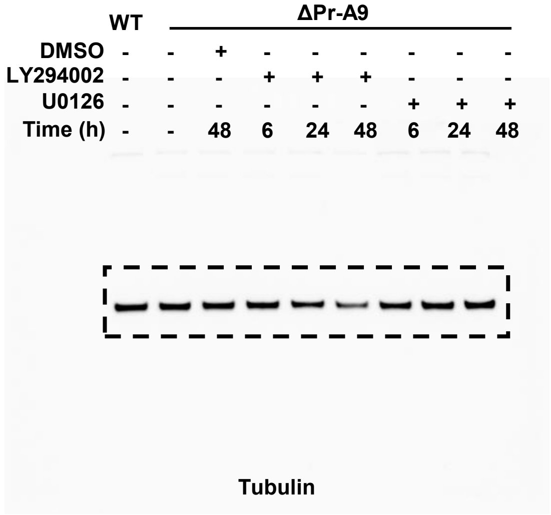
WT
ΔPr-A9
DMSO - - + - - - - - -
LY294002 - - - + + + - - -
U0126 - - - - - - + + +
Time (h) - - 48 6 24 48 6 24 48
Tubulin
