# Supplementary material for: The long noncoding RNA lnc-FANCI-2 intrinsically restricts RAS signaling in human papillomavirus type 16-infected cervical cancer cells
Source: eLife. 2025 Aug 29;13:RP102681. doi: 10.7554/eLife.102681 (PMC12396819; doi:10.7554/eLife.102681)

**Figure 7-figure supplement 2**

**A**

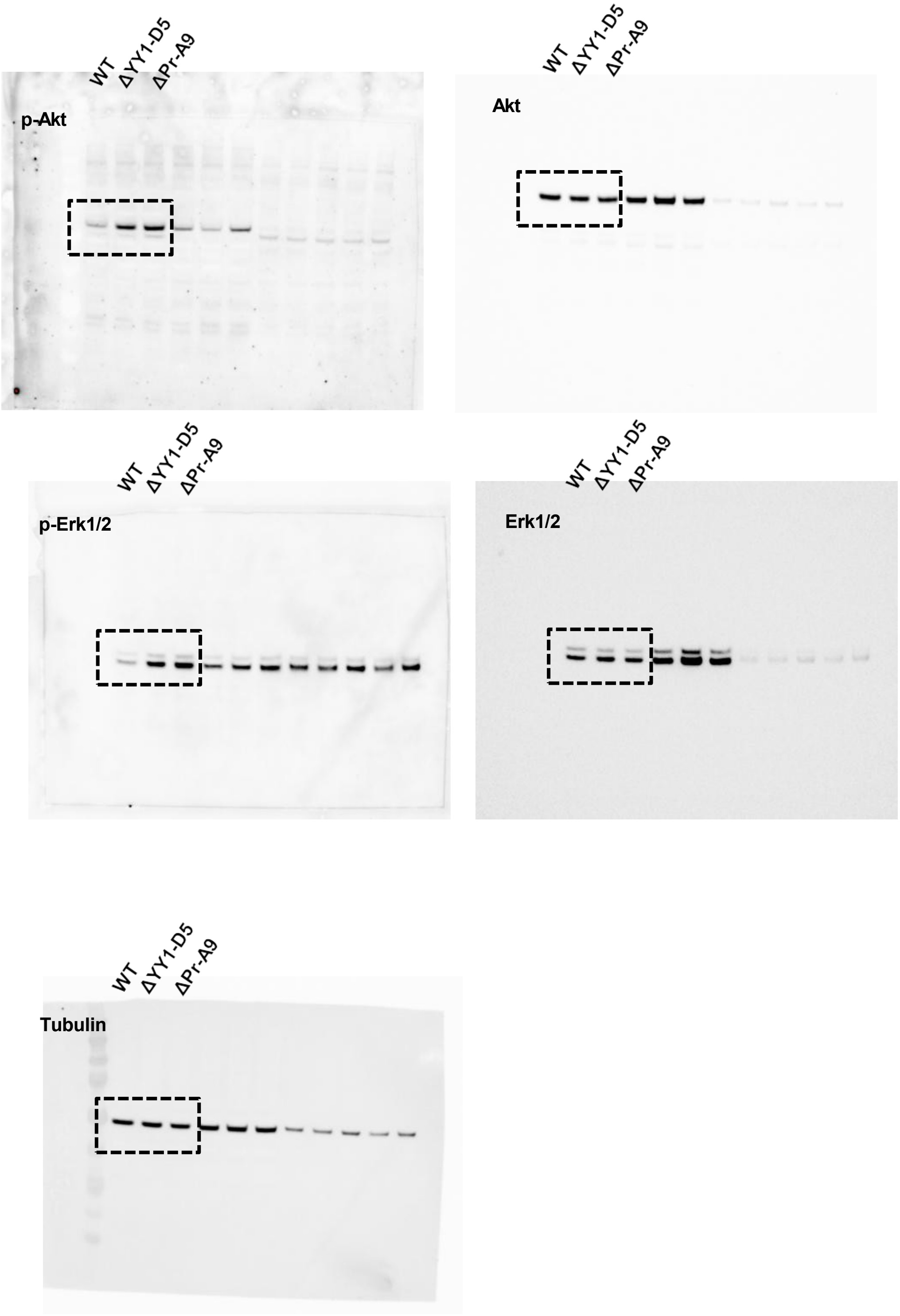

**Figure 7-figure supplement 2**

**A**

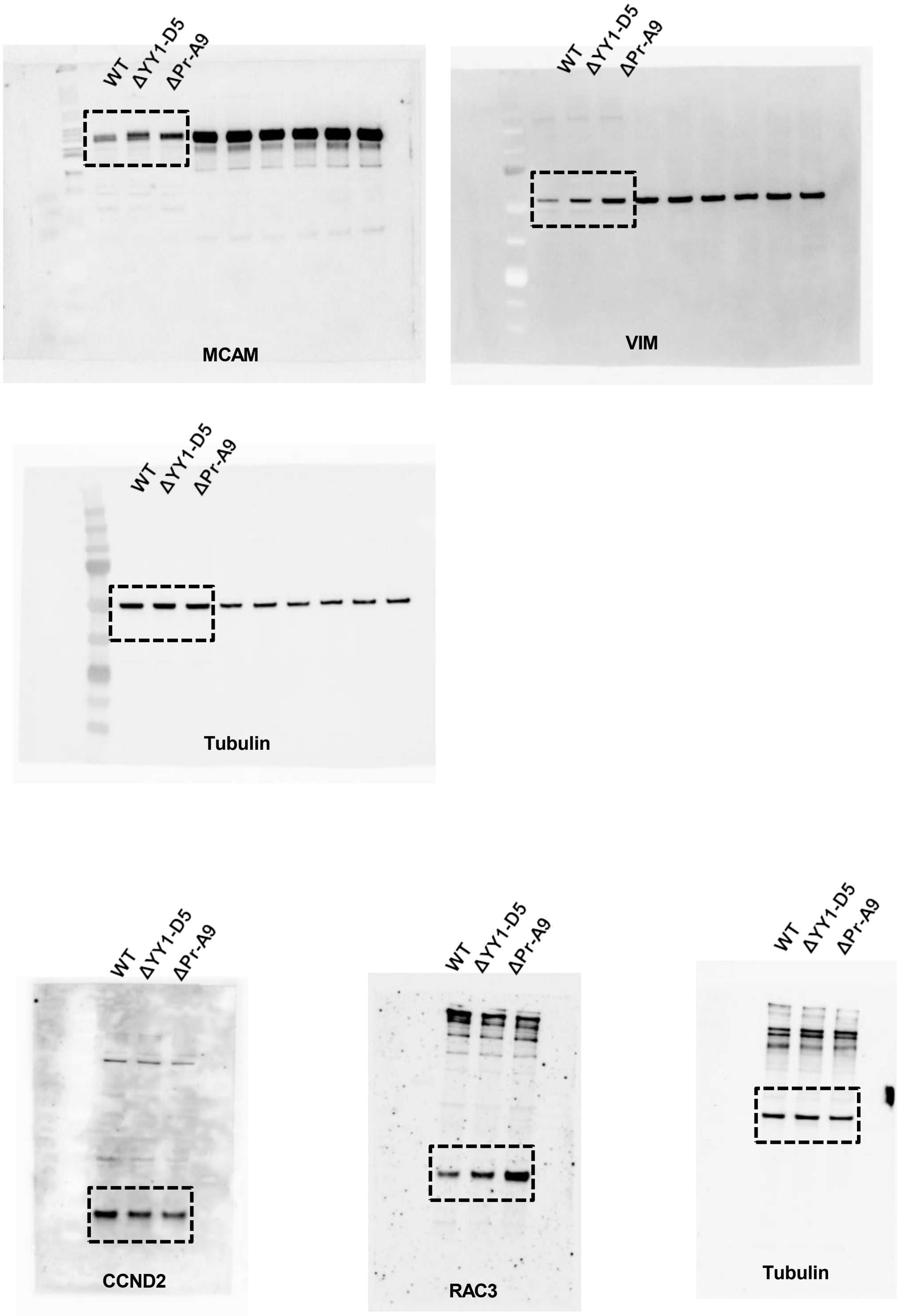

## Figure 7-figure supplement 2

**B**

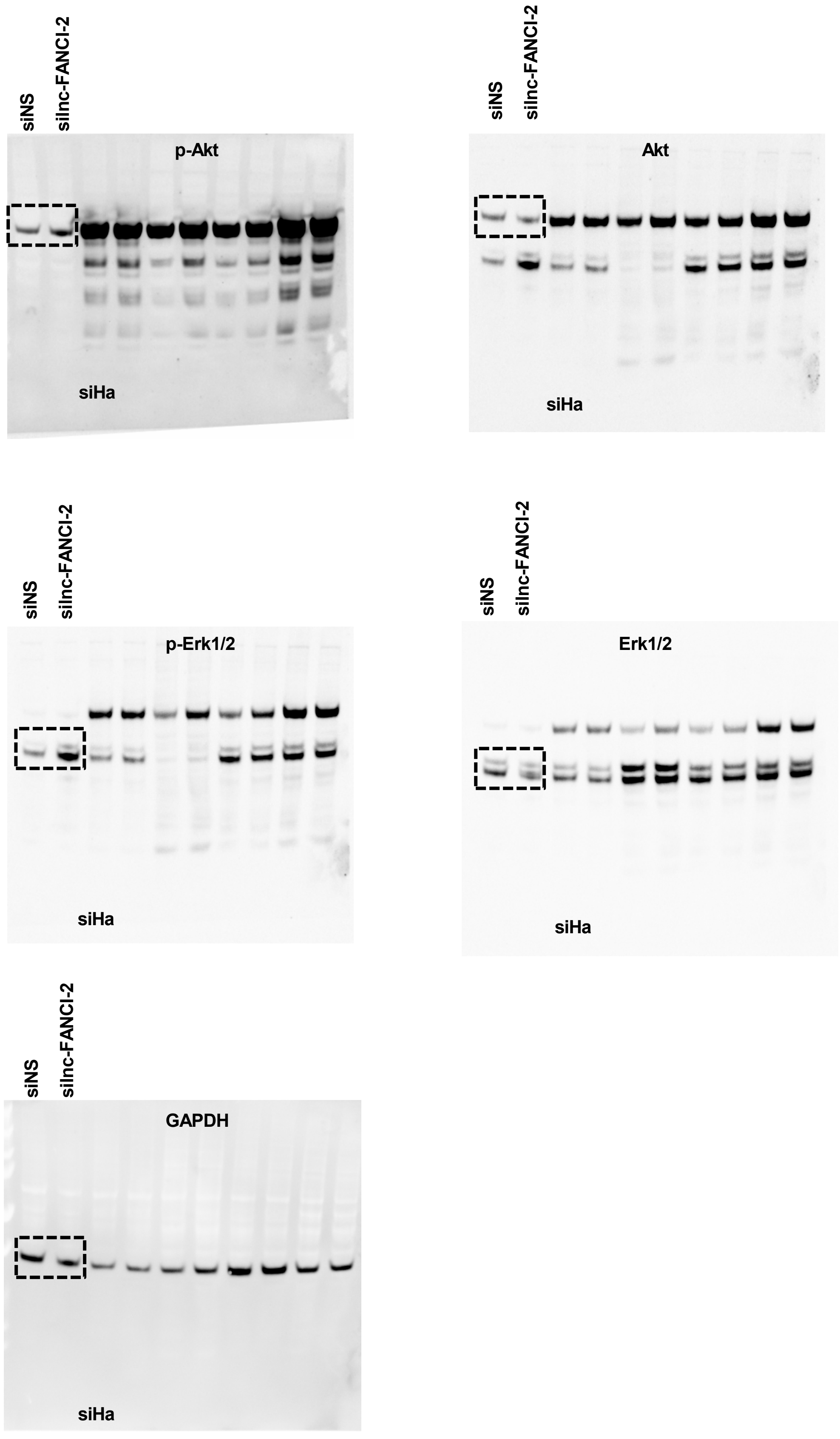

**Figure 7-figure supplement 2**

**B**

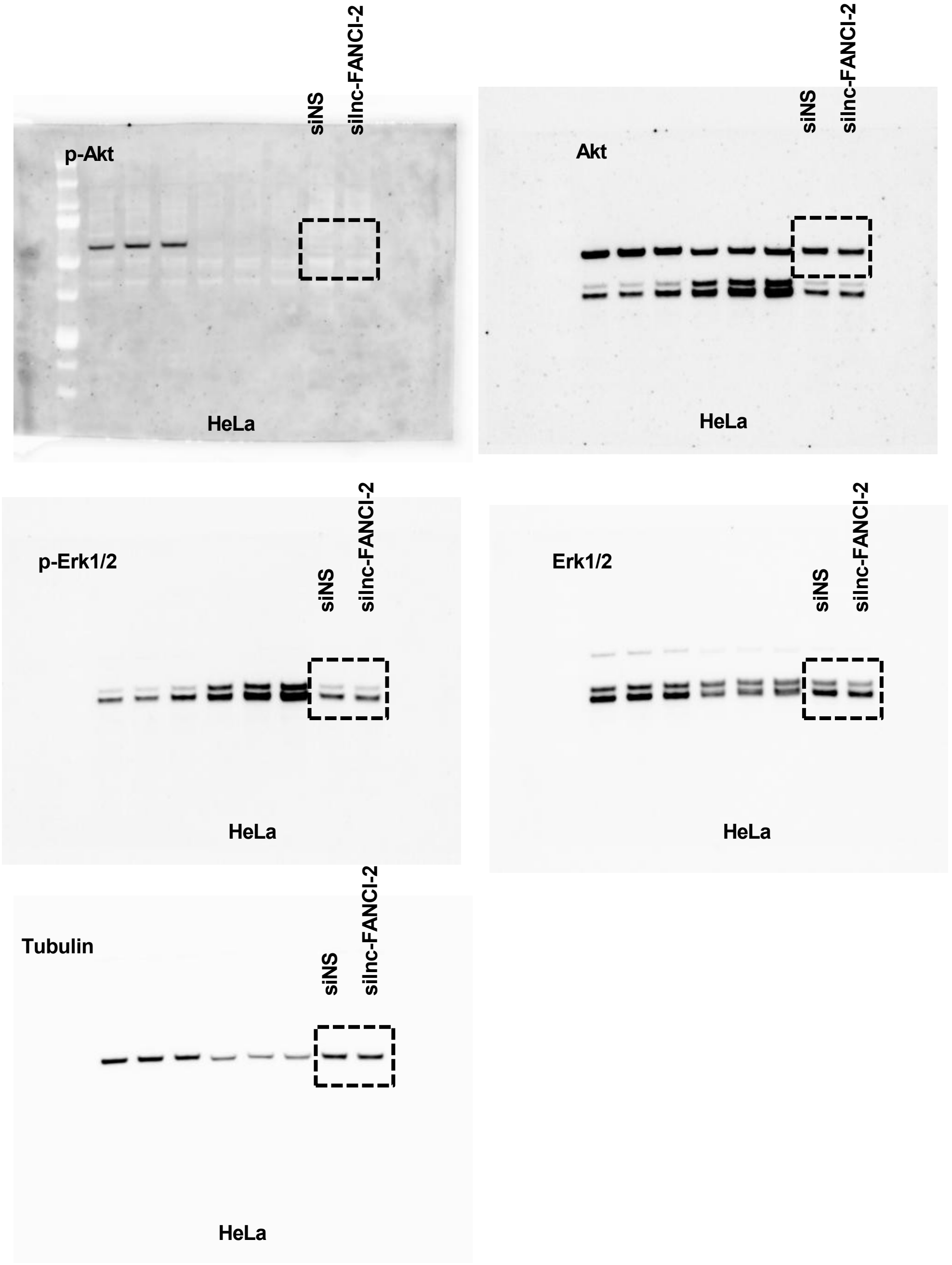

Supplement: Figure 7—figure supplement 2—source data 1. [file elife-102681-fig7-figsupp2-data1.pdf]
