# Supplementary figures and images for: The long noncoding RNA lnc-FANCI-2 intrinsically restricts RAS signaling in human papillomavirus type 16-infected cervical cancer cells (part 2 of 2)
Source: eLife. 2025 Aug 29;13:RP102681. doi: 10.7554/eLife.102681 (PMC12396819; doi:10.7554/eLife.102681)

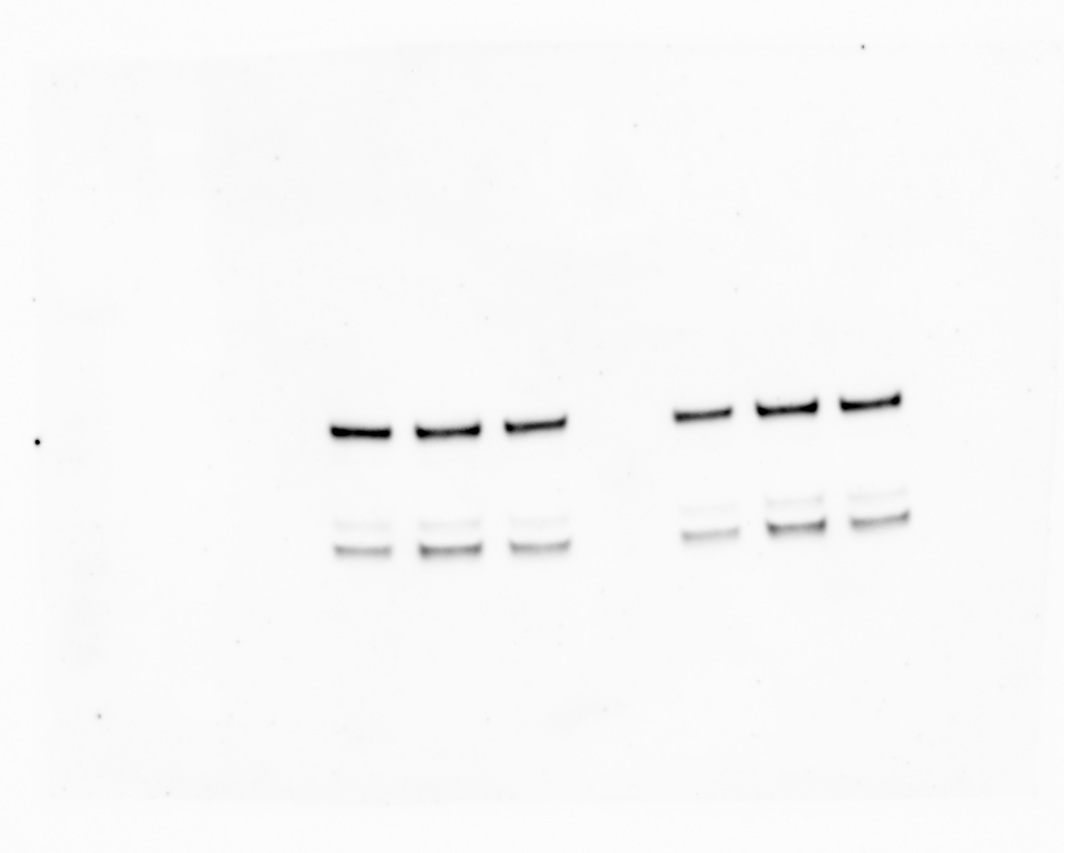

Supplement: Figure 8—source data 2. [file elife-102681-fig8-data2.zip › Figure 8-source data_2/zheng lab 2021-02-05 12h51m30s.tif]

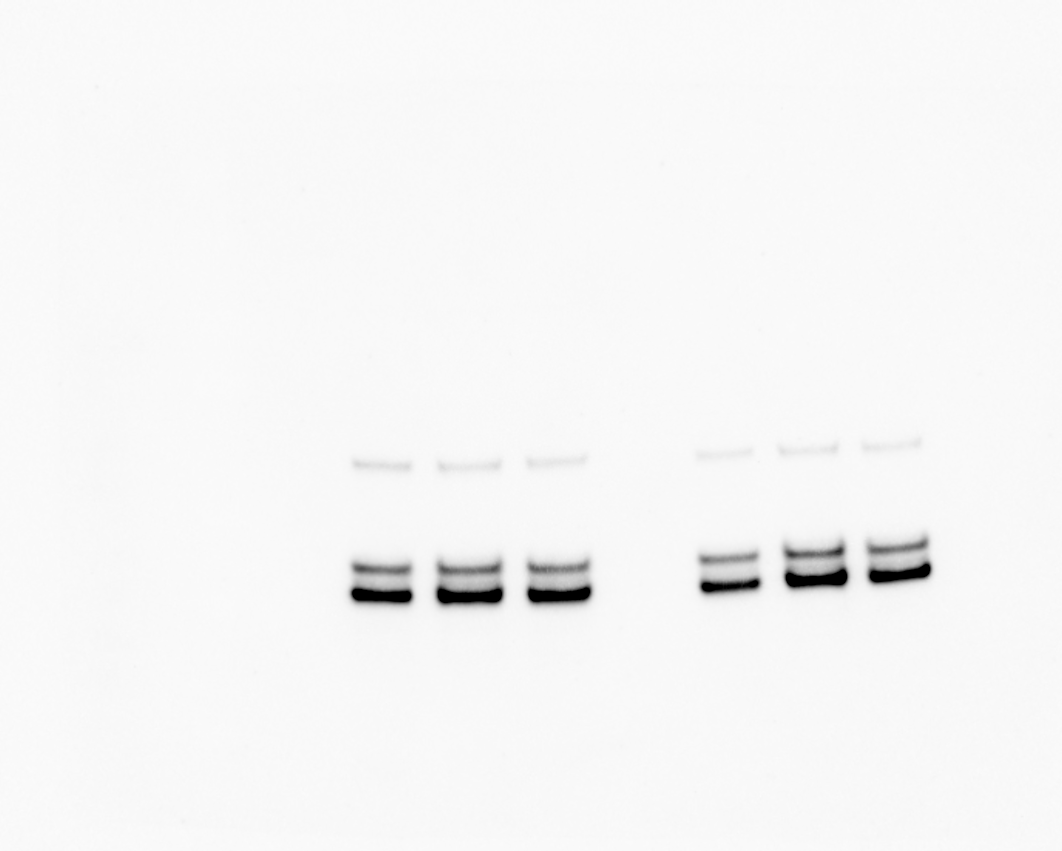

Supplement: Figure 8—source data 2. [file elife-102681-fig8-data2.zip › Figure 8-source data_2/zheng lab 2021-02-08 13h01m45s.tif]

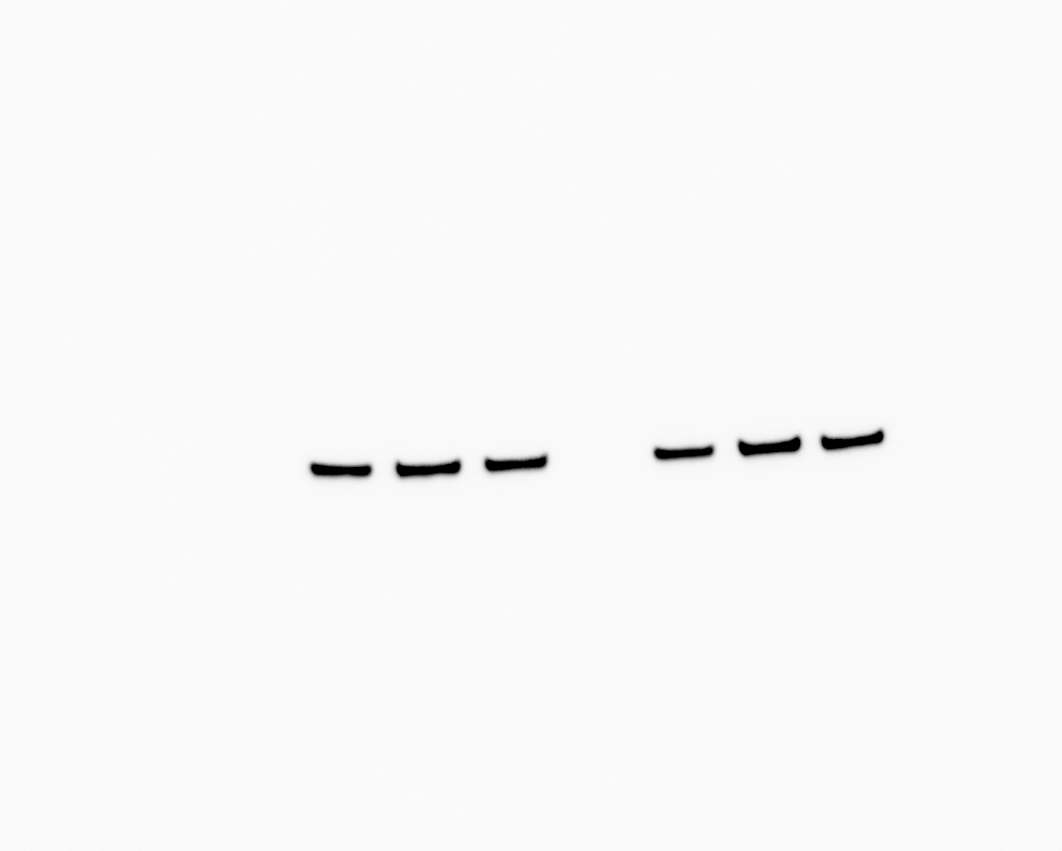

Supplement: Figure 8—source data 2. [file elife-102681-fig8-data2.zip › Figure 8-source data_2/zheng lab 2021-02-09 15h09m37s.tif]

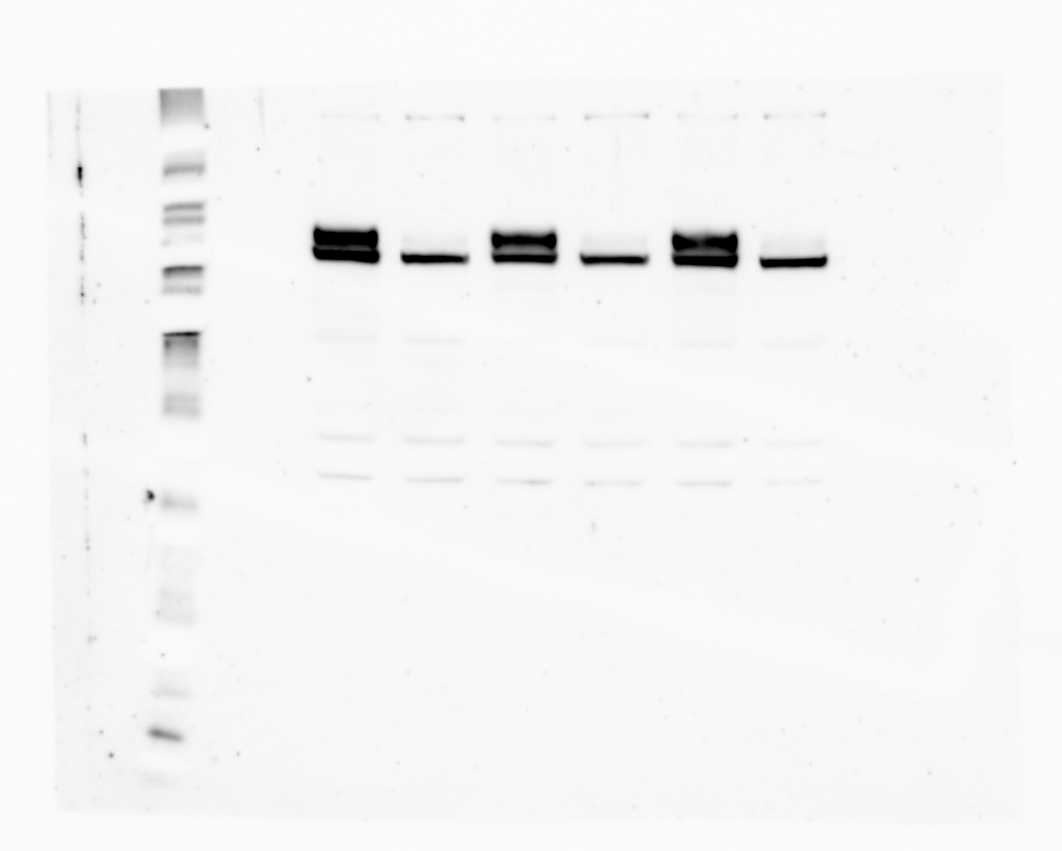

Supplement: Figure 8—source data 2. [file elife-102681-fig8-data2.zip › Figure 8-source data_2/zheng lab 2021-06-23 12h23m57s.tif]

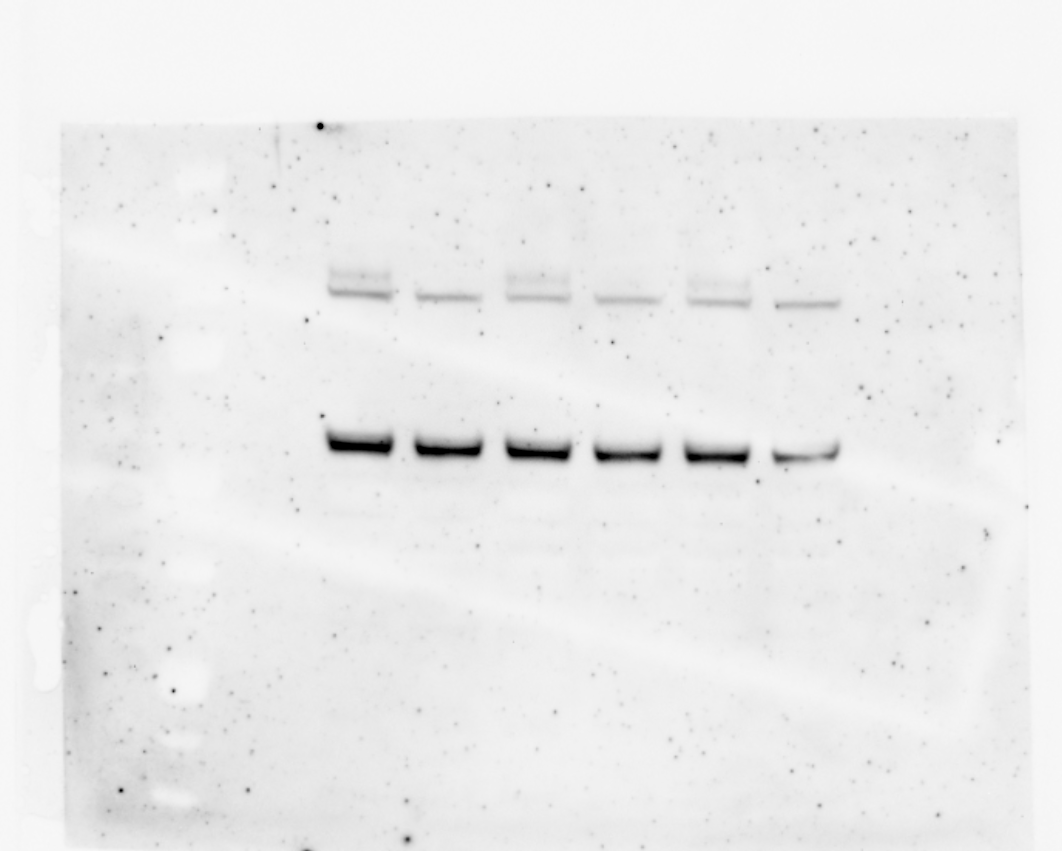

Supplement: Figure 8—source data 2. [file elife-102681-fig8-data2.zip › Figure 8-source data_2/zheng lab 2021-06-24 12h47m15s.tif]

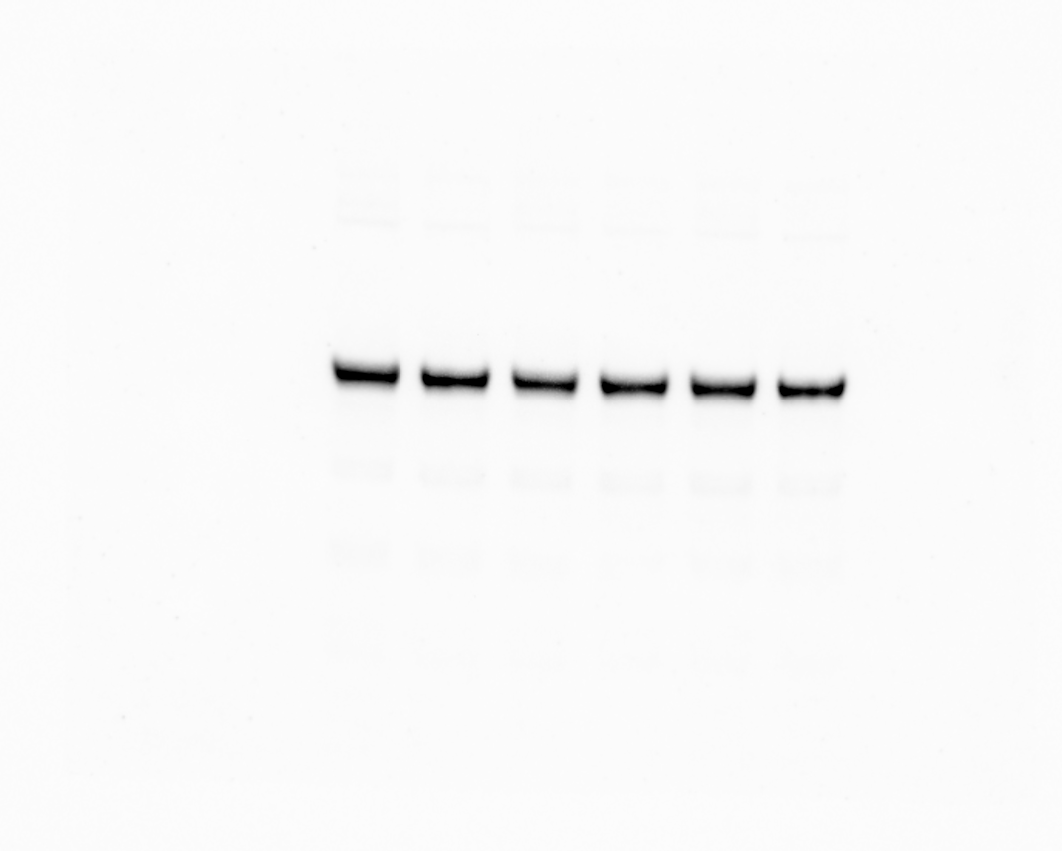

Supplement: Figure 8—source data 2. [file elife-102681-fig8-data2.zip › Figure 8-source data_2/zheng lab 2021-06-25 12h40m50s.tif]

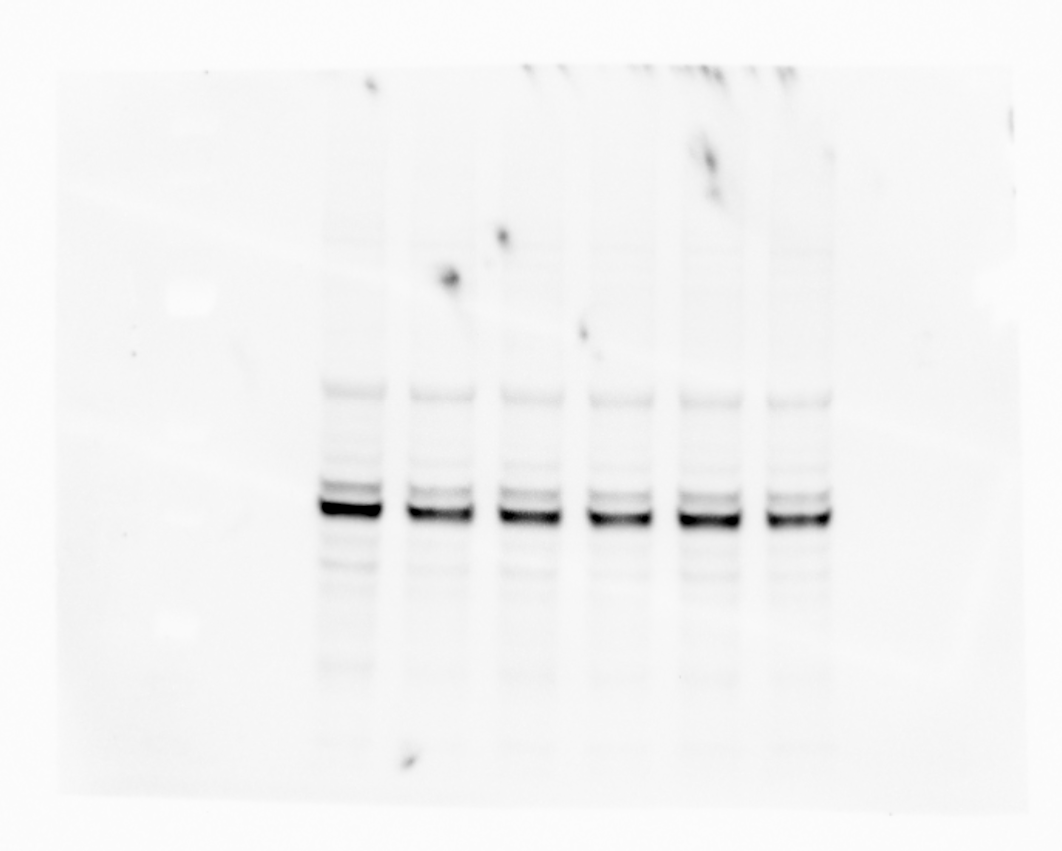

Supplement: Figure 8—source data 2. [file elife-102681-fig8-data2.zip › Figure 8-source data_2/zheng lab 2021-06-28 12h23m17s.tif]

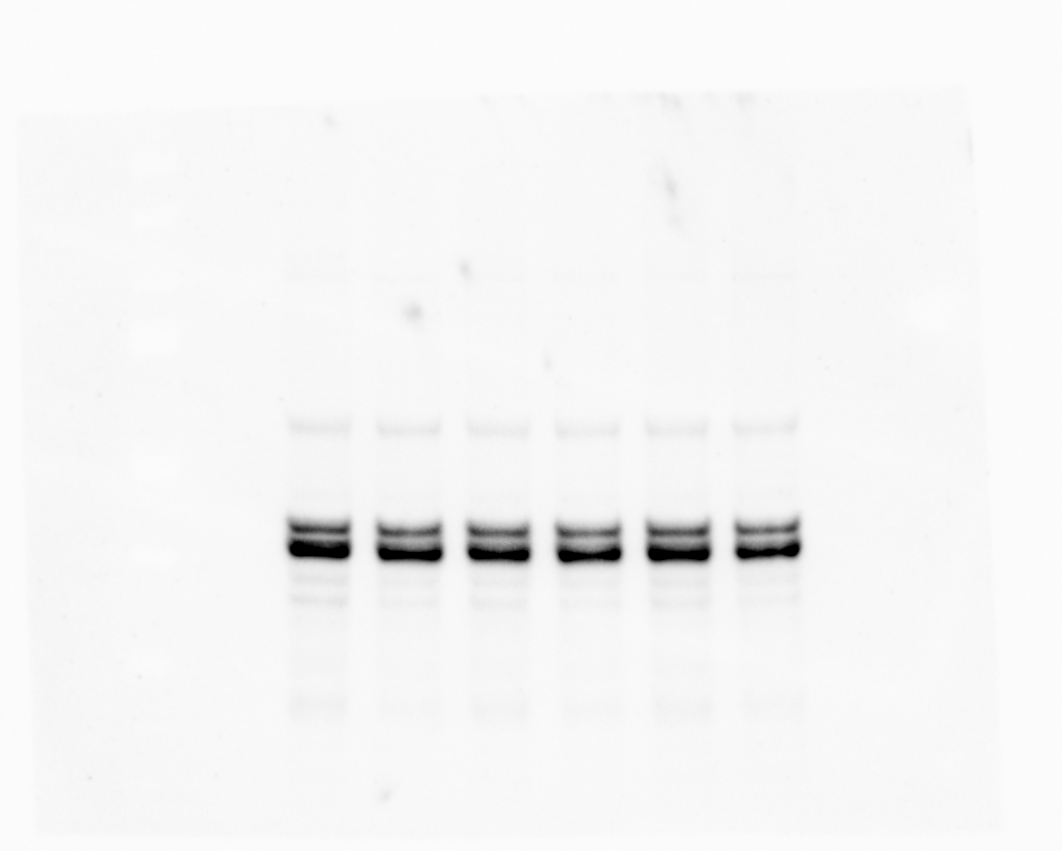

Supplement: Figure 8—source data 2. [file elife-102681-fig8-data2.zip › Figure 8-source data_2/zheng lab 2021-06-29 11h46m41s.tif]

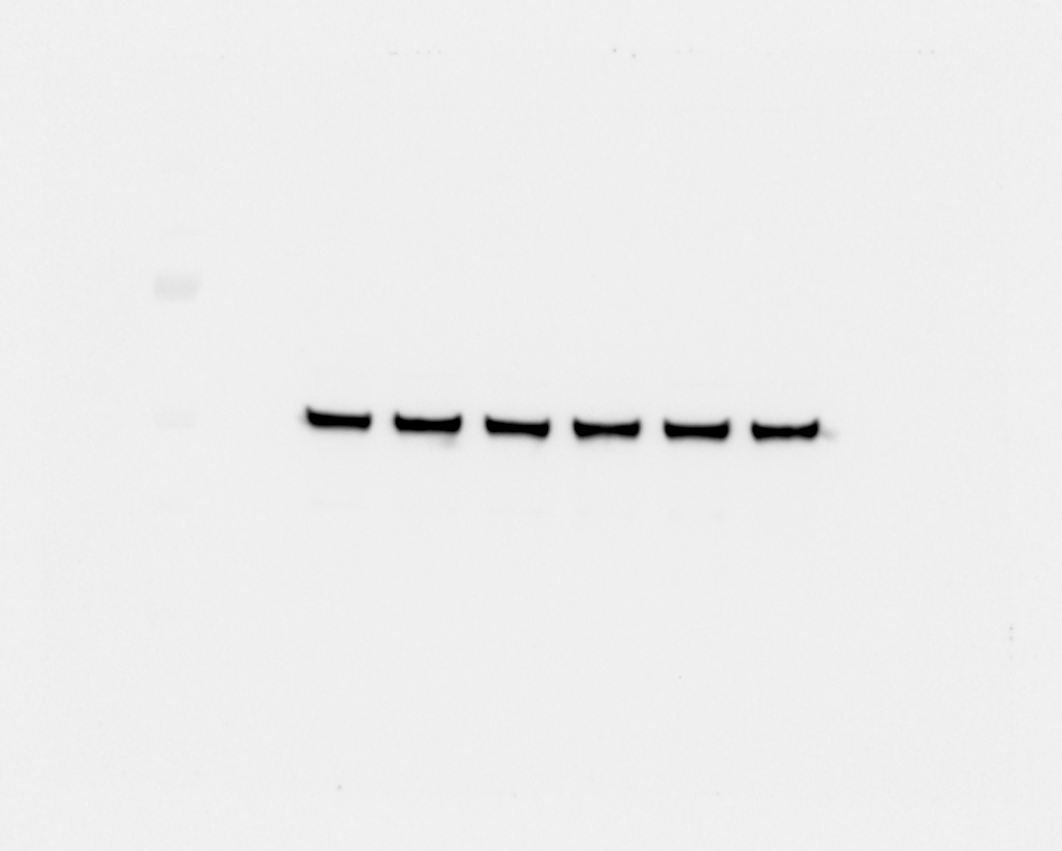

Supplement: Figure 8—source data 2. [file elife-102681-fig8-data2.zip › Figure 8-source data_2/zheng lab 2021-06-30 12h30m56s.tif]

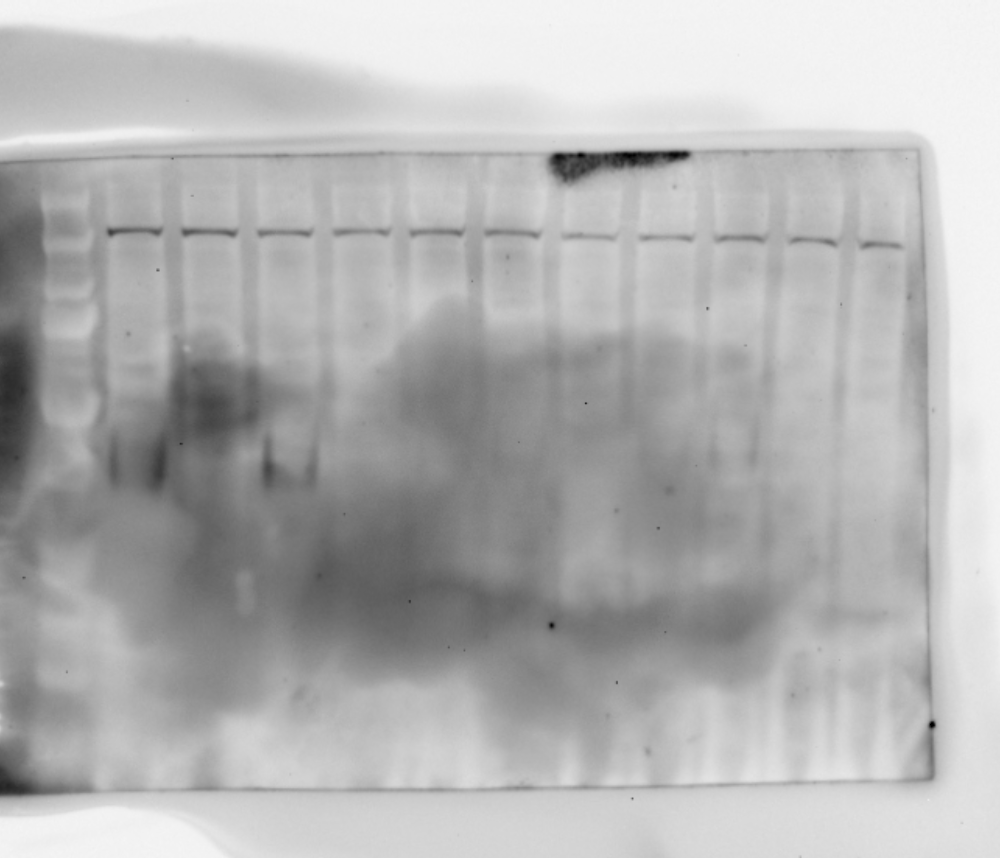

Supplement: Figure 8—source data 2. [file elife-102681-fig8-data2.zip › Figure 8-source data_2/zheng lab 2024-07-07 08h28m06s.tif]

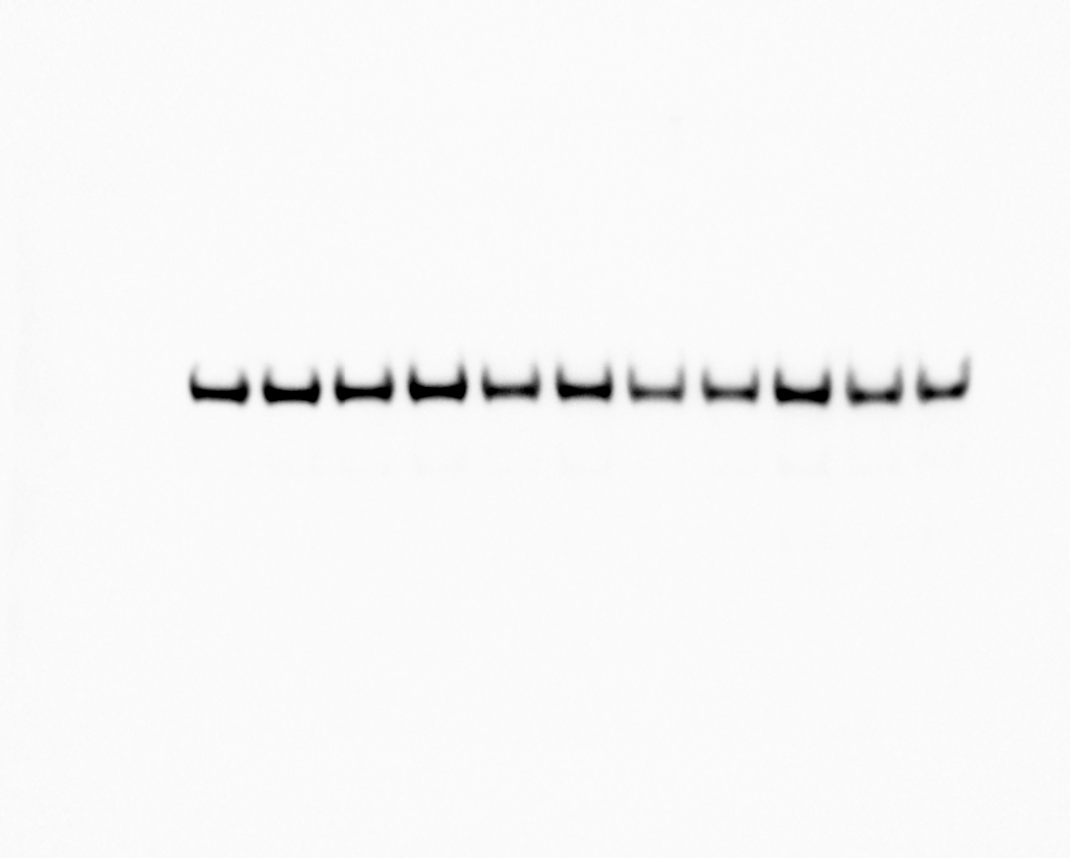

Supplement: Figure 8—source data 2. [file elife-102681-fig8-data2.zip › Figure 8-source data_2/zheng lab 2024-07-08 08h17m18s.tif]

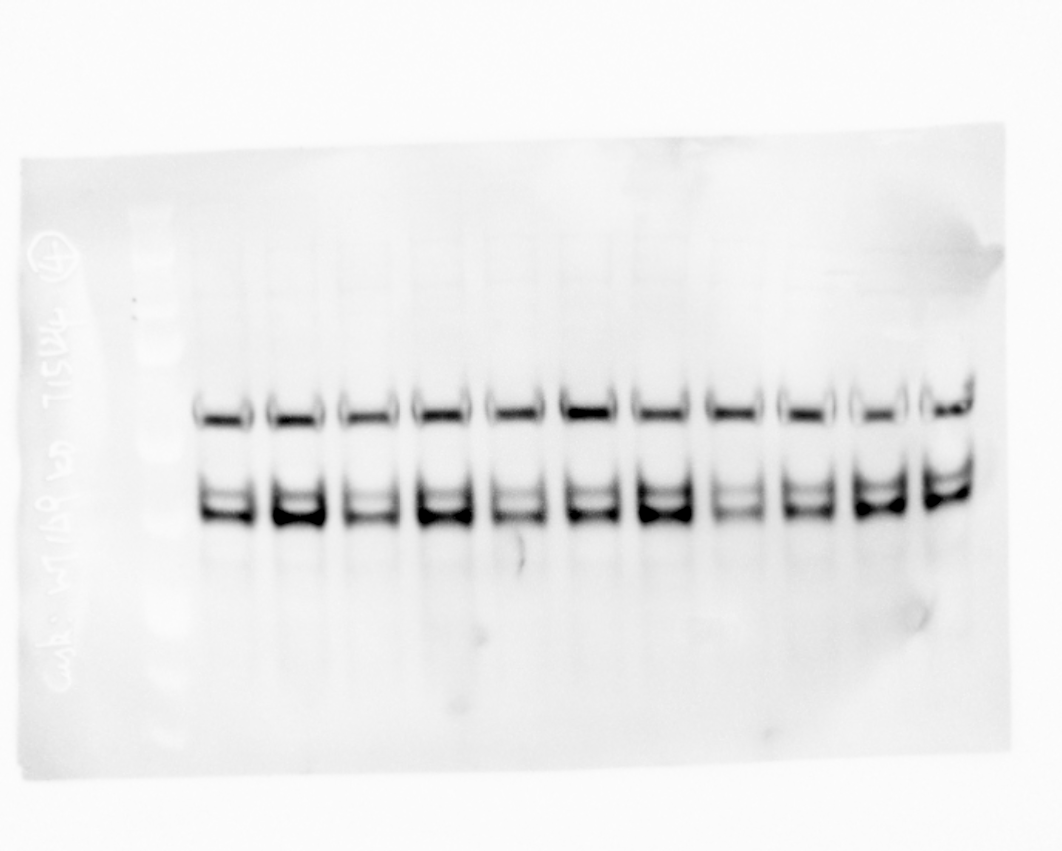

Supplement: Figure 8—source data 2. [file elife-102681-fig8-data2.zip › Figure 8-source data_2/zheng lab 2024-07-09 11h19m46s.tif]

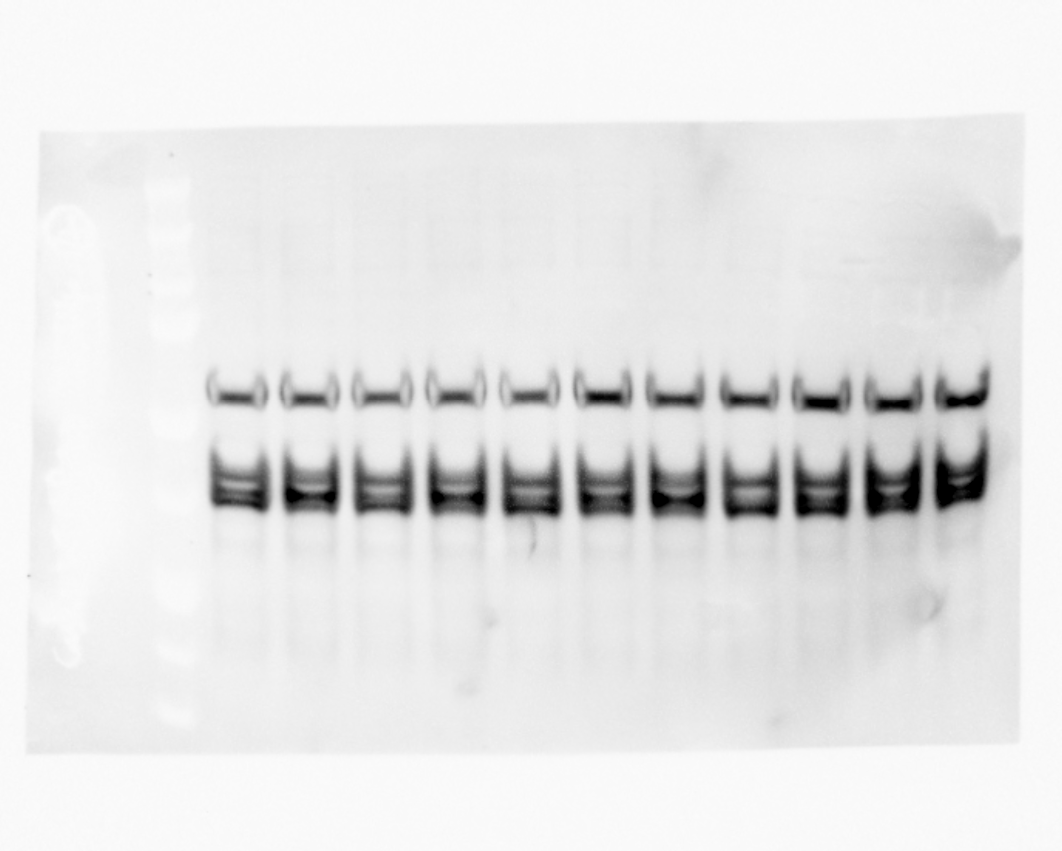

Supplement: Figure 8—source data 2. [file elife-102681-fig8-data2.zip › Figure 8-source data_2/zheng lab 2024-07-10 10h21m50s.tif]

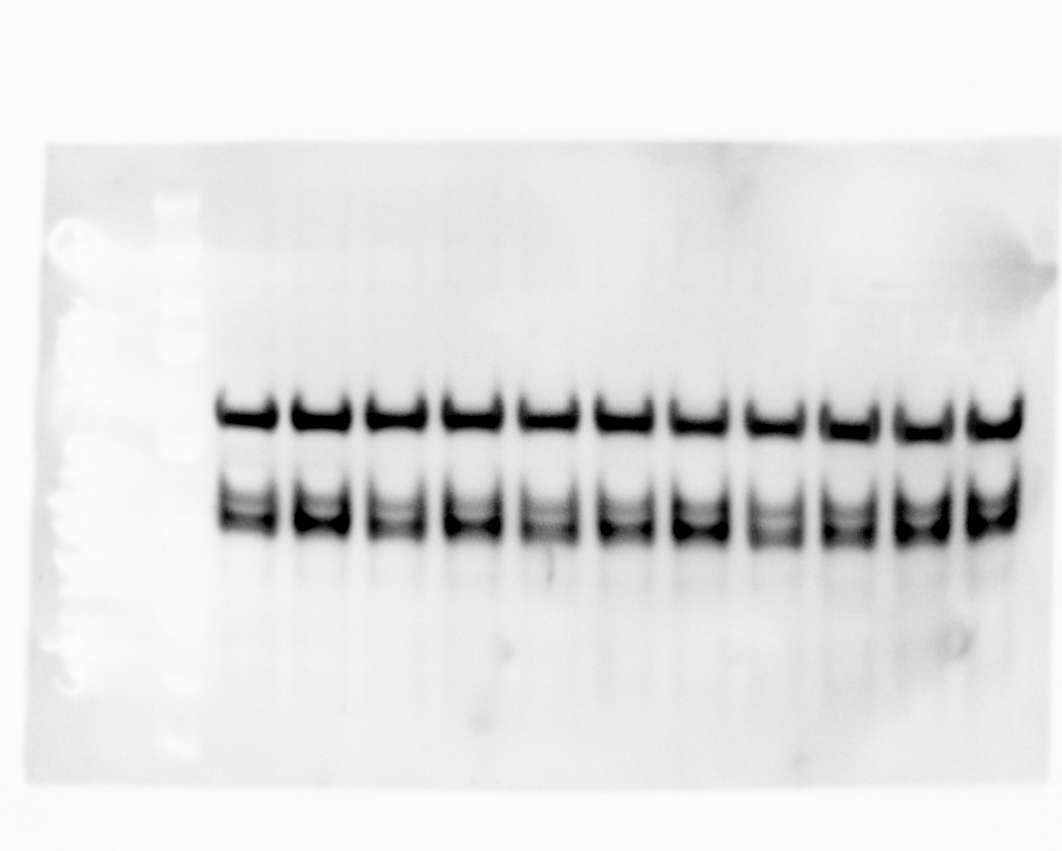

Supplement: Figure 8—source data 2. [file elife-102681-fig8-data2.zip › Figure 8-source data_2/zheng lab 2024-07-11 10h02m47s.tif]

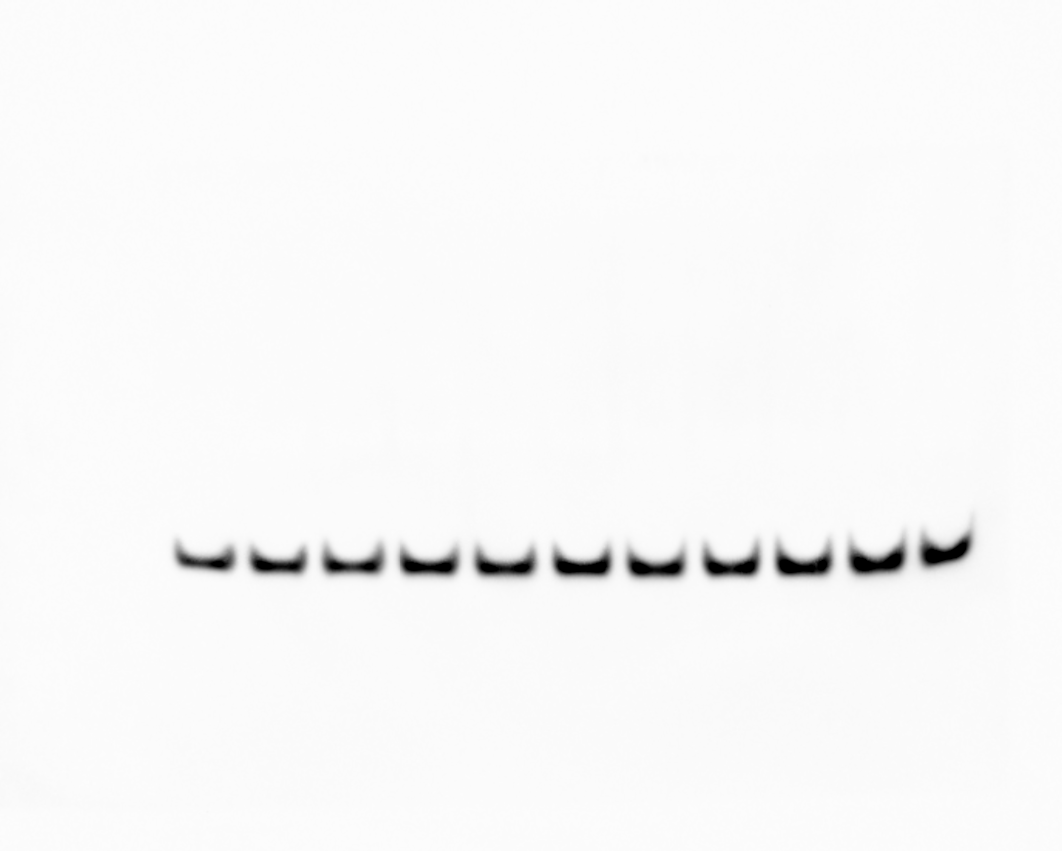

Supplement: Figure 8—source data 2. [file elife-102681-fig8-data2.zip › Figure 8-source data_2/zheng lab 2024-07-11 14h45m38s.tif]

Figure 8-figure supplement 1

C

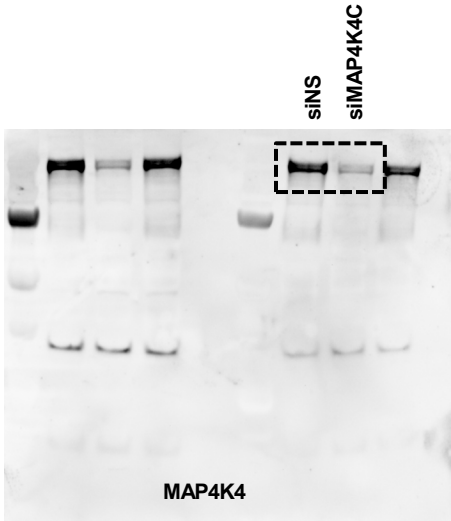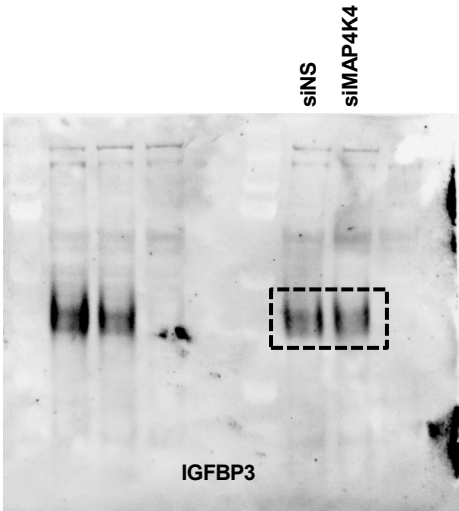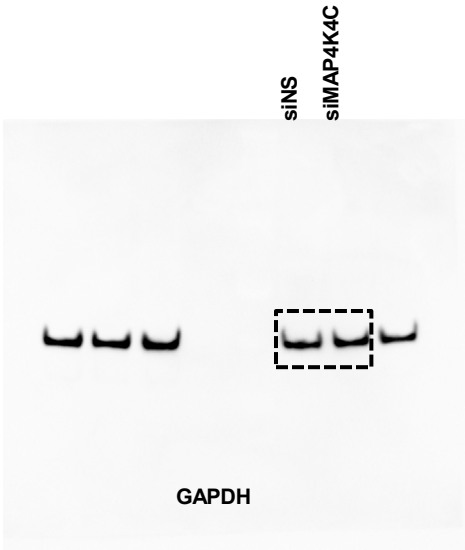

Figure 8-figure supplement 1

C

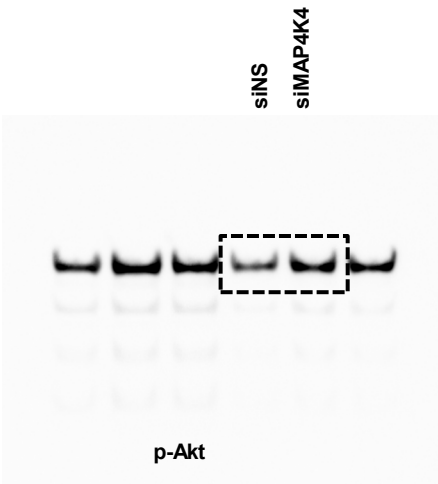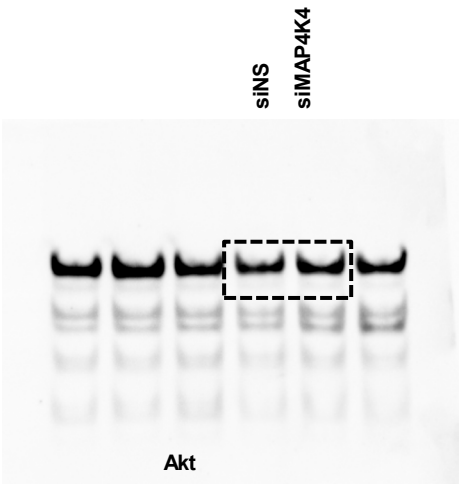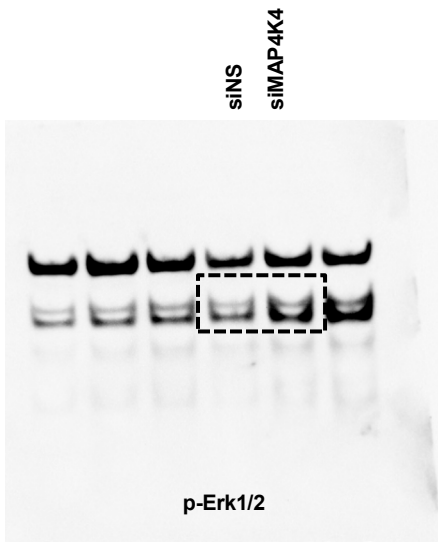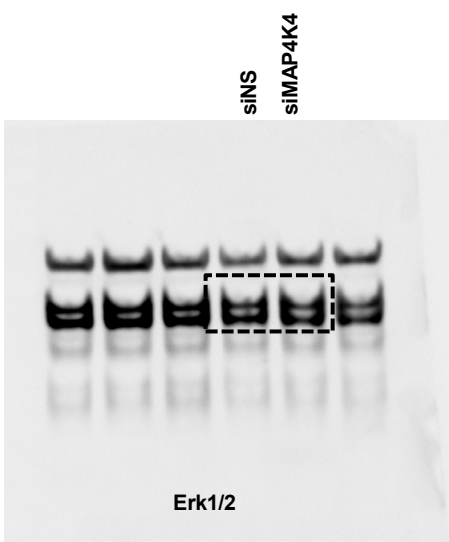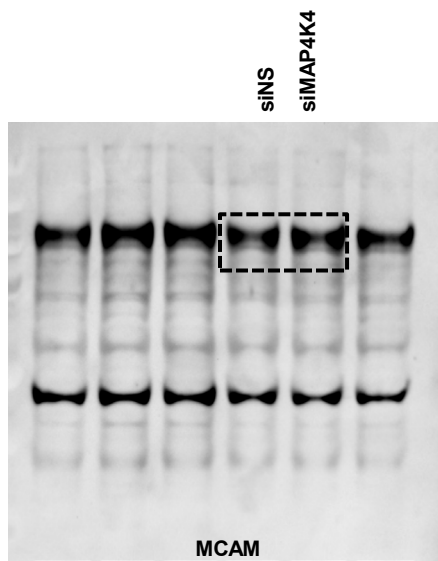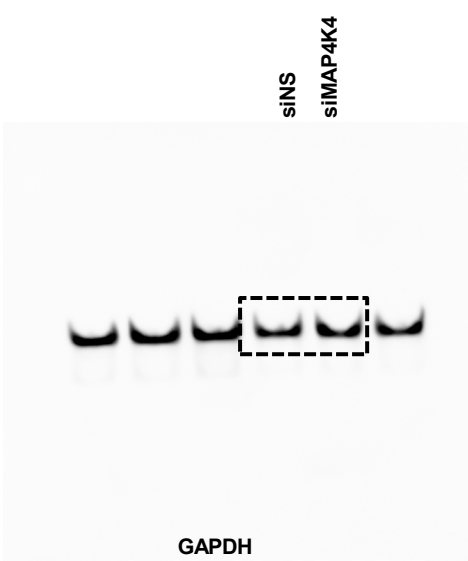

Supplement: Figure 8—figure supplement 1—source data 1. [file elife-102681-fig8-figsupp1-data1.pdf]

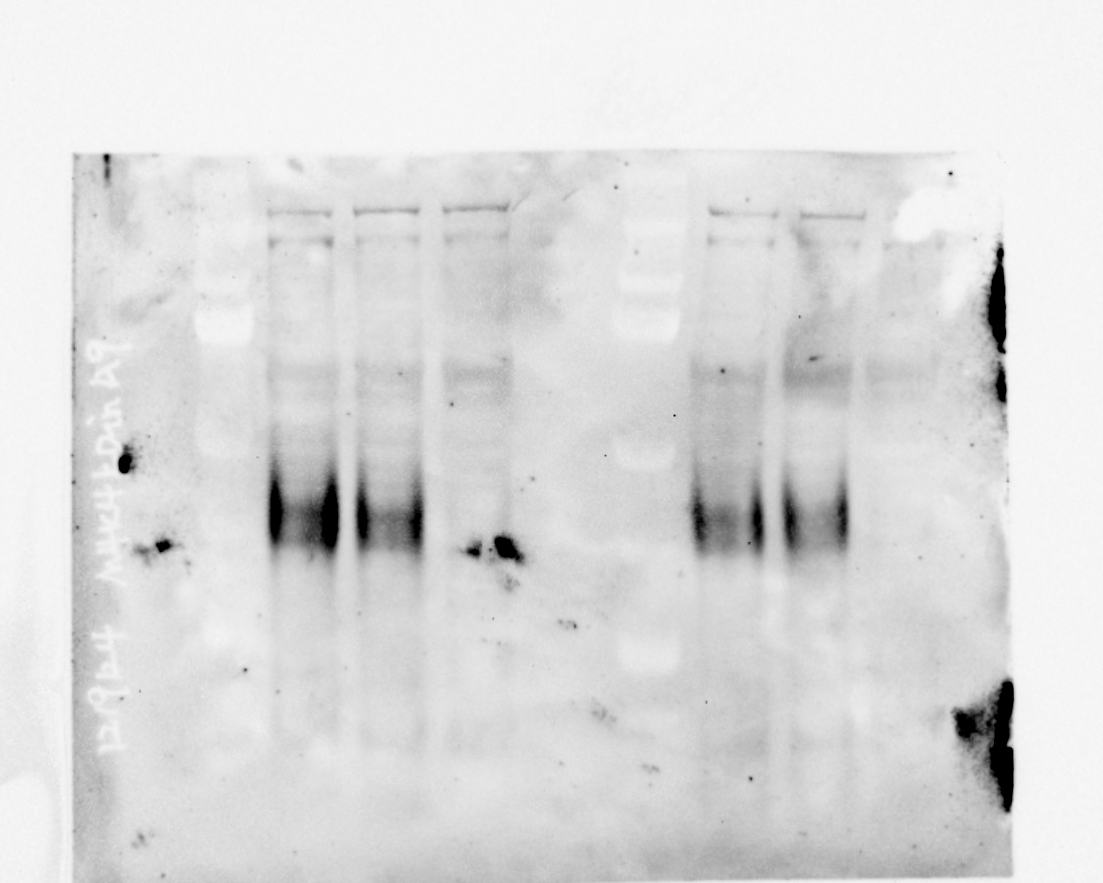

Supplement: Figure 8—figure supplement 1—source data 2. [file elife-102681-fig8-figsupp1-data2.zip › Figure 8-figure supplement 1-source_data_2/zheng lab 2024-12-12 09h48m55s.tif]

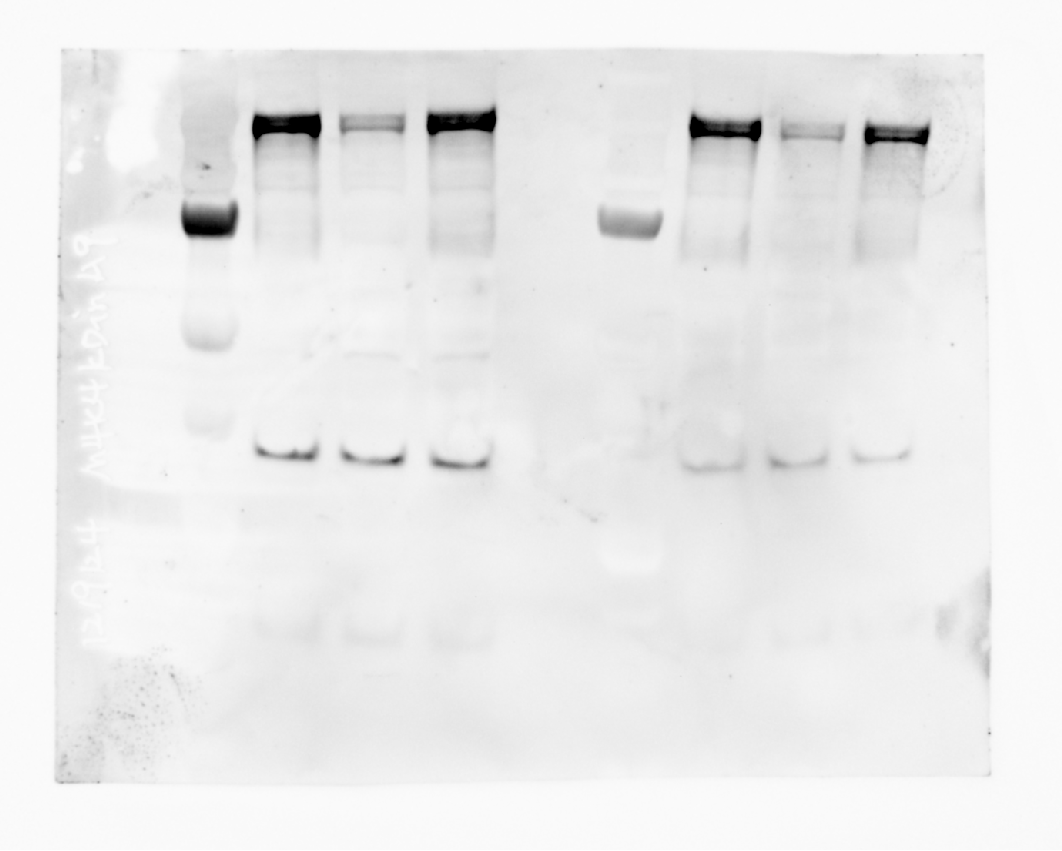

Supplement: Figure 8—figure supplement 1—source data 2. [file elife-102681-fig8-figsupp1-data2.zip › Figure 8-figure supplement 1-source_data_2/zheng lab 2024-12-13 10h01m14s.tif]

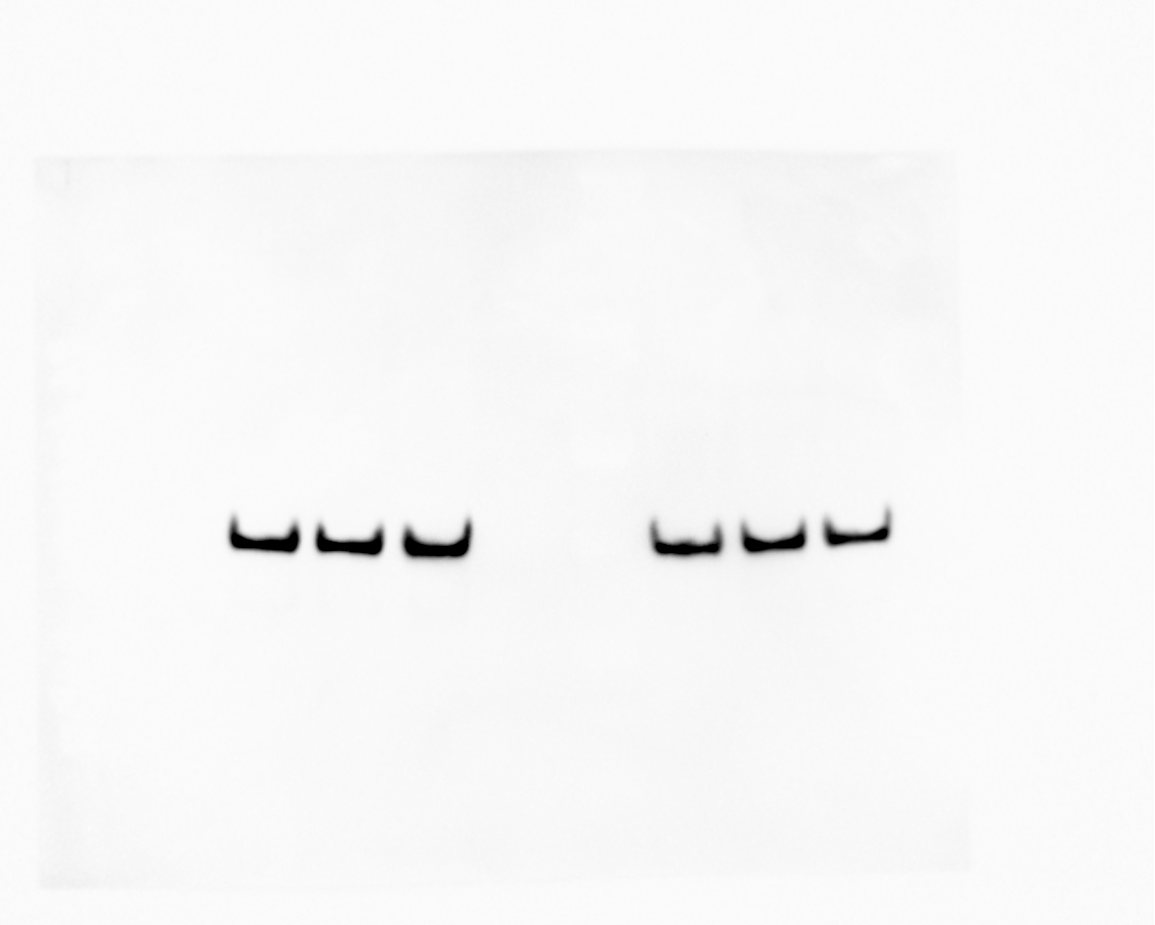

Supplement: Figure 8—figure supplement 1—source data 2. [file elife-102681-fig8-figsupp1-data2.zip › Figure 8-figure supplement 1-source_data_2/zheng lab 2024-12-13 14h01m27s.tif]

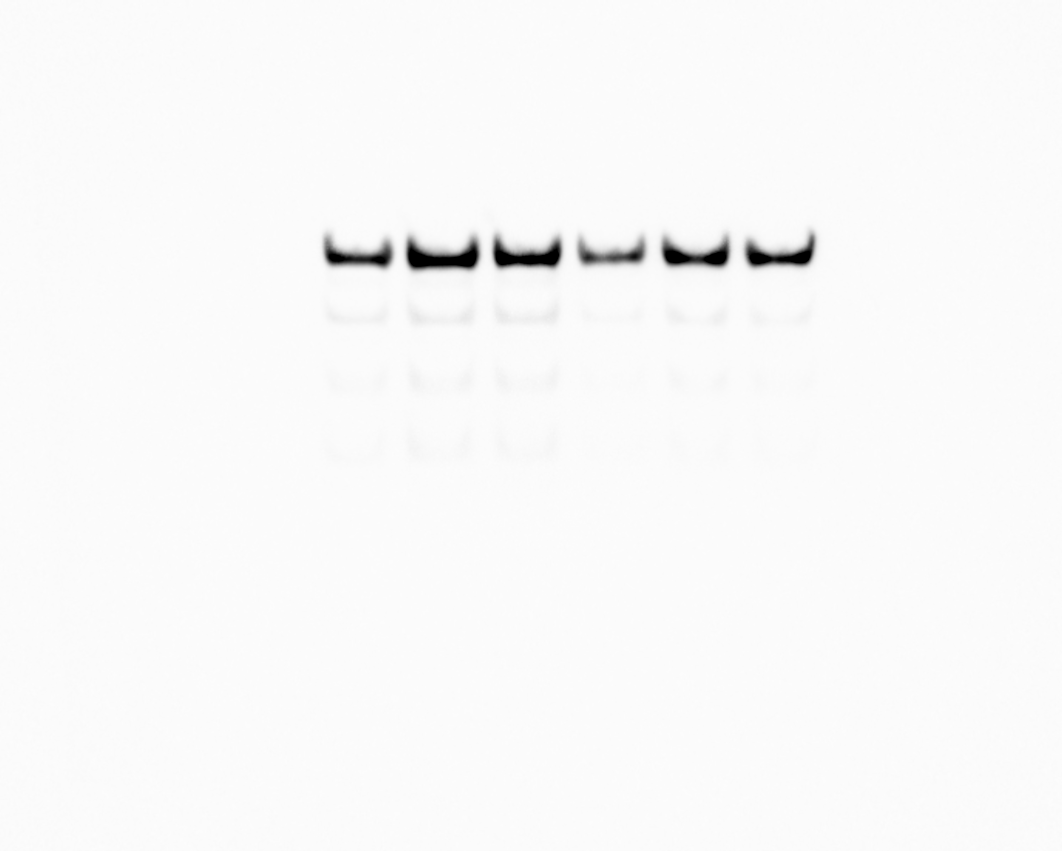

Supplement: Figure 8—figure supplement 1—source data 2. [file elife-102681-fig8-figsupp1-data2.zip › Figure 8-figure supplement 1-source_data_2/zheng lab 2025-01-22 10h27m34s.tif]

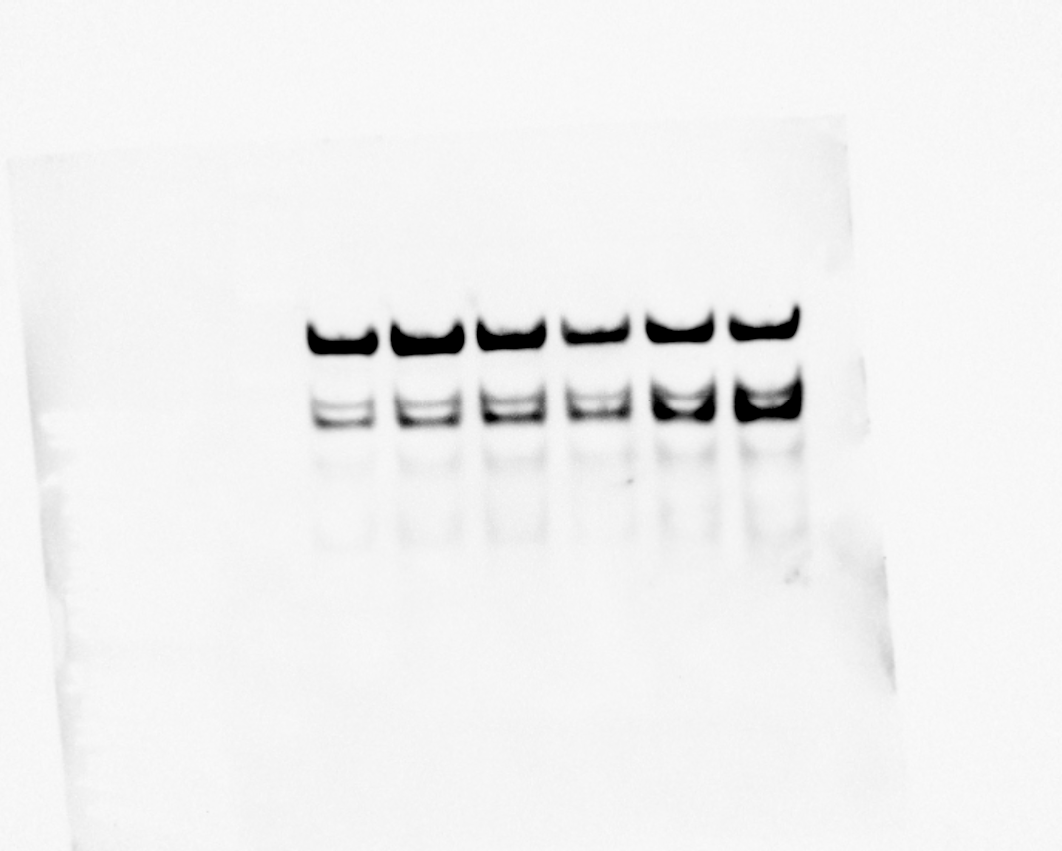

Supplement: Figure 8—figure supplement 1—source data 2. [file elife-102681-fig8-figsupp1-data2.zip › Figure 8-figure supplement 1-source_data_2/zheng lab 2025-01-23 10h46m10s.tif]

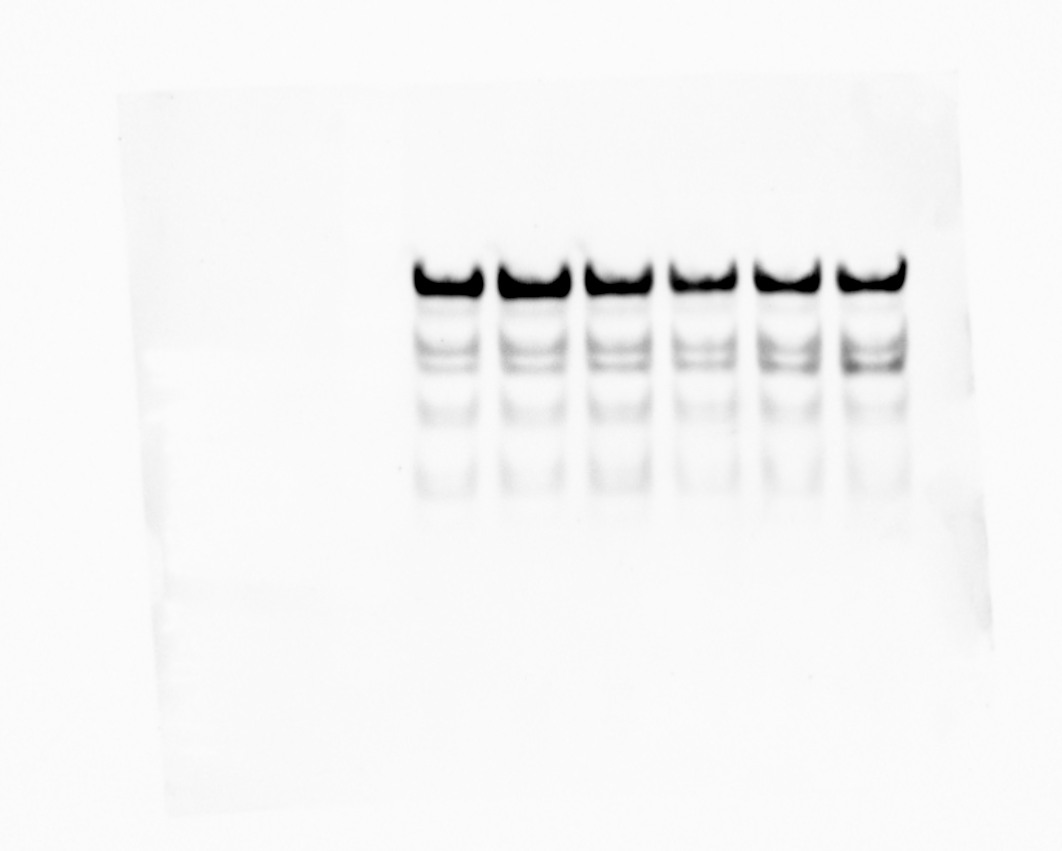

Supplement: Figure 8—figure supplement 1—source data 2. [file elife-102681-fig8-figsupp1-data2.zip › Figure 8-figure supplement 1-source_data_2/zheng lab 2025-01-27 09h44m00s.tif]

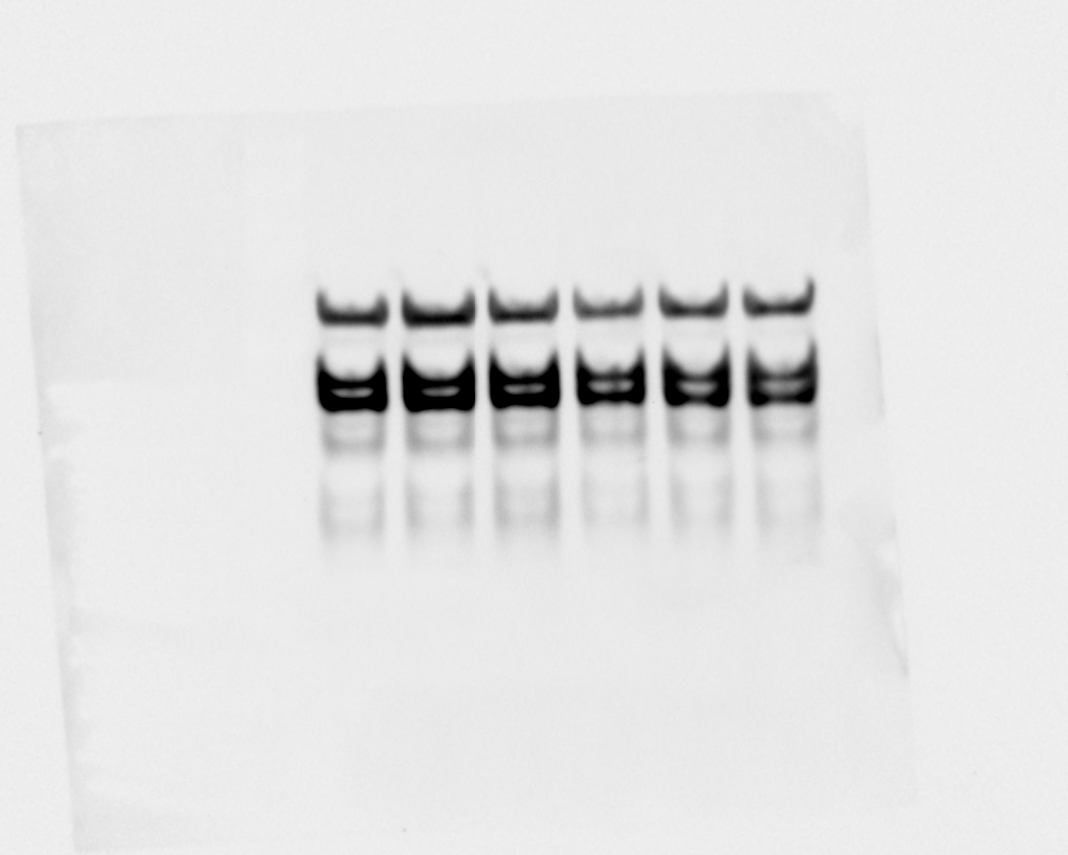

Supplement: Figure 8—figure supplement 1—source data 2. [file elife-102681-fig8-figsupp1-data2.zip › Figure 8-figure supplement 1-source_data_2/zheng lab 2025-01-30 09h56m57s.tif]

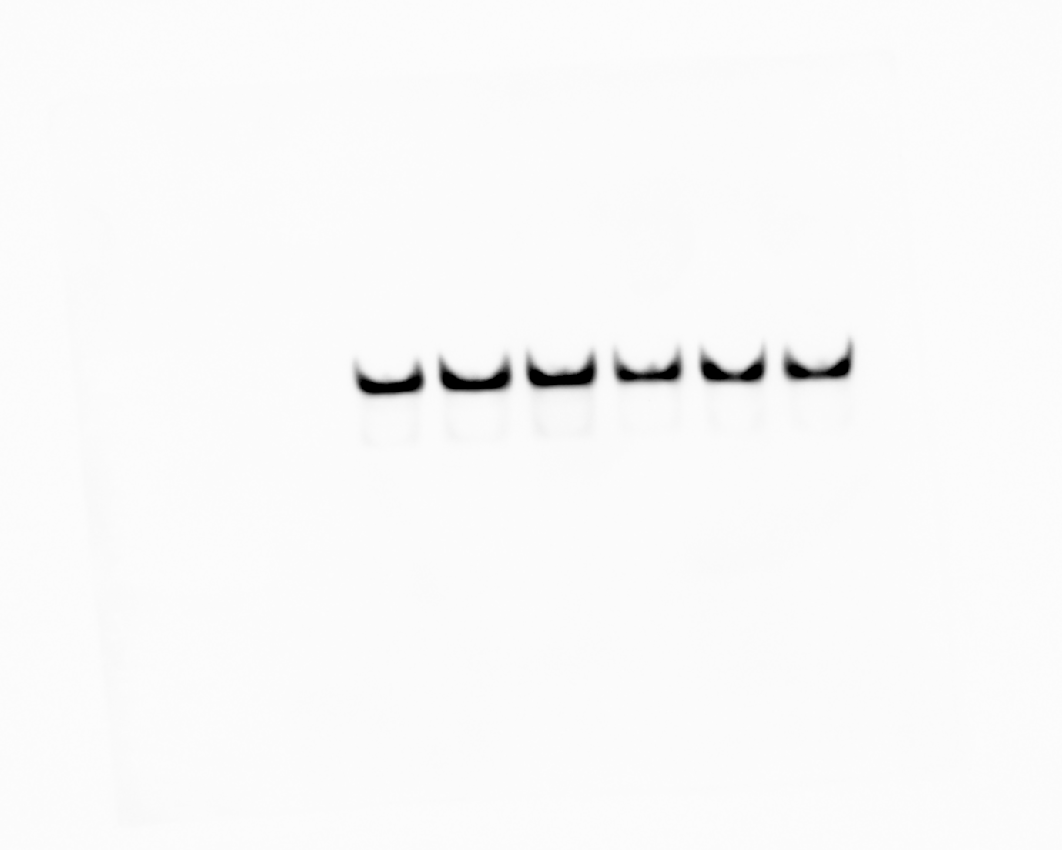

Supplement: Figure 8—figure supplement 1—source data 2. [file elife-102681-fig8-figsupp1-data2.zip › Figure 8-figure supplement 1-source_data_2/zheng lab 2025-01-30 15h06m14s.tif]

Figure 9

B

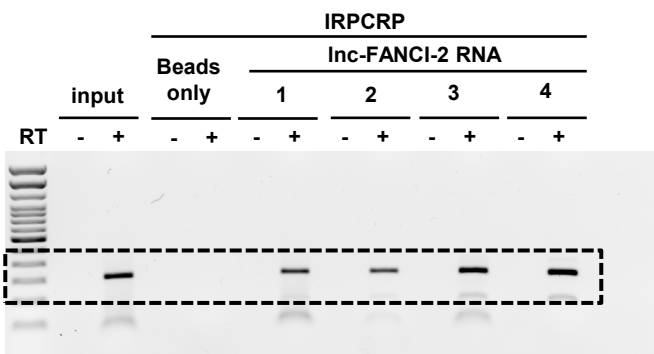

Figure 9

D

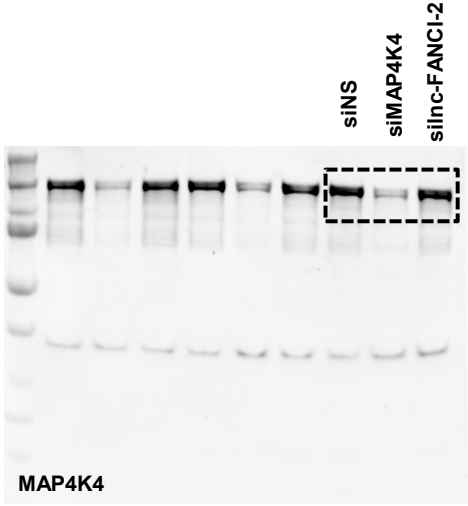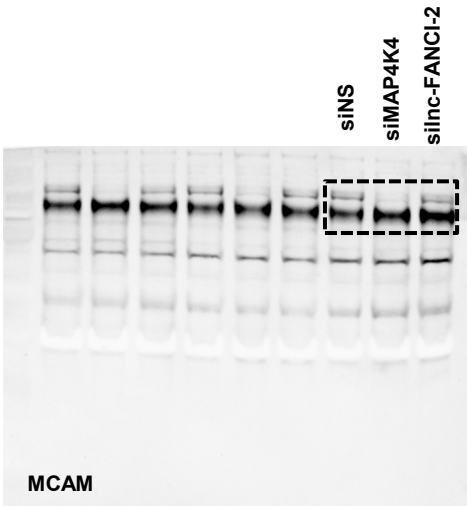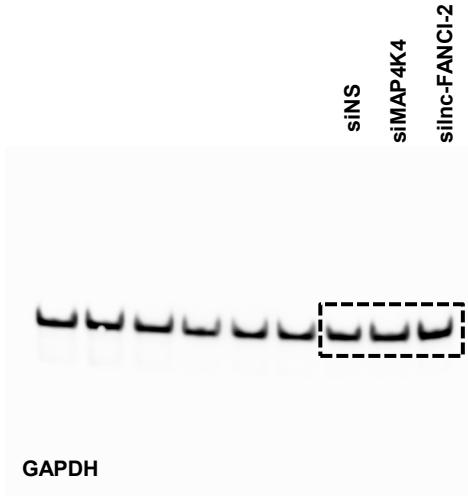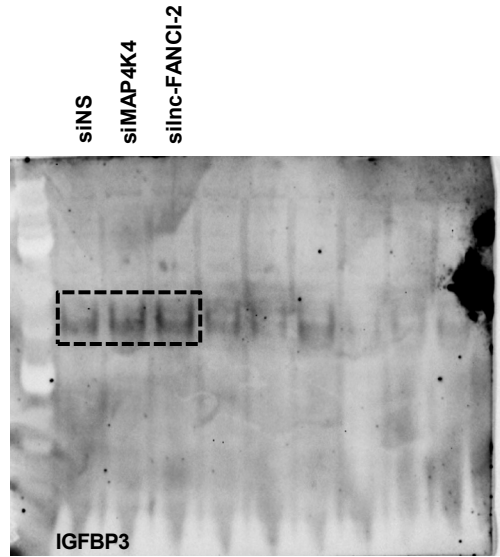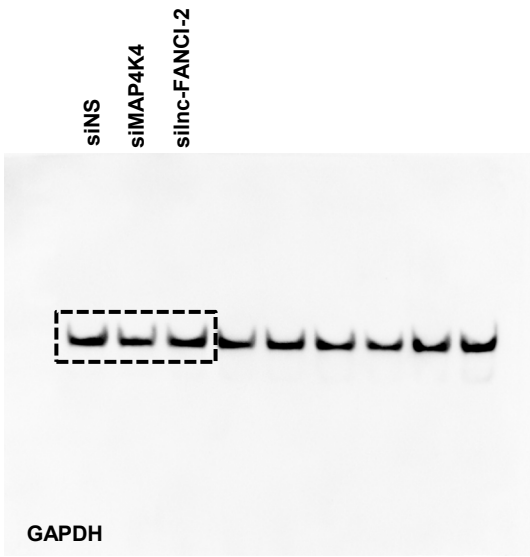

Figure 9

D

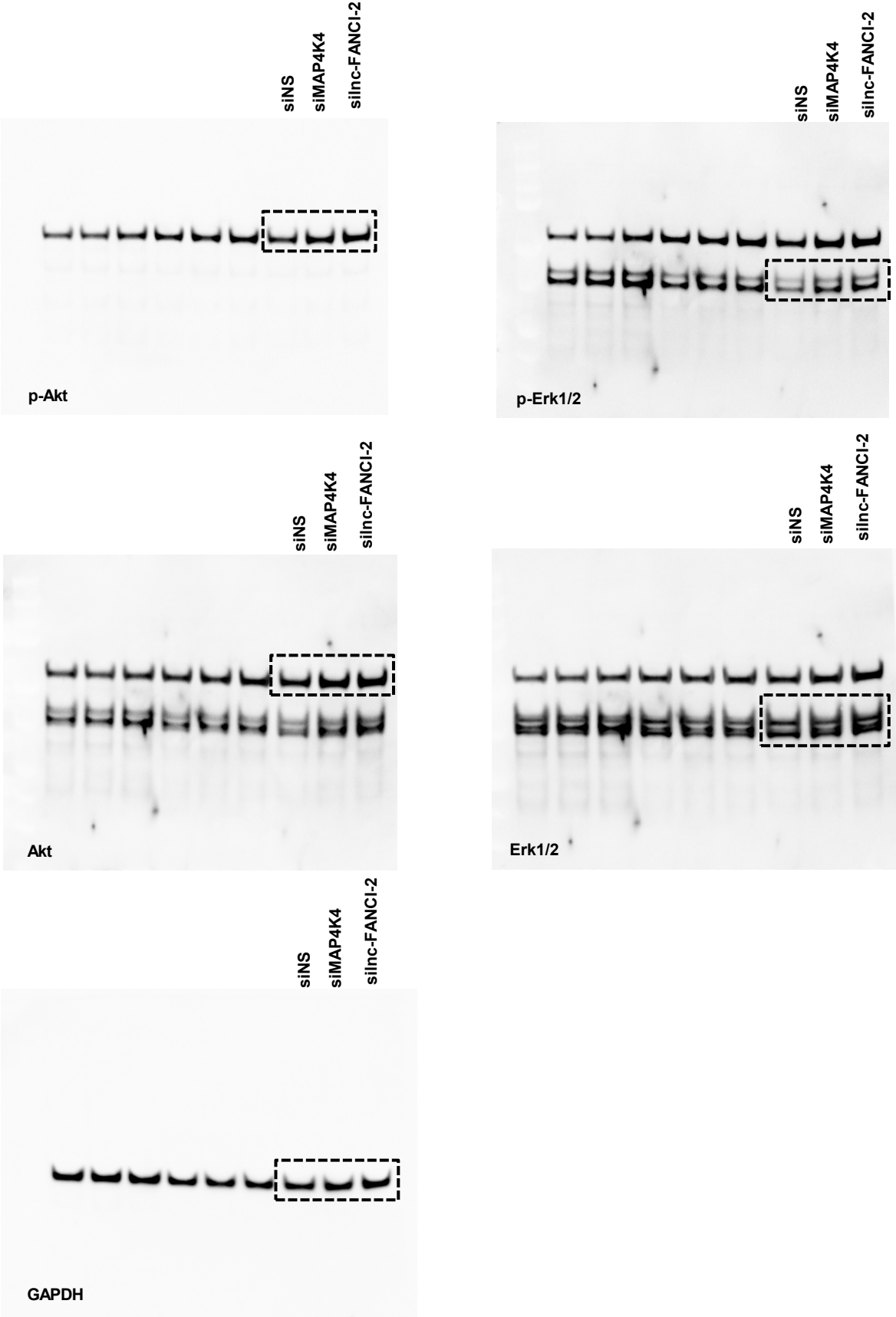

Supplement: Figure 9—source data 1. [file elife-102681-fig9-data1.pdf]

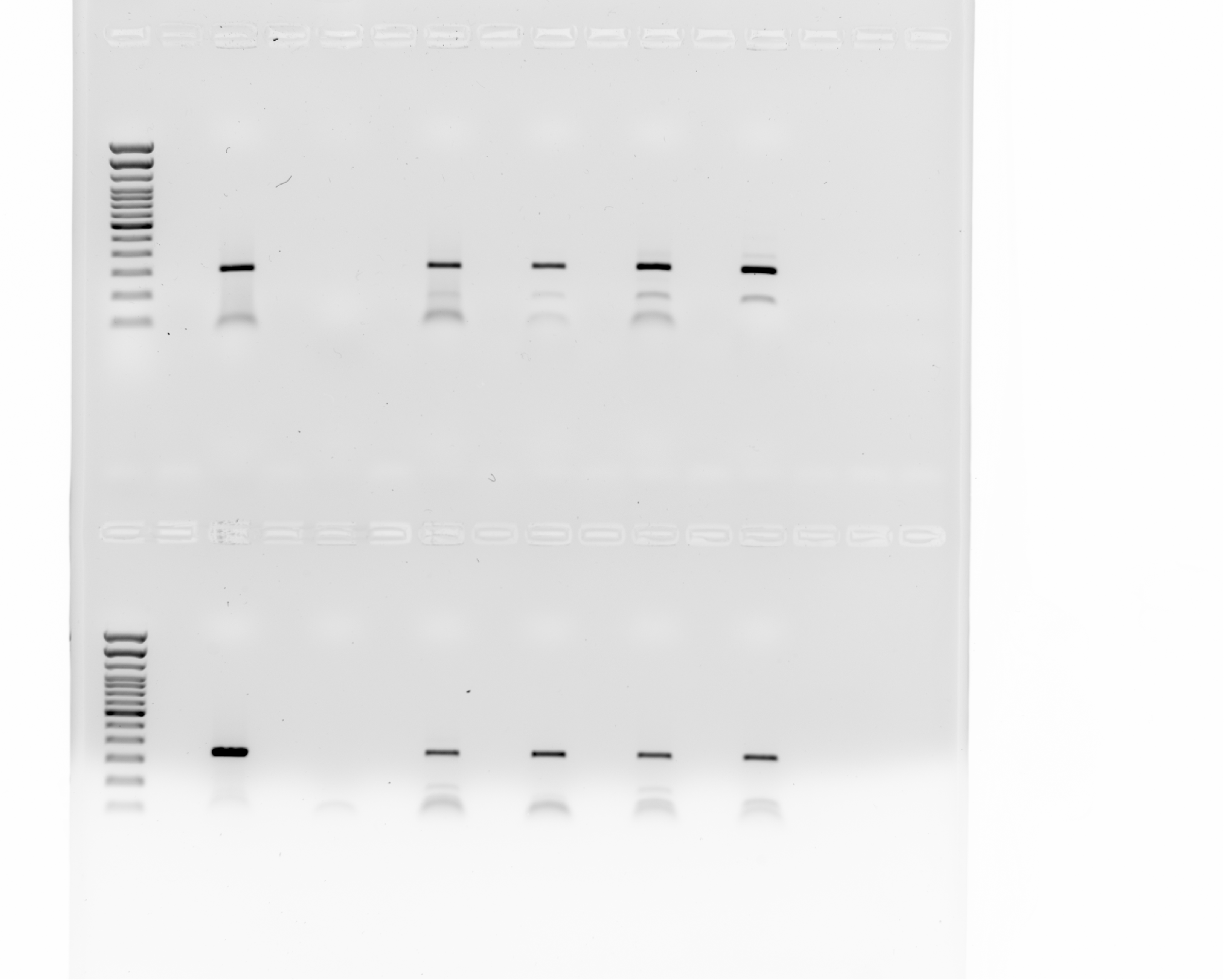

Supplement: Figure 9—source data 2. [file elife-102681-fig9-data2.zip › Figure 9-source data_2/zheng lab 2022-12-14 12h53m33s.tif]

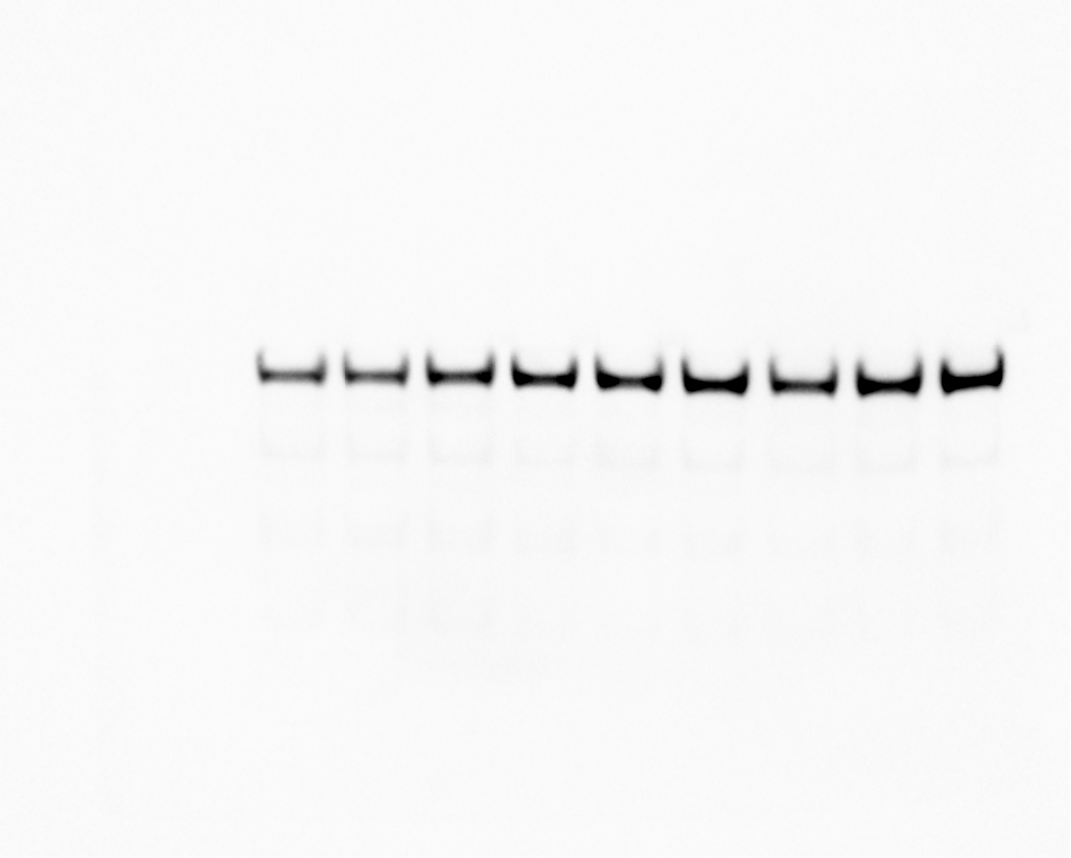

Supplement: Figure 9—source data 2. [file elife-102681-fig9-data2.zip › Figure 9-source data_2/zheng lab 2024-07-08 08h13m39s.tif]

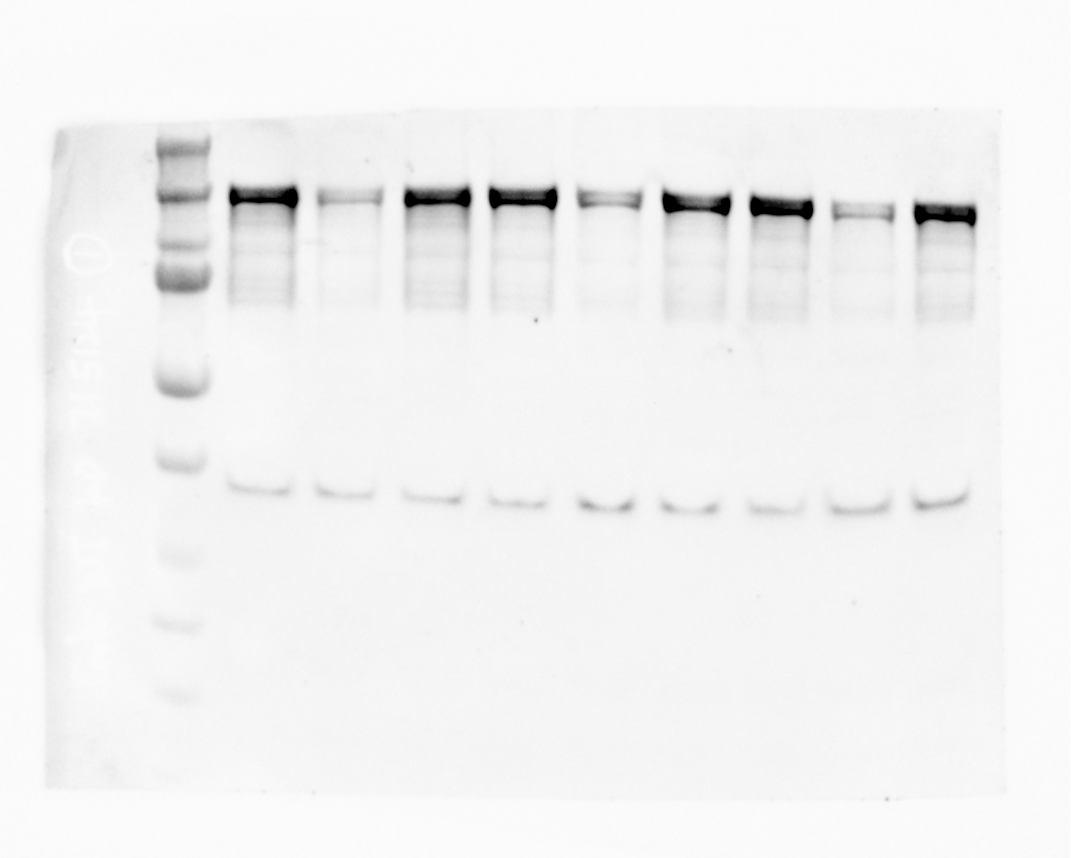

Supplement: Figure 9—source data 2. [file elife-102681-fig9-data2.zip › Figure 9-source data_2/zheng lab 2024-07-08 08h19m55s.tif]

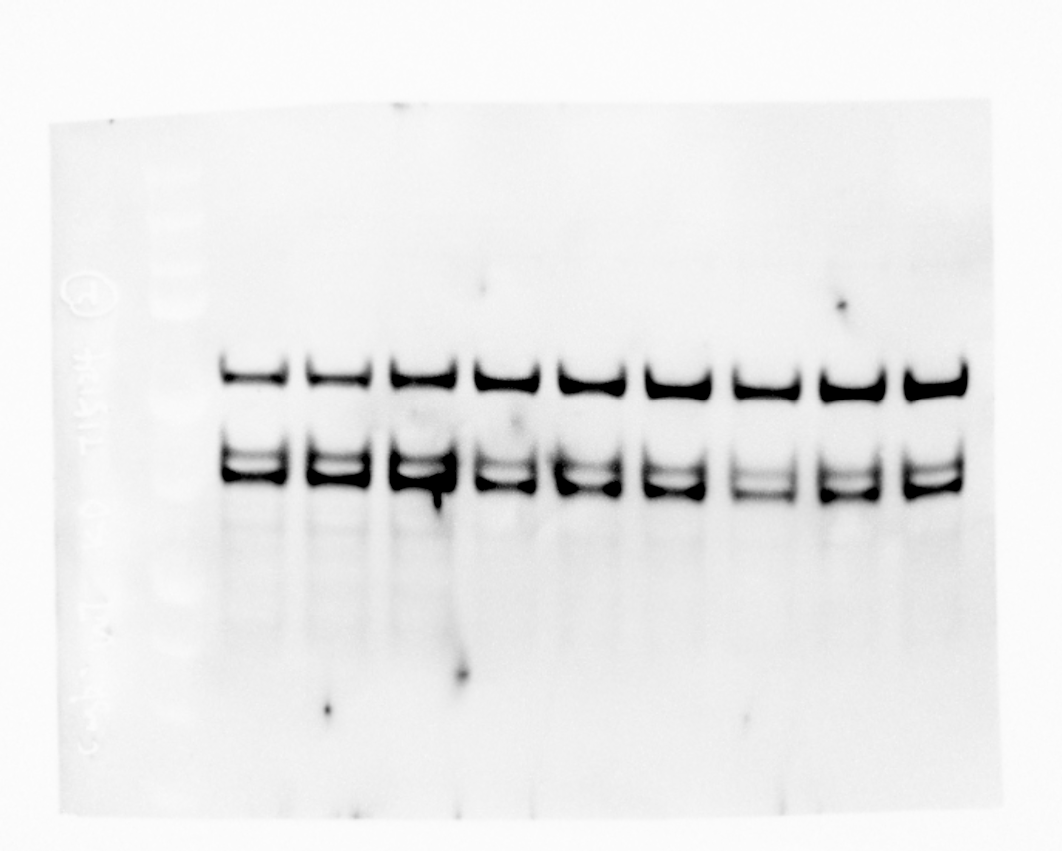

Supplement: Figure 9—source data 2. [file elife-102681-fig9-data2.zip › Figure 9-source data_2/zheng lab 2024-07-09 11h15m59s.tif]

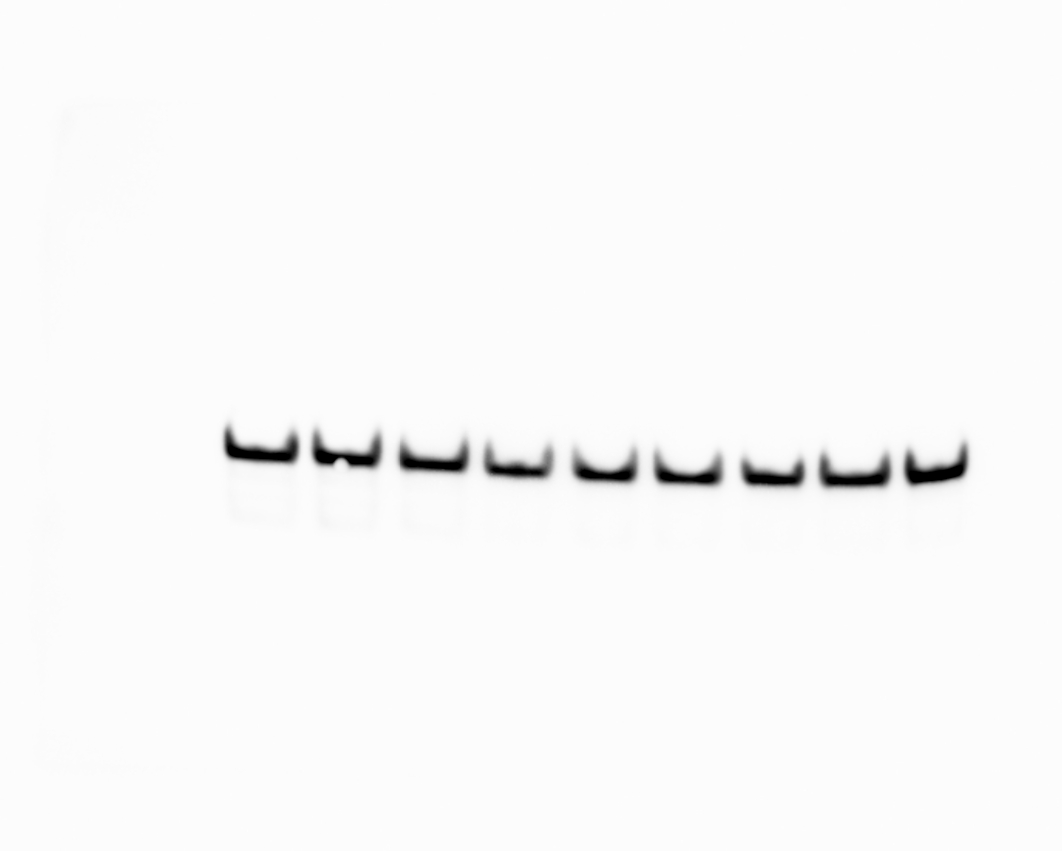

Supplement: Figure 9—source data 2. [file elife-102681-fig9-data2.zip › Figure 9-source data_2/zheng lab 2024-07-09 11h23m58s.tif]

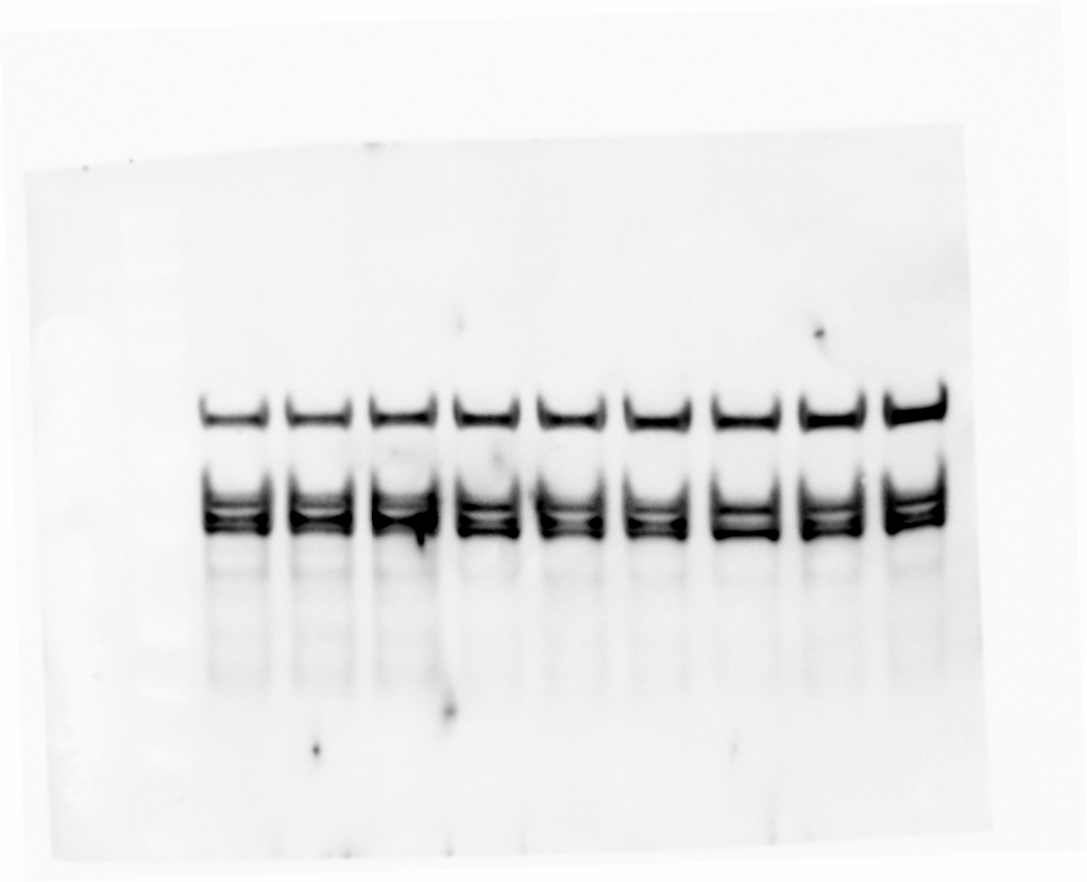

Supplement: Figure 9—source data 2. [file elife-102681-fig9-data2.zip › Figure 9-source data_2/zheng lab 2024-07-10 10h19m11s.tif]

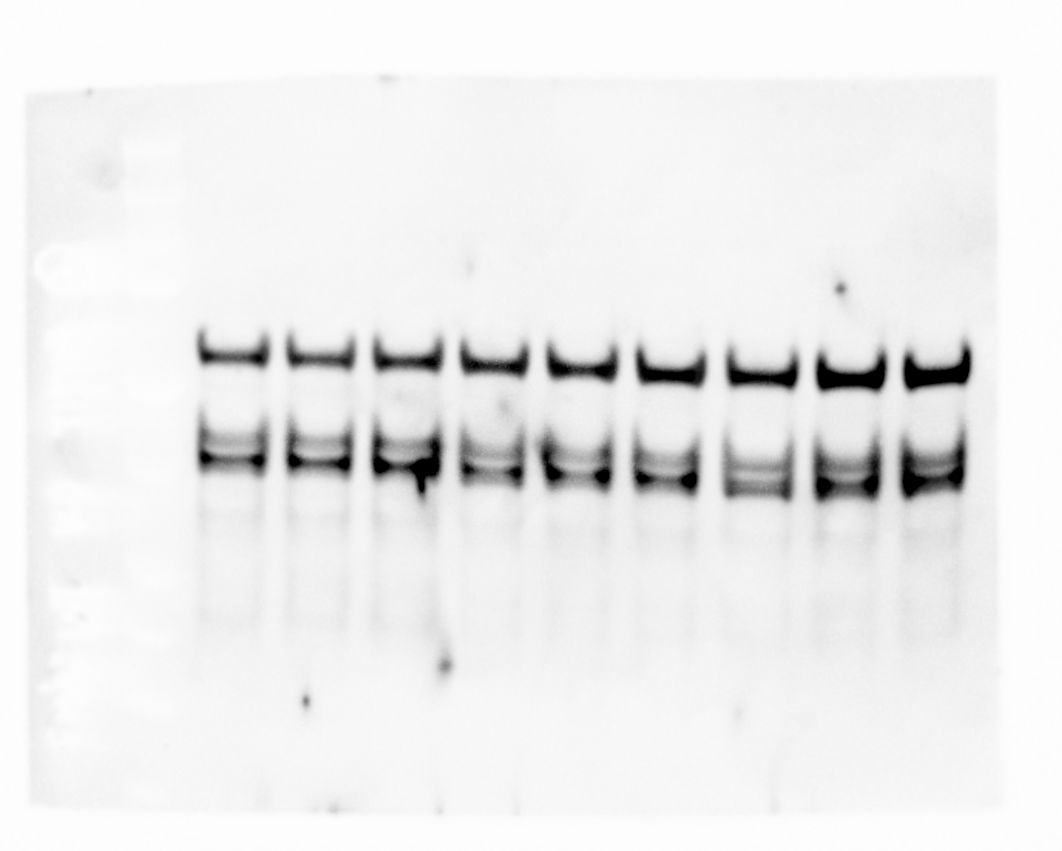

Supplement: Figure 9—source data 2. [file elife-102681-fig9-data2.zip › Figure 9-source data_2/zheng lab 2024-07-11 09h59m44s.tif]

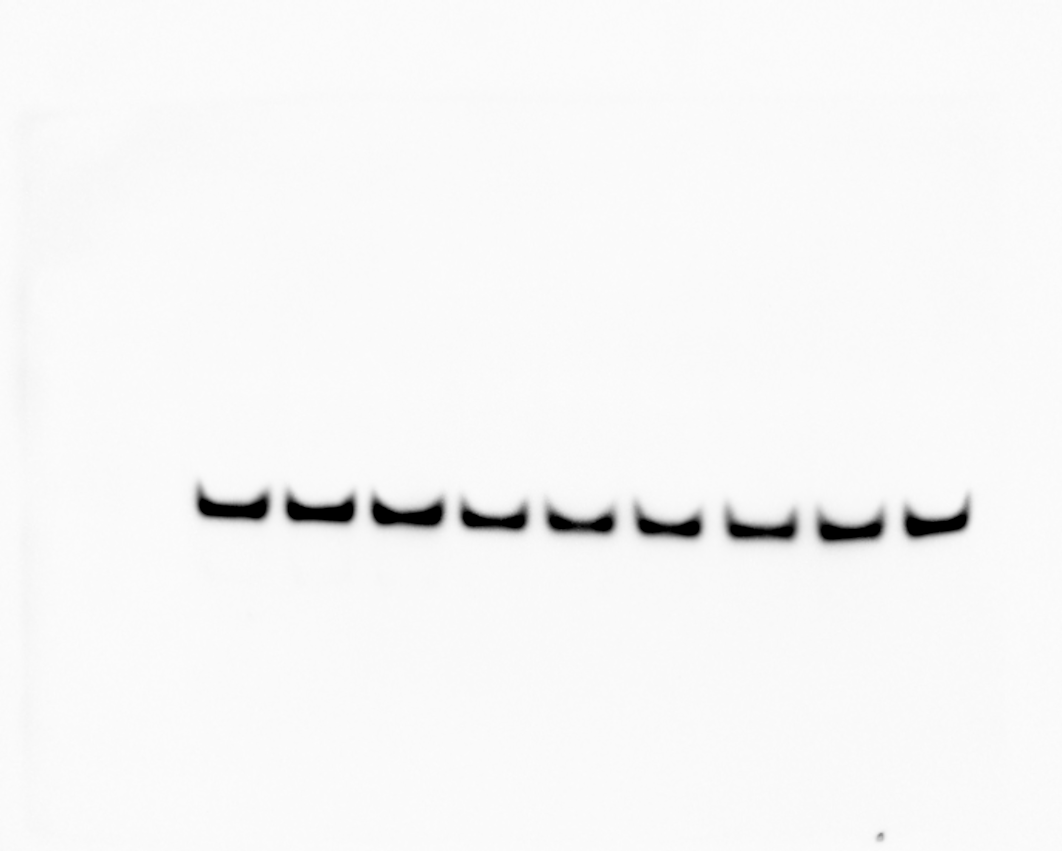

Supplement: Figure 9—source data 2. [file elife-102681-fig9-data2.zip › Figure 9-source data_2/zheng lab 2024-07-11 14h48m39s.tif]

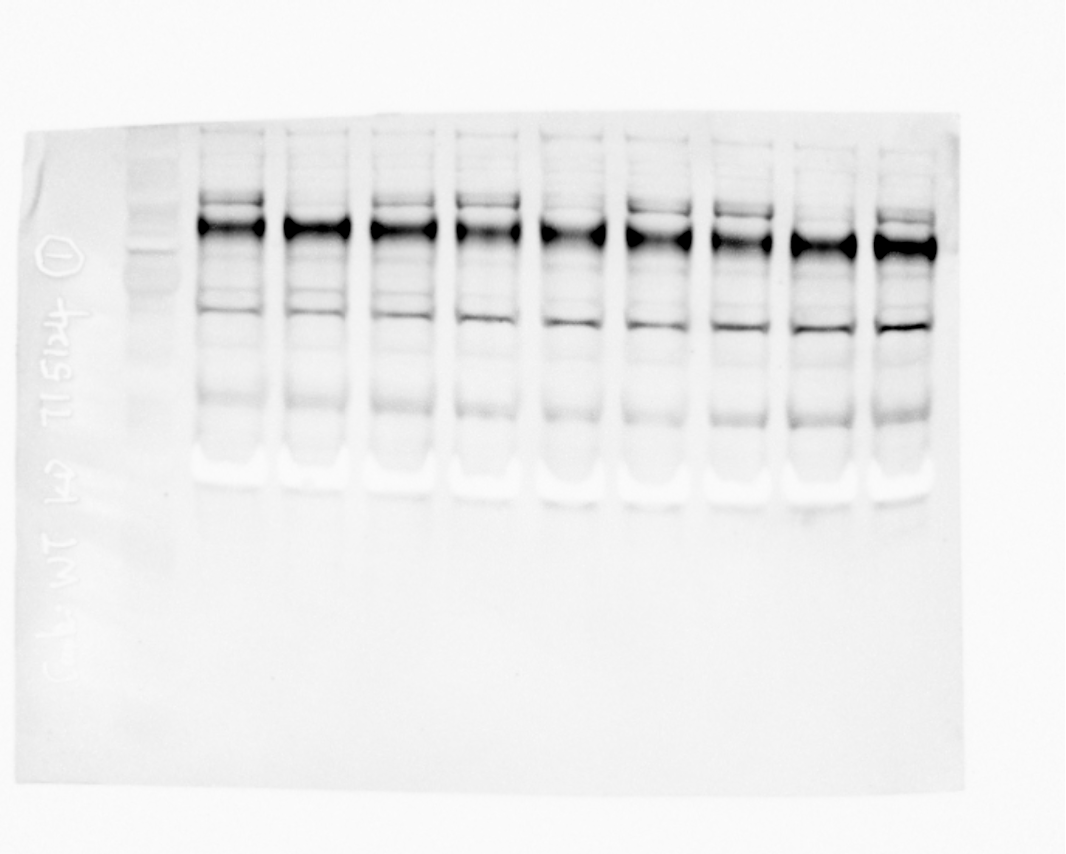

Supplement: Figure 9—source data 2. [file elife-102681-fig9-data2.zip › Figure 9-source data_2/zheng lab 2024-11-25 10h42m33s.tif]

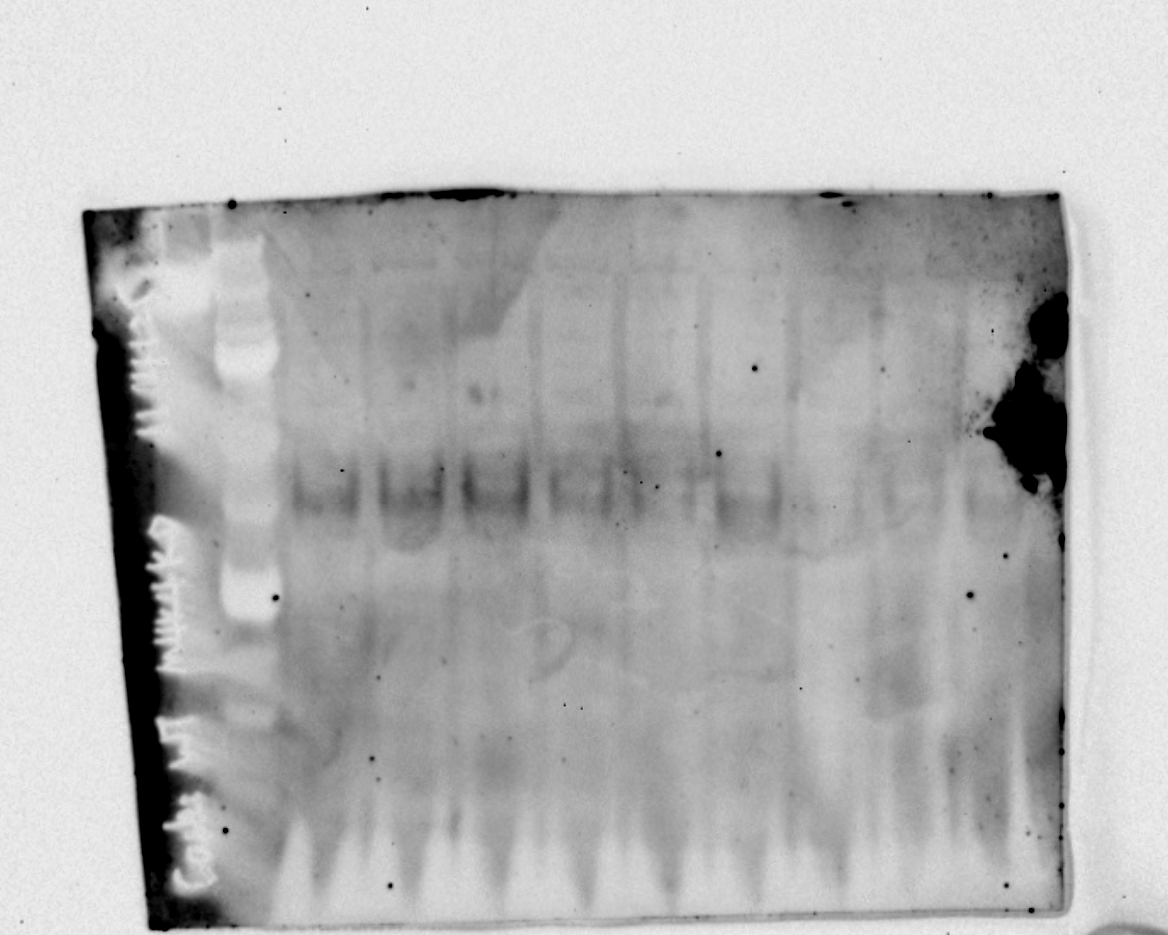

Supplement: Figure 9—source data 2. [file elife-102681-fig9-data2.zip › Figure 9-source data_2/zheng lab 2025-01-16 11h45m30s.tif]

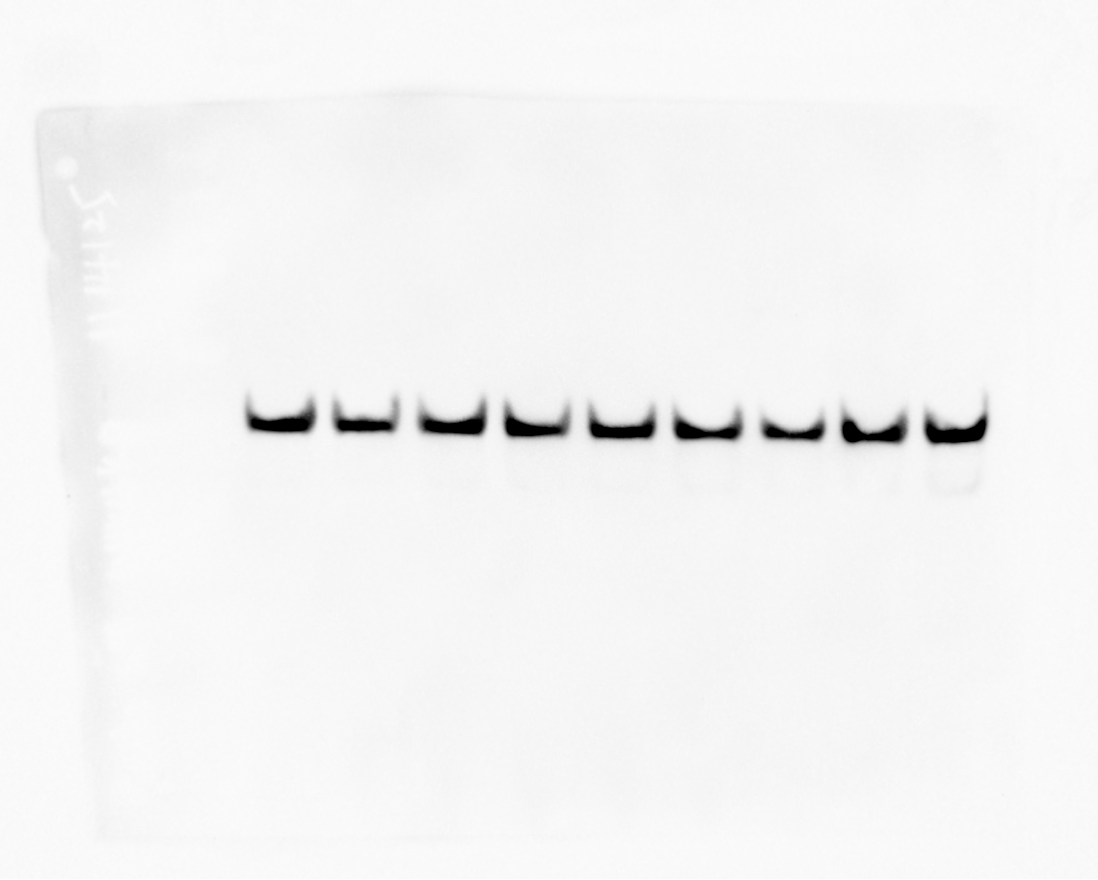

Supplement: Figure 9—source data 2. [file elife-102681-fig9-data2.zip › Figure 9-source data_2/zheng lab 2025-01-16 15h39m45s.tif]
